# The Histone Deacetylase Inhibitor Suberoylanilide Hydroxamic Acid Alleviates Salinity Stress in Cassava

**DOI:** 10.3389/fpls.2016.02039

**Published:** 2017-01-09

**Authors:** Onsaya Patanun, Minoru Ueda, Misao Itouga, Yukari Kato, Yoshinori Utsumi, Akihiro Matsui, Maho Tanaka, Chikako Utsumi, Hitoshi Sakakibara, Minoru Yoshida, Jarunya Narangajavana, Motoaki Seki

**Affiliations:** ^1^Plant Biochemistry and Molecular Genetics Laboratory, Department of Biotechnology, Faculty of Science, Mahidol UniversityBangkok, Thailand; ^2^Plant Genomic Network Research Team, RIKEN Center for Sustainable Resource ScienceYokohama, Japan; ^3^CREST, Japan Science and Technology AgencySaitama, Japan; ^4^Plant Productivity Systems Research Group, RIKEN Center for Sustainable Resource ScienceYokohama, Japan; ^5^Chemical Genomics Research Group, RIKEN Center for Sustainable Resource ScienceSaitama, Japan; ^6^Plant Genomic Network Science Division, Kihara Institute for Biological Research, Yokohama City UniversityYokohama, Japan

**Keywords:** cassava, high salinity stress, epigenetics, histone modification, suberoylanilide hydroxamic acid (SAHA)

## Abstract

Cassava (*Manihot esculenta* Crantz) demand has been rising because of its various applications. High salinity stress is a major environmental factor that interferes with normal plant growth and limits crop productivity. As well as genetic engineering to enhance stress tolerance, the use of small molecules is considered as an alternative methodology to modify plants with desired traits. The effectiveness of histone deacetylase (HDAC) inhibitors for increasing tolerance to salinity stress has recently been reported. Here we use the HDAC inhibitor, suberoylanilide hydroxamic acid (SAHA), to enhance tolerance to high salinity in cassava. Immunoblotting analysis reveals that SAHA treatment induces strong hyper-acetylation of histones H3 and H4 in roots, suggesting that SAHA functions as the HDAC inhibitor in cassava. Consistent with increased tolerance to salt stress under SAHA treatment, reduced Na^+^ content and increased K^+^/Na^+^ ratio were detected in SAHA-treated plants. Transcriptome analysis to discover mechanisms underlying salinity stress tolerance mediated through SAHA treatment reveals that SAHA enhances the expression of 421 genes in roots under normal condition, and 745 genes at 2 h and 268 genes at 24 h under both SAHA and NaCl treatment. The mRNA expression of genes, involved in phytohormone [abscisic acid (ABA), jasmonic acid (JA), ethylene, and gibberellin] biosynthesis pathways, is up-regulated after high salinity treatment in SAHA-pretreated roots. Among them, an *allene oxide cyclase* (*MeAOC4*) involved in a crucial step of JA biosynthesis is strongly up-regulated by SAHA treatment under salinity stress conditions, implying that JA pathway might contribute to increasing salinity tolerance by SAHA treatment. Our results suggest that epigenetic manipulation might enhance tolerance to high salinity stress in cassava.

## Introduction

Cassava (*Manihot esculenta* Crantz) originated in South America and is an important root crop, of which worldwide cultivation has progressed throughout tropical and subtropical regions (Olsen and Schaal, 1999). This perennial crop grows a starchy root, with starch making up 70–90% of the total dry weight (El-Sharkawy, 2004; Nuwamanya et al., 2008). Over 500 million people use cassava root starch as a source of carbohydrates (FAO, 1998; El-Sharkawy, 2004). Moreover, cassava has multiple applications including as animal feed and as a raw material for biofuel production, which contributes to building a sustainable ecosystem (Fu et al., 2016).

Soil salinity is one of the leading factors that hinder crop production globally, and development of cassava plants that are more tolerant to salinity stress is required (Carretero et al., 2008). It is well known that soil salinity affects plant cells in two ways: water deficit caused by high concentrations of salt in soil leading to decreasing water uptake by roots (osmotic stress); and high accumulation of salt in the plant, which alters Na^+^/K^+^ ratios as well as leading to excessive Na^+^ and Cl^−^ content (ion cytotoxicity; Munns and Tester, 2008; Julkowska and Testerink, 2015). Previous studies have revealed that several mechanisms such as maintenance of ion homeostasis, accumulation of compatible solutes, hormonal control, antioxidant systems, and Ca^2+^ signaling are essential for plants to survive under high salinity stress (Jia et al., 2015). Based on those findings, genetic engineering and conventional breeding have been widely used to develop salt-tolerant plants. To improve salt tolerance of transgenic plants, genes involved in several pathways against salt stress have been used as targets for expression modification. These include transporters for ion homeostasis such as NHX1 (Apse et al., 1999), SOS1/2/3 (Shi et al., 2003; Yang et al., 2009), and HKT1 (Mølle et al., 2009); and for the accumulation of osmolytes such as proline (Kishor et al., 1995) and glycine betaine (Sakamoto and Murata, 1998); late embryogenesis abundant (LEA) proteins (Xu et al., 1996); and enzymes for antioxidant synthesis such as GST/GPX (Roxas et al., 1997) and SOD (McKersie et al., 1999).

In addition to osmolytes, treatment with small molecules such as plant hormones has been used to enhance salt tolerance in plants. It is reported that the use of salicylic acid (SA) increases tolerance to drought and salt stress in wheat (Shakirova et al., 2003). In Arabidopsis, β-aminobutyric acid (BABA) functions as priming effect on abscisic acid (ABA) responses for drought and salinity stress, resulted in increasing these stress tolerance (Jakab et al., 2005). These results show that application of small molecules allows the improvement of plant traits responsible for stress tolerance, particularly for crops in which it is difficult to introduce traits by transformation or crossing. Furthermore, constitutive expression of stress responsive genes often induces growth inhibition. Treatment with small molecules during the optimized period may have the advantage of minimizing the growth inhibition, because stopping small-molecules application can release from the growth inhibition.

Histone deacetylase (HDAC) inhibitors are effective small molecules against environmental stresses. HDAC controls the level of histone acetylation with histone acetyltransferase (HAT; Seto and Yoshida, 2014; Verdin and Ott, 2015). Histone acetylation is one of the histone modifications involved in epigenetic regulation, and recent evidence has increasingly revealed that the balance of histone acetylation plays a pivotal role in response to salinity stress (Kim et al., 2015). For example, the transcriptional co-activator *ADA2b* is a component of several multiprotein HAT complexes that contain GCN5 as their catalytic subunit. The mutant *ada2b* shows hypersensitivity to salt. This result suggests that *ADA2b* is involved in the regulation of histone acetylation levels for salt-induced genes through the modulation of HAT activity (Kaldis et al., 2010). Conversely, the loss of an HDAC, HDA9, resulted in reduced sensitivity to salt and drought stresses in *Arabidopsis* (Zheng et al., 2016). These studies imply that modulation of induced histone acetylation is inextricably linked to salinity stress response, although other histone modifications such as methylation are also known to be involved (Shen et al., 2014; Kim et al., 2015; Asensi-Fabado et al., 2016). Interestingly, the HDAC inhibitor, Ky-2, can increase gene expression for an ion transporter (*SOS1*) and an enzyme involved in osmolyte accumulation (*P5CS*), leading to increased tolerance to salt stress (Sako et al., 2016). Therefore, there is the possibility that HDAC inhibitors could be used to manipulate crops and obtain desired characteristics.

In this study, we attempt to enhance tolerance to high salinity in cassava using the commercially available HDAC inhibitor suberoylanilide hydroxamic acid (SAHA, vorinostat; Dokmanovic et al., 2007). The survival rate shows that SAHA helps cassava become less sensitive to high salinity stress. Immunoblotting analysis reveals that SAHA induces hyperacetylation of histones H3 and H4 in roots. Transcriptome analysis using a microarray revealed that mRNA expression of genes, involved in phytohormone ABA and jasmonic acid (JA) biosynthesis pathways, is strongly induced under high salinity stress condition. In addition to above two phytophormone pathways, SAHA treatment is likely to enhance ethylene and gibberellin biosynthesis pathways. Among them, an *allene oxide cyclase* (*MeAOC4*) catalyzing a crucial step in JA biosynthesis is strongly up-regulated. We discuss which pathway plays a pivotal role in inducing tolerance to salinity stress by SAHA treatment in cassava. Taken together, our results provide fundamental information on the high salinity stress response and suggest the possibility that the epigenetic manipulation via HDAC inhibition might be applicable for increasing tolerance to high salinity stress in cassava.

## Materials and methods

### Plant materials and growth conditions

Cassava cultivar TMS60444 was asexually propagated in a plastic pot with Murashige and Skoog (MS) media (pH5.8, KOH) containing 4.4 g L^−1^ MS salts (Murashige and Skoog, 1962), 20 g L^−1^ sucrose, 2 μM CuSO_4_, and 3 g L^−1^ Gelrite (Wako) under a light intensity of 40–80 μmol photons m^−2^s^−1^ with a 12 h/12 h photoperiod at 28 ± 1°C. The 5-cm length shoot tops of 3-month-old cassava plantlets were cut and then transferred to liquid MS media without agar. After 1 week incubation, the plantlets showing root elongation were subjected to each analysis as follows.

### Effect of SAHA treatment on survival rate and biomass under salinity stress condition

To reveal to which extent SAHA treatment alleviates cassava plants from salinity stress, survival rate and biomass (fresh and dry weight) were measured as follows. At 1 week-incubation after the transfer of cut plantlets to liquid medium, the plantlets were incubated on liquid MS media supplemented with 100 μM SAHA (suberoylanilide hydroxamic acid: N-hydroxy-N′-phenyloctanediamide: vorinostat, from Tokyo Chemical Industry, code-H1388) for 24 h. SAHA was dissolved in dimethylsulfoxide (DMSO) and DMSO treatment served as the control for all analyses. Then, the plantlets were transferred to 200 mM NaCl containing liquid MS media for 10 days and subsequently transferred to normal liquid MS medium for 1 month to observe phenotype and count survival rate in each condition. At least 13 cassava plantlets were used for each condition. The survival rate was based on the percentage of surviving plants. Plants with regenerated leaves and green stems were counted as surviving plants, while the plants with white stems having no new leaves were counted as dead ones. The fresh weight (FW) of root and shoot samples was measured immediately after harvesting. To measure the dry weight (DW), cassava samples were incubated at 60°C for 1 week. FW and DW were measured from the regenerated plants that had 3 or more leaves. Three biological replicates were conducted for all analyses.

### Measurement of Na^+^ and K^+^ contents

The cassava samples were washed for 5 s three times using distilled water. Stems and leaves were cut separately using mineral-free carbon knives (Feather 5) and kept at −80°C until use. Analytical measurement of ^23^Na and ^39^K content was conducted using an inductively coupled plasma mass spectrometer (ICP-MS; NexION300, Perkin Elmer). Firstly, the cassava samples were dried using a freeze dryer for 3 days. The cassava sample weight was measured. Then 5 mL of conc. HNO_3_ was added to the cassava samples. The cassava samples were incubated with concentrated HNO_3_ overnight. After that, the cassava samples were digested using a microwave sample preparation system (MultiWave-3000, Perkin Elmer). All samples were completely digested prior to running on the ICP-MS. The digested samples were adjusted to a volume of 50 mL with Milli-Q water, then filtered through 5B filter paper (Advantec). The ICP-MS analysis was carried out as described previously (Itouga et al., 2014). Three independent biological replicates were generated for each condition. Na^+^ and K^+^ content were analyzed statistically with a *t*-test.

### Protein extraction and immunoblotting analysis

The leaf and root samples were ground using a Multi-beads shocker (Yasui Kikai) and 200 μL of 2 × Laemmli sample buffer [2% SDS, 50.4% (w/v) glycerol, 0.02 M Tris-HCl (pH 6.8) with 2% 2-mercaptoethenol] was immediately added to the powder. The mixture was heated at 95°C for 3 min then placed on ice for 5 min, and centrifuged at 4°C for 10 min at 14,000 × g. The protein supernatant was transferred to a new tube, and separated by 12.5% SDS-PAGE gel electrophoresis. After gel electrophoresis, the protein was blotted on equilibrated Immobilon-P PVDF membrane (Millipore). Then the blotted membrane was blocked using 5% skim milk at room temperature for 1 h. Next, the membrane was incubated at 4°C overnight for the primary antigen–antibody interaction. Secondary antibodies were incubated with the membrane for 1 h at room temperature. Finally, detection was carried out using an ImageQuant LAS 4000 (GE). Primary antibodies were H3 (Abcam, 1791), H4 (Abcam, 10158), acH3 (Merck Millipore, 06-599), and acH4 (Merck Millipore, 06-866), diluted 1:5000, 1:3000, 1:2000, and 1:5000, respectively. The secondary antibody was goat anti-rabbit IgG (GE, NA931), diluted 1:25,000.

### RNA extraction

Total RNA was extracted using Plant RNA Isolation Reagent (Invitrogen) following spin-column RNA precipitation using RNeasy Plant Mini Kit (Qiagen). Briefly, the sample tissues were ground into fine powder using a Multi-beads shocker. The tissue powder was used for RNA extraction using Plant RNA Isolation Reagent according to the manufacturer's instruction (Invitrogen). To clean the RNA solution, after adding 20 μL of RNase-free water to the RNA pellet, the RNA solution was purified using spin-column RNA precipitation followed by addition of RNase-Free DNase (Qiagen). In order to remove contaminated DNA completely, the process for RNA purification using spin-column RNA precipitation with DNase treatment was repeated twice. All samples were stored at −80°C until use.

### Microarray analysis

For microarray analysis, the plantlets were treated with 100 μM SAHA for 24 h, followed by treatment with 200 mM NaCl for 2 or 24 h. The quality of total RNA was evaluated using a Bioanalyzer system (Agilent). Microarray analysis was performed according to the procedure of Utsumi et al. (2012). Briefly, total RNA was labeled with cyanin-3 (Cy3) using the Quick Amp Labeling kit (Agilent Technologies). Next, the Cy3-labeled cRNA was fragmented and hybridized to the Agilent microarray (GPL14139; Utsumi et al., 2016). After hybridization, the microarrays were washed and immediately scanned on an Agilent DNA Microarray Scanner (G2505B) using one color scan setting for 8 × 60 K array slides. Feature Extraction software (Ver. 9.1, Agilent Technologies) was used to process the digital images. Three independent experiments were performed for each condition. After normalization, microarray probes were detected based on one-way ANOVA with Benjamini–Hochberg correction (FDR) < 0.0001. An AGI gene code is shown in Tables 1–**6**, if the protein encoded in each gene (probe ID) has the sequence similarity with those in *Arabidopsis* at *E* ≤ 1 × 10^−5^. The *E*-value for each probe ID is available in Tables S1–S5. Gene ontology (GO) analyses were carried out using agriGO at http://bioinfo.cau.edu.cn/agriGO/. The information from the microarray data is available on the GEO website (GEO ID: GSE84715).

**Table 1:** **List of top 40 cassava genes upregulated (log_**2**_ ratio > 1; FDR < 0.0001) in roots by treatment with 200 mM NaCl for 2 h in non-SAHA-pretreated plants**.

**ProbeID**	**AGI code^a^**	**Encoded proteins/other features^b^**	**w/o SAHA^c^**	**FDR**
			**log_2_ ratio (2 h NaCl/0 h NaCl)**	
RknMes02_006505	AT1G52690	Late embryogenesis abundant protein (LEA) family protein	10.250	3.36E-06
RknMes02_053823	AT2G42850	Cytochrome P450, family 718	8.743	2.44E-07
RknMes02_033569	AT3G63060	EID1-like 3	8.738	3.62E-05
RknMes02_025528	AT3G14440	Nine-cis-epoxycarotenoid dioxygenase 3	8.526	4.44E-05
RknMes02_038832	AT3G24310	Myb domain protein 305	8.298	1.01E-05
RknMes02_057161	AT4G33467	Unknown protein	8.062	1.13E-05
RknMes02_052347	AT3G51810	Stress induced protein	6.657	7.15E-05
RknMes02_056041	AT3G30210	Myb domain protein 121	6.620	1.01E-06
RknMes02_051689	AT4G35680	Arabidopsis protein of unknown function (DUF241)	6.399	1.88E-05
RknMes02_039694	AT3G03341	Unknown protein	6.309	6.19E-06
RknMes02_008877	AT4G31240	Protein kinase C-like zinc finger protein	5.793	7.87E-06
RknMes02_003939	AT5G51760	ABA-hypersensitive germination 1	5.679	5.34E-05
RknMes02_027987	AT1G60420	DC1 domain-containing protein	5.659	6.85E-06
RknMes02_056865	AT3G61060	Phloem protein 2-A13	5.645	2.96E-05
RknMes02_055843	AT5G59190	Subtilase family protein	5.563	2.73E-05
RknMes02_054690	AT4G11360	RING/U-box superfamily protein	5.531	4.68E-06
RknMes02_026274	AT5G40390	Raffinose synthase family protein	5.531	3.30E-06
RknMes02_055373	AT3G55646	Unknown protein	5.518	1.07E-05
RknMes02_009395	AT2G40170	Stress induced protein	5.427	9.73E-05
RknMes02_056706	AT1G11530	C-terminal cysteine residue is changed to a serine 1	5.401	1.08E-05
RknMes02_028787	AT1G19640	Jasmonic acid carboxyl methyltransferase	5.366	3.94E-06
RknMes02_017023	AT4G16160	ATOEP16-2, ATOEP16-S	5.332	8.36E-07
RknMes02_019566	AT4G27450	Aluminum induced protein with YGL and LRDR motifs	5.239	3.25E-05
RknMes02_010142	AT5G66110	Heavy metal transport/detoxification superfamily protein	5.097	5.83E-06
RknMes02_002573	AT5G59220	Highly ABA-induced PP2C gene 1	5.084	4.68E-05
RknMes02_055268	AT4G25410	Basic helix-loop-helix (bHLH) DNA-binding superfamily protein	5.070	1.15E-05
RknMes02_031261	AT4G27410	Responsive desiccation 26	5.040	1.01E-05
RknMes02_010484	AT5G57050	ABA insensitive 2	5.009	3.93E-05
RknMes02_006418	AT1G18100	Mother of FT and TFL1	4.998	4.15E-06
RknMes02_013801		Ethylene-responsive transcription factor15-related	4.960	4.62E-05
RknMes02_034838	AT2G29380	Highly ABA-induced PP2C gene 3	4.949	1.08E-05
RknMes02_006068	AT1G07430	Highly ABA-induced PP2C gene 2	4.937	7.43E-06
RknMes02_038687	AT5G42290	Transcription activator-related	4.840	8.20E-07
RknMes02_051430	AT5G23960	Terpene synthase 21	4.821	2.45E-05
RknMes02_035128	AT1G44446	Arabidopsis thaliana Chlorophyll A Oxygenase	4.785	8.07E-05
RknMes02_056689	AT3G59850	Pectin lyase-like superfamily protein	4.766	4.17E-06
RknMes02_023528		Protein phosphate 2C 3-related	4.718	2.74E-05
RknMes02_036024	AT5G64750	ABA repressor1	4.671	5.55E-05
RknMes02_018746		Protein phosphatase 2C	4.638	6.38E-05
RknMes02_047856	AT5G03850	Nucleic acid-binding, OB-fold-like protein	4.621	5.20E-05

a *AGI code is shown if proteins encoded in each cassava gene (probe ID) have high amino acid sequence similarity (E ≤ 10^−5^) to Arabidopsis homologs*.

b *Encoded proteins/other features indicate the putative functions of the gene products that are expected from sequence similarity. The information for the NCBI protein reference sequence with the highest sequence similarity to the probes is shown*.

c *Plants that were not pretreated with SAHA were used*.

### Quantitative real-time RT-PCR (qRT-PCR) analysis

First-strand cDNA was synthesized from 500 ng total RNA with random primers. ReverTra Ace (TOYOBO) was used for the reverse transcription reaction according to the manufacturer's instructions. Transcript levels were assayed using Fast SYBR Green Master Mix (Applied Biosystems) and a StepOnePlus Real-Time PCR System (Applied Biosystems) according to the manufacturer's protocols. Gene-specific primers were designed using the PrimerQuest tool (http://sg.idtdna.com/primerquest/Home/Index). Melting curve analysis was conducted to validate the specificity of the PCR amplification. For qRT-PCR, three biological replicates were conducted. Actin was used as reference gene to normalize data (cassava4.1_009807m). The relevant primers are listed in Table S6. Three independent biological replicates were generated for each condition. Changes in gene expression were analyzed statistically with a *t*-test.

### Construction of phylogenetic tree

An evolutionary tree was constructed using the maximum likelihood method in MEGA7 (Kumar et al., 2016). The tree was evaluated with 1000 bootstrap replicates.

## Results

### SAHA enhances salinity stress tolerance in cassava

**Figure 1:**
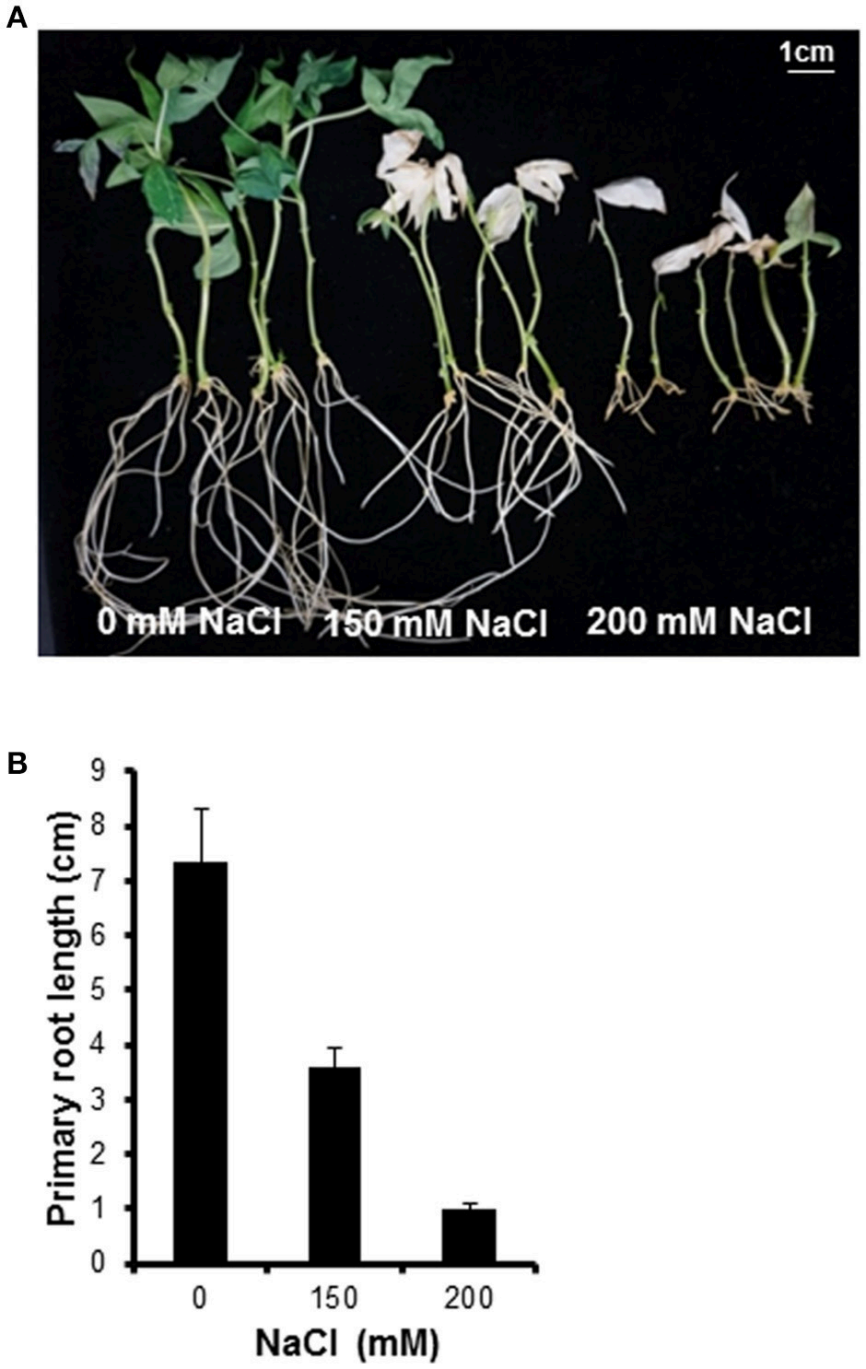
**Plant phenotype after treating with various NaCl concentrations. (A)** Morphological observation of cassava after 14-day salt stress treatment. NaCl concentrations of 0, 150, and 200 mM were used. **(B)** Inhibition of primary root elongation by salinity stress. Error bars represent the means ± SD (*n* = 5–6).

To confirm which NaCl concentration inhibits growth in cassava, plantlets were incubated in liquid medium containing different NaCl concentrations. A concentration of 200 mM NaCl clearly inhibited the growth of cassava plantlets (Figure 1A). After transfer to 200 mM NaCl salt medium for 1 week, the plantlets' older leaves started to turn yellow. At 2 weeks after transfer to salt medium, leaves showed chlorosis at both 150 and 200 mM NaCl. After 14-d incubation with 0, 150, and 200 mM NaCl, plants showed root growth of 7.4 ± 2.1, 3.6 ± 0.8, and 1 ± 0.2 cm, respectively (Figure 1B). The 200 mM NaCl concentration was selected for further experiments.

**Figure 2:**
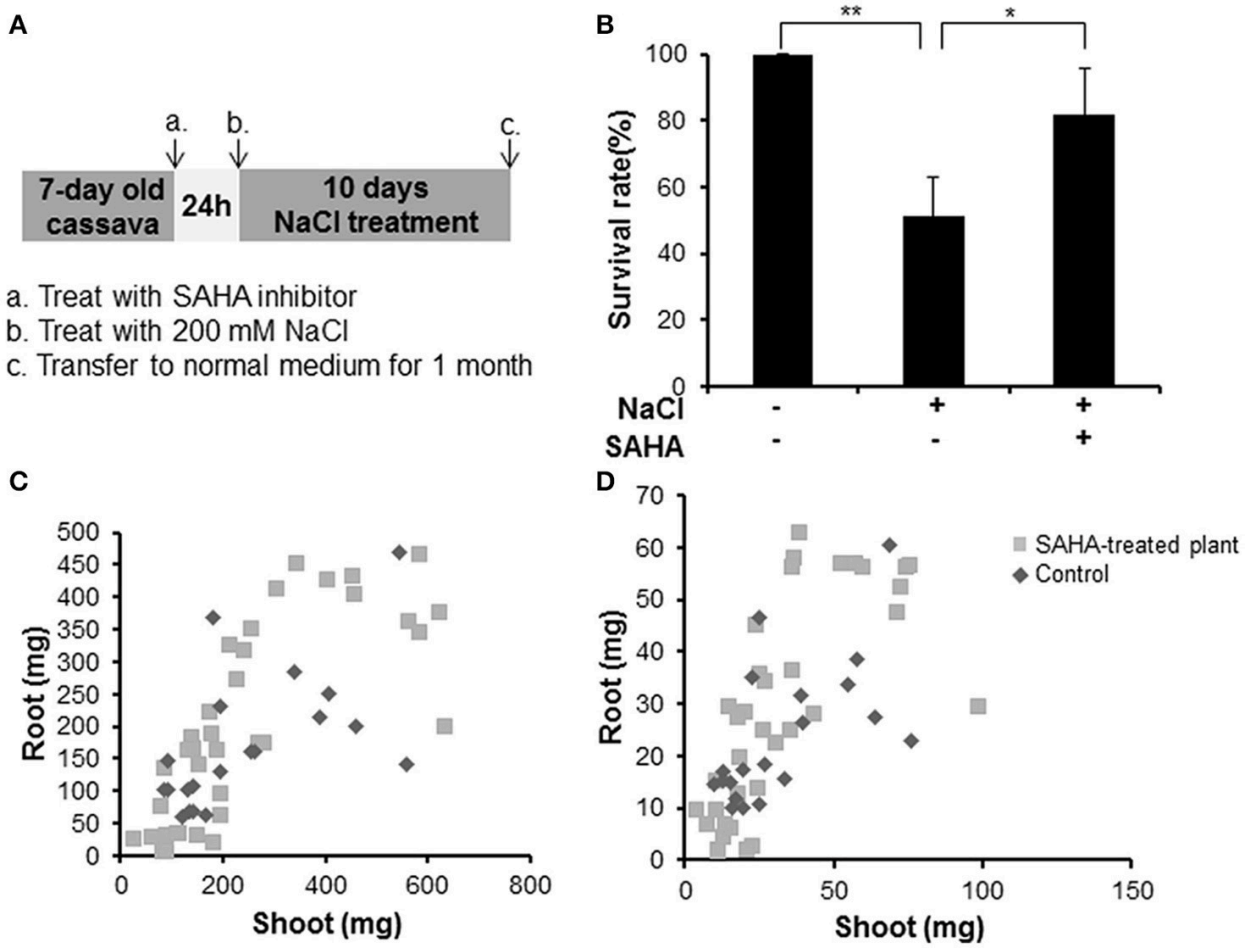
**Increase in survival rate and plant weight by SAHA treatment under high salinity stress conditions in cassava. (A)** Experimental condition. Cassava plantlets were treated with 100 μM SAHA for 24 h then subjected to 200 mM NaCl medium for 10 days. The survival rate was counted after transferring cassava to normal medium for 1 month. **(B)** Survival rate under high salinity stress of non-treated plants and SAHA-treated plants. Three independent experiments were performed (at least 13 plants per experiment). **(C,D)** Comparison of plant fresh weight **(C)** and dry weight **(D)** between non-treated plants and SAHA-treated plants. Asterisks indicate significantly different means (^*^p < 0.05, ^**^p < 0.005) as determined with a *t*-test. Error bars represent the mean ± SD.

To analyze whether SAHA treatment enhances tolerance to salinity stress in cassava, we counted the survival rate of SAHA-treated plants (Figure 2A). SAHA-treated plants showed a 30.6% higher survival rate than non-treated plants under salinity stress conditions (Figure 2B). Fresh and dry weight measurement revealed that SAHA treatment increased biomass in roots (Figures 2C,D). These results suggest that SAHA treatment improves tolerance to high salinity stress in cassava.

### Concentration of Na^+^ and K^+^ in stems and leaves under high salinity stress

**Figure 3:**
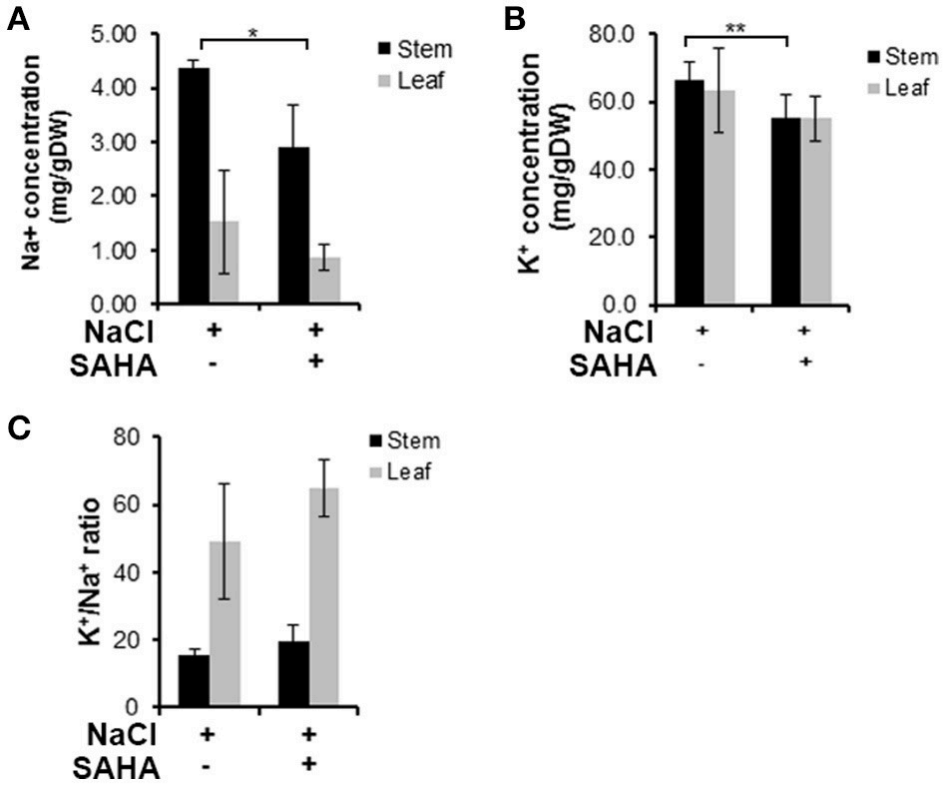
**Na^**+**^ and K^**+**^ content in stems and leaves under high salinity stress. (A,B)** Na^+^ and K^+^ concentrations in the stems and leaves of plants exposed to 100 μM SAHA then subjected to 200 mM NaCl for 2 h. **(C)** the K^+^/Na^+^ ratio of stems and leaves of cassava plants. Asterisks indicate significantly different means (^*^p < 0.02, ^**^p < 0.05) as determined with a *t*-test. Error bars represent the mean ± SD. Three independent biological replicates were performed for each condition.

The concentration of sodium (^23^Na) and potassium (^39^K) in stem and leaf samples was quantitatively analyzed by ICP-MS to reveal the extent of SAHA treatment's influence on ion homeostasis under salinity stress conditions. SAHA-treated plants had reduced Na^+^ concentrations in both stems (2.9 mg g^−1^ DW) and leaves (0.87 mg g^−1^ DW) compared with those in control plants (stems and leaves: 4.36 and 1.52 mg g^−1^ DW, respectively; Figure 3A). However, there was no obvious difference in K^+^ concentrations in SAHA-treated plants and control plants, resulting in higher K^+^/Na^+^ ratios in SAHA-treated plants than in control plants (Figures 3B,C). These results suggest that SAHA treatment allows cassava to control ion homeostasis under high salinity stress conditions.

### Immunoblotting analysis of histones H3 and H4 acetylation

**Figure 4:**
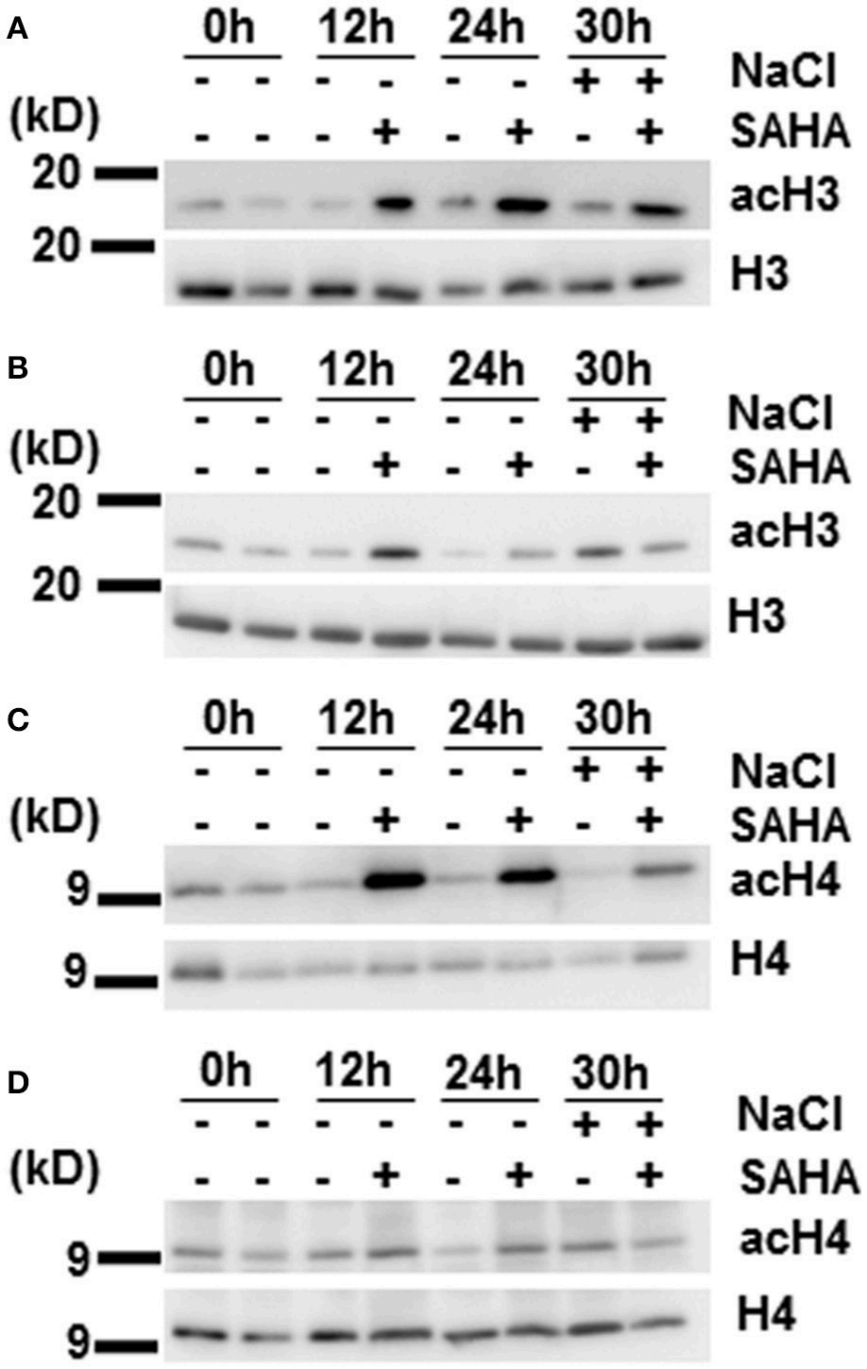
**Changes in histones H3 and H4 acetylation levels during SAHA-treatment**. Cassava plantlets were treated with 100 μM SAHA for 12 and 24 h. The 24 h SAHA treated-plants were the subjected to 200 mM NaCl for 6 h. **(A,B)** Detection of histone acetylation levels by western blotting (WB) with anti-AcH3 antibody in root **(A)** and leaf **(B)** samples. **(C,D)** The detection of histone acetylation levels by WB with anti-AcH4 antibody in root **(C)** and leaf **(D)** samples.

It has so far been unclear whether SAHA functions as the HDAC inhibitor in plants. We performed immunoblotting analysis to determine whether SAHA has an effect on histone acetylation levels. Cassava plantlets treated with 100 μM SAHA for 12 or 24 h, and cassava plantlets treated with 100 μM SAHA for 24 h then subjected to 200 mM NaCl for 6 h, were used for detecting histone acetylation status. Hyperacetylation of histones H3 and H4 was detected in the SAHA-treated plants compared with the control plants, especially in root samples (Figures 4A,C). Levels of histone acetylation were significantly increased at 12 and 24 h of SAHA treatment and remained higher up to 30 h after additional NaCl treatment. In contrast to roots, the acetylation level of histone H3 in leaf samples was slightly induced at 12 h and returned to a level similar to that of non-SAHA treated plants at 24 h (Figure 4B). There was no difference in acetylation levels of histone H4 in leaves between SAHA-treated plants and control plants (Figure 4D). These results suggest that SAHA mainly induces hyperacetylation of histones H3 and H4 in the roots, which may lead to transcriptional changes that enhance tolerance to high salinity stress in cassava.

### Transcriptome analysis in response to salinity stress

**Figure 5:**
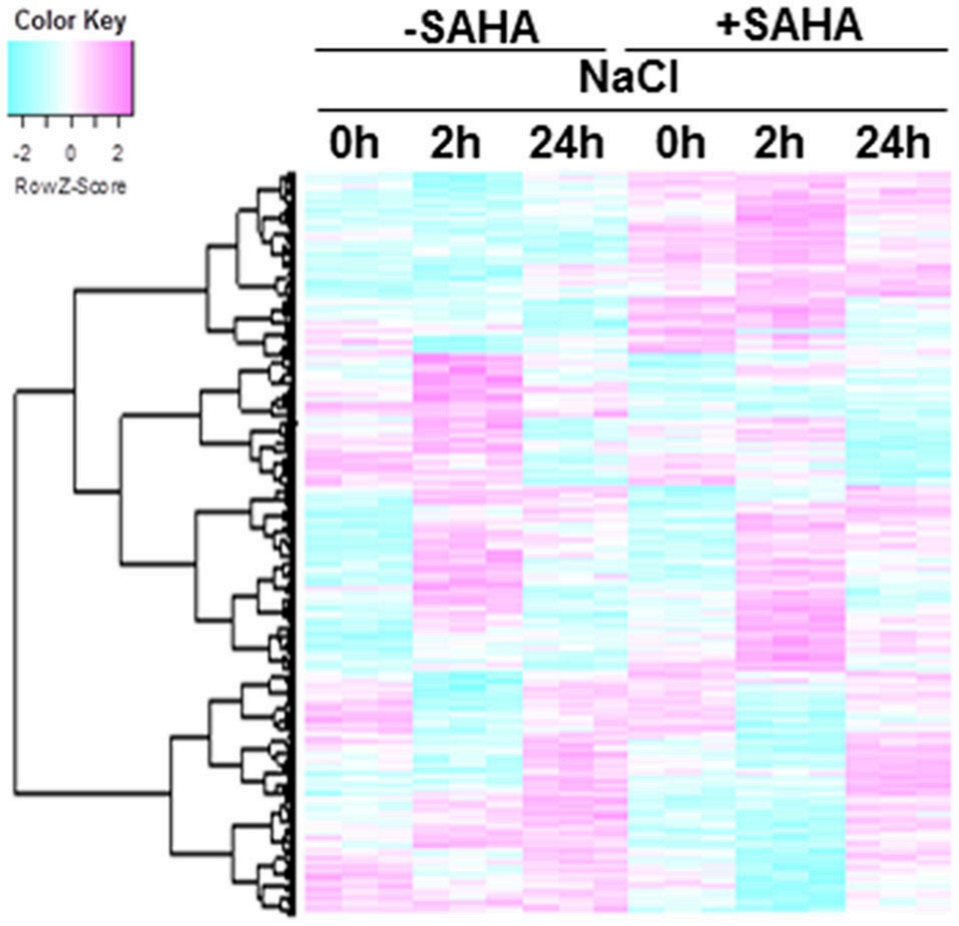
**Hierarchical cluster analysis of salt-responsive genes and SAHA-responsive genes in cassava roots**. The expression profiles of cassava genes were obtained from cassava plantlets treated with 100 μM SAHA for 24 h then subjected with 200 mM NaCl for 2 and 24 h. Transcript data was generated from 3 replicates. The heat map represents the Z-score. The key shows the Z-score region from −2 to 2. Red represents up-regulated genes while blue represents down-regulated genes. The 4389 differentially expressed genes were detected using one-way ANOVA, BH FDR < 0.0001.

To understand responses to salt stress and unveil the mechanism underlying increased tolerance to salinity stress under SAHA treatment in cassava, the transcriptional changes in roots and leaves were analyzed. For root samples, there were 4389 genes with significant expression changes [one-way ANOVA with Benjamini–Hochberg correction (FDR) < 0.0001] in at least one condition (Figure 5). In contrast, only 53 genes showed significant differences in mRNA expression in leaves (Table S7). These data are consistent with the results of immunoblotting analysis.

**Figure 6:**
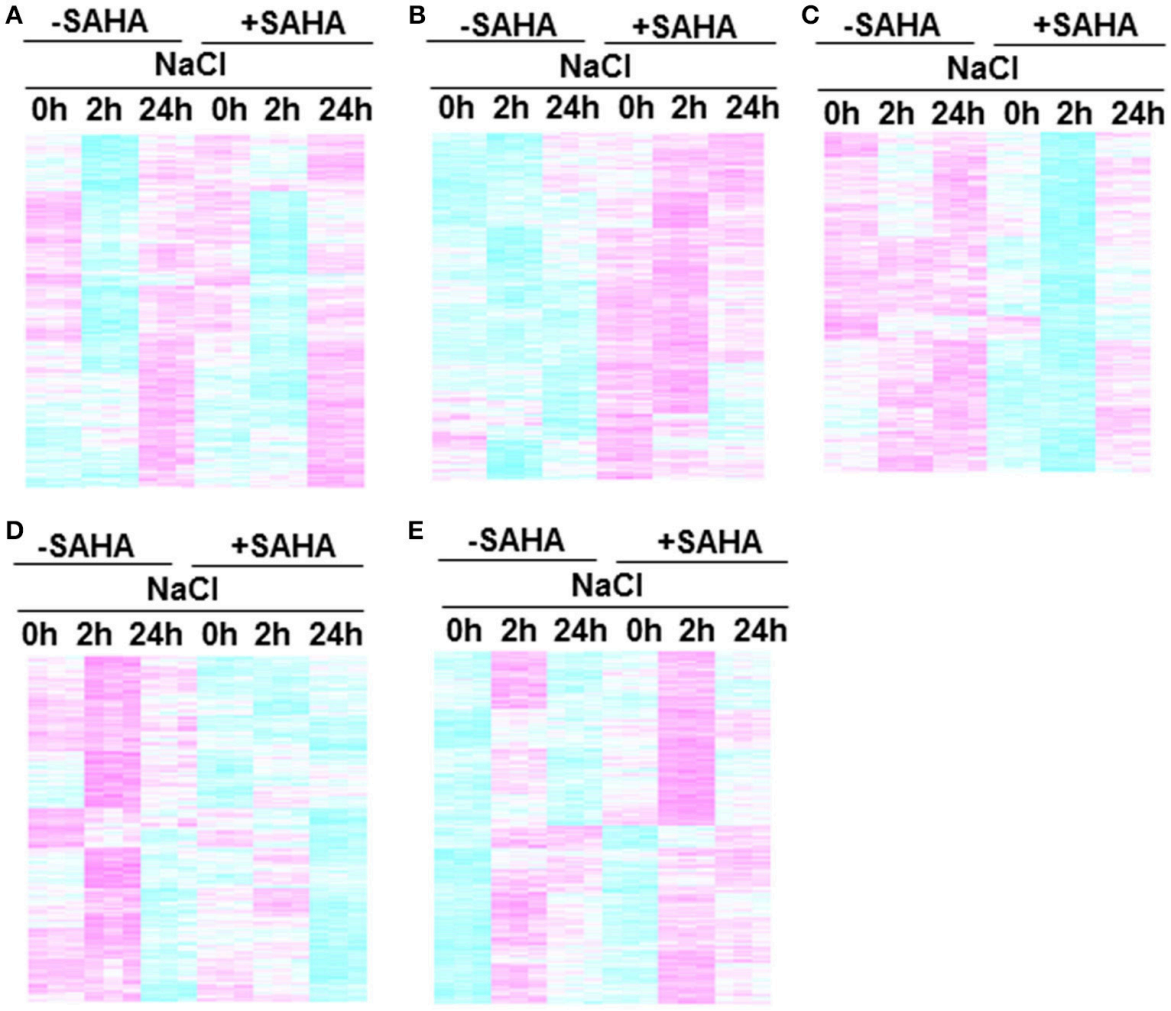
**Clustering of genes in root transcriptome**. Each cluster includes genes that responded to treatment conditions: **(A)** Class 1, SAHA-unaffected genes; **(B)** Class 2, genes up-regulated by SAHA treatment; **(C)** Class 3, enriched salt-stress unresponsive genes down-regulated at 2 h after NaCl addition in SAHA-pretreated plants **(D)** Class 4, enriched salt-stress responsive genes down-regulated at 2 h after NaCl addition in SAHA-pretreated plants, **(E)** Class 5, salt-responsive genes up-regulated at 2 h after NaCl addition in SAHA-pretreated plants.

The heatmap shows significant differences among treatment conditions in root samples. According to their expression profiles, genes differentially expressed in roots can be divided into five classes (Figures 6A–E). GO analysis was used to gain an overview of the functions of genes in each class. Class 1 contains SAHA-unaffected genes with functions assigned to GO:0000003 (reproduction), GO:0009791 (post-embryonic development), and GO:0008152 (metabolic process; Figure 6A). Class 2 contains genes up-regulated by SAHA treatment. GO enrichment analysis revealed that the majority of the genes in this group belong to GO:0050896 (response to stimulus), GO:0044237 (cellular metabolic process), and GO:0044238 (primary metabolic process; Figure 6B). Classes 3 and 4 contain genes down-regulated by the effect of SAHA treatment at 2 h and 24 h NaCl treatment. Salt stress-unresponsive genes and transient salt stress-responsive genes were enriched in class 3 (Figure 6C) and class 4 (Figure 6D), respectively. GO enrichment analysis indicated that GO:0051179 (localization), GO:0006810 (transport), GO:0016043 (cellular component organization), and GO:0048856 (anatomical structure development) are included in class 3, whereas GO:0051179 (localization) and GO:0006810 (transport) are included in class 4. Class 5 contains the most genes (1103 genes). Class 5 is a group of salt-responsive genes that are up-regulated at 2 h under both NaCl and SAHA treatment. Several gene ontology (GO) categories were enriched among these genes, such as GO:0006950 (response to stress), GO:0009414 (response to water deprivation), GO:0009651 (response to salt stress), GO:0009737 (response to abscisic acid stimulus), and GO:0006082 (organic acid metabolic process; Figure 6E).

### Genes related to salinity stress response in cassava

As GO ontology analysis revealed, class 5 contains high salinity stress-responsive genes involved in ABA biosynthesis and signal transduction, including homologs of *Arabidopsis* genes *NCED3* (*MeNCED3*: RknMes02_025528: cassava4.1_026283m, Tables 1, 2), *EDL3* (*MeEDL3*: RknMes02_033569: cassava4.1_030886m, Tables 1, 2), *ABI1* (*MeABI1*: RknMes02_033475: cassava4.1_020355m, Tables S1, S2), *ABI2* (*MeABI2*: RknMes02_010484: cassava4.1_010060m, Tables 1, 2), and other *PP2Cs*, such as *AHG1* (*MeAHG1*: RknMes02_003939: cassava4.1_013309m, Tables 1, 2), *AHG3* (*MeAHG3*: RknMes02_013552: cassava4.1_008067m, Tables S1, S2), *HAI1* (*MeHAI1*: RknMes02_002573: cassava4.1_007998m, Table 1), *HAI2* (*MeHAI2*: RknMes02_006068: cassava4.1_007913m, Tables 1, 2), *HAB1* (*MeHAB1*: RknMes02_048414: cassava4.1_005959m, Tables S1, S2) (Ma et al., 2006). In *Arabidopsis*, the salt-induced upregulation of several transcription factors including *ATAF1, ATHB12, NAP, AZF2, HSF2, RD26*, and *ATERF4*, has been reported (Ma et al., 2006; Matsui et al., 2008). In cassava, salt stress induced the expression of their homologs *MeATAF1* (RknMes02_016900: cassava4.1_013132m, Tables S1, S2), *MeATHB12* (RknMes02_016407: cassava4.1_015049m, Table 2), *MeNAP* (RknMes02_036224: cassava4.1_013467m, Tables S1, S2), and *MeRD26* (RknMes02_031261: cassava4.1_010999m, Tables 1, 2). LEA proteins, whose overexpression gives tolerance to salinity stress, are regulated by ABA in land plants and thought to function as molecular shields to prevent aggregation caused by dehydration (Shinde et al., 2012). *MeLEA* (RknMes02_006505: cassava4.1_025947m) showed the highest the highest up-regulated gene by salt stress (Table 1, Table S1). These results suggest that ABA synthesis and its signal transduction pathway coordinate the basis of the response to salinity stress in cassava. This is consistent with previous data in *Arabidopsis* (Matsui et al., 2008).

**Table 2:** **List of top 40 cassava genes upregulated (log_**2**_ ratio > 1; FDR < 0.0001) in roots by treatment with 200 mM NaCl for 24 h in non-SAHA-pretreated plants**.

**ProbeID**	**AGI code^a^**	**Encoded proteins/other features^b^**	**w/o SAHA^c^**	**FDR**
			**log_2_ ratio (24 h NaCl/0 h NaCl)**	
RknMes02_038832	AT3G24310	Myb domain protein 305	6.437	1.01E-05
RknMes02_006505	AT1G52690	Late embryogenesis abundant protein (LEA) family protein	5.890	3.36E-06
RknMes02_033569	AT3G63060	EID1-like 3	5.813	3.62E-05
RknMes02_057161	AT4G33467	Unknown protein	5.701	1.13E-05
RknMes02_049529	AT3G54420	Chitinase class IV	5.653	4.12E-05
RknMes02_010887	AT2G43590	Chitinase family protein	5.245	6.27E-05
RknMes02_008877	AT4G31240	Protein kinase C-like zinc finger protein	5.162	7.87E-06
RknMes02_027987	AT1G60420	DC1 domain-containing protein	5.143	6.85E-06
RknMes02_017023	AT4G16160	ATOEP16-2, ATOEP16-S	5.118	8.36E-07
RknMes02_003939	AT5G51760	ABA-hypersensitive germination 1	4.407	5.34E-05
RknMes02_052347	AT3G51810	Stress induced protein	4.200	7.15E-05
RknMes02_010484	AT5G57050	ABA insensitive 2	4.153	3.93E-05
RknMes02_039460	AT5G59220	Highly ABA-induced PP2C gene 1	3.978	4.47E-05
RknMes02_056041	AT3G30210	Myb domain protein 121	3.893	1.01E-06
RknMes02_018746		Protein phosphatase 2C	3.850	6.38E-05
RknMes02_055947	AT1G16770	Unknown protein	3.846	1.54E-06
RknMes02_025528	AT3G14440	Nine-cis-epoxycarotenoid dioxygenase 3	3.832	4.44E-05
RknMes02_047856	AT5G03850	Nucleic acid-binding, OB-fold-like protein	3.734	5.20E-05
RknMes02_051689	AT4G35680	Arabidopsis protein of unknown function (DUF241)	3.571	1.88E-05
RknMes02_006068	AT1G07430	Highly ABA-induced PP2C gene 2	3.526	7.43E-06
RknMes02_023528		Protein phosphatase 2C 3-related	3.493	2.74E-05
RknMes02_034838	AT2G29380	Highly ABA-induced PP2C gene 3	3.479	1.08E-05
RknMes02_016407	AT3G61890	Homeobox 12**/**homeobox 7	3.312	1.38E-05
RknMes02_039694	AT3G03341	Unknown protein	3.288	6.19E-06
RknMes02_013832		Homeobox-leucine zipper protein ATHB-12-related	3.211	1.30E-05
RknMes02_035128	AT1G44446	Arabidopsis thaliana Chrolophyll A oxygenase	3.193	8.07E-05
RknMes02_053159	AT4G16835	Tetratricopeptide repeat (TPR)-like superfamily protein	3.142	8.93E-06
RknMes02_054176	AT5G20230	Blue-copper-binding protein	3.117	1.93E-05
RknMes02_048392	AT2G38940	Phosphate transporter 1;4	2.986	5.87E-06
RknMes02_010463		Homeobox-leucine zipper protein ATHB-12-related	2.957	1.26E-05
RknMes02_031261	AT4G27410	Responsive to desiccation 26	2.952	1.01E-05
RknMes02_056689	AT3G59850	Pectin lyase-like superfamily protein	2.922	4.17E-06
RknMes02_058983	AT1G24530	Transducin/WD40 repeat-like superfamily protein	2.901	1.15E-05
RknMes02_054233	AT5G42200	RING/U-box superfamily protein	2.829	4.97E-06
RknMes02_006418	AT1G18100	PEBP (phosphatidylethanolamine-binding protein) family protein	2.819	4.15E-06
RknMes02_010142	AT5G66110	Heavy metal transport/detoxification superfamily protein	2.742	5.83E-06
RknMes02_054543	AT1G60190	ARM repeat superfamily protein	2.669	3.46E-05
RknMes02_046170	AT2G43870	Pectin lyase-like superfamily protein	2.653	1.88E-05
RknMes02_009426	AT5G14860	UDP-Glycosyltransferase superfamily protein	2.641	5.69E-05
RknMes02_025204	AT1G09960	Sucrose transporter 4	2.611	9.43E-05

a *AGI code is shown if proteins encoded in each cassava gene (probe ID) have high amino acid sequence similarity (E ≤ 10^−5^) to Arabidopsis homologs*.

b *Encoded proteins/other features indicate the putative functions of the gene products that are expected from sequence similarity. The information for the NCBI protein reference sequence with the highest sequence similarity to the probes is shown*.

c *Plants that were not pretreated with SAHA were used*.

In addition to ABA, the hormones JA and methyl jasmonate (MeJA) are also involved in salt stress response. Consistent with previous reports that MeJA induces salt responsive genes in roots (Ma et al., 2006), our transcriptome analysis from root sample detected the induced mRNA expression of genes for JA/MeJA biosynthesis (*MeACX2*: RknMes02_027394: cassava4.1_002855m, Table S1; *MeACX3*: RknMes02_012214: cassava4.1_002966m, Table S1; and *MeJMT*: RknMes02_028787, cassava4.1_010155m, Table 1), suggesting that the JA signaling pathway is also involved in response to salt stress in cassava.

The expression levels of osmoprotectant biosynthesis-related genes, such as Δ*-1-pyrroline-5-carboxylate synthetase* (*P5CS*) and *raffinose synthase* were induced under salinity stress condition. *P5CS1* (*MeP5CS1*: RknMes02_003952: cassava4.1_002374m) catalyzes the rate-limiting step in the biosynthesis of proline. *MeP5CS1* was up-regulated at 2 and 24 h NaCl treatment (Tables S1, S2). Soluble sugars of the raffinose family have been associated with plant response to abiotic stresses. *Raffinose synthase* (*RS*) is responsible for raffinose biosynthesis. It has been reported that high salinity stress increased *RS5* transcription (Egert et al., 2013; ElSayed et al., 2014). The expression of *MeRS5* (RknMes02_026274: cassava4.1_002019m) was upregulated at 2 h NaCl treatment (Table S1). Proline and raffinose are likely to be synthesized and function as osmoprotectants under salinity stress in cassava.

### SAHA pretreatment-upregulated genes identified by transcriptome analysis

We identified 421 genes whose expression was upregulated by 24 h SAHA treatment (Table S3). After pre-treatment of SAHA, 745 and 268 genes were upregulated by 2 and 24 h salt treatment, respectively (Tables S4, S5).

**Figure 7:**
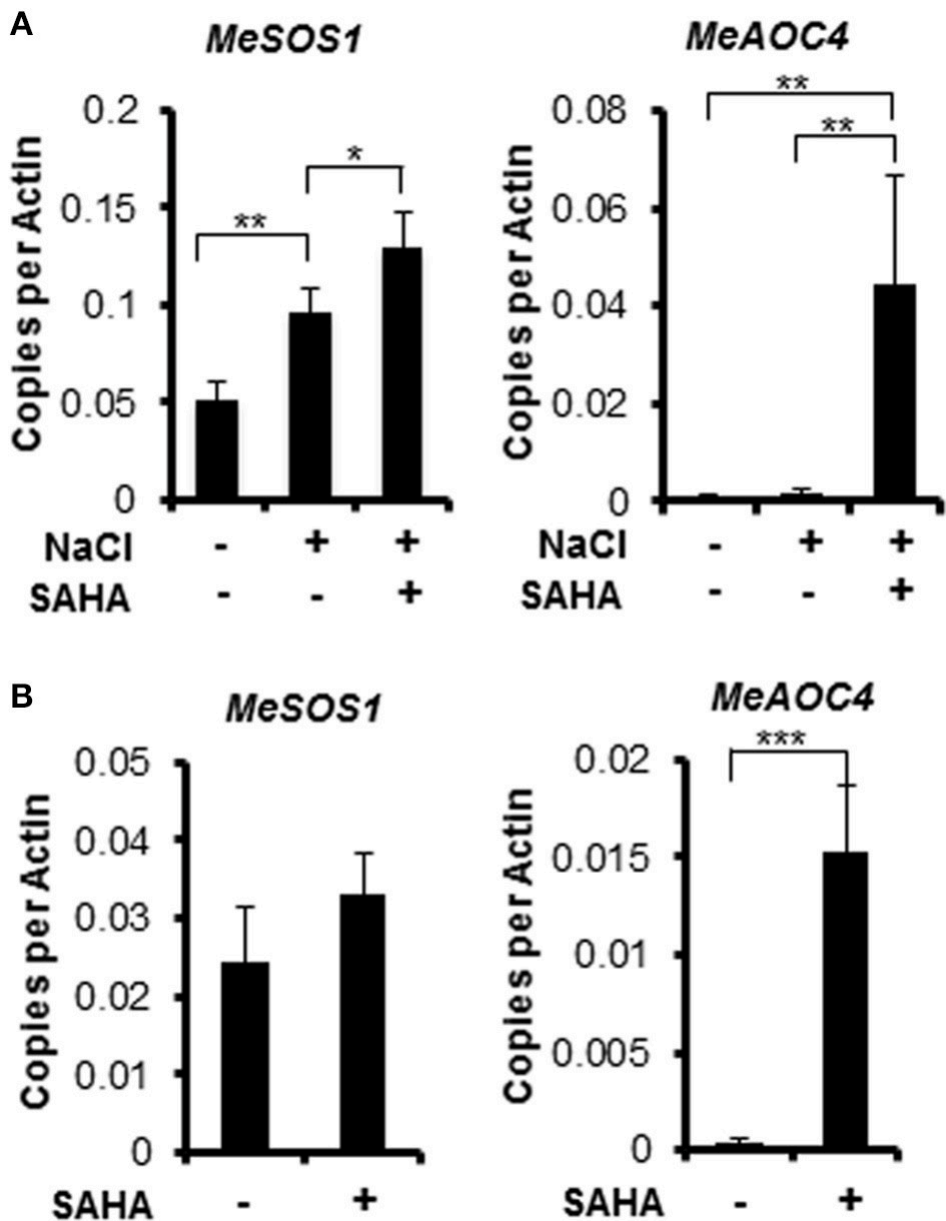
**Expression profiles of ***MeSOS1*** (***Salt Overly Sensitive 1***) and ***MeAOC4*** (***Allene Oxide Cyclase 4***) genes using quantitative real-time RT-PCR (qRT-PCR) analysis. (A)** Cassava plantlets were treated with 100 μM SAHA for 24 h then subjected to 200 mM salt medium for 2 h. **(B)** Cassava plantlets were treated with 100 μM SAHA for 24 h. Root samples were collected. Asterisks indicate significantly different means (^*^p < 0.05, ^**^p < 0.005, ^***^p < 0.001) as determined with a *t*-test. Actin was used as reference gene. Error bars represent the means ± SD. Three independent biological replicates were performed for each condition.

*Salt Overly Sensitive1* (*SOS1*) is a gene responsible for increased salinity tolerance in *Arabidopsis* plants treated with the HDAC inhibitor Ky-2. The HDAC inhibitor strongly induced the expression of *AtSOS1* to 5-, 3.5-, and 1.67-fold during NaCl treatment for 0, 2 and 10 h, respectively (Sako et al., 2016). Overexpression of *SOS1* enhances tolerance to salinity stress in *Arabidopsis* (Shi et al., 2003; Yang et al., 2009) and tobacco (Yue et al., 2012). SAHA treatment enhanced *MeSOS1* gene expression to 1.35-fold compared with untreated plants (Figure 7A). Although, the upregulation of *MeSOS1* gene by SAHA treatment was not so significant (Figure 7B), the slight enhanced expression of *MeSOS1* might contribute to the increased salinity stress tolerance by SAHA pretreatment in cassava.

Transcriptome analysis revealed that the mRNA expression of genes, involved in phytohormone [abscisic acid (ABA), jasmonic acid (JA), ethylene, and gibberellin] biosynthesis pathways, was up-regulated after NaCl treatment in SAHA-pretreated roots (Table S8). Among them, the expression of an *allene oxide cyclase* gene (*MeAOC4*: RknMes02_051874: cassava4.1_022180m) was strongly induced by SAHA treatment (Tables 3–5). SAHA treatment enhanced *MeAOC4* expression to 32.8-fold compared with untreated plants (Figure 7A). Previous studies have revealed that overexpression of *AOCs* can confer salinity stress tolerance to several crops (Yamada et al., 2002; Pi et al., 2009; Zhao et al., 2014). According to the current cassava genome database, *AOCs* constitute a small gene family (*MeAOC3-1*: RknMes02_049533: cassava4.1_014582m; *MeAOC3-2*: RknMes02_054684: cassava4.1_026961m; and *MeAOC4*: RknMes02_051874: cassava4.1_022180m) in cassava (Figure S1). Our transcriptome analysis revealed that *MeAOC3-2* is a high salinity stress-responsive gene and the expression of *MeAOC4* is not upregulated by high-salinity stress (Figure S1). SAHA treatment increased the expression of *MeAOC4* to 35-fold (Figure 7B), suggesting that activation of the JA pathway mediated by overexpression of *MeAOC4* might play a pivotal role in alleviating high-salinity stress in cassava.

**Table 3:** **List of top 40 cassava genes upregulated (log_**2**_ ratio > 1; FDR < 0.0001) in roots by SAHA treatment**.

**ProbeID**	**AGI code^a^**	**Encoded proteins/other features^b^**	**log_2_ ratio (SAHA 24 h/non-treated)**	**FDR**
RknMes02_051874	AT1G13280	Allene oxide cyclase 4	5.135	1.70E-07
RknMes02_058141	AT3G06720	Importin alpha isoform 1	4.941	1.01E-06
RknMes02_055141	AT2G40210	AGAMOUS-like 48	4.914	1.18E-06
RknMes02_048392	AT2G38940	Phosphate transporter 1;4	4.837	5.87E-06
RknMes02_058688	AT3G28880	Ankyrin repeat family protein	4.808	4.22E-07
RknMes02_055845	AT3G51880	High mobility group B2///high mobility group B1	4.757	1.37E-05
RknMes02_058115	AT3G07390	Auxin-induced in root cultures 12	4.728	2.84E-05
RknMes02_001781	AT3G58110	Unknown protein	4.715	1.34E-06
RknMes02_052316	AT4G01950	Glycerol-3-phosphate acyltransferase 3	4.700	8.32E-06
RknMes02_001711	AT4G32460	Protein of unknown function, DUF642	4.685	1.13E-06
RknMes02_057300	AT2G13810	AGD2-like defense response protein 1	4.524	1.13E-06
RknMes02_055925	AT4G34135	UDP-glucosyltransferase 73B2	4.435	1.80E-06
RknMes02_054888	AT1G01690	Putative recombination initiation defects 3	4.407	5.59E-06
RknMes02_050833	AT4G39250	RAD-like 1**/**RAD-like 6	4.361	1.48E-06
RknMes02_039148		Zein-binding	4.350	1.13E-06
RknMes02_013200	AT4G34131	UDP-glucosyl transferase 73B3	4.301	3.27E-06
RknMes02_055406	AT5G17350	Unknown protein	4.299	1.08E-07
RknMes02_057295	AT4G37770	1-amino-cyclopropane-1-carboxylate synthase 8	4.083	1.20E-05
RknMes02_001575	AT4G25640	Detoxifying efflux carrier 35	4.064	1.37E-05
RknMes02_057627	AT4G32480	Protein of unknown function (DUF506)	4.056	1.66E-05
RknMes02_058010	AT5G07610	F-box family protein	4.005	7.95E-06
RknMes02_011864		Glucosyl/Glucuronosyl transferase	3.971	3.36E-06
RknMes02_057486	AT2G21220	SAUR-like auxin-responsive protein family	3.878	2.98E-07
RknMes02_024608	AT3G04620	Alba DNA/RNA-binding protein	3.876	2.89E-05
RknMes02_054063	AT1G14440	Homeobox protein 33///homeobox protein 31	3.836	7.51E-05
RknMes02_051551	AT2G33480	NAC domain containing protein 41	3.799	3.89E-06
RknMes02_057805	AT3G24060	Plant self-incompatibility protein S1 family	3.776	1.47E-05
RknMes02_048226	AT3G16380	Poly(A) binding protein 6	3.775	1.01E-05
RknMes02_055397	AT2G26140	FTSH protease 4	3.745	7.16E-06
RknMes02_001499	AT5G54160	O-methyltransferase 1	3.697	8.90E-05
RknMes02_004432	AT5G11420	Protein of unknown function, DUF642	3.646	1.65E-06
RknMes02_053062	AT1G68390	Core-2/I-branching beta-1,6-N-acetylglucosaminyltransferase family protein	3.612	6.51E-07
RknMes02_050655	AT3G04710	Ankyrin repeat family protein	3.533	5.74E-06
RknMes02_022851	AT3G47800	Galactose mutarotase-like superfamily protein	3.424	1.18E-06
RknMes02_026790	AT2G26870	Non-specific phospholipase C2	3.386	6.01E-08
RknMes02_051740	AT1G54115	Cation calcium exchanger 4	3.375	1.66E-06
RknMes02_054668	AT4G36740	Homeobox protein 40///homeobox protein 21	3.366	1.46E-06
RknMes02_053782	AT4G17380	MUTS-like protein 4	3.333	1.34E-05
RknMes02_052163	AT2G22840	Growth-regulating factor 1	3.294	3.76E-05
RknMes02_054016	AT5G42120	Concanavalin A-like lectin protein kinase family protein	3.244	1.52E-05

a *AGI code is shown if proteins encoded in each cassava gene (probe ID) have high amino acid sequence similarity (E ≤ 10^−5^) to Arabidopsis homologs*.

b *Encoded proteins/other features indicate the putative functions of the gene products that are expected from sequence similarity. The information for the NCBI protein reference sequence with the highest sequence similarity to the probes is shown*.

**Table 4:** **List of top 40 cassava genes with higher expression (log_**2**_ ratio > 1; FDR < 0.0001) in roots of SAHA-pretreated plants compared with non-SAHA-pretreated plants in the presence of NaCl for 2 h**.

**ProbeID**	**AGI code^a^**	**Encoded proteins/other features^b^**	**log_2_ ratio {(NaCl 2 h after SAHA 24 h)/(NaCl 2 h after non-SAHA 24 h)}**	**FDR**
RknMes02_057300	AT2G13810	AGD2-like defense response protein 1	7.348	1.13E-06
RknMes02_058141	AT3G06720	Importin alpha isoform 1	6.563	1.01E-06
RknMes02_051874	AT1G13280	Allene oxide cyclase 4	5.966	1.70E-07
RknMes02_052316	AT4G01950	Glycerol-3-phosphate acyltransferase 3	5.717	8.32E-06
RknMes02_055141	AT2G40210	AGAMOUS-like 48	5.695	1.18E-06
RknMes02_058688	AT3G28880	Ankyrin repeat family protein	5.456	4.22E-07
RknMes02_050833	AT4G39250	RAD-like 1/RAD-like 6	5.386	1.48E-06
RknMes02_009639	AT1G34300	Lectin protein kinase family protein	5.369	1.57E-05
RknMes02_053062	AT1G68390	Core-2/I-branching beta-1,6-N-acetylglucosaminyltransferase family protein	5.273	6.51E-07
RknMes02_057007	AT5G57620	Myb domain protein 36	5.228	3.40E-05
RknMes02_058115	AT3G07390	Auxin-induced in root cultures 12	5.167	2.84E-05
RknMes02_058010	AT5G07610	F-box family protein	4.974	7.95E-06
RknMes02_053766	AT4G33870	Peroxidase superfamily protein	4.907	1.74E-05
RknMes02_001711	AT4G32460	Protein of unknown function, DUF642	4.892	1.13E-06
RknMes02_057698	AT2G45400	NAD(P)-binding Rossmann-fold superfamily protein	4.872	3.32E-06
RknMes02_051856	AT1G35910	Trehalose-6-phosphate phosphatase	4.833	4.21E-05
RknMes02_050655	AT3G04710	Ankyrin repeat family protein	4.790	5.74E-06
RknMes02_054888	AT1G01690	Putative recombination initiation defects 3	4.788	5.59E-06
RknMes02_055406	AT5G17350	Unknown protein	4.768	1.08E-07
RknMes02_001781	AT3G58110	Unknown protein	4.741	1.34E-06
RknMes02_054063	AT1G14440	Homeobox protein 33/homeobox protein 31	4.683	7.51E-05
RknMes02_052664	AT5G64310	Arabinogalactan protein 1	4.529	4.99E-05
RknMes02_037315	AT4G00330	Calmodulin-binding receptor-like cytoplasmic kinase 2	4.438	1.88E-06
RknMes02_051803	AT3G09270	Glutathione S-transferase TAU 8	4.369	1.29E-05
RknMes02_037654	AT4G29110	Unknown protein	4.337	7.18E-06
RknMes02_051464	AT3G06240	F-box family protein	4.284	2.95E-06
RknMes02_053782	AT4G17380	MUTS-like protein 4	4.231	1.34E-05
RknMes02_049288	AT5G52060	BCL-2-associated athanogene 1	4.218	2.22E-05
RknMes02_000416	AT2G26870	Non-specific phospholipase C2	4.138	1.20E-07
RknMes02_051638	AT4G22660	F-box family protein with a domain of unknown function (DUF295)	4.008	5.43E-05
RknMes02_051551	AT2G33480	NAC domain containing protein 52	3.998	3.89E-06
RknMes02_052689	AT5G16080	Carboxyesterase 17	3.989	3.58E-05
RknMes02_020256	AT3G19270	Cytochrome P450, family 707, subfamily A, polypeptide 4	3.985	3.39E-05
RknMes02_011799	AT2G28090	Heavy metal transport/detoxification superfamily protein	3.984	4.66E-05
RknMes02_055845	AT3G51880	High mobility group B2**/**high mobility group B1	3.974	1.37E-05
RknMes02_007372		Heavy metal transport/detoxication domain-containing protein-related	3.962	3.23E-05
RknMes02_051562	AT5G64360	Chaperone DnaJ-domain superfamily protein	3.929	5.97E-08
RknMes02_054294	AT1G67810	Sulfur E2	3.918	8.44E-07
RknMes02_056535	AT5G53980	Homeobox protein 52	3.912	3.92E-06
RknMes02_055397	AT2G26140	FTSH protease 4	3.786	7.16E-06

a *AGI code is shown if proteins encoded in each cassava gene (probe ID) have high amino acid sequence similarity (E ≤ 10^−5^) to Arabidopsis homologs*.

b *Encoded proteins/other features indicate the putative functions of the gene products that are expected from sequence similarity. The information for the NCBI protein reference sequence with the highest sequence similarity to the probes is shown*.

**Table 5:** **List of top 40 cassava genes with higher expression (log_**2**_ ratio > 1; FDR < 0.0001) in roots of SAHA-pretreated plants compared with non-SAHA-pretreated plants in the presence of NaCl for 24 h**.

**ProbeID**	**AGI code^a^**	**Encoded proteins/other features^b^**	**log_2_ ratio {(NaCl 24 h after SAHA 24 h)/(NaCl 24 h after non-SAHA 24 h)}**	**FDR**
RknMes02_057300	AT2G13810	AGD2-like defense response protein 1	7.049	1.13E-06
RknMes02_058115	AT3G07390	Auxin-responsive family protein	5.000	2.84E-05
RknMes02_051874	AT1G13280	Allene oxide cyclase 4	4.707	1.70E-07
RknMes02_057486	AT2G21220	SAUR-like auxin-responsive protein family	4.596	2.98E-07
RknMes02_052316	AT4G01950	Glycerol-3-phosphate acyltransferase 3	4.487	8.32E-06
RknMes02_051551	AT2G33480	NAC domain containing protein 52	4.457	3.89E-06
RknMes02_039148		Zein-binding	4.044	1.13E-06
RknMes02_058141	AT3G06720	Importin alpha isoform 1	4.044	1.01E-06
RknMes02_055141	AT2G40210	AGAMOUS-like 48	3.809	1.18E-06
RknMes02_055406	AT5G17350	Unknown protein	3.765	1.08E-07
RknMes02_057007	AT5G57620	Myb domain protein 36	3.757	3.40E-05
RknMes02_056640	AT3G11930	Adenine nucleotide alpha hydrolases-like superfamily protein	3.720	3.24E-06
RknMes02_028787	AT1G19640	Jasmonic acid carboxyl methyltransferase	3.695	3.94E-06
RknMes02_057539	AT5G07610	F-box family protein	3.609	2.44E-07
RknMes02_054063	AT1G14440	Homeobox protein 33**/**homeobox protein 31	3.598	7.51E-05
RknMes02_016408	AT4G36470	S-adenosyl-L-methionine-dependent methyltransferases superfamily protein	3.550	9.03E-06
RknMes02_052163	AT2G22840	Growth-regulating factor 1	3.540	3.76E-05
RknMes02_053062	AT1G68390	Core-2/I-branching beta-1,6-N-acetylglucosaminyltransferase family protein	3.491	6.51E-07
RknMes02_053782	AT4G17380	MUTS-like protein 4	3.320	1.34E-05
RknMes02_055845	AT3G51880	High mobility group B2/high mobility group B1	3.292	1.37E-05
RknMes02_050833	AT4G39250	RAD-like 1/RAD-like 6	3.292	1.48E-06
RknMes02_001711	AT4G32460	Protein of unknown function, DUF642	3.224	1.13E-06
RknMes02_058688	AT3G28880	Ankyrin repeat family protein	3.222	4.22E-07
RknMes02_001781	AT3G58110	Unknown protein	3.199	1.34E-06
RknMes02_044414			3.137	2.80E-05
RknMes02_054888	AT1G01690	Putative recombination initiation defects 3	3.128	5.59E-06
RknMes02_037654	AT4G29110	Unknown protein	2.989	7.18E-06
RknMes02_055397	AT2G26140	FTSH protease 4	2.940	7.16E-06
RknMes02_052664	AT5G64310	Arabinogalactan protein 1	2.918	4.99E-05
RknMes02_001499	AT5G54160	O-methyltransferase 1	2.918	8.90E-05
RknMes02_012269	AT1G06460	Alpha-crystallin domain 32.1	2.841	7.23E-05
RknMes02_051562	AT5G64360	Chaperone DnaJ-domain superfamily protein	2.832	5.97E-08
RknMes02_014532	AT1G17020	Senescence-related gene 1	2.798	3.67E-05
RknMes02_051803	AT3G09270	Glutathione S-transferase TAU 8/glutathione S-transferase tau 7	2.770	1.29E-05
RknMes02_006016			2.766	9.03E-06
RknMes02_051840	AT3G09280	Unknown protein	2.697	2.61E-05
RknMes02_013200	AT4G34131	UDP-glucosyl transferase 73B3	2.665	3.27E-06
RknMes02_019058	AT1G80050	Adenine phosphoribosyl transferase 2	2.655	3.44E-06
RknMes02_012739	AT2G18550	Homeobox protein 21	2.639	2.53E-06

a *AGI code is shown if proteins encoded in each cassava gene (probe ID) have high amino acid sequence similarity (E ≤ 10^−5^) to Arabidopsis homologs*.

b *Encoded proteins/other features indicate the putative functions of the gene products that are expected from sequence similarity. The information for the NCBI protein reference sequence with the highest sequence similarity to the probes is shown*.

In the case of the HDAC inhibitor Ky-2, 72.9% of the HDAC inhibitor-inducible genes are salt-responsive genes in *Arabidopsis* (Sako et al., 2016). In contrast, 28.3% of SAHA-upregulated genes are high salinity stress-responsive (Figure S2A), and only 27 SAHA-upregulated genes were upregulated by high-salinity stress (2 and/or 24 h NaCl treatment; Table 6). In mangrove plants, lignin accumulation functions in blocking metal-ion influx with higher suberization, suggesting that lignification can prohibit ions from flowing inside (Cheng et al., 2014). We analyzed the following three genes that are believed to play critical roles in regulating lignin accumulation: *L-phenylalanine ammonia lyase* (*PAL*), *cinnamyl alcohol dehydrogenase* (*CAD*), and *caffeic acid 3-Omethyltransferase* (*COMT*) genes (Whetten and Sederoff, 1995; Ma and Xu, 2008; Nguyen et al., 2016). Among them, the expression of only *COMT* was significantly enhanced by SAHA treatment (Figure S3), suggesting that the lignin accumulation might be changed by overexpression of *COMT* induced by SAHA treatment and block ion uptake.

**Table 6:** **List of 27 SAHA-upregulated and high-salinity-stress-upregulated genes (log_**2**_ ratio > 1; FDR < 0.0001) in roots**.

**Probe ID**	**AGI Code^a^**	**Encoded proteins/other features^b^**	**Log**_**2**_ **ratio**		
			**SAHA 24 h/non-treated**	**w/o SAHA**^c^	
				**2 h NaCl/0 h NaCl**	**24 h NaCl/0 h NaCl**
RknMes02_048392	AT2G38940	Phosphate transporter 1;4	4.837	2.503	2.986
RknMes02_048720	AT1G03790	Tandam CCCH zinc finger protein4	2.912	2.844	1.541
RknMes02_022193	AT1G67810	Sulfur E2	2.217	1.650	1.967
RknMes02_049538	AT1G74650	Myb domain protein 31	1.903	2.312	2.343
RknMes02_049529	AT3G54420	Chitinase class IV	1.733	3.678	5.653
RknMes02_009426	AT5G14860	UDP-Glycosyltransferase superfamily protein	1.668	2.277	2.641
RknMes02_004082	AT1G17840	ATP-binding cassette G11	1.617	1.030	1.541
RknMes02_057376			1.616	2.369	1.867
RknMes02_029383	AT2G41190	Transmembrane amino acid transporter family protein	1.580	2.052	2.285
RknMes02_023158	AT5G02230	Haloacid dehalogenase-like hydrolase (HAD) superfamily protein	1.578	1.794	1.288
RknMes02_005365	AT2G37980	O-fucosyltransferase family protein	1.551	2.893	1.568
RknMes02_034114	AT5G26667	PYR6	1.529	1.525	1.991
RknMes02_049514	AT5G02230	Haloacid dehalogenase-like hydrolase (HAD) superfamily protein	1.408	1.462	1.008
RknMes02_036843	AT2G30860	Glutathione S-transferase PHI 9	1.403	1.735	1.360
RknMes02_019499			1.382	1.791	1.452
RknMes02_054233	AT5G42200	RING/U-box superfamily protein	1.378	3.304	2.829
RknMes02_028518	AT5G05690	Cytochrome P450 90A1	1.292	2.265	1.357
RknMes02_056529	AT2G37980	O-fucosyltransferase family protein	1.288	2.722	1.424
RknMes02_049315	AT1G13990	Unknown protein	1.274	2.322	1.425
RknMes02_008877	AT4G31240	Nucleoredoxin2	1.266	5.793	5.162
RknMes02_015088	AT1G13990	Unknown protein	1.212	2.360	1.508
RknMes02_040118	AT3G07130	Purple acid phosphatase 15	1.195	2.667	2.143
RknMes02_036162	AT1G21790	TRAM, LAG1 and CLN8 (TLC) lipid-sensing domain containing protein	1.179	2.241	1.159
RknMes02_024400		Arginine decarboxylase/L-arginine carboxy-lyase	1.143	2.592	2.085
RknMes02_027987	AT1G60420	Nucleoredoxin1	1.097	5.659	5.143
RknMes02_010471	AT3G54100	O-fucosyltransferase family protein	1.086	2.717	1.854
RknMes02_036257	AT5G23240	DNAJ protein C76	1.010	1.510	1.065

a *AGI code is shown if proteins encoded in each cassava gene (probe ID) have high amino acid sequence similarity (E ≤ 10^−5^) to Arabidopsis homologs*.

b *Encoded proteins/other features indicate the putative functions of the gene products that are expected from sequence similarity. The information for the NCBI protein reference sequence with the highest sequence similarity to the probes is shown*.

c *Plants that were not pretreated with SAHA were used*.

SAHA treatment decreased the expression of 141 high salinity stress-responsive genes including cell wall-related genes such as *beta-galactosidases* (RknMes02_003559, cassava4.1_001503m; RknMes02_030412, cassava4.1_001733m; RknMes02_034218, cassava4.1_001885m), *beta-glucosidase* (RknMes02_058028, cassava4.1_032518m), *pectin lyase* (RknMes02_051310, cassava4.1_021247m), *pectinesterase* (RknMes02_057990, cassava4.1_032455m), and *pectin methylesterase inhibitor 4* (RknMes02_055023, cassava4.1_027551m) (Figure S2, Table S9). The decreased expression of these genes might contribute to changes in cell-wall components caused by SAHA treatment (see Discussion).

## Discussion

Here we demonstrated that the HDAC inhibitor, SAHA, can decrease sodium ion content particularly in the stems, resulting in increased survival rates under high salinity stress conditions in cassava. Transcriptome analysis identified candidate genes, such as an allene oxide cyclase catalyzing an essential step in the biosynthesis of JA, whose expression is strongly upregulated by SAHA. This study shows evidence that the HDAC inhibitor is an effective small molecule for alleviating salinity stress in crops, and will improve understanding of the mechanisms by which histone acetylation regulates response to abiotic stress in cassava. In this study, we identified many salinity stress-upregulated genes in cassava. This study is the first report on the transcriptome analysis using microarray under high-salinity stress in cassava and the information will be useful for the development of high-salinity stress tolerant cassava plants.

This study showed that SAHA treatment reduced Na^+^ concentration in both stems and leaves. Plants are able to survive high salinity stress conditions by the maintenance of K^+^ and Na^+^ homeostasis using several transporters (Zhu, 2003; Ji et al., 2013; Julkowska and Testerink, 2015). Several transporters function in the alleviation of high-salinity stress. *SOS1* encodes the plasma-membrane Na^+^/H^+^ antiporter. Its activity can be detected only during salt stress conditions and is mediated by the Ca^2+^-responsive SOS3-SOS2 protein kinase complex (Qiu et al., 2002). When plants are exposed to high salinity stress conditions, *SOS1* functions in the control of Na^+^ efflux to maintain ion homeostasis. When the expression of *SOS1* is constitutively driven in *Arabidopsis*, the overexpressors become considerably tolerant to salt stress (Shi et al., 2003). In the woody plant *Populus*, introduction of the constitutively active *SOS2* isoform with induced expression of *SOS1* can enhance tolerance to salinity stress (Zhou et al., 2014). Plants also prevent excessive Na^+^ in cells by Na^+^ compartmentation by *Na*^+/^*H*^+^ *exchanger 1* (*NHX1*). *NHX1* functions to compartmentalize Na^+^ in the vacuole, resulting in low Na^+^ concentration and thus adjusting osmotic pressure to maintain water uptake. In *Arabidopsis*, co-expression of *SOS1* and *NHX1* enhances tolerance to NaCl concentrations up to 250 mM, suggesting the crucial functions of these genes. Furthermore, high-affinity K^+^ transporters (HKT) play an important role in Na^+^ exclusion from leaves in monocots and dicots. The overexpression of *AtHKT1;1* improves salt tolerance in *Arabidopsis* (Horie et al., 2009; Mølle et al., 2009). Our transcriptome analysis did not find significant alteration of their mRNA expression (data not shown).

Screening of HDAC inhibitors that may induce higher expression of genes for ion transporters (e.g., *NHX1* and *HKT*) is of interest as it may help to improve salt tolerance in cassava. HDACs in plants can be separated into three distinct families. The largest family is type I (RPD3-like), which consists of Zn^2+^-dependent deacetylases and is classified into three classes (I, II, and IV; Yang and Seto, 2008; Seto and Yoshida, 2014). They are generally conserved in eukaryotes, and there are 12 and 6 putative members in *Arabidopsis* (Hollender and Liu, 2008) and cassava (Figure S4), respectively. The second group of HDACs is plant-specific, and consists of HD-tuins. Four HD-tuins (HDT1-4) and 3 putative HD-tuins have been identified in *Arabidopsis* and cassava, respectively (Figure S4). The last group consists of homologs to the yeast Sir2 protein, which is a NAD^+^-dependent deacetylase. Two sirtuin proteins, SRT1 and SRT2 have been found in *Arabidopsis* (Hollender and Liu, 2008). Two putative sirtuins have been identified in cassava (Figure S4). HDAC inhibitors including Trichostatin A, SAHA, and sodium butyrate, whose target is Zn^2+^-dependent deacetylase, do not inhibit sirtuin activity (Richon, 2006). SAHA and related hydroxamic acid-based HDAC inhibitors have inhibitory effects on class I (HDAC1, 2, 3 and 8), class II (HDAC4, 5, 6, 7, 9 and 10), and class IV (HDAC11) human HDACs (Bolden et al., 2006). SAHA is a non-class selective inhibitor. Different selective inhibitors might be valuable for increasing tolerance to salt stress in cassava, because different gene sets are activated based on class or type selectivity of HDAC inhibitors and transcript abundance is also changed depending on inhibitory efficiency of each HDAC.

SAHA treatment strongly induced the mRNA expression of *MeAOC4*. *AOC* regulates a crucial step in JA biosynthesis, and the JA derivative, MeJA, alleviates salt stress in soybean (Yoon et al., 2009). The constitutive expression of *AOCs* can confer salinity tolerance to plants such as tobacco cell lines (Yamada et al., 2002) and wheat (Zhao et al., 2014), and references for the involvement of JA in environmental stresses can be found in Riemann et al. (2015). In light of these findings, it is highly possible that overexpression of *MeAOC4* contributes to increased tolerance to salt stress under SAHA treatment in cassava. AOC enzyme activity is altered by heteromerization with its own isomers, and the specific heteromerized pair *AtAOC2* and *AtAOC4* shows the highest activity of the four AOC proteins in *Arabidopsis* (Otto et al., 2016). As tissue-specific expression of *AOC* genes is observed in *Arabidopsis* (Stenzel et al., 2012), each *AOC* seems to be regulated in a tissue- or environmental-stress specific manner in cassava. Our transcriptomic data reveal that *MeAOC4* is not salt-responsive under our growth conditions and might be specifically expressed in a tissue such as flower or tuber that we have not analyzed. SAHA treatment induced the expression of *MeAOC4*, which might allow heteromerization of artificially induced *MeAOC4* with salt-induced *MeAOC3-2*, and play a pivotal role in increased tolerance under SAHA treatment.

## Author contributions

OP, MU, and MS designed the experiments; OP, MU, MI, YK, YU, AM, MT, CU, and HS conducted the experiments; OP, MU, YU, and AM analyzed the data; OP, MU, MY, JN, and MS wrote the manuscript.

### Conflict of interest statement

The authors declare that the research was conducted in the absence of any commercial or financial relationships that could be construed as a potential conflict of interest.
