# Supplementary material for: The Histone Deacetylase Inhibitor Suberoylanilide Hydroxamic Acid Alleviates Salinity Stress in Cassava
Source: Front Plant Sci. 2017 Jan 9;7:2039. doi: 10.3389/fpls.2016.02039 (PMC5220070; doi:10.3389/fpls.2016.02039)
Supplement: Supplementary file 1 [file Presentation1.PDF]

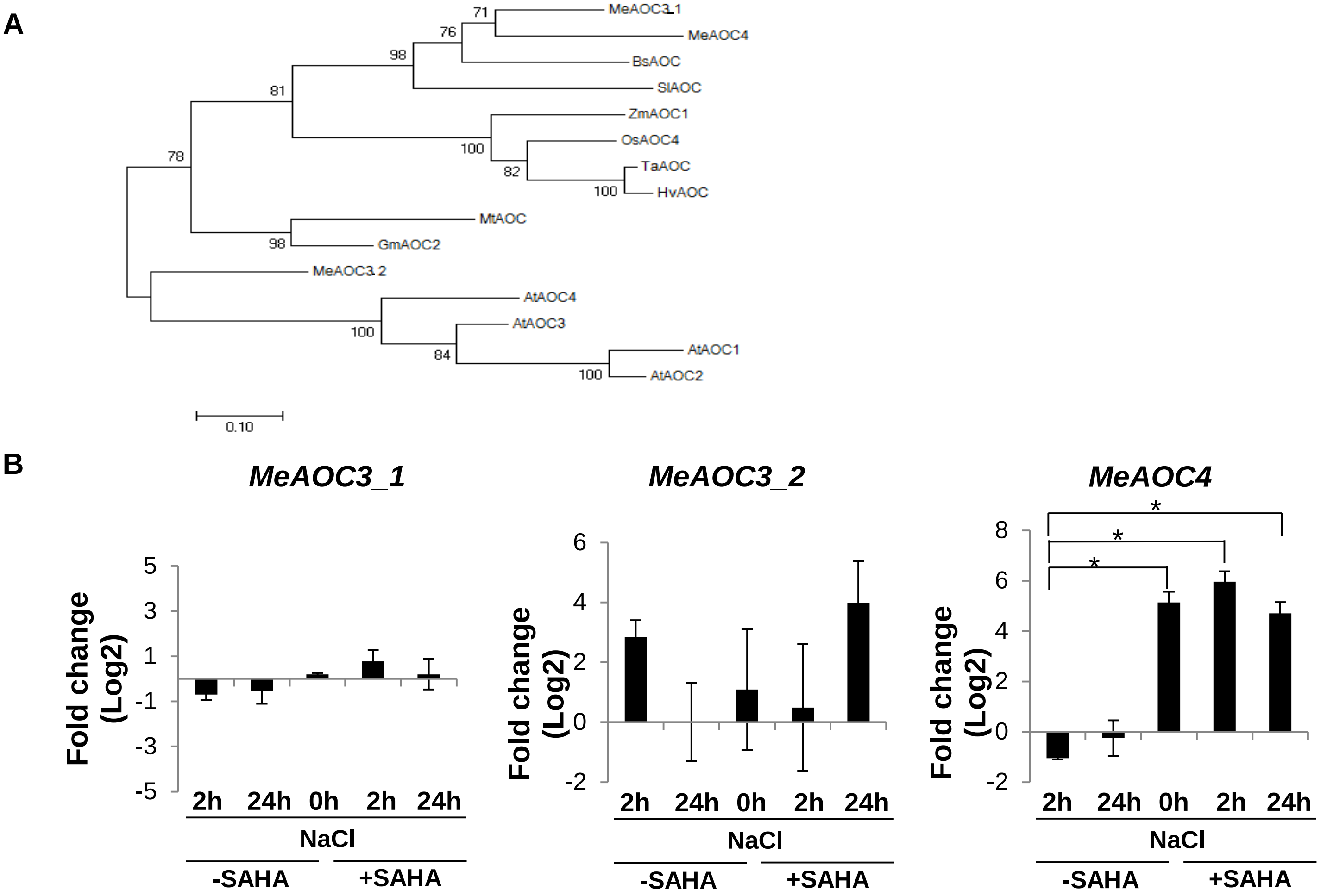

FIGURE S1. Molecular phylogenetic analysis of deduced amino acid sequences of AOC and the mRNA expression analysis of each *MeAOC* isoform from transcriptome data. Phylogenetic analysis was performed using maximum likelihood. AOC protein amino acid sequences from: *Manihot esculenta*, Me; *Bruguiera sexangula*, Bs; *Solanum lycopersicum*, Sl; *Zea mays*, Zm; *Oryza sativa*, Os; *Triticum aestivum*, Ta; *Hordeum vulgare*, Hv; *Medicago truncatula*, Mt; *Glycine max*, Gm; and *Arabidopsis thaliana*, At, were used for phylogenetic tree construction. *MeAOC4* is SAHA-responsive while *MeAOC3-2* is salt-responsive. (B) Expression of *MeAOC3-1*, *MeAOC3-2* and *MeAOC4* genes from microarray data. The expression profiles of cassava genes were obtained from cassava plantlets treated with 100 µM SAHA for 24 h then subjected to 200mM NaCl for 2 or 24 h. The normalized $\log_2$ values were used to plot the expression of genes. Asterisks indicate significantly different means (*$p < 0.001$) as determined with a t-test. Error bars represent the means ± SD. Transcript data was generated from three replicates.

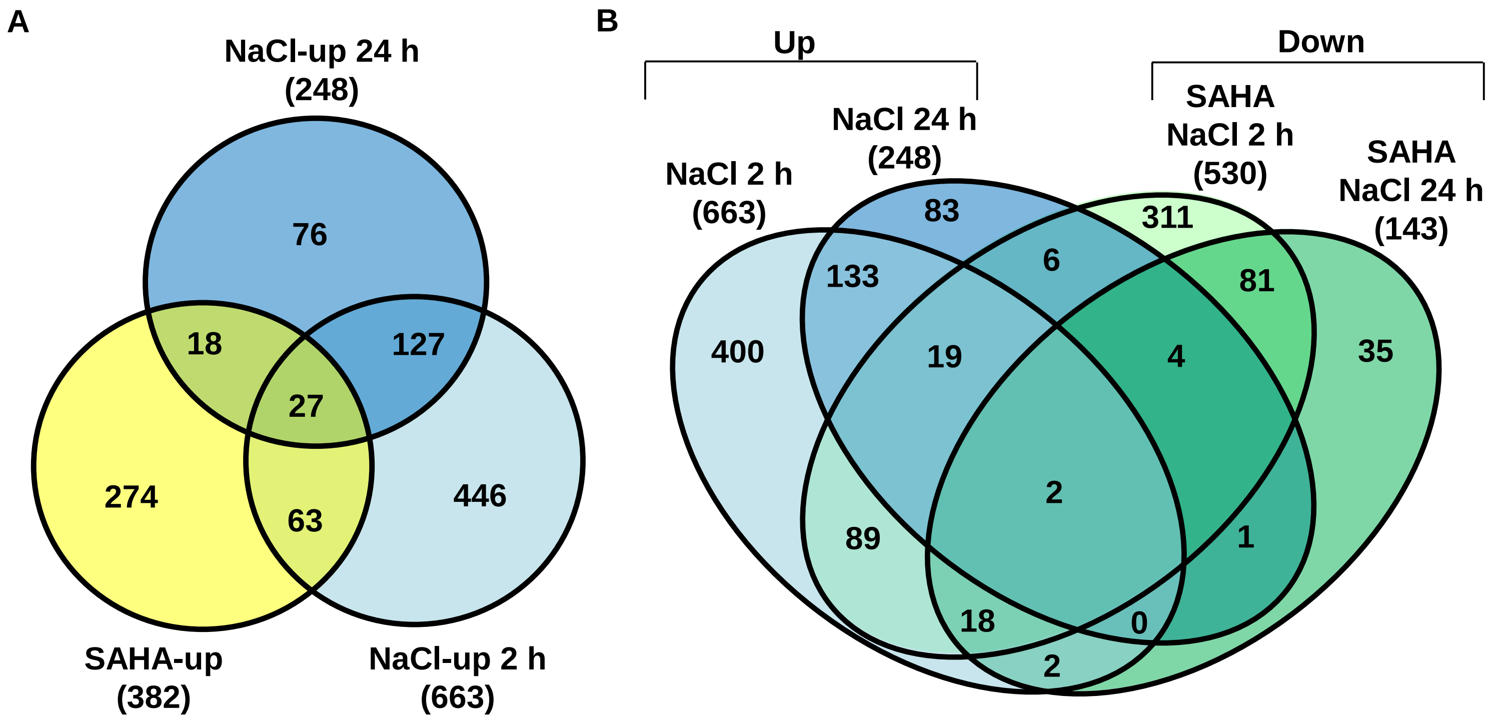

FIGURE S2. Genes that were significantly up-regulated in each condition. (A) Venn diagram representing SAHA-upregulated genes under normal conditions (light yellow) and salt-responsive genes at 2 and 24 h NaCl treatment (light blue and dark blue, respectively). (B) Venn diagram representing salt-upregulated genes at 2 and 24 h NaCl treatment (light blue and dark blue, respectively) and SAHA-downregulated genes under salt stress conditions at 2 and 24 h NaCl treatment (light green and dark green, respectively). The numbers in circles indicate the total number of significant genes under each condition.

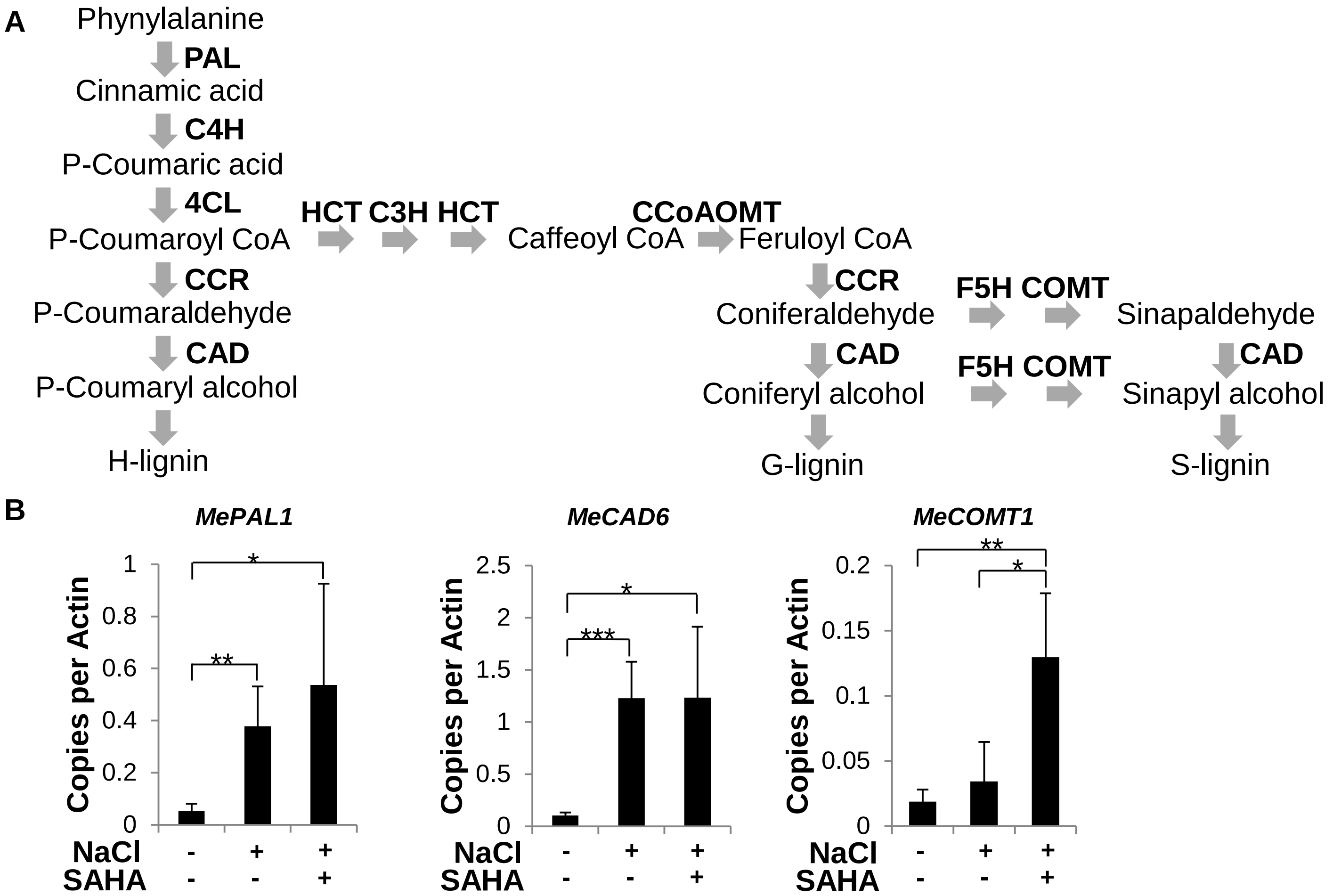

FIGURE S3. Lignin biosynthesis pathway. Representation of genes related to lignin biosynthesis (A). Abbreviations; PAL, *L-phenylalanine ammonia lyase*; C4H, *cinnamate 4-hydroxylase*; 4CL, *4-coumarate:CoA ligase*; CCR, *cinnamoyl-CoA reductase*; CAD, *cinnamyl alcohol dehydrogenase*; HCT, *hydroxycinnamoyl-CoA transferase*; C3H, *p-coumarate 3-hydroxylase*; CCoAOMT, *caffeoyl-CoA 3-O-methyl-transferase*; F5H, *ferulate 5-hydroxylase*; COMT, *caffeic acid O-methyltransferase*. (B) Expression profiles of *MePAL1* (RknMes02_004093: cassava4.1_003117m), *MeCAD6* (RknMes02_032531: cassava4.1_010429m) and *MeCOMT1* (RknMes02_001499: cassava4.1_013376) genes using quantitative real-time RT-PCR (qRT-PCR) analysis. Cassava plantlets were treated with 100 µM SAHA for 24 h then subjected with 200 mM salt medium for 2 h. Root samples were collected. Asterisks indicate significantly different means (*$p < 0.05$, **$p < 0.01$, ***$p < 0.005$) as determined with a t-test. Actin was used as reference gene. Error bars represent the means ± SD. Three independent biological replicates were performed for each condition.

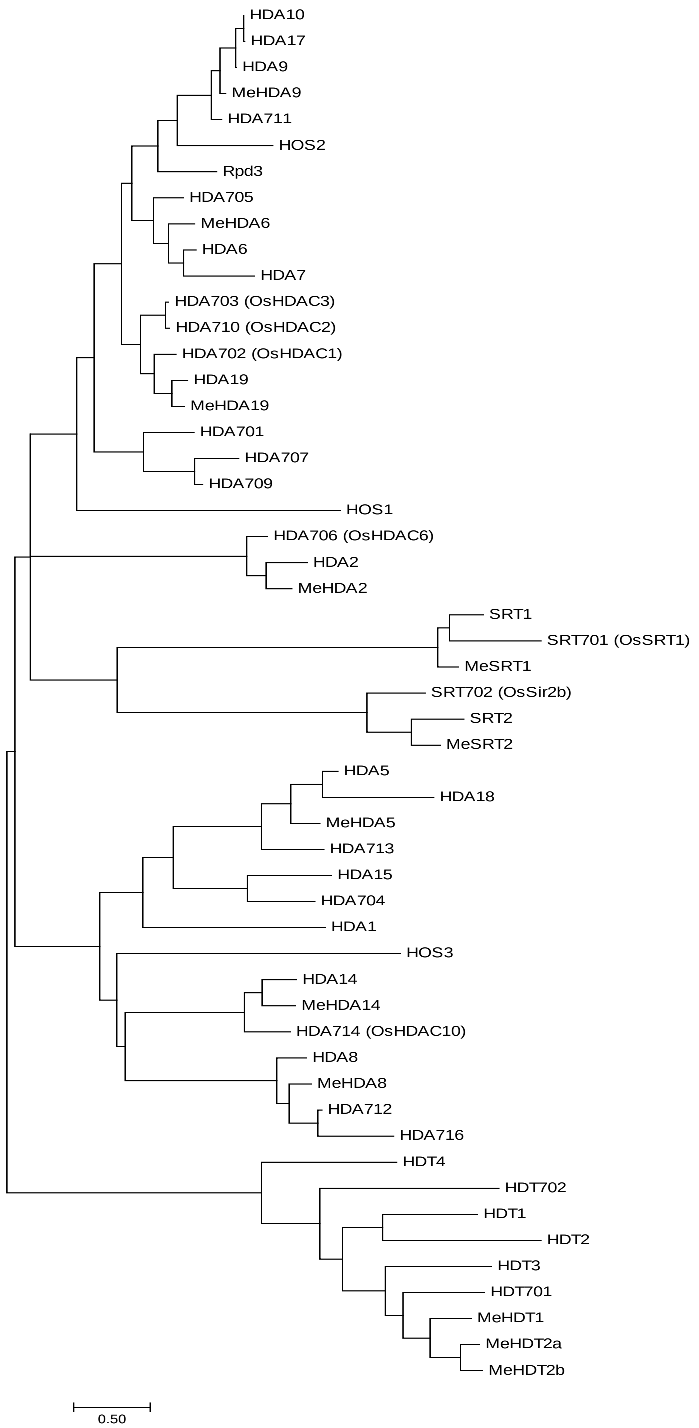

Figure S4. Molecular phylogenetic analysis of deduced amino acid sequences of HDAC. Phylogenetic analysis was performed using maximum likelihood. The tree was evaluated with 1,000 bootstrap replicates. The analysis was carried out using HDAC protein sequence from *Arabidopsis*, rice, yeast and cassava. *Arabidopsis* HDACs; SRT1: NP_200387, SRT2: NP_001078550, HDT1: NP_566872, HDT2: NP_851056, HDT3: NP_195994, HDT4: NP_565661, HDA2: NP_568480, HDA5: NP_001190583, HDA6: NP_201116, HDA7: NP_198410, HDA8: NP_563817, HDA9: NP_190054, HDA10: NP_190052, HDA14: NP_567921, HDA15: NP_850609, HDA17: NP_190035, HDA18: NP_200915, HDA19: NP_195526. Rice HDACs; HDA701: NM_192536, HDA702: AK068051, HDA703: AK120027, HDA704: AK111557, HDA705: AK111861, HDA706: AK100812, HDA707: NM_188519, HDA709: AK064282, HDA710: AK103097, HDA711: AK066667, HDA712: AK102786, HDA713: AK111892, HDA714: AK072557, HDA716: AK068179, SRT701: AK060337, SRT702: AK067069, HDT701: AK072845, HDT702 XM_463594. Yeast HDACs; HDA1: Z71297, RPD3: P32561, HOS1: Z4921923, HOS2: X91837, HOS3: U43503.

TABLE S1. Genes up-regulated in cassava roots in response to addition of 200 mM NaCl for 2 h

Probe ID	AGI code[1)]	E-value[2)]	Cassava ID	Encoded proteins/other features[3)]	w/o SAHA[4)] log_2 ratio (2 h NaCl /0 h NaCl)	p-value	BH FDR
RknMes02_006505	AT1G52690.2	3.00E-18	cassava4.1_025947m	Late embryogenesis abundant protein (LEA) family protein	10.250	2.25E-08	3.36E-06
RknMes02_053823	AT2G42850.1	0	cassava4.1_025512m	cytochrome P450, family 718	8.743	9.15E-11	2.44E-07
RknMes02_033569	AT3G63060.1	0	cassava4.1_030886m	EID1-like 3	8.738	1.61E-06	3.62E-05
RknMes02_025528	AT3G14440.1	0	cassava4.1_026283m	nine-cis-epoxycarotenoid dioxygenase 3	8.526	2.19E-06	4.44E-05
RknMes02_038832	AT3G24310.1	0	cassava4.1_014512m	myb domain protein 305	8.298	1.85E-07	1.01E-05
RknMes02_057161	AT4G33467.2	3.00E-09	cassava4.1_031129m	AT4G33467.2	8.062	2.29E-07	1.13E-05
RknMes02_004737	AT3G14440.1	0	cassava4.1_026283m	nine-cis-epoxycarotenoid dioxygenase 3	8.053	2.84E-06	5.21E-05
RknMes02_035302	AT3G24310.1	0	cassava4.1_013947m	myb domain protein 305	7.016	1.20E-06	3.00E-05
RknMes02_052347	AT3G51810.1	1.00E-38	cassava4.1_022993m	Stress induced protein	6.657	4.54E-06	7.15E-05
RknMes02_056041	AT3G30210.1	0	cassava4.1_029259m	myb domain protein 121	6.620	1.78E-09	1.01E-06
RknMes02_051689	AT4G35680.1	7.00E-39	cassava4.1_021861m	Arabidopsis protein of unknown function (DUF241)	6.399	5.83E-07	1.88E-05
RknMes02_039694	AT3G03341.1	2.00E-26	cassava4.1_020659m	unknown protein; Has 30201 Blast hits to 17322 proteins in 7	6.309	7.35E-08	6.19E-06
RknMes02_034350	AT3G14440.1	0	cassava4.1_034379m	nine-cis-epoxycarotenoid dioxygenase 3	6.104	1.19E-07	7.97E-06
RknMes02_023155	AT3G14440.1	0	cassava4.1_034379m	nine-cis-epoxycarotenoid dioxygenase 3	5.895	7.76E-07	2.29E-05
RknMes02_008877	AT4G31240.2	6.00E-06	cassava4.1_012358m	protein kinase C-like zinc finger protein	5.793	1.14E-07	7.87E-06
RknMes02_003939	AT5G51760.1	5.00E-25	cassava4.1_013309m	Protein phosphatase 2C family protein///Unknown	5.679	2.96E-06	5.34E-05
RknMes02_027987	AT1G60420.1	0	cassava4.1_008293m	DC1 domain-containing protein	5.659	8.74E-08	6.85E-06
RknMes02_056865	AT3G61060.1	0	cassava4.1_030662m	phloem protein 2-A13	5.645	1.18E-06	2.96E-05
RknMes02_006481	AT5G51760.1	6.00E-18	cassava4.1_013309m	Protein phosphatase 2C family protein///Unknown	5.580	5.48E-06	8.12E-05
RknMes02_055843	AT5G59190.1	0	cassava4.1_028946m	Subtilisin-like serine endopeptidase family protein///subtilase	5.563	1.04E-06	2.73E-05
RknMes02_054690	AT4G11360.1	3.00E-14	cassava4.1_026970m	RING/U-box superfamily protein	5.531	4.25E-08	4.68E-06
RknMes02_026274	AT5G40390.1	0	cassava4.1_002019m	Raffinose synthase family protein	5.531	2.13E-08	3.30E-06
RknMes02_055373	AT3G55646.1	1.00E-25	cassava4.1_028135m	AT3G55646.1///AT2G39855.2	5.518	2.07E-07	1.07E-05
RknMes02_009395	AT2G40170.1	3.00E-14	cassava4.1_022993m	Stress induced protein	5.427	7.15E-06	9.73E-05
RknMes02_056706	AT1G11530.1	2.00E-37	cassava4.1_030398m	C-terminal cysteine residue is changed to a serine 1	5.401	2.12E-07	1.08E-05
RknMes02_028787	AT1G19640.1	2.00E-12	cassava4.1_010155m	S-adenosyl-L-methionine-dependent methyltransferases super	5.366	3.04E-08	3.94E-06
RknMes02_017023	AT4G16160.2	2.00E-44	cassava4.1_017521m	Mitochondrial import inner membrane translocase subunit Ti	5.332	1.12E-09	8.36E-07
RknMes02_019566	AT4G27450.1	1.00E-39	cassava4.1_014315m	Aluminium induced protein with YGL and LRDR motifs	5.239	1.35E-06	3.25E-05
RknMes02_050300	AT3G55646.1	6.00E-26	cassava4.1_018345m	AT3G55646.1///AT2G39855.2	5.109	1.20E-09	8.41E-07
RknMes02_010142	AT5G66110.1	4.00E-14	cassava4.1_018348m	Heavy metal transport/detoxification superfamily protein	5.097	6.49E-08	5.83E-06
RknMes02_002573	AT5G59220.1	3.00E-15	cassava4.1_007998m	Protein phosphatase 2C family protein///highly ABA-induced	5.084	2.37E-06	4.68E-05
RknMes02_055268	AT4G25410.1	7.00E-33	cassava4.1_027962m	basic helix-loop-helix (bHLH) DNA-binding superfamily prot	5.070	2.34E-07	1.15E-05
RknMes02_031261	AT4G27410.2	0	cassava4.1_010999m	NAC (No Apical Meristem) domain transcriptional regulator	5.040	1.87E-07	1.01E-05
RknMes02_010484	AT5G57050.1	3.00E-11	cassava4.1_010060m	Protein phosphatase 2C family protein	5.009	1.82E-06	3.93E-05
RknMes02_006418	AT1G18100.1	1.00E-26	cassava4.1_021034m	PEBP (phosphatidylethanolamine-binding protein) family pro	4.998	3.32E-08	4.15E-06
RknMes02_013801			cassava4.1_007311m		4.960	2.33E-06	4.62E-05
RknMes02_034838	AT2G29380.1	4.00E-37	cassava4.1_007913m	highly ABA-induced PP2C gene 3	4.949	2.12E-07	1.08E-05
RknMes02_006068	AT1G07430.1	2.00E-20	cassava4.1_007913m	highly ABA-induced PP2C gene 2	4.937	1.03E-07	7.43E-06
RknMes02_011555	AT1G60420.1	1.00E-06	cassava4.1_008371m	DC1 domain-containing protein	4.879	9.67E-07	2.61E-05
RknMes02_038687	AT5G42290.1	4.00E-17	cassava4.1_020242m	transcription activator-related	4.840	1.06E-09	8.20E-07
RknMes02_051430	AT5G23960.2	0	cassava4.1_021437m	terpene synthase 21///Terpenoid cyclases/Protein prenyltransf	4.821	8.71E-07	2.45E-05
RknMes02_024963	AT5G59220.1	1.00E-12	cassava4.1_007913m	Protein phosphatase 2C family protein///highly ABA-induced	4.797	1.42E-07	8.83E-06
RknMes02_039460	AT5G59220.1	1.00E-25	cassava4.1_010060m	Protein phosphatase 2C family protein///highly ABA-induced	4.796	2.21E-06	4.47E-05
RknMes02_030127	AT1G60420.1	2.00E-33	cassava4.1_008371m	DC1 domain-containing protein	4.789	2.92E-06	5.30E-05
RknMes02_035128	AT1G44446.3	2.00E-34	cassava4.1_023978m	Pheophorbide a oxygenase family protein with Rieske [2Fe-2S	4.785	5.42E-06	8.07E-05
RknMes02_056689	AT3G59850.1	5.60E-45	cassava4.1_030364m	Pectin lyase-like superfamily protein	4.766	3.36E-08	4.17E-06
RknMes02_023528			cassava4.1_007913m		4.718	1.04E-06	2.74E-05
RknMes02_036024	AT5G64750.1	5.00E-38	cassava4.1_007311m	ethylene response factor 110///Unknown///Integrase-type DN	4.671	3.12E-06	5.55E-05
RknMes02_018746			cassava4.1_010060m		4.638	3.80E-06	6.38E-05
RknMes02_047856	AT5G03850.1	1.00E-21		Nucleic acid-binding, OB-fold-like protein	4.621	2.84E-06	5.20E-05
RknMes02_055947	AT1G16770.1	0	cassava4.1_029113m	AT1G16770.1	4.608	4.98E-09	1.54E-06
RknMes02_042836	AT5G03850.1	1.00E-21		Nucleic acid-binding, OB-fold-like protein	4.509	2.31E-06	4.59E-05
RknMes02_050301	AT5G66110.1	0	cassava4.1_018348m	Heavy metal transport/detoxification superfamily protein	4.508	2.01E-06	4.19E-05
RknMes02_013832			cassava4.1_015049m		4.416	2.96E-07	1.30E-05
RknMes02_052192	AT4G35690.1	0	cassava4.1_022736m	Arabidopsis protein of unknown function (DUF241)///Unknov	4.412	1.53E-06	3.50E-05
RknMes02_054543	AT1G60190.1	0	cassava4.1_026727m	ARM repeat superfamily protein	4.401	1.50E-06	3.46E-05
RknMes02_024608	AT3G04620.1	0	cassava4.1_023523m	Alba DNA/RNA-binding protein	4.384	1.13E-06	2.89E-05
RknMes02_022851	AT3G47800.1	0	cassava4.1_025611m	Galactose mutarotase-like superfamily protein	4.292	2.62E-09	1.18E-06
RknMes02_014532	AT1G17020.1	2.00E-22	cassava4.1_010623m	2-oxoglutarate (2OG) and Fe(II)-dependent oxygenase superfa	4.222	1.64E-06	3.67E-05
RknMes02_039174	AT1G17020.1	3.00E-28	cassava4.1_010623m	2-oxoglutarate (2OG) and Fe(II)-dependent oxygenase superfa	4.219	1.29E-06	3.15E-05
RknMes02_002504	AT4G27450.1	8.00E-25	cassava4.1_014315m	Aluminium induced protein with YGL and LRDR motifs	4.213	6.00E-07	1.91E-05
RknMes02_016407	AT3G61890.1	1.00E-12	cassava4.1_015049m	homeobox 12///homeobox 7	4.209	3.26E-07	1.38E-05
RknMes02_057726			cassava4.1_032036m		4.203	1.46E-06	3.39E-05
RknMes02_038567					4.179	1.08E-06	2.80E-05
RknMes02_001296	AT1G07430.1	2.00E-12	cassava4.1_008067m	highly ABA-induced PP2C gene 2	4.178	4.03E-08	4.53E-06
RknMes02_042075	AT5G43150.1	3.00E-06	cassava4.1_020299m	unknown protein; FUNCTIONS IN: molecular function unkn	4.147	5.70E-10	5.85E-07
RknMes02_058932	AT5G43870.1	1.00E-26	cassava4.1_034018m	Plant protein of unknown function (DUF828) with plant pleck	4.140	2.06E-08	3.25E-06
RknMes02_016270					4.127	3.05E-08	3.94E-06
RknMes02_007842	AT2G29380.1	7.00E-27	cassava4.1_013372m	highly ABA-induced PP2C gene 3	4.113	3.73E-08	4.40E-06
RknMes02_009448	AT1G05650.1	6.00E-30	cassava4.1_031277m	Pectin lyase-like superfamily protein	4.101	2.11E-06	4.33E-05
RknMes02_021498	AT3G56400.1	2.00E-35	cassava4.1_033846m	WRKY DNA-binding protein 70	4.084	2.48E-07	1.18E-05
RknMes02_025883	AT4G05120.1	0	cassava4.1_008484m	Major facilitator superfamily protein	4.053	1.39E-06	3.31E-05
RknMes02_051397	AT4G35690.1	2.00E-30	cassava4.1_021395m	Arabidopsis protein of unknown function (DUF241)///Unknov	4.043	6.70E-06	9.31E-05
RknMes02_058273	AT1G35910.1	0	cassava4.1_032922m	Haloacid dehalogenase-like hydrolase (HAD) superfamily pro	4.020	8.57E-07	2.43E-05
RknMes02_053675	AT5G13910.1	1.00E-25	cassava4.1_025242m	Integrase-type DNA-binding superfamily protein	4.010	1.39E-06	3.32E-05
RknMes02_012739	AT2G18550.1	3.00E-32	cassava4.1_027319m	homeobox protein 21	3.982	1.32E-08	2.53E-06
RknMes02_053786	AT2G24130.1	0	cassava4.1_025435m	Leucine-rich receptor-like protein kinase family protein	3.973	8.68E-08	6.83E-06
RknMes02_007712	AT3G16240.1	4.00E-13	cassava4.1_014710m	delta tonoplast integral protein///Unknown	3.969	5.44E-06	8.08E-05
RknMes02_010463			cassava4.1_015049m		3.947	2.80E-07	1.26E-05
RknMes02_050561	AT3G04620.1	2.00E-33	cassava4.1_019564m	Alba DNA/RNA-binding protein	3.881	1.61E-07	9.31E-06
RknMes02_024402	AT4G01970.1	0	cassava4.1_028359m	stachyose synthase	3.859	1.90E-06	4.04E-05
RknMes02_026845	AT4G30780.1	0	cassava4.1_005969m	unknown protein; BEST Arabidopsis thaliana protein match i	3.837	8.93E-08	6.93E-06
RknMes02_041021	AT5G15190.2	4.00E-08	cassava4.1_019923m	AT5G15190.1///Unknown///"unknown protein; FUNCTIONS	3.815	2.56E-06	4.87E-05
RknMes02_013552	AT3G11410.1	6.00E-06	cassava4.1_008067m	protein phosphatase 2CA	3.807	5.16E-07	1.75E-05
RknMes02_033475	AT4G26080.1	4.00E-22	cassava4.1_020355m	Protein phosphatase 2C family protein	3.765	2.78E-07	1.25E-05
RknMes02_004327	AT5G40390.1	4.00E-33	cassava4.1_002019m	Raffinose synthase family protein	3.726	4.64E-07	1.66E-05
RknMes02_042961					3.717	1.03E-08	2.18E-06
RknMes02_049529	AT3G54420.1	0	cassava4.1_014554m	homolog of carrot EP3-3 chitinase	3.678	1.95E-06	4.12E-05
RknMes02_013327	AT2G24100.1	8.00E-06	cassava4.1_005969m	Unknown///AT2G24100.1///unknown protein; BEST Arabido	3.644	3.34E-07	1.40E-05
RknMes02_058176	AT1G29670.1	0	cassava4.1_032751m	GDSL-like Lipase/Acylhydrolase superfamily protein	3.642	5.51E-07	1.82E-05
RknMes02_051310	AT5G14650.1	0	cassava4.1_021247m	Pectin lyase-like superfamily protein	3.625	1.40E-07	8.77E-06
RknMes02_010887	AT2G43590.1	2.00E-12	cassava4.1_014554m	Chitinase family protein	3.623	3.71E-06	6.27E-05
RknMes02_052991	AT1G17020.1	0	cassava4.1_024085m	2-oxoglutarate (2OG) and Fe(II)-dependent oxygenase superfa	3.614	1.39E-06	3.32E-05
RknMes02_056363	AT3G59440.1	6.00E-06	cassava4.1_029796m	Calcium-binding EF-hand family protein	3.614	1.27E-06	3.12E-05
RknMes02_016408	AT4G36470.1	6.00E-09	cassava4.1_010155m	S-adenosyl-L-methionine-dependent methyltransferases super	3.602	1.47E-07	9.03E-06
RknMes02_000783	AT4G27460.1	1.00E-32	cassava4.1_009282m	CBS domain-containing protein	3.586	4.23E-06	6.83E-05
RknMes02_006260			cassava4.1_005969m		3.552	3.60E-07	1.45E-05
RknMes02_010763			cassava4.1_014981m		3.547	6.27E-07	1.98E-05
RknMes02_039645	AT1G74930.1	3.00E-29	cassava4.1_018072m	Integrase-type DNA-binding superfamily protein	3.489	2.07E-07	1.07E-05
RknMes02_004093	AT3G10340.1	3.00E-12	cassava4.1_003117m	phenylalanine ammonia-lyase 2///phenylalanine ammonia-lyas	3.477	2.55E-06	4.86E-05
RknMes02_046170	AT2G43870.1	7.00E-11		Pectin lyase-like superfamily protein	3.476	5.77E-07	1.88E-05
RknMes02_055484	AT5G60520.1	0	cassava4.1_028326m	Late embryogenesis abundant (LEA) protein-related	3.451	5.62E-10	5.85E-07
RknMes02_050361	AT5G50720.1	1.40E-45	cassava4.1_018636m	HVA22 homologue E	3.420	4.80E-08	4.95E-06
RknMes02_037324	AT5G53750.1	4.00E-12	cassava4.1_009282m	CBS domain-containing protein///Cystathionine beta-synthase	3.411	6.18E-06	8.81E-05
RknMes02_055925	AT4G34135.1	0	cassava4.1_029076m	UDP-glucosyltransferase 73B2///UDP-glucosyl transferase 73	3.411	7.19E-09	1.80E-06
RknMes02_033796	AT5G53750.1	4.00E-13	cassava4.1_009282m	CBS domain-containing protein///Cystathionine beta-synthase	3.407	6.62E-06	9.24E-05
RknMes02_049597	AT3G61890.1	0	cassava4.1_014981m	homeobox 12///homeobox 7	3.366	9.07E-07	2.51E-05
RknMes02_049656	AT3G61890.1	0	cassava4.1_015295m	homeobox 12///homeobox 7	3.366	6.70E-07	2.07E-05
RknMes02_026630	AT2G17840.1	0	cassava4.1_008726m	Senescence/dehydration-associated protein-related	3.336	1.57E-06	3.56E-05
RknMes02_013666	AT3G47950.1	1.00E-12	cassava4.1_001144m	H(+)-ATPase 11///H(+)-ATPase 4	3.333	2.95E-06	5.34E-05
RknMes02_058983	AT1G24530.1	0	cassava4.1_034104m	Transducin/WD40 repeat-like superfamily protein	3.325	2.37E-07	1.15E-05
RknMes02_054233	AT5G42200.1	2.00E-33	cassava4.1_026205m	RING/U-box superfamily protein	3.304	4.88E-08	4.97E-06
RknMes02_051493	AT3G07510.2	2.00E-07	cassava4.1_021534m	unknown protein; FUNCTIONS IN: molecular function unkn	3.301	3.07E-08	3.95E-06
RknMes02_051011	AT3G48660.1	2.00E-28	cassava4.1_020761m	related to ABI3/VP1 2///Protein of unknown function (DUF 3	3.298	3.77E-07	1.48E-05
RknMes02_017072	AT3G10340.1	0	cassava4.1_003117m	phenylalanine ammonia-lyase 2///phenylalanine ammonia-lyas	3.285	1.43E-06	3.35E-05
RknMes02_050463	AT2G34340.1	1.00E-28	cassava4.1_019132m	Protein of unknown function, DUF584	3.279	2.01E-09	1.09E-06
RknMes02_050357	AT5G50720.1	1.40E-45	cassava4.1_018619m	HVA22 homologue E	3.228	2.54E-07	1.20E-05
RknMes02_026846	AT2G24100.1	7.00E-13	cassava4.1_005969m	Unknown///AT2G24100.1///unknown protein; BEST Arabido	3.188	6.99E-08	6.05E-06
RknMes02_048776	AT2G19810.1	0	cassava4.1_009450m	Zinc finger C-x8-C-x5-C-x3-H type family protein///CCCH-ty	3.166	4.21E-07	1.58E-05
RknMes02_050840	AT4G22190.1	1.00E-06	cassava4.1_020395m	unknown protein; Has 283 Blast hits to 154 proteins in 44 spe	3.165	4.52E-07	1.64E-05
RknMes02_010899	AT5G53750.1	3.00E-15	cassava4.1_009282m	CBS domain-containing protein///Cystathionine beta-synthase	3.140	2.00E-06	4.16E-05
RknMes02_048414	AT1G72770.3	0	cassava4.1_005959m	homology to ABI1	3.128	8.72E-10	7.37E-07

RknMes02_039925	AT5G55090.1	0	cassava4.1_008192m	NPK1-related protein kinase 2///Unknown///mitogen-activate	3.110	5.73E-07	1.87E-05
RknMes02_034016	AT4G12300.1	0	cassava4.1_005921m	cytochrome P450, family 706, subfamily A, polypeptide 4	3.109	1.40E-06	3.33E-05
RknMes02_016327	AT4G36470.1	7.00E-07	cassava4.1_010155m	S-adenosyl-L-methionine-dependent methyltransferases super	3.084	1.99E-06	4.16E-05
RknMes02_055658	AT5G52570.1	0	cassava4.1_028637m	beta-carotene hydroxylase 2	3.073	3.42E-06	5.91E-05
RknMes02_049247	AT4G36830.1	0	cassava4.1_012827m	GNS1/SUR4 membrane protein family	3.047	4.74E-06	7.36E-05
RknMes02_037858	AT3G51250.1	0	cassava4.1_008726m	Senescence/dehydration-associated protein-related	3.043	4.41E-06	7.00E-05
RknMes02_056065	AT3G22830.1	0	cassava4.1_029306m	heat shock transcription factor A7A///heat shock transcriptio	3.033	1.13E-06	2.88E-05
RknMes02_055209	AT4G35720.1	1.00E-39	cassava4.1_027864m	Arabidopsis protein of unknown function (DUF241)	3.021	5.99E-08	5.57E-06
RknMes02_052856	AT1G32583.1	2.00E-35	cassava4.1_023864m	AT1G32583.1	3.002	6.23E-06	8.85E-05
RknMes02_039824	AT1G60190.1	9.00E-39	cassava4.1_002876m	ARM repeat superfamily protein	3.001	3.62E-07	1.46E-05
RknMes02_032464	AT4G05070.1	8.00E-07		Unknown	2.998	2.83E-06	5.20E-05
RknMes02_049457	AT1G49320.1	0	cassava4.1_014108m	unknown seed protein like 1	2.994	3.05E-07	1.33E-05
RknMes02_030281	AT4G02040.1	6.00E-16	cassava4.1_016790m	unknown protein; FUNCTIONS IN: molecular function unkn	2.946	6.52E-07	2.03E-05
RknMes02_058917	AT1G23550.1	0	cassava4.1_033992m	similar to RCD one 2	2.915	5.41E-06	8.05E-05
RknMes02_005365	AT2G37980.1	1.00E-14	cassava4.1_030087m	O-fucosyltransferase family protein	2.893	2.09E-07	1.07E-05
RknMes02_014452	AT3G05500.1	1.00E-31	cassava4.1_028020m	Rubber elongation factor protein (REF)	2.886	8.41E-11	2.44E-07
RknMes02_001499	AT5G54160.1	1.00E-21	cassava4.1_013376m	O-methyltransferase 1	2.881	6.28E-06	8.90E-05
RknMes02_025998	AT2G18193.1	3.00E-28	cassava4.1_009105m	P-loop containing nucleoside triphosphate hydrolases superfa	2.878	2.15E-06	4.38E-05
RknMes02_054668	AT4G36740.1	0	cassava4.1_026937m	homeobox protein 40///homeobox protein 21	2.874	4.30E-09	1.46E-06
RknMes02_030412	AT4G26140.1	0	cassava4.1_001733m	beta-galactosidase 12	2.866	2.13E-08	3.30E-06
RknMes02_017212	AT3G22800.1	2.00E-26	cassava4.1_020058m	thiazole biosynthetic enzyme, chloroplast (ARA6) (THI1) (TH	2.861	3.46E-06	5.96E-05
RknMes02_011864			cassava4.1_029076m		2.845	2.23E-08	3.36E-06
RknMes02_048720	AT1G03790.1	0	cassava4.1_008973m	Zinc finger C-x8-C-x5-C-x3-H type family protein	2.844	4.36E-08	4.76E-06
RknMes02_003789	AT3G26170.1	8.00E-15	cassava4.1_005921m	cytochrome P450, family 71, subfamily B, polypeptide 2	2.836	1.20E-06	3.00E-05
RknMes02_057053	AT1G06620.1	0	cassava4.1_030971m	2-oxoglutarate (2OG) and Fe(II)-dependent oxygenase superfa	2.807	1.34E-06	3.24E-05
RknMes02_007438	AT3G13750.1	2.00E-40	cassava4.1_001733m	beta galactosidase 1///beta-galactosidase 12///beta-galactosida	2.800	1.07E-07	7.61E-06
RknMes02_029587	AT4G27450.1	0	cassava4.1_014539m	Aluminium induced protein with YGL and LRDR motifs	2.796	6.84E-08	5.99E-06
RknMes02_024282			cassava4.1_027300m		2.786	5.31E-07	1.79E-05
RknMes02_013200	AT4G34131.1	3.00E-08	cassava4.1_029076m	UDP-glucosyl transferase 73B3	2.784	2.09E-08	3.27E-06
RknMes02_039868	AT3G04070.2	8.00E-16	cassava4.1_010010m	NAC domain containing protein 47	2.771	1.69E-07	9.58E-06
RknMes02_056016	AT2G21490.1	1.00E-35	cassava4.1_029213m	dehydrin LEA	2.763	7.32E-11	2.44E-07
RknMes02_050337	AT3G21510.1	0	cassava4.1_018535m	histidine-containing phosphotransmitter 2///histidine-containi	2.751	8.00E-08	6.46E-06
RknMes02_053272	AT5G60520.1	0	cassava4.1_024577m	Late embryogenesis abundant (LEA) protein-related	2.738	4.57E-08	4.86E-06
RknMes02_025374	AT1G69840.7	0	cassava4.1_013311m	SPFH/Band 7/PHB domain-containing membrane-associated	2.735	2.53E-06	4.83E-05
RknMes02_056529	AT2G37980.1	0	cassava4.1_030087m	O-fucosyltransferase family protein	2.722	1.36E-06	3.27E-05
RknMes02_010471	AT3G54100.1	1.00E-09	cassava4.1_030087m	O-fucosyltransferase family protein	2.717	2.45E-06	4.75E-05
RknMes02_049259	AT1G28310.2	4.00E-35	cassava4.1_012902m	Dof-type zinc finger DNA-binding family protein	2.697	5.35E-06	7.98E-05
RknMes02_055888			cassava4.1_029018m		2.686	3.80E-08	4.44E-06
RknMes02_040118	AT3G07130.1	0	cassava4.1_005206m	purple acid phosphatase 15	2.667	4.95E-06	7.59E-05
RknMes02_020487					2.658	8.10E-08	6.48E-06
RknMes02_039697	AT5G57560.1	0	cassava4.1_013165m	Xyloglucan endotransglucosylase/hydrolase family protein///x	2.654	3.94E-06	6.53E-05
RknMes02_003559	AT5G56870.1	2.00E-34	cassava4.1_001503m	beta-galactosidase 4	2.653	2.69E-08	3.67E-06
RknMes02_050758	AT5G43150.1	1.00E-11	cassava4.1_020196m	unknown protein; FUNCTIONS IN: molecular function unkn	2.645	3.14E-08	3.99E-06
RknMes02_039276	AT5G06300.1	0	cassava4.1_013240m	Putative lysine decarboxylase family protein	2.642	4.95E-06	7.58E-05
RknMes02_051754	AT2G44940.1	0	cassava4.1_021971m	Integrase-type DNA-binding superfamily protein	2.640	1.80E-07	9.93E-06
RknMes02_013897	AT3G13750.1	3.00E-24	cassava4.1_001503m	beta galactosidase 1///beta-galactosidase 12///beta-galactosida	2.634	1.72E-08	2.92E-06
RknMes02_023600	AT1G45230.1	2.00E-20		Protein of unknown function (DUF3223)	2.621	7.31E-07	2.20E-05
RknMes02_057295	AT4G37770.1	0	cassava4.1_031356m	1-amino-cyclopropane-1-carboxylate synthase 8	2.608	2.55E-07	1.20E-05
RknMes02_008099	AT3G52840.1	1.00E-36	cassava4.1_001503m	beta-galactosidase 2	2.608	9.88E-09	2.13E-06
RknMes02_024400					2.592	5.85E-07	1.88E-05
RknMes02_033557	AT2G36630.1	0	cassava4.1_007299m	Sulfite exporter TauE/SafE family protein	2.589	1.20E-07	8.01E-06
RknMes02_044166					2.589	2.36E-06	4.66E-05
RknMes02_013420			cassava4.1_010019m		2.573	6.00E-09	1.66E-06
RknMes02_004720	AT5G56870.1	8.00E-29	cassava4.1_001503m	beta-galactosidase 4	2.562	1.89E-08	3.08E-06
RknMes02_038342	AT1G08570.4	2.00E-43	cassava4.1_013279m	atypical CYS HIS rich thioredoxin 4	2.555	5.29E-06	7.93E-05
RknMes02_007072	AT1G62660.1	9.00E-26	cassava4.1_004675m	Glycosyl hydrolases family 32 protein	2.547	4.78E-07	1.69E-05
RknMes02_057990	AT2G21610.1	0	cassava4.1_032455m	pectinesterase 11	2.530	4.26E-08	4.68E-06
RknMes02_033631	AT4G35770.1	0	cassava4.1_017198m	Rhodanese/Cell cycle control phosphatase superfamily protein	2.525	1.00E-08	2.13E-06
RknMes02_030608	AT2G40610.1	0	cassava4.1_014549m	expansin A8	2.521	5.36E-08	5.27E-06
RknMes02_008470	AT2G36630.1	1.00E-36	cassava4.1_007299m	Sulfite exporter TauE/SafE family protein	2.512	7.14E-08	6.14E-06
RknMes02_002662	AT4G02700.1	2.00E-32	cassava4.1_003230m	sulfate transporter 3;2	2.511	2.99E-07	1.31E-05
RknMes02_003707	AT4G26140.2	9.00E-41	cassava4.1_001503m	beta-galactosidase 12	2.511	1.24E-09	8.44E-07
RknMes02_048392	AT2G38940.1	0	cassava4.1_005715m	phosphate transporter 1;7///Unknown///phosphate transporter	2.503	6.58E-08	5.87E-06
RknMes02_000880	AT1G71960.1	1.00E-09	cassava4.1_004508m	ATP-binding casette family G25///Unknown	2.500	7.12E-06	9.71E-05
RknMes02_022438	AT4G26140.2	0	cassava4.1_001733m	beta-galactosidase 12	2.487	3.50E-06	6.01E-05
RknMes02_002754	AT1G23870.1	4.00E-35	cassava4.1_001541m	trehalose-phosphatase/synthase 9	2.468	1.60E-06	3.61E-05
RknMes02_002166	AT5G20250.4	2.00E-31	cassava4.1_002004m	Raffinose synthase family protein	2.464	2.48E-07	1.18E-05
RknMes02_045701					2.455	5.17E-06	7.82E-05
RknMes02_039735	AT1G71120.1	0	cassava4.1_034345m	GDSL-motif lipase/hydrolase 6	2.449	4.69E-08	4.92E-06
RknMes02_032798	AT2G26070.1	0	cassava4.1_014738m	Protein of unknown function (DUF778)	2.436	1.13E-06	2.89E-05
RknMes02_032910	AT3G10420.2	2.00E-39	cassava4.1_002788m	P-loop containing nucleoside triphosphate hydrolases superfa	2.434	2.33E-07	1.14E-05
RknMes02_013976	AT1G59740.1	4.00E-12	cassava4.1_003848m	Major facilitator superfamily protein	2.434	5.49E-06	8.12E-05
RknMes02_003889	AT3G04300.1	4.00E-36	cassava4.1_023553m	RmlC-like cupins superfamily protein	2.424	3.45E-07	1.42E-05
RknMes02_050288	AT3G21510.1	0	cassava4.1_018311m	histidine-containing phosphotransmitter 2///histidine-containi	2.409	4.98E-07	1.72E-05
RknMes02_003801	AT3G13750.1	4.00E-28	cassava4.1_001503m	beta galactosidase 1///beta-galactosidase 12///beta-galactosida	2.407	5.00E-08	5.03E-06
RknMes02_031931	AT4G01870.1	0	cassava4.1_034137m	tolB protein-related	2.395	4.83E-07	1.69E-05
RknMes02_017319	AT3G51895.1	0	cassava4.1_003230m	sulfate transporter 3;1	2.391	3.66E-06	6.21E-05
RknMes02_000146	AT2G46270.1	3.00E-31	cassava4.1_008455m	G-box binding factor 3	2.390	1.72E-08	2.92E-06
RknMes02_036684	AT5G54240.1	0	cassava4.1_013599m	Protein of unknown function (DUF1223)	2.390	7.58E-07	2.26E-05
RknMes02_020844	AT2G46270.2	2.00E-35	cassava4.1_008455m	G-box binding factor 3	2.383	1.71E-07	9.64E-06
RknMes02_033789	AT1G73480.1	6.00E-09	cassava4.1_006764m	Unknown///alpha/beta-Hydrolases superfamily protein	2.380	1.78E-06	3.88E-05
RknMes02_048226	AT3G16380.1	0	cassava4.1_004058m	poly(A) binding protein 6	2.380	1.86E-07	1.01E-05
RknMes02_004901	AT3G52840.1	3.00E-38	cassava4.1_001733m	beta-galactosidase 2	2.370	3.52E-06	6.04E-05
RknMes02_057376			cassava4.1_031490m		2.369	1.06E-07	7.53E-06
RknMes02_015088	AT1G13990.1	6.00E-18	cassava4.1_013257m	AT1G13990.1///unknown protein; FUNCTIONS IN: molecula	2.360	3.65E-07	1.46E-05
RknMes02_031868	AT1G59740.1	0	cassava4.1_003848m	Major facilitator superfamily protein	2.349	4.13E-06	6.72E-05
RknMes02_015109			cassava4.1_017002m		2.345	4.00E-09	1.42E-06
RknMes02_058864			cassava4.1_033900m		2.329	2.03E-06	4.21E-05
RknMes02_049315	AT1G13990.1	0	cassava4.1_013257m	AT1G13990.1///unknown protein; FUNCTIONS IN: molecula	2.322	1.81E-07	9.93E-06
RknMes02_038531			cassava4.1_019298m		2.318	1.11E-06	2.84E-05
RknMes02_002071			cassava4.1_017002m		2.314	5.51E-09	1.60E-06
RknMes02_049538	AT1G74650.1	0	cassava4.1_014624m	myb domain protein 31///myb domain protein 96	2.312	1.26E-08	2.44E-06
RknMes02_011243	AT1G23730.1	8.00E-12	cassava4.1_030179m	beta carbonic anhydrase 3	2.292	2.39E-06	4.69E-05
RknMes02_051021			cassava4.1_020782m		2.287	1.81E-06	3.92E-05
RknMes02_048662	AT1G03220.1	0	cassava4.1_008441m	Eukaryotic aspartyl protease family protein	2.286	4.05E-06	6.64E-05
RknMes02_031789	AT1G68238.1	2.00E-08	cassava4.1_019868m	Unknown///AT1G68238.1	2.285	1.42E-06	3.35E-05
RknMes02_009426	AT5G14860.1	2.90E-44	cassava4.1_022533m	UDP-Glycosyltransferase superfamily protein	2.277	3.24E-06	5.69E-05
RknMes02_025517	AT5G59720.1	1.00E-08		heat shock protein 18.2///HSP20-like chaperones superfamily	2.269	5.67E-06	8.31E-05
RknMes02_028518	AT5G05690.2	0	cassava4.1_006864m	Cytochrome P450 superfamily protein	2.265	1.64E-08	2.84E-06
RknMes02_038250	AT1G72200.1	0	cassava4.1_008325m	RING/U-box superfamily protein	2.261	6.14E-07	1.94E-05
RknMes02_014156			cassava4.1_023613m		2.260	3.11E-08	3.98E-06
RknMes02_031316	AT5G13200.1	0	cassava4.1_013991m	GRAM domain family protein	2.258	5.05E-06	7.70E-05
RknMes02_015540	AT3G10420.2	1.00E-32	cassava4.1_002788m	P-loop containing nucleoside triphosphate hydrolases superfa	2.254	4.66E-08	4.91E-06
RknMes02_036162	AT1G21790.1	0	cassava4.1_024644m	TRAM, LAG1 and CLN8 (TLC) lipid-sensing domain contai	2.241	1.85E-09	1.04E-06
RknMes02_018836			cassava4.1_019524m		2.238	7.76E-09	1.85E-06
RknMes02_043452	AT5G05480.1	2.00E-17		Peptide-N4-(N-acetyl-beta-glucosaminyl)asparagine amidase	2.229	5.53E-06	8.17E-05
RknMes02_057627	AT4G32480.1	0	cassava4.1_031890m	Protein of unknown function (DUF506)	2.217	4.63E-07	1.66E-05
RknMes02_050489	AT3G60370.1	4.00E-06	cassava4.1_019262m	FKBP-like peptidyl-prolyl cis-trans isomerase family protein	2.208	4.01E-07	1.54E-05
RknMes02_037868	AT1G53210.1	0	cassava4.1_004277m	sodium/calcium exchanger family protein / calcium-binding E	2.191	4.04E-10	4.82E-07
RknMes02_031268			cassava4.1_010104m		2.188	2.80E-08	3.75E-06
RknMes02_041396					2.185	5.72E-07	1.87E-05
RknMes02_010022	AT1G23870.1	4.00E-38	cassava4.1_029065m	trehalose-phosphatase/synthase 9	2.178	5.82E-06	8.45E-05
RknMes02_023840	AT2G41430.5	5.00E-13	cassava4.1_019973m	dehydration-induced protein (ERD15)///Protein containing PA	2.175	2.65E-07	1.23E-05
RknMes02_010968	AT4G33580.2	5.00E-10	cassava4.1_030179m	beta carbonic anhydrase 5	2.172	4.41E-08	4.79E-06
RknMes02_014344	AT2G38000.1	5.00E-12	cassava4.1_008257m	chaperone protein dnaJ-related	2.161	2.18E-07	1.10E-05
RknMes02_004784	AT4G01870.1	0	cassava4.1_034137m	tolB protein-related	2.161	3.80E-07	1.49E-05
RknMes02_056263	AT2G34930.1	0	cassava4.1_029622m	disease resistance family protein / LRR family protein	2.150	2.46E-07	1.18E-05
RknMes02_057407	AT2G23240.1	5.00E-27	cassava4.1_031534m	Plant EC metallothionein-like protein, family 15	2.148	6.83E-09	1.76E-06
RknMes02_007690	AT1G21410.1	8.00E-06	cassava4.1_019051m	F-box/RNI-like superfamily protein	2.146	2.73E-06	5.08E-05
RknMes02_055023	AT4G25250.1	1.00E-36	cassava4.1_027551m	Plant invertase/pectin methylesterase inhibitor superfamily pr	2.140	9.78E-07	2.63E-05
RknMes02_050020	AT5G51160.1	2.00E-19	cassava4.1_017002m	Ankyrin repeat family protein	2.140	2.14E-09	1.12E-06
RknMes02_006585	AT3G10420.2	2.00E-26	cassava4.1_002788m	P-loop containing nucleoside triphosphate hydrolases superfa	2.140	5.24E-07	1.77E-05
RknMes02_013711					2.140	2.81E-06	5.17E-05
RknMes02_013908			cassava4.1_011360m		2.132	1.87E-06	4.01E-05
RknMes02_047242					2.130	1.41E-06	3.33E-05
RknMes02_008284	AT1G70290.1	2.00E-41	cassava4.1_001537m	trehalose-6-phosphatase synthase S8	2.128	1.63E-07	9.37E-06
RknMes02_003150	AT5G08790.1	2.00E-20	cassava4.1_012943m	NAC (No Apical Meristem) domain transcriptional regulator	2.125	1.46E-06	3.40E-05

RknMes02_006377	AT1G64060.1	1.00E-34	cassava4.1_001193m	respiratory burst oxidase protein F	2.122	2.63E-07	1.22E-05
RknMes02_027786	AT1G22340.1	0	cassava4.1_005558m	UDP-glucosyl transferase 85A7///UDP-glucosyl transferase 85	2.110	3.59E-07	1.45E-05
RknMes02_046123			cassava4.1_030952m		2.110	3.65E-07	1.46E-05
RknMes02_049851	AT3G22160.1	2.00E-14	cassava4.1_016216m	VQ motif-containing protein///Unknown	2.106	5.92E-06	8.55E-05
RknMes02_040089	AT5G07680.2	2.00E-30	cassava4.1_010869m	NAC domain containing protein 80///NAC domain containing	2.103	5.68E-08	5.42E-06
RknMes02_006698	AT4G21920.1	4.00E-08	cassava4.1_030675m	unknown protein; FUNCTIONS IN: molecular function unkn	2.101	4.51E-06	7.12E-05
RknMes02_056527	AT5G65300.1	6.00E-11	cassava4.1_030085m	AT5G65300.1	2.097	6.36E-06	8.97E-05
RknMes02_052004	AT1G32700.1	0	cassava4.1_022404m	PLATZ transcription factor family protein	2.096	9.51E-09	2.10E-06
RknMes02_005199	AT5G20250.4	0	cassava4.1_002004m	Raffinose synthase family protein	2.087	4.00E-06	6.59E-05
RknMes02_051750	AT1G58330.1	4.00E-14	cassava4.1_021958m	RESPONSE TO ABA AND SALT 1	2.078	4.13E-07	1.56E-05
RknMes02_005901	AT1G09020.1	2.00E-22	cassava4.1_008100m	homolog of yeast sucrose nonfermenting 4	2.070	3.30E-07	1.39E-05
RknMes02_031259	AT5G65280.1	0	cassava4.1_008284m	GCR2-like 1	2.061	2.72E-07	1.24E-05
RknMes02_003688	AT1G13740.1	5.00E-07	cassava4.1_010125m	Unknown///ABI five binding protein 2	2.058	8.94E-08	6.93E-06
RknMes02_024239	AT1G67300.2	3.00E-39	cassava4.1_006947m	Major facilitator superfamily protein	2.054	1.48E-08	2.65E-06
RknMes02_029383	AT2G41190.1	0	cassava4.1_007924m	Transmembrane amino acid transporter family protein	2.052	3.41E-06	5.90E-05
RknMes02_006060	AT1G47980.1	1.00E-34	cassava4.1_012167m	unknown protein; FUNCTIONS IN: molecular function unkn	2.048	3.98E-06	6.57E-05
RknMes02_024352	AT1G68820.1	0	cassava4.1_026740m	Transmembrane Fragile-X-F-associated protein	2.046	9.49E-08	7.18E-06
RknMes02_053159	AT4G16835.1	3.00E-14	cassava4.1_024383m	Tetratricopeptide repeat (TPR)-like superfamily protein	2.046	1.44E-07	8.93E-06
RknMes02_010945	AT5G15410.1	0	cassava4.1_005026m	Cyclic nucleotide-regulated ion channel family protein	2.041	2.64E-06	4.97E-05
RknMes02_048044	AT3G09840.1	0	cassava4.1_001970m	cell division cycle 48///Unknown///"ATPase, AAA-type, CDC	2.041	2.88E-06	5.26E-05
RknMes02_013377	AT5G05690.1	2.80E-45	cassava4.1_006864m	Cytochrome P450 superfamily protein	2.038	8.62E-08	6.81E-06
RknMes02_013722	AT5G20250.4	1.00E-08	cassava4.1_002004m	Raffinose synthase family protein	2.034	3.53E-06	6.04E-05
RknMes02_028371	AT4G01870.1	0	cassava4.1_034137m	tolB protein-related	2.026	4.85E-06	7.50E-05
RknMes02_020779	AT1G09020.1	0	cassava4.1_008100m	homolog of yeast sucrose nonfermenting 4	2.020	3.91E-06	6.49E-05
RknMes02_012344	AT5G53450.2	7.00E-15	cassava4.1_003020m	OBP3-responsive gene 1	2.017	6.58E-06	9.19E-05
RknMes02_007075	AT1G73480.1	8.00E-38	cassava4.1_006764m	Unknown///alpha/beta-Hydrolases superfamily protein	2.012	2.32E-06	4.60E-05
RknMes02_009458			cassava4.1_002004m		2.006	2.39E-06	4.69E-05
RknMes02_057542	AT3G20660.1	0	cassava4.1_031755m	organic cation/carnitine transporter4	1.988	4.22E-07	1.58E-05
RknMes02_021431					1.981	3.48E-07	1.42E-05
RknMes02_049918	AT3G50830.1	0	cassava4.1_016552m	cold-regulated 413-plasma membrane 2	1.977	1.01E-06	2.69E-05
RknMes02_036711	AT1G72770.3	0	cassava4.1_004984m	homology to ABI1	1.959	8.02E-10	6.97E-07
RknMes02_040003	AT1G67300.1	0	cassava4.1_006947m	Major facilitator superfamily protein	1.956	5.93E-08	5.54E-06
RknMes02_007559	AT1G60140.1	0	cassava4.1_001517m	trehalose phosphate synthase///trehalose-phosphatase/synthas	1.956	2.11E-06	4.33E-05
RknMes02_036205	AT3G06500.1	0	cassava4.1_002913m	Plant neutral invertase family protein	1.956	3.12E-09	1.24E-06
RknMes02_029387	AT5G67350.1	2.00E-38	cassava4.1_014725m	unknown protein; Has 1807 Blast hits to 1807 proteins in 277	1.956	1.44E-06	3.37E-05
RknMes02_000186	AT3G59140.1	1.00E-39	cassava4.1_000219m	multidrug resistance-associated protein 14///Unknown	1.953	9.21E-07	2.53E-05
RknMes02_013004	AT3G59140.1	2.00E-25	cassava4.1_000219m	multidrug resistance-associated protein 14///Unknown	1.948	5.27E-07	1.78E-05
RknMes02_006121			cassava4.1_010545m		1.946	1.19E-09	8.41E-07
RknMes02_005791	AT1G60140.1	0	cassava4.1_001537m	trehalose phosphate synthase///trehalose-phosphatase/synthas	1.938	1.57E-07	9.23E-06
RknMes02_050179	AT2G26540.1	0	cassava4.1_017765m	uroporphyrinogen-III synthase family protein	1.937	2.35E-06	4.65E-05
RknMes02_007343	AT2G26540.1	2.00E-07		uroporphyrinogen-III synthase family protein	1.930	1.22E-06	3.02E-05
RknMes02_012665	AT4G02050.1	1.00E-11		sugar transporter protein 7	1.929	4.49E-06	7.10E-05
RknMes02_035809			cassava4.1_012760m		1.926	1.27E-08	2.45E-06
RknMes02_049458	AT2G23620.1	0	cassava4.1_014110m	methyl esterase 1	1.910	2.52E-06	4.83E-05
RknMes02_001770	AT5G05690.3	0	cassava4.1_015740m	Cytochrome P450 superfamily protein	1.901	7.21E-08	6.16E-06
RknMes02_003522	AT5G02800.1	1.00E-39	cassava4.1_008502m	Protein kinase superfamily protein	1.897	5.82E-07	1.88E-05
RknMes02_006356	AT3G28510.1	0	cassava4.1_005875m	P-loop containing nucleoside triphosphate hydrolases superfa	1.893	4.33E-06	6.93E-05
RknMes02_032316	AT5G64700.1	0	cassava4.1_010374m	nodulin MtN21 /EamA-like transporter family protein	1.891	3.27E-06	5.73E-05
RknMes02_053711	AT1G60190.1	0	cassava4.1_025313m	ARM repeat superfamily protein	1.891	2.29E-08	3.36E-06
RknMes02_057805	AT3G24060.1	1.00E-13	cassava4.1_032165m	Plant self-incompatibility protein S1 family	1.889	3.68E-07	1.47E-05
RknMes02_004432	AT5G11420.1	2.00E-23	cassava4.1_010027m	Protein of unknown function, DUF642	1.885	5.92E-09	1.65E-06
RknMes02_031836	AT1G11360.4	5.00E-44	cassava4.1_014804m	Unknown///Adenine nucleotide alpha hydrolases-like superfa	1.884	5.65E-06	8.29E-05
RknMes02_047454	AT2G28680.1	4.00E-38	cassava4.1_010513m	RmlC-like cupins superfamily protein	1.883	4.10E-08	4.57E-06
RknMes02_010019			cassava4.1_002004m		1.871	6.27E-06	8.89E-05
RknMes02_053380	AT2G19330.1	0	cassava4.1_024749m	plant intracellular ras group-related LRR 6	1.863	1.59E-08	2.78E-06
RknMes02_006326	AT3G16350.1	1.00E-08	cassava4.1_010545m	Homeodomain-like superfamily protein	1.857	2.14E-10	3.62E-07
RknMes02_040383	AT5G53450.2	0	cassava4.1_003020m	OBP3-responsive gene 1	1.855	5.64E-06	8.29E-05
RknMes02_035500	AT2G42280.1	0	cassava4.1_010085m	basic helix-loop-helix (bHLH) DNA-binding superfamily prot	1.854	2.26E-08	3.36E-06
RknMes02_016900	AT1G01720.1	0	cassava4.1_013132m	NAC (No Apical Meristem) domain transcriptional regulator	1.853	2.31E-08	3.37E-06
RknMes02_035368	AT2G25625.2	8.00E-12	cassava4.1_027500m	AT2G25625.2///"unknown protein; FUNCTIONS IN: molecul	1.853	4.53E-07	1.64E-05
RknMes02_024994	AT1G77380.1	0	cassava4.1_006535m	amino acid permease 3	1.851	3.69E-09	1.37E-06
RknMes02_035498	AT2G40080.1	8.00E-28	cassava4.1_019555m	Protein of unknown function (DUF1313)	1.843	6.24E-08	5.71E-06
RknMes02_035900	AT1G45976.1	5.00E-12	cassava4.1_012855m	S-ribonuclease binding protein 1	1.843	8.90E-07	2.49E-05
RknMes02_047043	AT5G60900.1	1.00E-07		receptor-like protein kinase 1	1.833	2.74E-06	5.10E-05
RknMes02_049175	AT1G18460.1	0	cassava4.1_012397m	alpha/beta-Hydrolases superfamily protein	1.833	1.86E-08	3.04E-06
RknMes02_029530	AT2G23810.1	0	cassava4.1_013791m	tetraspanin11///tetraspanin8///tetraspanin7	1.829	1.37E-06	3.29E-05
RknMes02_052270	AT1G65570.1	0	cassava4.1_022864m	Pectin lyase-like superfamily protein	1.825	2.24E-07	1.12E-05
RknMes02_002996	AT5G60760.1	7.00E-26	cassava4.1_002581m	P-loop containing nucleoside triphosphate hydrolases superfa	1.824	5.33E-07	1.79E-05
RknMes02_049903	AT3G50830.1	0	cassava4.1_016484m	cold-regulated 413-plasma membrane 2	1.817	7.30E-07	2.20E-05
RknMes02_031151	AT3G20660.1	0	cassava4.1_025738m	organic cation/carnitine transporter4	1.816	9.98E-07	2.65E-05
RknMes02_054271	AT5G19650.1	1.00E-37	cassava4.1_026271m	ovate family protein 8	1.815	5.57E-06	8.20E-05
RknMes02_057004	AT3G59850.1	0	cassava4.1_030897m	Pectin lyase-like superfamily protein	1.813	2.43E-08	3.44E-06
RknMes02_012078					1.811	1.93E-06	4.09E-05
RknMes02_009909	AT4G20360.1	1.00E-12		RAB GTPase homolog E1B///Unknown///"Nucleic acid-bindi	1.808	3.81E-06	6.39E-05
RknMes02_000447	AT4G34480.1	0	cassava4.1_007157m	O-Glycosyl hydrolases family 17 protein///Glycosyl hydrolase	1.808	3.69E-09	1.37E-06
RknMes02_022445					1.803	2.96E-09	1.21E-06
RknMes02_023158	AT5G02230.2	8.00E-31	cassava4.1_013061m	Haloacid dehalogenase-like hydrolase (HAD) superfamily pro	1.794	2.38E-06	4.68E-05
RknMes02_050519			cassava4.1_019400m		1.791	3.26E-06	5.72E-05
RknMes02_019499					1.791	1.57E-07	9.25E-06
RknMes02_036224	AT1G69490.1	0	cassava4.1_013467m	NAC (No Apical Meristem) domain transcriptional regulator	1.790	4.34E-06	6.94E-05
RknMes02_046751	AT3G05890.1	5.00E-20		Low temperature and salt responsive protein family	1.788	1.31E-07	8.43E-06
RknMes02_027432	AT3G60290.1	3.00E-33	cassava4.1_012576m	2-oxoglutarate (2OG) and Fe(II)-dependent oxygenase superfa	1.788	3.00E-08	3.92E-06
RknMes02_025252	AT1G77380.1	0	cassava4.1_006535m	amino acid permease 3	1.787	2.25E-08	3.36E-06
RknMes02_058028	AT5G54570.1	0	cassava4.1_032518m	beta glucosidase 41	1.783	6.18E-07	1.95E-05
RknMes02_005864	AT5G02230.2	7.00E-08	cassava4.1_014451m	Haloacid dehalogenase-like hydrolase (HAD) superfamily pro	1.782	1.52E-06	3.49E-05
RknMes02_032596	AT3G56400.1	2.00E-42	cassava4.1_013417m	WRKY DNA-binding protein 70	1.775	4.11E-07	1.56E-05
RknMes02_024731	AT3G26770.1	5.00E-29	cassava4.1_017063m	Unknown///cassava4.1_020185m///NAD(P)-binding Rossman	1.771	1.41E-06	3.34E-05
RknMes02_047663					1.770	7.99E-07	2.33E-05
RknMes02_007011	AT3G01470.1	9.00E-31	cassava4.1_012760m	homeobox 1	1.770	1.46E-08	2.64E-06
RknMes02_039292	AT1G10380.1	0	cassava4.1_012756m	Putative membrane lipoprotein	1.766	1.53E-07	9.15E-06
RknMes02_049025	AT1G73920.1	0	cassava4.1_011251m	alpha/beta-Hydrolases superfamily protein	1.765	3.03E-09	1.23E-06
RknMes02_001338			cassava4.1_034118m		1.765	3.28E-06	5.74E-05
RknMes02_052293	AT3G52970.1	0	cassava4.1_022898m	cytochrome P450, family 76, subfamily G, polypeptide 1///"cy	1.755	1.39E-06	3.30E-05
RknMes02_039995	AT4G36990.1	0	cassava4.1_013174m	heat shock factor 4	1.751	5.70E-06	8.34E-05
RknMes02_053712	AT1G15930.2	6.00E-31	cassava4.1_025316m	Ribosomal protein L7Ae/L30e/S12e/Gadd45 family protein	1.742	3.97E-06	6.57E-05
RknMes02_004479	AT5G53450.2	7.00E-25	cassava4.1_003020m	OBP3-responsive gene 1	1.741	5.33E-07	1.79E-05
RknMes02_025563	AT4G37300.1	3.00E-17	cassava4.1_017664m	maternal effect embryo arrest 59	1.736	1.16E-07	7.87E-06
RknMes02_036843	AT2G30860.1	0	cassava4.1_016203m	glutathione S-transferase PHI 9	1.735	2.52E-06	4.83E-05
RknMes02_054521	AT3G48950.1	0	cassava4.1_026691m	Pectin lyase-like superfamily protein	1.732	8.37E-07	2.39E-05
RknMes02_003761	AT1G42430.1	0	cassava4.1_018440m	unknown protein; BEST Arabidopsis thaliana protein match i	1.728	4.78E-06	7.42E-05
RknMes02_020291	AT2G47600.1	4.10E-44	cassava4.1_007724m	magnesium/proton exchanger	1.727	4.38E-07	1.62E-05
RknMes02_036097	AT1G60010.1	0	cassava4.1_017235m	unknown protein; FUNCTIONS IN: molecular function unkn	1.727	2.36E-06	4.66E-05
RknMes02_027464	AT2G30150.1	4.00E-39	cassava4.1_007201m	UDP-Glycosyltransferase superfamily protein	1.718	1.27E-09	8.44E-07
RknMes02_051939	AT2G41905.1	6.00E-09	cassava4.1_022285m	AT2G41905.1	1.715	1.34E-08	2.55E-06
RknMes02_005227	AT4G36790.1	5.00E-21	cassava4.1_006777m	Major facilitator superfamily protein	1.712	4.45E-07	1.63E-05
RknMes02_004364	AT5G26820.1	3.00E-35	cassava4.1_004129m	iron-regulated protein 3	1.711	9.92E-07	2.65E-05
RknMes02_000538	AT5G20180.2	2.00E-16	cassava4.1_020514m	Ribosomal protein L36	1.711	4.89E-06	7.54E-05
RknMes02_048619	AT5G66460.1	0	cassava4.1_007999m	Glycosyl hydrolase superfamily protein	1.707	6.96E-06	9.55E-05
RknMes02_022019			cassava4.1_020536m		1.706	2.34E-06	4.63E-05
RknMes02_017763			cassava4.1_020325m		1.704	3.08E-06	5.50E-05
RknMes02_030347	AT5G02220.1	2.00E-15	cassava4.1_020849m	AT5G02220.1///unknown protein; Has 30201 Blast hits to 17	1.701	6.10E-07	1.94E-05
RknMes02_024133	AT5G08380.1	0	cassava4.1_011258m	alpha-galactosidase 1	1.699	8.03E-09	1.88E-06
RknMes02_015269	AT4G33220.1	0	cassava4.1_008249m	pectin methylesterase 44	1.688	3.93E-07	1.52E-05
RknMes02_020964	AT1G12780.1	0	cassava4.1_010908m	UDP-D-glucose/UDP-D-galactose 4-epimerase 1	1.682	5.38E-07	1.80E-05
RknMes02_051505	AT1G52540.1	0	cassava4.1_021554m	Protein kinase superfamily protein	1.678	4.01E-06	6.60E-05
RknMes02_038653	AT5G65280.1	2.00E-36	cassava4.1_008284m	GCR2-like 1	1.675	5.00E-07	1.72E-05
RknMes02_058899	AT1G02400.1	0	cassava4.1_033960m	gibberellin 2-oxidase 6	1.674	8.04E-07	2.33E-05
RknMes02_050209	AT1G80920.1	0	cassava4.1_017918m	Chaperone DnaJ-domain superfamily protein	1.674	2.96E-07	1.30E-05
RknMes02_046476			cassava4.1_019490m		1.664	7.85E-07	2.30E-05
RknMes02_031133	AT5G56840.1	0	cassava4.1_014027m	myb-like transcription factor family protein	1.662	9.09E-08	7.01E-06
RknMes02_003650	AT3G08730.1	3.00E-25	cassava4.1_006705m	protein-serine kinase 1	1.658	2.72E-07	1.24E-05
RknMes02_029029	AT3G11410.1	0	cassava4.1_008162m	protein phosphatase 2CA	1.657	6.73E-06	9.34E-05
RknMes02_058439	AT1G65920.1	0	cassava4.1_033184m	Regulator of chromosome condensation (RCC1) family with F	1.653	5.82E-06	8.45E-05
RknMes02_021080	AT3G23150.1	0	cassava4.1_002152m	ethylene response sensor 2///"Signal transduction histidine ki	1.652	2.98E-06	5.37E-05
RknMes02_029205	AT5G03230.1	1.00E-33		Protein of unknown function, DUF584	1.651	3.34E-08	4.16E-06
RknMes02_022193	AT1G67810.1	1.00E-23	cassava4.1_014023m	sulfur E2	1.650	2.00E-07	1.05E-05
RknMes02_032047			cassava4.1_015111m		1.648	2.23E-06	4.49E-05

RknMes02_010395			cassava4.1_001592m		1.647	3.67E-07	1.46E-05
RknMes02_041405	AT3G05890.1	4.00E-20		Low temperature and salt responsive protein family	1.645	2.06E-07	1.07E-05
RknMes02_031583	AT5G22920.1	0	cassava4.1_013514m	CHY-type/CTCHY-type/RING-type Zinc finger protein	1.644	5.06E-06	7.71E-05
RknMes02_041887			cassava4.1_019298m		1.643	3.96E-07	1.52E-05
RknMes02_028681	AT5G24530.1	0	cassava4.1_018326m	2-oxoglutarate (2OG) and Fe(II)-dependent oxygenase superfa	1.640	7.01E-06	9.60E-05
RknMes02_046403					1.640	1.39E-06	3.31E-05
RknMes02_047156			cassava4.1_029851m		1.621	1.32E-06	3.20E-05
RknMes02_013456	AT5G26820.1	3.00E-29	cassava4.1_004129m	iron-regulated protein 3	1.620	2.93E-06	5.31E-05
RknMes02_004216	AT1G77380.1	0	cassava4.1_006405m	amino acid permease 3	1.614	2.77E-06	5.12E-05
RknMes02_032101	AT3G06500.1	4.00E-19	cassava4.1_002913m	Plant neutral invertase family protein	1.613	5.59E-09	1.60E-06
RknMes02_010876	AT3G48530.1	9.00E-13	cassava4.1_010004m	SNF1-related protein kinase regulatory subunit gamma 1	1.613	1.59E-07	9.28E-06
RknMes02_010910			cassava4.1_012985m		1.612	6.77E-08	5.96E-06
RknMes02_005380	AT3G20660.1	1.00E-11	cassava4.1_031755m	organic cation/carnitine transporter4	1.605	1.01E-06	2.68E-05
RknMes02_055219	AT3G01600.1	0	cassava4.1_027882m	NAC (No Apical Meristem) domain transcriptional regulator	1.604	1.16E-08	2.33E-06
RknMes02_036667	AT5G21940.1	4.00E-39	cassava4.1_013924m	unknown protein; BEST Arabidopsis thaliana protein match i	1.603	8.48E-08	6.73E-06
RknMes02_026298	AT1G07040.1	1.00E-29	cassava4.1_022494m	unknown protein; INVOLVED IN: biological process unknov	1.597	3.46E-07	1.42E-05
RknMes02_003207	AT3G23150.1	6.00E-22	cassava4.1_002152m	ethylene response sensor 2///"Signal transduction histidine ki	1.590	2.76E-06	5.12E-05
RknMes02_014958	AT1G55000.3	1.00E-13	cassava4.1_014166m	peptidoglycan-binding LysM domain-containing protein	1.589	3.62E-06	6.15E-05
RknMes02_034166	AT1G14740.1	0	cassava4.1_001896m	Protein of unknown function (DUF1423)	1.589	1.67E-07	9.53E-06
RknMes02_058065	AT4G37420.1	0	cassava4.1_032582m	Domain of unknown function (DUF23)	1.583	9.41E-07	2.57E-05
RknMes02_027912	AT3G05500.1	0	cassava4.1_014920m	Rubber elongation factor protein (REF)	1.581	2.66E-07	1.23E-05
RknMes02_025601					1.571	6.81E-06	9.42E-05
RknMes02_040846	AT4G10270.1	3.00E-07		Wound-responsive family protein///Unknown	1.569	1.87E-06	4.00E-05
RknMes02_033342	AT4G30360.1	0	cassava4.1_009806m	cyclic nucleotide-gated channel 17///cyclic nucleotide-gated cl	1.568	6.45E-07	2.01E-05
RknMes02_009534	AT1G07870.2	0	cassava4.1_008779m	Protein kinase superfamily protein	1.568	8.27E-07	2.37E-05
RknMes02_012531	AT5G52510.1	8.00E-06	cassava4.1_003853m	Unknown///SCARECROW-like 8	1.566	3.84E-07	1.50E-05
RknMes02_011731			cassava4.1_005450m		1.564	8.66E-07	2.44E-05
RknMes02_008222	AT3G20300.1	0	cassava4.1_008956m	Protein of unknown function (DUF3537)	1.554	2.47E-10	3.87E-07
RknMes02_024633					1.551	3.26E-06	5.72E-05
RknMes02_003968	AT2G39720.1	2.00E-40	cassava4.1_023055m	zinc finger (C3HC4-type RING finger) family protein///RING	1.548	1.41E-09	8.96E-07
RknMes02_012490	AT2G19380.1	1.00E-09	cassava4.1_012985m	RNA recognition motif (RRM)-containing protein	1.548	3.64E-08	4.33E-06
RknMes02_042235			cassava4.1_013018m		1.546	2.32E-06	4.61E-05
RknMes02_032133	AT1G67300.2	7.00E-34	cassava4.1_008245m	Major facilitator superfamily protein	1.543	1.02E-09	8.07E-07
RknMes02_021018					1.539	1.61E-07	9.33E-06
RknMes02_033481	AT2G38000.1	0	cassava4.1_008257m	chaperone protein dnaJ-related	1.538	2.77E-08	3.73E-06
RknMes02_055311	AT5G54370.1	3.00E-39	cassava4.1_028042m	Late embryogenesis abundant (LEA) protein-related	1.534	8.84E-07	2.48E-05
RknMes02_012035	AT3G14050.1	6.00E-31	cassava4.1_002395m	RELA/SPOT homolog 2	1.531	1.32E-07	8.50E-06
RknMes02_034114	AT5G26667.3	7.00E-27	cassava4.1_016091m	P-loop containing nucleoside triphosphate hydrolases superfa	1.525	2.46E-07	1.18E-05
RknMes02_015555	AT3G13110.1	4.00E-28	cassava4.1_034339m	serine acetyltransferase 2;2	1.525	9.83E-09	2.13E-06
RknMes02_048737	AT2G39720.1	0	cassava4.1_009150m	zinc finger (C3HC4-type RING finger) family protein///RING	1.524	7.33E-10	6.67E-07
RknMes02_035616	AT2G28680.1	0	cassava4.1_010513m	RmlC-like cupins superfamily protein	1.519	3.78E-08	4.44E-06
RknMes02_015768	AT3G21700.3	2.00E-14	cassava4.1_015859m	Ras-related small GTP-binding family protein	1.518	1.00E-08	2.13E-06
RknMes02_008232	AT5G23240.1	9.00E-38	cassava4.1_006681m	DNAJ heat shock N-terminal domain-containing protein	1.516	7.07E-06	9.66E-05
RknMes02_026194			cassava4.1_009647m		1.512	2.84E-07	1.27E-05
RknMes02_036257	AT5G23240.1	3.00E-29	cassava4.1_006681m	DNAJ heat shock N-terminal domain-containing protein	1.510	5.15E-07	1.75E-05
RknMes02_021005	AT2G46270.2	1.00E-38	cassava4.1_008360m	G-box binding factor 3	1.510	1.57E-06	3.56E-05
RknMes02_058987	AT1G28310.2	4.00E-32	cassava4.1_034118m	Dof-type zinc finger DNA-binding family protein	1.509	4.96E-07	1.72E-05
RknMes02_049551	AT4G33000.2	0	cassava4.1_014701m	calcineurin B-like protein 10	1.509	4.95E-07	1.72E-05
RknMes02_008336	AT5G42810.1	8.00E-23	cassava4.1_007401m	inositol-pentakisphosphate 2-kinase 1	1.497	6.33E-06	8.93E-05
RknMes02_034689	AT3G23920.1	1.00E-34	cassava4.1_004325m	beta-amylase 1	1.495	7.21E-06	9.79E-05
RknMes02_052026	AT3G57230.1	6.00E-28	cassava4.1_022444m	AGAMOUS-like 16	1.489	2.81E-06	5.17E-05
RknMes02_049206	AT4G38660.1	0	cassava4.1_012600m	Pathogenesis-related thaumatin superfamily protein	1.489	2.14E-06	4.36E-05
RknMes02_027635					1.486	4.54E-08	4.85E-06
RknMes02_001354	AT5G52510.1	0	cassava4.1_003853m	Unknown///SCARECROW-like 8	1.477	3.10E-07	1.34E-05
RknMes02_025011	AT1G23870.1	8.00E-25	cassava4.1_001517m	trehalose-phosphatase/synthase 9	1.477	1.82E-07	9.95E-06
RknMes02_053203	AT2G22590.1	0	cassava4.1_024465m	UDP-Glycosyltransferase superfamily protein	1.471	8.65E-07	2.44E-05
RknMes02_054995	AT5G19790.1	3.00E-24	cassava4.1_027506m	related to AP2 11	1.469	5.84E-06	8.46E-05
RknMes02_056242	AT2G04240.2	0	cassava4.1_029590m	Unknown///RING/U-box superfamily protein///brassinosteroid	1.467	1.74E-07	9.78E-06
RknMes02_014407	AT2G28680.1	9.00E-15	cassava4.1_010513m	RmlC-like cupins superfamily protein	1.466	9.08E-08	7.01E-06
RknMes02_053395	AT5G05840.1	0	cassava4.1_024773m	Protein of unknown function (DUF620)	1.465	2.58E-06	4.90E-05
RknMes02_035747	AT4G21920.1	4.00E-12	cassava4.1_019677m	unknown protein; FUNCTIONS IN: molecular function unkn	1.464	5.97E-06	8.60E-05
RknMes02_038656			cassava4.1_012985m		1.463	2.64E-07	1.23E-05
RknMes02_049514	AT5G02230.2	0	cassava4.1_014451m	Haloacid dehalogenase-like hydrolase (HAD) superfamily pro	1.462	1.73E-06	3.81E-05
RknMes02_025204	AT1G09960.1	4.00E-33	cassava4.1_007925m	Unknown///sucrose transporter 4	1.457	6.83E-06	9.43E-05
RknMes02_037016	AT3G26300.1	0	cassava4.1_005635m	cytochrome P450, family 71, subfamily B, polypeptide 34///"c	1.455	2.30E-06	4.58E-05
RknMes02_041924	AT1G74520.1	6.00E-09	cassava4.1_020745m	HVA22 homologue A	1.455	4.04E-07	1.54E-05
RknMes02_026131	AT5G64170.2	2.00E-33	cassava4.1_006345m	dentin sialophosphoprotein-related	1.450	1.69E-07	9.54E-06
RknMes02_059045	AT2G41430.5	3.00E-36	cassava4.1_034338m	dehydration-induced protein (ERD15)///Protein containing PA	1.447	3.23E-07	1.37E-05
RknMes02_013408	AT1G08250.1	2.00E-37	cassava4.1_034329m	arogenate dehydratase 6	1.446	1.76E-06	3.85E-05
RknMes02_051345	AT4G05120.1	0	cassava4.1_021308m	Major facilitator superfamily protein	1.445	5.92E-06	8.55E-05
RknMes02_000847	AT1G54130.1	0	cassava4.1_002395m	RELA/SPOT homolog 3	1.437	3.41E-07	1.41E-05
RknMes02_029813			cassava4.1_013018m		1.436	1.06E-06	2.78E-05
RknMes02_010488	AT1G22540.1	9.00E-08	cassava4.1_004502m	Unknown///Major facilitator superfamily protein	1.435	1.11E-08	2.27E-06
RknMes02_003886	AT1G22540.1	4.00E-18	cassava4.1_004502m	Unknown///Major facilitator superfamily protein	1.425	1.59E-07	9.28E-06
RknMes02_039445	AT2G41380.1	0	cassava4.1_014142m	S-adenosyl-L-methionine-dependent methyltransferases super	1.420	3.28E-08	4.13E-06
RknMes02_009353	AT2G38760.1	4.00E-28	cassava4.1_012075m	annexin 3	1.413	2.12E-07	1.08E-05
RknMes02_031570	AT2G28680.1	0	cassava4.1_010513m	RmlC-like cupins superfamily protein	1.412	7.89E-08	6.42E-06
RknMes02_052138	AT1G06620.1	0	cassava4.1_022646m	2-oxoglutarate (2OG) and Fe(II)-dependent oxygenase superfa	1.410	6.92E-09	1.78E-06
RknMes02_052833	AT1G14870.1	1.40E-45	cassava4.1_023829m	PLANT CADMIUM RESISTANCE 2	1.410	7.66E-07	2.27E-05
RknMes02_048252	AT2G46500.2	0	cassava4.1_004287m	phosphoinositide 4-kinase gamma 4	1.409	3.05E-06	5.46E-05
RknMes02_005464	AT4G19200.1	7.00E-23	cassava4.1_009199m	Unknown///Class I glutamine amidotransferase-like superfam	1.407	2.29E-07	1.13E-05
RknMes02_027145	AT1G09140.2	0	cassava4.1_012620m	SERINE-ARGININE PROTEIN 30	1.405	1.81E-07	9.93E-06
RknMes02_049384	AT5G59480.1	0	cassava4.1_013675m	Haloacid dehalogenase-like hydrolase (HAD) superfamily pro	1.403	3.48E-06	5.99E-05
RknMes02_037703	AT2G45400.1	3.00E-33	cassava4.1_014136m	NAD(P)-binding Rossmann-fold superfamily protein	1.402	1.42E-06	3.35E-05
RknMes02_049251	AT4G35070.2	0	cassava4.1_012855m	SBP (S-ribonuclease binding protein) family protein	1.402	1.20E-06	3.00E-05
RknMes02_057524	AT1G65980.1	0	cassava4.1_031724m	thioredoxin-dependent peroxidase 1	1.400	1.47E-07	9.03E-06
RknMes02_029874	AT5G16010.1	0	cassava4.1_014084m	3-oxo-5-alpha-steroid 4-dehydrogenase family protein	1.394	1.42E-06	3.35E-05
RknMes02_054534	AT3G12160.1	0	cassava4.1_026712m	RAB GTPase homolog A4D	1.390	5.09E-07	1.74E-05
RknMes02_022554	AT1G74520.1	7.00E-22	cassava4.1_020745m	HVA22 homologue A	1.390	3.61E-08	4.32E-06
RknMes02_050873	AT5G65207.1	2.00E-07	cassava4.1_020474m	AT5G65207.1	1.390	7.26E-08	6.18E-06
RknMes02_014916			cassava4.1_011689m		1.386	6.13E-07	1.94E-05
RknMes02_018927			cassava4.1_012035m		1.385	1.08E-06	2.80E-05
RknMes02_049641	AT5G64700.1	0	cassava4.1_015199m	nodulin MtN21 /EamA-like transporter family protein	1.385	6.31E-06	8.92E-05
RknMes02_037752	AT4G29010.1	1.00E-29	cassava4.1_006963m	Unknown///Enoyl-CoA hydratase/isomerase family	1.383	3.09E-07	1.34E-05
RknMes02_015829					1.380	9.79E-07	2.63E-05
RknMes02_052796	AT3G12490.2	0	cassava4.1_023762m	Unknown///cystatin B	1.379	5.99E-08	5.57E-06
RknMes02_025610	AT2G26540.1	0	cassava4.1_031984m	uroporphyrinogen-III synthase family protein	1.379	1.16E-07	7.87E-06
RknMes02_030630	AT2G31560.2	0	cassava4.1_016742m	Protein of unknown function (DUF1685)	1.377	5.10E-06	7.75E-05
RknMes02_054073	AT5G20710.1	0	cassava4.1_025948m	beta-galactosidase 15///beta-galactosidase 7	1.374	1.20E-07	8.01E-06
RknMes02_036718	AT1G27320.1	4.00E-23	cassava4.1_000780m	histidine kinase 3	1.371	2.28E-06	4.56E-05
RknMes02_039442	AT5G55560.1	0	cassava4.1_012772m	Protein kinase superfamily protein	1.371	2.41E-08	3.43E-06
RknMes02_025417	AT3G08720.2	0	cassava4.1_006705m	serine/threonine protein kinase 2	1.369	7.03E-06	9.62E-05
RknMes02_033786	AT5G21940.1	1.00E-35	cassava4.1_013741m	unknown protein; BEST Arabidopsis thaliana protein match i	1.368	1.09E-06	2.82E-05
RknMes02_032390	AT3G21760.1	0	cassava4.1_031478m	UDP-Glycosyltransferase superfamily protein///UDP-glucosyl	1.366	1.67E-06	3.72E-05
RknMes02_050168	AT4G38060.2	3.00E-14	cassava4.1_017718m	unknown protein; BEST Arabidopsis thaliana protein match i	1.365	6.96E-07	2.13E-05
RknMes02_009054	AT4G35590.1	4.00E-31	cassava4.1_024901m	NIN like protein 7///RWP-RK domain-containing protein	1.362	1.94E-08	3.14E-06
RknMes02_039401	AT3G47640.3	6.00E-11	cassava4.1_015506m	basic helix-loop-helix (bHLH) DNA-binding superfamily prot	1.359	3.36E-06	5.85E-05
RknMes02_033605	AT5G10650.2	8.00E-18	cassava4.1_004931m	RING/U-box superfamily protein	1.359	7.61E-08	6.26E-06
RknMes02_013312	AT1G31280.1	1.00E-13	cassava4.1_000920m	Argonaute family protein	1.358	1.38E-07	8.73E-06
RknMes02_001315	AT4G29010.1	0	cassava4.1_006963m	Unknown///Enoyl-CoA hydratase/isomerase family	1.357	1.53E-07	9.15E-06
RknMes02_013203	AT2G37770.2	6.00E-16	cassava4.1_016997m	NAD(P)-linked oxidoreductase superfamily protein	1.353	1.20E-06	3.00E-05
RknMes02_024410	AT5G25350.1	0	cassava4.1_003247m	EIN3-binding F box protein 2	1.352	8.51E-07	2.42E-05
RknMes02_003081	AT3G08720.2	3.00E-27	cassava4.1_006705m	serine/threonine protein kinase 2	1.350	6.03E-06	8.65E-05
RknMes02_059057	AT3G60220.1	2.00E-16	cassava4.1_034393m	RING/U-box superfamily protein///Unknown	1.346	7.88E-07	2.31E-05
RknMes02_037299	AT5G59520.1	1.00E-21	cassava4.1_011392m	ZRT/IRT-like protein 2	1.346	3.21E-09	1.26E-06
RknMes02_006744	AT3G48990.1	7.00E-42	cassava4.1_005514m	Unknown///AMP-dependent synthetase and ligase family prot	1.345	4.81E-06	7.44E-05
RknMes02_000130	AT3G60390.1	2.00E-37	cassava4.1_023832m	homeobox-leucine zipper protein 3///homeobox-leucine zippe	1.345	4.10E-08	4.57E-06
RknMes02_054305	AT3G57120.1	0	cassava4.1_026334m	Protein kinase superfamily protein///Unknown	1.340	3.08E-07	1.33E-05
RknMes02_033587	AT5G26731.1	3.00E-17	cassava4.1_033664m	unknown protein; Has 30201 Blast hits to 17322 proteins in 7	1.339	1.85E-07	1.01E-05
RknMes02_006016			cassava4.1_015713m		1.338	1.47E-07	9.03E-06
RknMes02_014995	AT5G04040.1	4.00E-22	cassava4.1_020983m	Patatin-like phospholipase family protein///Unknown	1.338	2.92E-06	5.30E-05
RknMes02_053251	AT2G41905.1	4.00E-08	cassava4.1_024547m	AT2G41905.1	1.329	4.18E-08	4.63E-06
RknMes02_009959			cassava4.1_028001m		1.328	1.12E-07	7.82E-06
RknMes02_025278	AT2G46270.1	4.00E-37	cassava4.1_008360m	G-box binding factor 3	1.327	2.12E-06	4.34E-05
RknMes02_008572	AT4G15417.1	3.00E-37	cassava4.1_031224m	RNAse II-like 1	1.326	7.85E-08	6.40E-06
RknMes02_007607	AT4G25620.1	5.00E-06	cassava4.1_006120m	Unknown///hydroxyproline-rich glycoprotein family protein	1.321	7.00E-08	6.05E-06
RknMes02_049041	AT2G42040.1	2.00E-29	cassava4.1_011424m	AT2G42040.1	1.320	5.45E-06	8.09E-05

RknMes02_052892			cassava4.1_023929m		1.318	1.80E-06	3.91E-05
RknMes02_028547	AT2G32560.1	1.00E-35	cassava4.1_011457m	F-box family protein	1.317	1.92E-07	1.03E-05
RknMes02_034252	AT5G24870.2	0	cassava4.1_004931m	RING/U-box superfamily protein	1.313	1.11E-09	8.36E-07
RknMes02_044719					1.312	1.05E-07	7.53E-06
RknMes02_044359	AT5G46730.1	4.00E-07		Unknown	1.311	9.04E-10	7.43E-07
RknMes02_009918	AT4G08620.1	1.00E-09		sulphate transporter 1;1	1.311	6.31E-06	8.92E-05
RknMes02_007092			cassava4.1_000175m		1.309	1.12E-06	2.87E-05
RknMes02_030517	AT1G51140.1	3.00E-23	cassava4.1_008877m	basic helix-loop-helix (bHLH) DNA-binding superfamily prot	1.308	2.36E-07	1.15E-05
RknMes02_032772	AT1G53035.1	0	cassava4.1_018451m	AT1G53035.1///unknown protein; FUNCTIONS IN: molecula	1.304	1.90E-07	1.02E-05
RknMes02_010067	AT4G29130.1	2.00E-06		Unknown///hexokinase 1///hexokinase 2	1.304	5.45E-06	8.09E-05
RknMes02_023982	AT3G06760.1	2.00E-19	cassava4.1_016736m	Drought-responsive family protein	1.301	1.45E-06	3.38E-05
RknMes02_007470	AT5G60800.2	1.00E-22	cassava4.1_033242m	Unknown///Heavy metal transport/detoxification superfamily	1.301	4.12E-07	1.56E-05
RknMes02_012445	AT2G36320.1	5.00E-25	cassava4.1_018151m	A20/AN1-like zinc finger family protein	1.301	5.15E-07	1.75E-05
RknMes02_025637	AT4G35750.1	0	cassava4.1_016359m	SEC14 cytosolic factor family protein / phosphoglyceride tran	1.301	2.72E-08	3.70E-06
RknMes02_030504	AT1G01720.1	0	cassava4.1_013132m	NAC (No Apical Meristem) domain transcriptional regulator	1.300	6.62E-06	9.23E-05
RknMes02_035621	AT3G52105.1	4.00E-16	cassava4.1_020764m	AT3G52105.1///unknown protein; FUNCTIONS IN: molecula	1.299	2.13E-06	4.36E-05
RknMes02_032932	AT4G29010.1	0	cassava4.1_029596m	Unknown///Enoyl-CoA hydratase/isomerase family	1.297	2.29E-07	1.13E-05
RknMes02_008946	AT1G26580.1	2.00E-17	cassava4.1_005827m	FUNCTIONS IN: molecular function unknown; INVOLVED	1.295	4.41E-07	1.62E-05
RknMes02_038088	AT5G07290.1	0	cassava4.1_001535m	MEI2-like 4	1.291	1.18E-08	2.37E-06
RknMes02_039208	AT1G15520.1	0	cassava4.1_000251m	pleiotropic drug resistance 3///pleiotropic drug resistance 12	1.287	9.48E-07	2.58E-05
RknMes02_051840	AT3G09280.1	1.00E-12	cassava4.1_022123m	AT3G09280.1///Unknown	1.287	9.67E-07	2.61E-05
RknMes02_057298	AT1G45616.1	0	cassava4.1_031360m	receptor like protein 7///receptor like protein 6///disease resis	1.286	2.92E-06	5.30E-05
RknMes02_051417	AT1G19250.1	0	cassava4.1_021420m	flavin-dependent monooxygenase 1	1.286	4.86E-09	1.53E-06
RknMes02_001339	AT1G32700.2	2.00E-11	cassava4.1_015881m	Unknown///PLATZ transcription factor family protein	1.284	2.65E-07	1.23E-05
RknMes02_036682	AT1G33420.1	5.00E-19	cassava4.1_002981m	RING/FYVE/PHD zinc finger superfamily protein	1.284	3.98E-09	1.42E-06
RknMes02_038617	AT4G19200.1	5.00E-14		Unknown///Class I glutamine amidotransferase-like superfam	1.284	7.37E-07	2.22E-05
RknMes02_054030	AT4G03510.2	0	cassava4.1_025866m	RING membrane-anchor 1	1.282	4.89E-08	4.98E-06
RknMes02_049452	AT4G03510.2	0	cassava4.1_014098m	RING membrane-anchor 1	1.280	7.87E-09	1.87E-06
RknMes02_038894	AT1G77280.1	0	cassava4.1_002273m	Protein kinase protein with adenine nucleotide alpha hydrolas	1.277	4.47E-06	7.07E-05
RknMes02_006780	AT1G02640.1	0	cassava4.1_002149m	beta-xylosidase 2///Glycosyl hydrolase family protein	1.277	3.20E-07	1.37E-05
RknMes02_007981	AT3G05500.1	6.00E-23	cassava4.1_014920m	Rubber elongation factor protein (REF)	1.276	1.96E-07	1.04E-05
RknMes02_037154	AT1G61260.1	2.00E-20	cassava4.1_011422m	Protein of unknown function (DUF761)	1.273	5.75E-07	1.87E-05
RknMes02_033442	AT2G43060.1	5.00E-18	cassava4.1_034432m	ILI1 binding bHLH 1	1.270	2.03E-07	1.06E-05
RknMes02_032606					1.267	4.57E-07	1.65E-05
RknMes02_056656	AT5G54370.1	0	cassava4.1_030303m	Late embryogenesis abundant (LEA) protein-related	1.267	8.91E-07	2.49E-05
RknMes02_006189	AT4G28300.2	5.00E-08	cassava4.1_011905m	Protein of unknown function (DUF1421)	1.265	5.28E-06	7.92E-05
RknMes02_008012	AT1G07710.1	0	cassava4.1_005121m	Ankyrin repeat family protein	1.264	1.56E-06	3.55E-05
RknMes02_010817	AT1G08510.1	2.00E-11	cassava4.1_008444m	fatty acyl-ACP thioesterases B	1.260	5.03E-08	5.05E-06
RknMes02_027394	AT5G65110.2	0	cassava4.1_002855m	acyl-CoA oxidase 2	1.259	5.32E-07	1.79E-05
RknMes02_045659					1.259	7.30E-06	9.88E-05
RknMes02_033706	AT1G33060.2	2.00E-25	cassava4.1_011070m	NAC 014	1.258	8.46E-07	2.41E-05
RknMes02_056665	AT5G20150.1	1.40E-45	cassava4.1_030318m	SPX domain gene 3///SPX domain gene 1	1.257	1.07E-06	2.78E-05
RknMes02_045014					1.253	5.80E-06	8.44E-05
RknMes02_012214	AT1G06290.1	4.00E-18	cassava4.1_002966m	acyl-CoA oxidase 3	1.252	7.21E-06	9.79E-05
RknMes02_026090	AT1G70330.1	0	cassava4.1_008204m	equilibrative nucleotide transporter 1	1.251	4.12E-06	6.71E-05
RknMes02_043051	AT3G57230.2	7.00E-26	cassava4.1_032220m	AGAMOUS-like 16	1.250	1.29E-06	3.16E-05
RknMes02_052907			cassava4.1_023951m		1.248	6.82E-06	9.43E-05
RknMes02_006939	AT5G60600.3	0	cassava4.1_002363m	4-hydroxy-3-methylbut-2-enyl diphosphate synthase	1.246	7.33E-08	6.19E-06
RknMes02_003582	AT4G18120.2	1.00E-08	cassava4.1_001549m	Unknown	1.245	2.81E-06	5.18E-05
RknMes02_012269	AT1G06460.1	2.00E-10	cassava4.1_015713m	alpha-crystallin domain 32.1	1.245	4.63E-06	7.23E-05
RknMes02_005209	AT2G39720.1	1.00E-08	cassava4.1_023055m	zinc finger (C3HC4-type RING finger) family protein///RING	1.242	1.69E-09	1.01E-06
RknMes02_013641	AT4G17840.1	2.00E-09	cassava4.1_009226m	FUNCTIONS IN: molecular function unknown; INVOLVED	1.241	1.86E-06	3.99E-05
RknMes02_044688	AT4G25680.1	4.00E-17	cassava4.1_013774m	PPPDE putative thiol peptidase family protein///AT5G52220.	1.240	2.56E-07	1.20E-05
RknMes02_049295	AT2G06025.1	0	cassava4.1_013144m	Acyl-CoA N-acyltransferases (NAT) superfamily protein	1.237	7.35E-09	1.81E-06
RknMes02_028012	AT1G06570.2	0	cassava4.1_007612m	phytoene desaturation 1	1.235	6.00E-07	1.91E-05
RknMes02_033495	AT1G70300.1	0	cassava4.1_002041m	K+ uptake permease 6	1.232	2.28E-07	1.13E-05
RknMes02_032229	AT1G07180.1	0	cassava4.1_008961m	alternative NAD(P)H dehydrogenase 2///alternative NAD(P)I	1.232	6.15E-06	8.79E-05
RknMes02_030656	AT3G15070.2	2.00E-40	cassava4.1_004952m	RING/U-box superfamily protein	1.230	4.66E-09	1.51E-06
RknMes02_013376			cassava4.1_006345m		1.229	8.40E-07	2.40E-05
RknMes02_037895	AT1G34220.2	8.00E-26	cassava4.1_005368m	Regulator of Vps4 activity in the MVB pathway protein	1.228	1.35E-06	3.24E-05
RknMes02_052064	AT1G65570.1	0	cassava4.1_022514m	Pectin lyase-like superfamily protein	1.226	5.41E-07	1.80E-05
RknMes02_034218	AT2G28470.2	0	cassava4.1_001885m	beta-galactosidase 8	1.225	6.23E-06	8.85E-05
RknMes02_013846	AT2G02870.3	2.00E-07	cassava4.1_008308m	Galactose oxidase/kelch repeat superfamily protein	1.224	3.28E-07	1.38E-05
RknMes02_015028			cassava4.1_032424m		1.223	4.60E-07	1.65E-05
RknMes02_049203	AT3G60390.1	0	cassava4.1_012590m	homeobox-leucine zipper protein 3///homeobox-leucine zipper	1.222	1.19E-09	8.41E-07
RknMes02_037563	AT2G17710.1	2.00E-40	cassava4.1_017894m	unknown protein; Has 39 Blast hits to 39 proteins in 14 speci	1.220	1.22E-06	3.02E-05
RknMes02_012197			cassava4.1_009431m		1.220	2.43E-08	3.44E-06
RknMes02_023533	AT3G26100.2	2.00E-13	cassava4.1_005225m	Regulator of chromosome condensation (RCC1) family protei	1.219	2.04E-06	4.23E-05
RknMes02_001523	AT3G52590.1	8.00E-41		ubiquitin extension protein 1	1.218	5.11E-06	7.76E-05
RknMes02_014915	AT4G31450.1	2.00E-16	cassava4.1_004931m	RING/U-box superfamily protein	1.217	5.22E-08	5.17E-06
RknMes02_036203	AT2G47910.1	0	cassava4.1_014553m	chlororespiratory reduction 6	1.214	1.10E-08	2.27E-06
RknMes02_011950	AT5G65110.2	2.00E-22	cassava4.1_002855m	acyl-CoA oxidase 2	1.211	1.62E-07	9.36E-06
RknMes02_031852	AT2G30620.1	2.80E-45	cassava4.1_015823m	winged-helix DNA-binding transcription factor family protein	1.210	3.46E-07	1.42E-05
RknMes02_015249	AT2G01450.4	2.00E-18	cassava4.1_006340m	MAP kinase 17	1.200	3.21E-06	5.66E-05
RknMes02_052578	AT1G52720.1	3.00E-23	cassava4.1_023383m	AT1G52720.1///unknown protein; BEST Arabidopsis thaliana	1.197	4.23E-06	6.83E-05
RknMes02_020100			cassava4.1_019828m		1.196	3.51E-07	1.43E-05
RknMes02_052131			cassava4.1_022635m		1.196	4.77E-08	4.94E-06
RknMes02_005496	AT5G05140.1	6.00E-14	cassava4.1_006748m	Transcription elongation factor (TFIIS) family protein	1.193	6.69E-07	2.07E-05
RknMes02_057031	AT5G01260.2	1.00E-25	cassava4.1_030934m	Carbohydrate-binding-like fold	1.191	2.94E-06	5.33E-05
RknMes02_029903	AT1G13360.1	1.00E-24	cassava4.1_017136m	unknown protein; BEST Arabidopsis thaliana protein match i	1.190	6.75E-06	9.36E-05
RknMes02_019648	AT1G30070.2	1.00E-08	cassava4.1_013041m	Unknown///SGS domain-containing protein	1.188	4.84E-06	7.48E-05
RknMes02_014960	AT4G28400.1	9.00E-08	cassava4.1_013399m	Protein phosphatase 2C family protein	1.187	3.03E-06	5.43E-05
RknMes02_009979	AT3G21700.3	1.00E-38	cassava4.1_026431m	Ras-related small GTP-binding family protein	1.186	1.93E-06	4.08E-05
RknMes02_001771	AT3G48990.1	0	cassava4.1_005514m	Unknown///AMP-dependent synthetase and ligase family prot	1.185	5.13E-06	7.78E-05
RknMes02_032086	AT4G13030.1	1.00E-37	cassava4.1_008236m	P-loop containing nucleoside triphosphate hydrolases superfa	1.185	2.26E-07	1.12E-05
RknMes02_026725	AT4G29130.1	0	cassava4.1_006138m	Unknown///hexokinase 1///hexokinase 2	1.184	3.53E-07	1.43E-05
RknMes02_022008	AT4G19200.1	0		Unknown///Class I glutamine amidotransferase-like superfam	1.182	1.39E-07	8.73E-06
RknMes02_031679	AT1G71880.1	5.00E-19	cassava4.1_004111m	sucrose-proton symporter 2	1.182	1.55E-06	3.53E-05
RknMes02_010347	AT4G35780.1	3.00E-25	cassava4.1_026472m	ACT-like protein tyrosine kinase family protein	1.180	2.28E-06	4.57E-05
RknMes02_050323	AT5G24800.1	1.00E-37	cassava4.1_018467m	basic leucine zipper 9	1.179	7.23E-07	2.18E-05
RknMes02_025737	AT5G20620.1	0	cassava4.1_016182m	polyubiquitin 3///polyubiquitin 10///cassava4.1_030654m	1.179	4.17E-06	6.77E-05
RknMes02_024920	AT5G39000.1	6.00E-34	cassava4.1_002842m	Unknown///Malectin/receptor-like protein kinase family prote	1.179	2.30E-06	4.58E-05
RknMes02_024117	AT1G27950.1	2.00E-40	cassava4.1_016986m	glycosylphosphatidylinositol-anchored lipid protein transfer 1	1.179	1.47E-07	9.03E-06
RknMes02_024720	AT4G25620.1	2.00E-40	cassava4.1_006603m	Unknown///hydroxyproline-rich glycoprotein family protein	1.178	1.36E-09	8.82E-07
RknMes02_043801					1.177	4.32E-08	4.73E-06
RknMes02_028645	AT5G62740.1	0	cassava4.1_013311m	SPFH/Band 7/PHB domain-containing membrane-associated	1.176	5.35E-06	7.98E-05
RknMes02_013899	AT1G43630.1	5.00E-10	cassava4.1_009431m	Protein of unknown function (DUF793)	1.175	3.98E-09	1.42E-06
RknMes02_039967	AT5G57710.1	1.00E-41	cassava4.1_000717m	Double Clp-N motif-containing P-loop nucleoside triphosphat	1.174	1.93E-06	4.09E-05
RknMes02_002000	AT1G79380.1	2.00E-43	cassava4.1_016026m	Ca(2)-dependent phospholipid-binding protein (Copine) famil	1.174	2.81E-07	1.26E-05
RknMes02_042045					1.173	1.97E-06	4.14E-05
RknMes02_004444			cassava4.1_006345m		1.172	9.05E-07	2.50E-05
RknMes02_047942	AT3G13380.1	0	cassava4.1_000618m	BRI1-like 3///BRI1 like	1.170	9.15E-08	7.03E-06
RknMes02_035812	AT1G17080.1	1.00E-17	cassava4.1_027425m	Ribosomal protein L18ae family	1.168	6.28E-09	1.68E-06
RknMes02_013062			cassava4.1_007184m		1.167	5.15E-08	5.14E-06
RknMes02_055301	AT4G27140.1	3.00E-07	cassava4.1_028018m	seed storage albumin 5	1.167	6.53E-07	2.03E-05
RknMes02_034314	AT4G17900.1	0	cassava4.1_015721m	PLATZ transcription factor family protein	1.167	5.94E-06	8.56E-05
RknMes02_040320	AT2G47600.1	1.00E-09	cassava4.1_005115m	magnesium/proton exchanger	1.166	2.15E-07	1.09E-05
RknMes02_057309	AT5G67140.1	0	cassava4.1_031373m	F-box/RNI-like superfamily protein	1.164	3.77E-06	6.33E-05
RknMes02_008717	AT3G10030.1	3.00E-18	cassava4.1_006212m	aspartate/glutamate/uridylate kinase family protein	1.163	7.95E-07	2.32E-05
RknMes02_030782	AT5G54585.1	6.00E-07	cassava4.1_018734m	Unknown	1.163	1.97E-06	4.14E-05
RknMes02_003788	AT2G23970.1	9.00E-30	cassava4.1_023112m	Class I glutamine amidotransferase-like superfamily protein	1.162	3.68E-07	1.47E-05
RknMes02_028904	AT5G11650.1	0	cassava4.1_009674m	alpha/beta-Hydrolases superfamily protein	1.162	2.00E-06	4.16E-05
RknMes02_031128	AT4G14220.1	2.00E-44	cassava4.1_009239m	RING-H2 group F1A	1.160	4.19E-07	1.57E-05
RknMes02_000125					1.158	2.39E-07	1.16E-05
RknMes02_004760	AT1G70300.1	2.00E-24	cassava4.1_002041m	K+ uptake permease 6	1.151	1.77E-08	2.94E-06
RknMes02_030872	AT1G56145.2	0	cassava4.1_025524m	Leucine-rich repeat transmembrane protein kinase	1.150	4.27E-06	6.86E-05
RknMes02_000457	AT3G17250.1	3.00E-17	cassava4.1_009647m	Protein phosphatase 2C family protein	1.149	9.38E-07	2.56E-05
RknMes02_000611	AT4G09150.2	1.00E-26	cassava4.1_000492m	T-complex protein 11	1.147	6.72E-09	1.75E-06
RknMes02_000581	AT5G25110.1	3.00E-24	cassava4.1_029811m	CBL-interacting protein kinase 25///CBL-interacting protein k	1.147	1.43E-06	3.37E-05
RknMes02_058966	AT5G49330.1	0	cassava4.1_034078m	myb domain protein 111///myb domain protein 15	1.145	2.52E-06	4.83E-05
RknMes02_054366	AT3G21700.3	0	cassava4.1_026431m	Ras-related small GTP-binding family protein	1.144	7.58E-08	6.25E-06
RknMes02_034254	AT2G28930.3	0	cassava4.1_014580m	protein kinase 1B	1.140	5.79E-07	1.88E-05
RknMes02_007395	AT4G30350.1	6.00E-10	cassava4.1_000717m	Double Clp-N motif-containing P-loop nucleoside triphosphat	1.137	6.41E-07	2.01E-05
RknMes02_032921	AT4G28300.1	0	cassava4.1_006851m	Protein of unknown function (DUF1421)///Unknown	1.135	4.83E-07	1.69E-05
RknMes02_057685	AT2G26540.1	0	cassava4.1_031984m	uroporphyrinogen-III synthase family protein	1.135	6.26E-09	1.68E-06
RknMes02_057999	AT1G52820.1	0	cassava4.1_032469m	2-oxoglutarate (2OG) and Fe(II)-dependent oxygenase superfa	1.134	1.98E-06	4.15E-05
RknMes02_058115	AT3G07390.1	0	cassava4.1_032657m	auxin-responsive family protein///Auxin-responsive family pro	1.130	1.10E-06	2.84E-05

RknMes02_032950	AT4G25970.1	0	cassava4.1_022166m	phosphatidylserine decarboxylase 3	1.128	5.26E-06	7.90E-05
RknMes02_029459					1.120	4.36E-09	1.47E-06
RknMes02_006582	AT5G11650.1	0	cassava4.1_009674m	alpha/beta-Hydrolases superfamily protein	1.119	6.25E-06	8.87E-05
RknMes02_004126	AT1G29400.2	0	cassava4.1_002304m	MEI2-like protein 5	1.118	4.47E-07	1.63E-05
RknMes02_036491	AT4G25150.1	0	cassava4.1_013955m	HAD superfamily, subfamily IIIB acid phosphatase	1.117	1.33E-07	8.50E-06
RknMes02_014371	AT2G19860.1	3.00E-15	cassava4.1_006138m	hexokinase 1	1.116	2.40E-08	3.43E-06
RknMes02_031068	AT1G80380.4	0	cassava4.1_006274m	P-loop containing nucleoside triphosphate hydrolases superfa	1.116	2.99E-06	5.38E-05
RknMes02_016443	AT3G62770.3	0	cassava4.1_009874m	Transducin/WD40 repeat-like superfamily protein	1.116	1.36E-06	3.27E-05
RknMes02_030235	AT1G28520.2	6.00E-33	cassava4.1_030824m	vascular plant one zinc finger protein	1.115	3.23E-06	5.69E-05
RknMes02_038791	AT1G22710.1	8.00E-17	cassava4.1_007460m	sucrose-proton symporter 2	1.112	1.00E-10	2.44E-07
RknMes02_055845	AT3G51880.4	6.00E-16	cassava4.1_028951m	high mobility group B2///high mobility group B1	1.111	3.24E-07	1.37E-05
RknMes02_029211	AT5G51100.1	0	cassava4.1_013844m	Fe superoxide dismutase 2	1.111	6.53E-06	9.14E-05
RknMes02_057766	AT4G33800.1	1.00E-20	cassava4.1_032095m	AT4G33800.1///unknown protein; Has 30201 Blast hits to 17	1.111	6.19E-07	1.96E-05
RknMes02_027041	AT2G30360.1	0	cassava4.1_008014m	serine/threonine protein kinase 1///SOS3-interacting protein 4	1.110	3.97E-07	1.53E-05
RknMes02_022308					1.110	2.09E-06	4.30E-05
RknMes02_015864	AT4G35840.1	0	cassava4.1_015188m	RING/U-box superfamily protein	1.110	5.51E-07	1.82E-05
RknMes02_037060	AT1G19600.1	1.00E-11	cassava4.1_012166m	pfkB-like carbohydrate kinase family protein	1.109	1.87E-06	4.01E-05
RknMes02_004074	AT3G52800.1	2.00E-17	cassava4.1_018151m	A20/AN1-like zinc finger family protein	1.108	1.93E-09	1.08E-06
RknMes02_038544	AT1G34220.2	6.00E-06	cassava4.1_005454m	Regulator of Vps4 activity in the MVB pathway protein	1.106	4.89E-07	1.70E-05
RknMes02_039954	AT1G67980.2	0	cassava4.1_015209m	caffeoyl-CoA 3-O-methyltransferase	1.102	1.44E-06	3.38E-05
RknMes02_038465	AT4G16210.1	0	cassava4.1_014036m	enoyl-CoA hydratase/isomerase A	1.101	8.07E-08	6.48E-06
RknMes02_050408	AT5G21170.1	8.00E-29	cassava4.1_018890m	5\'-AMP-activated protein kinase beta-2 subunit protein///5'-A	1.097	2.13E-08	3.30E-06
RknMes02_013918	AT5G14880.1	5.00E-25	cassava4.1_002050m	Potassium transporter family protein	1.096	2.69E-07	1.23E-05
RknMes02_030839					1.095	5.82E-07	1.88E-05
RknMes02_033429	AT4G02600.2	0	cassava4.1_005640m	Seven transmembrane MLO family protein	1.094	2.91E-07	1.29E-05
RknMes02_043315					1.094	2.33E-06	4.62E-05
RknMes02_005407	AT4G29130.1	0	cassava4.1_006138m	Unknown///hexokinase 1///hexokinase 2	1.092	7.88E-07	2.31E-05
RknMes02_024552	AT4G05120.1	0	cassava4.1_008482m	Major facilitator superfamily protein	1.092	7.14E-09	1.80E-06
RknMes02_004493	AT3G27020.1	7.00E-37	cassava4.1_002751m	YELLOW STRIPE like 6	1.091	4.01E-06	6.60E-05
RknMes02_016573	AT3G02875.1	9.00E-34	cassava4.1_026678m	Peptidase M20/M25/M40 family protein	1.090	3.40E-06	5.90E-05
RknMes02_052275	AT5G01350.1	5.00E-13	cassava4.1_022869m	AT5G01350.1///unknown protein; FUNCTIONS IN: molecula	1.090	1.82E-08	3.01E-06
RknMes02_006048			cassava4.1_016663m		1.088	1.86E-08	3.04E-06
RknMes02_052114	AT3G14670.2	3.00E-06	cassava4.1_022594m	cassava4.1_022594m	1.087	1.41E-06	3.34E-05
RknMes02_030333	AT5G07290.1	0	cassava4.1_001002m	MEI2-like 4	1.087	5.68E-08	5.42E-06
RknMes02_032249	AT3G27020.1	0	cassava4.1_003082m	YELLOW STRIPE like 6	1.086	6.84E-07	2.11E-05
RknMes02_002886	AT4G09150.2	2.00E-12	cassava4.1_000492m	T-complex protein 11	1.084	3.62E-08	4.33E-06
RknMes02_016203			cassava4.1_018507m		1.084	1.71E-08	2.91E-06
RknMes02_033630	AT3G47640.3	1.40E-45	cassava4.1_015582m	basic helix-loop-helix (bHLH) DNA-binding superfamily prot	1.083	2.22E-08	3.36E-06
RknMes02_018552					1.083	3.36E-06	5.85E-05
RknMes02_027144	AT2G06530.1	0	cassava4.1_015677m	Unknown///SNF7 family protein	1.077	4.29E-07	1.60E-05
RknMes02_005973	AT1G06570.2	0	cassava4.1_007612m	phytoene desaturation 1	1.077	6.35E-08	5.74E-06
RknMes02_036778	AT5G28680.1	0	cassava4.1_002842m	Malectin/receptor-like protein kinase family protein	1.076	5.40E-06	8.04E-05
RknMes02_000149	AT5G64170.2	1.00E-33	cassava4.1_004946m	dentin sialophosphoprotein-related	1.075	1.66E-06	3.69E-05
RknMes02_028974	AT1G13700.1	0	cassava4.1_014028m	6-phosphogluconolactonase 1	1.074	1.88E-06	4.01E-05
RknMes02_008708	AT1G60420.1	1.00E-27	cassava4.1_004565m	DC1 domain-containing protein	1.073	2.51E-06	4.82E-05
RknMes02_035736	AT2G30620.1	0	cassava4.1_015193m	winged-helix DNA-binding transcription factor family protein	1.066	2.47E-06	4.78E-05
RknMes02_014364	AT1G70300.1	2.00E-40	cassava4.1_029286m	K+ uptake permease 6	1.066	1.34E-06	3.23E-05
RknMes02_000060			cassava4.1_004946m		1.066	1.09E-06	2.82E-05
RknMes02_001721	AT4G01370.1	2.00E-29	cassava4.1_013552m	MAP kinase 4	1.064	2.45E-07	1.17E-05
RknMes02_005244	AT1G22100.1	5.00E-32	cassava4.1_030933m	Inositol-pentakisphosphate 2-kinase family protein	1.063	2.19E-06	4.45E-05
RknMes02_000305	AT5G15950.2	2.00E-44	cassava4.1_010527m	Adenosylmethionine decarboxylase family protein	1.061	4.70E-06	7.32E-05
RknMes02_023063					1.060	5.23E-06	7.88E-05
RknMes02_031655	AT4G13020.5	6.00E-22	cassava4.1_007990m	Protein kinase superfamily protein	1.056	9.20E-11	2.44E-07
RknMes02_057747	AT2G47270.1	4.00E-14	cassava4.1_032069m	sequence-specific DNA binding transcription factors;transcrip	1.052	3.76E-06	6.33E-05
RknMes02_000792	AT2G19440.1	3.00E-30	cassava4.1_006689m	O-Glycosyl hydrolases family 17 protein	1.052	6.75E-06	9.36E-05
RknMes02_011990	AT1G11790.2	2.00E-37	cassava4.1_008924m	arogenate dehydratase 1	1.051	6.47E-06	9.10E-05
RknMes02_007251	AT1G06570.1	1.00E-22	cassava4.1_007612m	phytoene desaturation 1	1.048	1.86E-07	1.01E-05
RknMes02_029234	AT1G65660.1	2.00E-25	cassava4.1_006364m	Pre-mRNA splicing Prp18-interacting factor	1.048	8.82E-08	6.89E-06
RknMes02_030135	AT3G08710.2	0	cassava4.1_018663m	C-terminal cysteine residue is changed to a serine 2///thioredo	1.047	3.37E-09	1.31E-06
RknMes02_011851	AT5G64170.2	6.00E-24	cassava4.1_026053m	dentin sialophosphoprotein-related	1.046	5.64E-06	8.29E-05
RknMes02_017125	AT5G12840.3	2.00E-26	cassava4.1_010819m	nuclear factor Y, subunit A1	1.046	3.85E-06	6.42E-05
RknMes02_054970	AT5G64170.2	1.00E-13	cassava4.1_027467m	dentin sialophosphoprotein-related	1.046	2.38E-06	4.68E-05
RknMes02_018895					1.042	6.97E-07	2.13E-05
RknMes02_006347	AT4G36750.1	0	cassava4.1_015848m	Quinone reductase family protein	1.039	1.61E-09	9.89E-07
RknMes02_005986	AT1G26190.1	2.00E-27	cassava4.1_003200m	Phosphoribulokinase / Uridine kinase family	1.036	5.03E-06	7.68E-05
RknMes02_008505			cassava4.1_015853m		1.035	1.97E-06	4.14E-05
RknMes02_006958					1.031	3.48E-07	1.42E-05
RknMes02_000231	AT1G29400.2	2.00E-16	cassava4.1_001549m	MEI2-like protein 5	1.031	8.84E-09	2.01E-06
RknMes02_004082	AT1G17840.1	1.00E-17	cassava4.1_005391m	white-brown complex homolog protein 11	1.030	5.04E-07	1.73E-05
RknMes02_003952	AT2G39800.4	8.00E-25	cassava4.1_002374m	delta1-pyrroline-5-carboxylate synthase 1	1.030	1.31E-06	3.19E-05
RknMes02_046633	AT5G59613.2	2.00E-13		unknown protein; FUNCTIONS IN: molecular function unkn	1.030	7.34E-08	6.19E-06
RknMes02_006328			cassava4.1_015582m		1.029	2.19E-07	1.10E-05
RknMes02_000049	AT3G01170.1	9.00E-39	cassava4.1_015265m	Ribosomal protein L34e superfamily protein	1.027	4.06E-06	6.64E-05
RknMes02_012263	AT3G10030.1	2.00E-24	cassava4.1_006212m	aspartate/glutamate/uridylate kinase family protein	1.026	1.41E-07	8.80E-06
RknMes02_048355	AT5G48910.1	0	cassava4.1_005328m	Pentatricopeptide repeat (PPR) superfamily protein	1.024	1.83E-07	9.96E-06
RknMes02_005750	AT2G12550.1	3.00E-10	cassava4.1_004722m	ubiquitin-associated (UBA)/TS-N domain-containing protein	1.017	5.91E-07	1.90E-05
RknMes02_034982	AT2G35800.1	0	cassava4.1_001770m	mitochondrial substrate carrier family protein	1.017	3.03E-07	1.32E-05
RknMes02_012981	AT5G41000.1	4.00E-22	cassava4.1_003082m	YELLOW STRIPE like 4	1.014	1.30E-06	3.17E-05
RknMes02_027326	AT4G30996.1	1.00E-35	cassava4.1_020275m	Protein of unknown function (DUF1068)	1.013	1.05E-06	2.75E-05
RknMes02_055626	AT1G48960.1	0	cassava4.1_028587m	Adenine nucleotide alpha hydrolases-like superfamily protein	1.013	2.39E-07	1.16E-05
RknMes02_006624	AT1G80380.4	2.00E-22	cassava4.1_006274m	P-loop containing nucleoside triphosphate hydrolases superfa	1.012	8.13E-07	2.34E-05
RknMes02_022632					1.009	2.34E-07	1.15E-05
RknMes02_024529			cassava4.1_007227m		1.009	2.48E-06	4.79E-05
RknMes02_011668	AT2G21430.1	5.00E-24	cassava4.1_011689m	Papain family cysteine protease	1.008	4.21E-06	6.81E-05
RknMes02_007393	AT5G51100.1	6.00E-40	cassava4.1_016018m	Fe superoxide dismutase 2	1.008	5.98E-06	8.61E-05
RknMes02_035124	AT3G05950.1	0	cassava4.1_015731m	RmlC-like cupins superfamily protein	1.008	3.89E-07	1.51E-05
RknMes02_005894			cassava4.1_010028m		1.007	3.44E-07	1.42E-05
RknMes02_003142	AT2G45750.1	7.00E-37	cassava4.1_028934m	S-adenosyl-L-methionine-dependent methyltransferases super	1.006	4.18E-06	6.77E-05
RknMes02_015026	AT1G08250.1	0	cassava4.1_034329m	arogenate dehydratase 6	1.006	3.33E-06	5.82E-05
RknMes02_023830	AT2G03340.1	1.00E-16	cassava4.1_009819m	WRKY DNA-binding protein 3///WRKY DNA-binding protei	1.006	5.09E-06	7.75E-05
RknMes02_058925	AT1G26580.1	0	cassava4.1_034005m	FUNCTIONS IN: molecular function unknown; INVOLVED	1.002	1.91E-08	3.10E-06
RknMes02_010304	AT4G29010.1	3.00E-06		Unknown///Enoyl-CoA hydratase/isomerase family	1.002	2.58E-07	1.21E-05
RknMes02_038435			cassava4.1_012036m		1.002	6.10E-06	8.72E-05
RknMes02_036378	AT2G32150.1	0	cassava4.1_014164m	Haloacid dehalogenase-like hydrolase (HAD) superfamily pro	1.001	2.10E-07	1.08E-05
RknMes02_039561	AT1G14590.1	2.00E-33	cassava4.1_008667m	Nucleotide-diphospho-sugar transferase family protein	1.001	6.52E-07	2.03E-05
RknMes02_004547			cassava4.1_005454m		1.000	1.57E-07	9.23E-06
RknMes02_014517	AT1G23090.1	1.00E-42	cassava4.1_006086m	sulfate transporter 91	1.000	3.28E-06	5.74E-05

1) AGI code is shown if proteins encoded in each cassava gene (probe ID) have high amino acid sequence similarity (E value $\leq 10^{-5}$) to *Arabidopsis* homologs.

2) E-value shows similarity in amino acid sequence between each cassava gene (probe ID) and *Arabidopsis* homolog.

3) Encoded proteins/other features indicate the putative functions of the gene products that are expected from sequence similarity. The information for the NCBI protein reference sequence with the highest sequence similarity to the probes is shown.

4) Plants that were not pretreated with SAHA were used.

TABLE S2. Genes up-regulated in cassava roots in response to addition of 200 mM NaCl for 24 h

Probe ID	AGI code[1)]	E-value[2)]	Cassava ID	Encoded proteins/other features[3)]	w/o SAHA[4)] log_2 ratio (24 h NaCl /0 h NaCl)	p-value	BH FDR
RknMes02_038832	AT3G24310.1	0	cassava4.1 014512r	myb domain protein 305	6.437	1.85E-07	1.01E-05
RknMes02_006505	AT1G52690.2	3.00E-18	cassava4.1 025947r	Late embryogenesis abundant protein (LEA) family protein	5.890	2.25E-08	3.36E-06
RknMes02_033569	AT3G63060.1	0	cassava4.1 030886r	EID1-like 3	5.813	1.61E-06	3.62E-05
RknMes02_057161	AT4G33467.2	3.00E-09	cassava4.1 031129r	AT4G33467.2	5.701	2.29E-07	1.13E-05
RknMes02_049529	AT3G54420.1	0	cassava4.1 014554r	homolog of carrot EP3-3 chitinase	5.653	1.95E-06	4.12E-05
RknMes02_035302	AT3G24310.1	0	cassava4.1 013947r	myb domain protein 305	5.443	1.20E-06	3.00E-05
RknMes02_010887	AT2G43590.1	2.00E-12	cassava4.1 014554r	Chitinase family protein	5.245	3.71E-06	6.27E-05
RknMes02_008877	AT4G31240.2	6.00E-06	cassava4.1 012358r	protein kinase C-like zinc finger protein	5.162	1.14E-07	7.87E-06
RknMes02_027987	AT1G60420.1	0	cassava4.1 008293r	DC1 domain-containing protein	5.143	8.74E-08	6.85E-06
RknMes02_017023	AT4G16160.2	2.00E-44	cassava4.1 017521r	Mitochondrial import inner membrane translocase subunit Tim17/Tim2	5.118	1.12E-09	8.36E-07
RknMes02_011555	AT1G60420.1	1.00E-06	cassava4.1 008371r	DC1 domain-containing protein	4.655	9.67E-07	2.61E-05
RknMes02_030127	AT1G60420.1	2.00E-33	cassava4.1 008371r	DC1 domain-containing protein	4.498	2.92E-06	5.30E-05
RknMes02_003939	AT5G51760.1	5.00E-25	cassava4.1 013309r	Protein phosphatase 2C family protein///Unknown	4.407	2.96E-06	5.34E-05
RknMes02_006481	AT5G51760.1	6.00E-18	cassava4.1 013309r	Protein phosphatase 2C family protein///Unknown	4.279	5.48E-06	8.12E-05
RknMes02_052347	AT3G51810.1	1.00E-38	cassava4.1 022993r	Stress induced protein	4.200	4.54E-06	7.15E-05
RknMes02_010484	AT5G57050.1	3.00E-11	cassava4.1 010060r	Protein phosphatase 2C family protein	4.153	1.82E-06	3.93E-05
RknMes02_039460	AT5G59220.1	1.00E-25	cassava4.1 010060r	Protein phosphatase 2C family protein///highly ABA-induced PP2C ger	3.978	2.21E-06	4.47E-05
RknMes02_056041	AT3G30210.1	0	cassava4.1 029259r	myb domain protein 121	3.893	1.78E-09	1.01E-06
RknMes02_018746			cassava4.1_010060m		3.850	3.80E-06	6.38E-05
RknMes02_055947	AT1G16770.1	0	cassava4.1 029113r	AT1G16770.1	3.846	4.98E-09	1.54E-06
RknMes02_025528	AT3G14440.1	0	cassava4.1 026283r	nine-cis-epoxycarotenoid dioxygenase 3	3.832	2.19E-06	4.44E-05
RknMes02_047856	AT5G03850.1	1.00E-21		Nucleic acid-binding, OB-fold-like protein	3.734	2.84E-06	5.20E-05
RknMes02_051689	AT4G35680.1	7.00E-39	cassava4.1 021861r	Arabidopsis protein of unknown function (DUF241)	3.571	5.83E-07	1.88E-05
RknMes02_042836	AT5G03850.1	1.00E-21		Nucleic acid-binding, OB-fold-like protein	3.564	2.31E-06	4.59E-05
RknMes02_006068	AT1G07430.1	2.00E-20	cassava4.1 007913r	highly ABA-induced PP2C gene 2	3.526	1.03E-07	7.43E-06
RknMes02_023528			cassava4.1_007913m		3.493	1.04E-06	2.74E-05
RknMes02_004737	AT3G14440.1	0	cassava4.1 026283r	nine-cis-epoxycarotenoid dioxygenase 3	3.486	2.84E-06	5.21E-05
RknMes02_034838	AT2G29380.1	4.00E-37	cassava4.1 007913r	highly ABA-induced PP2C gene 3	3.479	2.12E-07	1.08E-05
RknMes02_024963	AT5G59220.1	1.00E-12	cassava4.1 007913r	Protein phosphatase 2C family protein///highly ABA-induced PP2C ger	3.430	1.42E-07	8.83E-06
RknMes02_016407	AT3G61890.1	1.00E-12	cassava4.1 015049r	homeobox 12///homeobox 7	3.312	3.26E-07	1.38E-05
RknMes02_039694	AT3G03341.1	2.00E-26	cassava4.1 020659r	unknown protein; Has 30201 Blast hits to 17322 proteins in 780 specie	3.288	7.35E-08	6.19E-06
RknMes02_013832			cassava4.1_015049m		3.211	2.96E-07	1.30E-05
RknMes02_035128	AT1G44446.3	2.00E-34	cassava4.1 023978r	Pheophorbide a oxygenase family protein with Rieske [2Fe-2S] domain	3.193	5.42E-06	8.07E-05
RknMes02_002573	AT5G59220.1	3.00E-15	cassava4.1 007998r	Protein phosphatase 2C family protein///highly ABA-induced PP2C ger	3.161	2.37E-06	4.68E-05
RknMes02_053159	AT4G16835.1	3.00E-14	cassava4.1 024383r	Tetratricopeptide repeat (TPR)-like superfamily protein	3.142	1.44E-07	8.93E-06
RknMes02_054176	AT5G20230.1	6.00E-18	cassava4.1 026113r	Unknown///blue-copper-binding protein	3.117	6.08E-07	1.93E-05
RknMes02_048392	AT2G38940.1	0	cassava4.1 005715r	phosphate transporter 1;7///Unknown///phosphate transporter 1;5///pho:	2.986	6.58E-08	5.87E-06
RknMes02_010463			cassava4.1_015049m		2.957	2.80E-07	1.26E-05
RknMes02_031261	AT4G27410.2	0	cassava4.1 010999r	NAC (No Apical Meristem) domain transcriptional regulator superfami	2.952	1.87E-07	1.01E-05
RknMes02_056689	AT3G59850.1	5.60E-45	cassava4.1 030364r	Pectin lyase-like superfamily protein	2.922	3.36E-08	4.17E-06
RknMes02_058983	AT1G24530.1	0	cassava4.1 034104r	Transducin/WD40 repeat-like superfamily protein	2.901	2.37E-07	1.15E-05
RknMes02_054233	AT5G42200.1	2.00E-33	cassava4.1 026205r	RING/U-box superfamily protein	2.829	4.88E-08	4.97E-06
RknMes02_006418	AT1G18100.1	1.00E-26	cassava4.1 021034r	PEBP (phosphatidylethanolamine-binding protein) family protein	2.819	3.32E-08	4.15E-06
RknMes02_010142	AT5G66110.1	4.00E-14	cassava4.1 018348r	Heavy metal transport/detoxification superfamily protein	2.742	6.49E-08	5.83E-06
RknMes02_054543	AT1G60190.1	0	cassava4.1 026727r	ARM repeat superfamily protein	2.669	1.50E-06	3.46E-05
RknMes02_046170	AT2G43870.1	7.00E-11		Pectin lyase-like superfamily protein	2.653	5.77E-07	1.88E-05
RknMes02_009426	AT5G14860.1	2.90E-44	cassava4.1 022533r	UDP-Glycosyltransferase superfamily protein	2.641	3.24E-06	5.69E-05
RknMes02_025204	AT1G09960.1	4.00E-33	cassava4.1 007925r	Unknown///sucrose transporter 4	2.611	6.83E-06	9.43E-05
RknMes02_007842	AT2G29380.1	7.00E-27	cassava4.1 013372r	highly ABA-induced PP2C gene 3	2.593	3.73E-08	4.40E-06
RknMes02_050519			cassava4.1_019400m		2.586	3.26E-06	5.72E-05
RknMes02_008222	AT3G20300.1	0	cassava4.1 008956r	Protein of unknown function (DUF3537)	2.571	2.47E-10	3.87E-07
RknMes02_024239	AT1G67300.2	3.00E-39	cassava4.1 006947r	Major facilitator superfamily protein	2.569	1.48E-08	2.65E-06
RknMes02_016270					2.543	3.05E-08	3.94E-06
RknMes02_023448			cassava4.1_009880m		2.526	2.50E-06	4.80E-05
RknMes02_010763			cassava4.1_014981m		2.524	6.27E-07	1.98E-05
RknMes02_056706	AT1G11530.1	2.00E-37	cassava4.1 030398r	C-terminal cysteine residue is changed to a serine 1	2.512	2.12E-07	1.08E-05
RknMes02_001296	AT1G07430.1	2.00E-12	cassava4.1 008067r	highly ABA-induced PP2C gene 2	2.474	4.03E-08	4.53E-06
RknMes02_031316	AT5G13200.1	0	cassava4.1 013991r	GRAM domain family protein	2.471	5.05E-06	7.70E-05
RknMes02_049597	AT3G61890.1	0	cassava4.1 014981r	homeobox 12///homeobox 7	2.444	9.07E-07	2.51E-05
RknMes02_050000	AT4G15630.1	0	cassava4.1 016898r	Uncharacterised protein family (UPF0497)	2.439	4.30E-06	6.89E-05
RknMes02_056633	AT1G29050.1	0	cassava4.1 030267r	TRICHOME BIREFRINGENCE-LIKE 38	2.431	1.64E-07	9.40E-06
RknMes02_009395	AT2G40170.1	3.00E-14	cassava4.1 022993r	Stress induced protein	2.406	7.15E-06	9.73E-05
RknMes02_043452	AT5G05480.1	2.00E-17		Peptide-N4-(N-acetyl-beta-glucosaminyl)asparagine amidase A protein	2.358	5.53E-06	8.17E-05
RknMes02_050301	AT5G66110.1	0	cassava4.1 018348r	Heavy metal transport/detoxification superfamily protein	2.356	2.01E-06	4.19E-05
RknMes02_000283	AT5G38940.1	0	cassava4.1 029662r	RmlC-like cupins superfamily protein	2.346	4.39E-06	6.98E-05
RknMes02_049538	AT1G74650.1	0	cassava4.1 014624r	myb domain protein 31///myb domain protein 96	2.343	1.26E-08	2.44E-06
RknMes02_049656	AT3G61890.1	0	cassava4.1 015295r	homeobox 12///homeobox 7	2.325	6.70E-07	2.07E-05
RknMes02_047043	AT5G60900.1	1.00E-07		receptor-like protein kinase 1	2.289	2.74E-06	5.10E-05
RknMes02_029383	AT2G41190.1	0	cassava4.1 007924r	Transmembrane amino acid transporter family protein	2.285	3.41E-06	5.90E-05
RknMes02_013552	AT3G11410.1	6.00E-06	cassava4.1 008067r	protein phosphatase 2CA	2.267	5.16E-07	1.75E-05
RknMes02_013801			cassava4.1_007311m		2.263	2.33E-06	4.62E-05
RknMes02_051011	AT3G48660.1	2.00E-28	cassava4.1 020761r	related to ABI3/VP1 2///Protein of unknown function (DUF 3339)	2.245	3.77E-07	1.48E-05
RknMes02_038567					2.243	1.08E-06	2.80E-05
RknMes02_036024	AT5G64750.1	5.00E-38	cassava4.1 007311r	ethylene response factor 110///Unknown///Integrase-type DNA-binding	2.219	3.12E-06	5.55E-05
RknMes02_024402	AT4G01970.1	0	cassava4.1 028359r	stachyose synthase	2.201	1.90E-06	4.04E-05
RknMes02_041021	AT5G15190.2	4.00E-08	cassava4.1 019923r	AT5G15190.1///Unknown///"unknown protein; FUNCTIONS IN: mole	2.170	2.56E-06	4.87E-05
RknMes02_055268	AT4G25410.1	7.00E-33	cassava4.1 027962r	basic helix-loop-helix (bHLH) DNA-binding superfamily protein	2.162	2.34E-07	1.15E-05
RknMes02_034016	AT4G12300.1	0	cassava4.1 005921r	cytochrome P450, family 706, subfamily A, polypeptide 4	2.154	1.40E-06	3.33E-05
RknMes02_040118	AT3G07130.1	0	cassava4.1 005206r	purple acid phosphatase 15	2.143	4.95E-06	7.59E-05
RknMes02_026845	AT4G30780.1	0	cassava4.1 005969r	unknown protein; BEST Arabidopsis thaliana protein match is: unknow	2.131	8.93E-08	6.93E-06
RknMes02_040003	AT1G67300.1	0	cassava4.1 006947r	Major facilitator superfamily protein	2.089	5.93E-08	5.54E-06
RknMes02_024400					2.085	5.85E-07	1.88E-05
RknMes02_030608	AT2G40610.1	0	cassava4.1 014549r	expansin A8	2.045	5.36E-08	5.27E-06
RknMes02_024608	AT3G04620.1	0	cassava4.1 023523r	Alba DNA/RNA-binding protein	2.043	1.13E-06	2.89E-05
RknMes02_020964	AT1G12780.1	0	cassava4.1 010908r	UDP-D-glucose/UDP-D-galactose 4-epimerase 1	2.041	5.38E-07	1.80E-05
RknMes02_039645	AT1G74930.1	3.00E-29	cassava4.1 018072r	Integrase-type DNA-binding superfamily protein	2.028	2.07E-07	1.07E-05
RknMes02_013004	AT3G59140.1	2.00E-25	cassava4.1 000219r	multidrug resistance-associated protein 14///Unknown	2.027	5.27E-07	1.78E-05
RknMes02_003789	AT3G26170.1	8.00E-15	cassava4.1 005921r	cytochrome P450, family 71, subfamily B, polypeptide 2	2.009	1.20E-06	3.00E-05
RknMes02_013327	AT2G24100.1	8.00E-06	cassava4.1 005969r	Unknown///AT2G24100.1///unknown protein; BEST Arabidopsis thalia	1.995	3.34E-07	1.40E-05
RknMes02_034114	AT5G26667.3	7.00E-27	cassava4.1 016091r	P-loop containing nucleoside triphosphate hydrolases superfamily prote	1.991	2.46E-07	1.18E-05
RknMes02_019648	AT1G30070.2	1.00E-08	cassava4.1 013041r	Unknown///SGS domain-containing protein	1.988	4.84E-06	7.48E-05
RknMes02_039767			cassava4.1_008879m		1.986	4.71E-07	1.67E-05
RknMes02_048776	AT2G19810.1	0	cassava4.1 009450r	Zinc finger C-x8-C-x5-C-x3-H type family protein///CCCH-type zinc fi	1.979	4.21E-07	1.58E-05
RknMes02_046633	AT5G59613.2	2.00E-13		unknown protein; FUNCTIONS IN: molecular function unknown; INV	1.977	7.34E-08	6.19E-06
RknMes02_010945	AT5G15410.1	0	cassava4.1 005026r	Cyclic nucleotide-regulated ion channel family protein	1.973	2.64E-06	4.97E-05
RknMes02_057053	AT1G06620.1	0	cassava4.1 030971r	2-oxoglutarate (2OG) and Fe(II)-dependent oxygenase superfamily prot	1.972	1.34E-06	3.24E-05
RknMes02_022193	AT1G67810.1	1.00E-23	cassava4.1 014023r	sulfur E2	1.967	2.00E-07	1.05E-05
RknMes02_056406	AT2G30230.1	3.00E-07	cassava4.1 029869r	unknown protein; FUNCTIONS IN: molecular function unknown; INV	1.956	1.37E-08	2.57E-06
RknMes02_052114	AT3G14670.2	3.00E-06	cassava4.1 022594r	cassava4.1_022594m	1.948	1.41E-06	3.34E-05
RknMes02_036684	AT5G54240.1	0	cassava4.1 013599r	Protein of unknown function (DUF1223)	1.947	7.58E-07	2.26E-05
RknMes02_027432	AT3G60290.1	3.00E-33	cassava4.1 012576r	2-oxoglutarate (2OG) and Fe(II)-dependent oxygenase superfamily prot	1.945	3.00E-08	3.92E-06
RknMes02_046123			cassava4.1_030952m		1.945	3.65E-07	1.46E-05
RknMes02_039735	AT1G71120.1	0	cassava4.1 034345r	GDSL-motif lipase/hydrolase 6	1.936	4.69E-08	4.92E-06
RknMes02_007092			cassava4.1_000175m		1.933	1.12E-06	2.87E-05
RknMes02_054138	AT1G58350.2	0	cassava4.1 026056r	Putative serine esterase family protein	1.932	2.02E-07	1.06E-05
RknMes02_038531			cassava4.1_019298m		1.920	1.11E-06	2.84E-05
RknMes02_053786	AT2G24130.1	0	cassava4.1 025435r	Leucine-rich receptor-like protein kinase family protein	1.900	8.68E-08	6.83E-06
RknMes02_026790	AT2G26870.1	0	cassava4.1 005538r	non-specific phospholipase C2	1.894	4.07E-12	6.01E-08
RknMes02_000186	AT3G59140.1	1.00E-39	cassava4.1 000219r	multidrug resistance-associated protein 14///Unknown	1.893	9.21E-07	2.53E-05
RknMes02_024117	AT1G27950.1	2.00E-40	cassava4.1 016986r	glycosylphosphatidylinositol-anchored lipid protein transfer 1	1.884	1.47E-07	9.03E-06
RknMes02_056865	AT3G61060.1	0	cassava4.1 030662r	phloem protein 2-A13	1.880	1.18E-06	2.96E-05
RknMes02_031133	AT5G56840.1	0	cassava4.1 014027r	myb-like transcription factor family protein	1.867	9.09E-08	7.01E-06
RknMes02_057376			cassava4.1_031490m		1.867	1.06E-07	7.53E-06
RknMes02_010471	AT3G54100.1	1.00E-09	cassava4.1 030087r	O-fucosyltransferase family protein	1.854	2.45E-06	4.75E-05
RknMes02_006260			cassava4.1_005969m		1.836	3.60E-07	1.45E-05
RknMes02_025517	AT5G59720.1	1.00E-08		heat shock protein 18.2///HSP20-like chaperones superfamily protein	1.834	5.67E-06	8.31E-05
RknMes02_032133	AT1G67300.2	7.00E-34	cassava4.1 008245r	Major facilitator superfamily protein	1.834	1.02E-09	8.07E-07
RknMes02_051397	AT4G35690.1	2.00E-30	cassava4.1 021395r	Arabidopsis protein of unknown function (DUF241)///Unknown	1.827	6.70E-06	9.31E-05
RknMes02_055451	AT4G15630.1	0	cassava4.1 028267r	Uncharacterised protein family (UPF0497)	1.811	1.04E-06	2.74E-05
RknMes02_009448	AT1G05650.1	6.00E-30	cassava4.1 031277r	Pectin lyase-like superfamily protein	1.808	2.11E-06	4.33E-05
RknMes02_026846	AT2G24100.1	7.00E-13	cassava4.1 005969r	Unknown///AT2G24100.1///unknown protein; BEST Arabidopsis thalia	1.802	6.99E-08	6.05E-06
RknMes02_009330	AT5G60900.1	6.00E-25	cassava4.1 001958r	receptor-like protein kinase 1	1.786	2.36E-06	4.66E-05

RknMes02_034350	AT3G14440.1	0	cassava4.1	034379r	nine-cis-epoxycarotenoid dioxygenase 3	1.783	1.19E-07	7.97E-06
RknMes02_020779	AT1G09020.1	0	cassava4.1	008100r	homolog of yeast sucrose nonfermenting 4	1.759	3.91E-06	6.49E-05
RknMes02_039925	AT5G55090.1	0	cassava4.1	008192r	NPK1-related protein kinase 2///Unknown///mitogen-activated protein	1.743	5.73E-07	1.87E-05
RknMes02_055484	AT5G60520.1	0	cassava4.1	028326r	Late embryogenesis abundant (LEA) protein-related	1.723	5.62E-10	5.85E-07
RknMes02_027464	AT2G30150.1	4.00E-39	cassava4.1	007201r	UDP-Glycosyltransferase superfamily protein	1.723	1.27E-09	8.44E-07
RknMes02_052275	AT5G01350.1	5.00E-13	cassava4.1	022869r	AT5G01350.1///unknown protein; FUNCTIONS IN: molecular_functio	1.694	1.82E-08	3.01E-06
RknMes02_024282			cassava4.1_027300m			1.682	5.31E-07	1.79E-05
RknMes02_051350	AT3G16175.1	3.00E-22	cassava4.1	021315r	Thioesterase superfamily protein	1.675	1.77E-07	9.85E-06
RknMes02_002662	AT4G02700.1	2.00E-32	cassava4.1	003230r	sulfate transporter 3;2	1.674	2.99E-07	1.31E-05
RknMes02_025998	AT2G18193.1	3.00E-28	cassava4.1	009105r	P-loop containing nucleoside triphosphate hydrolases superfamily prote	1.673	2.15E-06	4.38E-05
RknMes02_058864			cassava4.1_033900m			1.672	2.03E-06	4.21E-05
RknMes02_033796	AT5G53750.1	4.00E-13	cassava4.1	009282r	CBS domain-containing protein///Cystathionine beta-synthase (CBS) fa	1.655	6.62E-06	9.24E-05
RknMes02_056106	AT5G24030.1	0	cassava4.1	029368r	SLAC1 homologue 3	1.652	3.82E-06	6.40E-05
RknMes02_053823	AT2G42850.1	0	cassava4.1	025512r	cytochrome P450, family 718	1.646	9.15E-11	2.44E-07
RknMes02_005901	AT1G09020.1	2.00E-22	cassava4.1	008100r	homolog of yeast sucrose nonfermenting 4	1.641	3.30E-07	1.39E-05
RknMes02_050561	AT3G04620.1	2.00E-33	cassava4.1	019564r	Alba DNA/RNA-binding protein	1.613	1.61E-07	9.31E-06
RknMes02_022578	AT1G48100.1	2.00E-14	cassava4.1	006566r	Pectin lyase-like superfamily protein	1.609	4.29E-07	1.60E-05
RknMes02_002996	AT5G60760.1	7.00E-26	cassava4.1	002581r	P-loop containing nucleoside triphosphate hydrolases superfamily prote	1.608	5.33E-07	1.79E-05
RknMes02_031931	AT4G01870.1	0	cassava4.1	034137r	tolB protein-related	1.591	4.83E-07	1.69E-05
RknMes02_023155	AT3G14440.1	0	cassava4.1	034379r	nine-cis-epoxycarotenoid dioxygenase 3	1.581	7.76E-07	2.29E-05
RknMes02_033475	AT4G26080.1	4.00E-22	cassava4.1	020355r	Protein phosphatase 2C family protein	1.580	2.78E-07	1.25E-05
RknMes02_042075	AT5G43150.1	3.00E-06	cassava4.1	020299r	unknown protein; FUNCTIONS IN: molecular_function unknown; INV	1.574	5.70E-10	5.85E-07
RknMes02_055925	AT4G34135.1	0	cassava4.1	029076r	UDP-glucosyltransferase 73B2///UDP-glucosyl transferase 73B3	1.572	7.19E-09	1.80E-06
RknMes02_008884	AT5G15450.1	0	cassava4.1	001040r	casein lytic proteinase B3	1.571	5.95E-08	5.55E-06
RknMes02_056016	AT2G21490.1	1.00E-35	cassava4.1	029213r	dehydrin LEA	1.568	7.32E-11	2.44E-07
RknMes02_005365	AT2G37980.1	1.00E-14	cassava4.1	030087r	O-fucosyltransferase family protein	1.568	2.09E-07	1.07E-05
RknMes02_000416	AT2G26870.1	0	cassava4.1	005538r	non-specific phospholipase C2	1.562	1.37E-11	1.20E-07
RknMes02_017319	AT3G51895.1	0	cassava4.1	003230r	sulfate transporter 3;1	1.555	3.66E-06	6.21E-05
RknMes02_053272	AT5G60520.1	0	cassava4.1	024577r	Late embryogenesis abundant (LEA) protein-related	1.552	4.57E-08	4.86E-06
RknMes02_015829						1.543	9.79E-07	2.63E-05
RknMes02_004082	AT1G17840.1	1.00E-17	cassava4.1	005391r	white-brown complex homolog protein 11	1.541	5.04E-07	1.73E-05
RknMes02_048720	AT1G03790.1	0	cassava4.1	008973r	Zinc finger C-x8-C-x5-C-x3-H type family protein	1.541	4.36E-08	4.76E-06
RknMes02_022851	AT3G47800.1	0	cassava4.1	025611r	Galactose mutarotase-like superfamily protein	1.537	2.62E-09	1.18E-06
RknMes02_040811						1.533	1.77E-06	3.86E-05
RknMes02_000880	AT1G71960.1	1.00E-09	cassava4.1	004508r	ATP-binding casette family G25///Unknown	1.531	7.12E-06	9.71E-05
RknMes02_051750	AT1G58330.1	4.00E-14	cassava4.1	021958r	RESPONSE TO ABA AND SALT 1	1.523	4.13E-07	1.56E-05
RknMes02_025374	AT1G69840.7	0	cassava4.1	013311r	SPFH/Band 7/PHB domain-containing membrane-associated protein fa	1.514	2.53E-06	4.83E-05
RknMes02_015088	AT1G13990.1	6.00E-18	cassava4.1	013257r	AT1G13990.1///unknown protein; FUNCTIONS IN: molecular_functio	1.508	3.65E-07	1.46E-05
RknMes02_013842	AT5G15450.1	1.00E-09	cassava4.1	029777r	casein lytic proteinase B3	1.507	8.71E-08	6.85E-06
RknMes02_029387	AT5G67350.1	2.00E-38	cassava4.1	014725r	unknown protein; Has 1807 Blast hits to 1807 proteins in 277 species:	1.505	1.44E-06	3.37E-05
RknMes02_042961						1.501	1.03E-08	2.18E-06
RknMes02_057298	AT1G45616.1	0	cassava4.1	031360r	receptor like protein 7///receptor like protein 6///disease resistance fam	1.498	2.92E-06	5.30E-05
RknMes02_057990	AT2G21610.1	0	cassava4.1	032455r	pectinesterase 11	1.491	4.26E-08	4.68E-06
RknMes02_000783	AT4G27460.1	1.00E-32	cassava4.1	009282r	CBS domain-containing protein	1.490	4.23E-06	6.83E-05
RknMes02_058966	AT5G49330.1	0	cassava4.1	034078r	myb domain protein 111///myb domain protein 15	1.484	2.52E-06	4.83E-05
RknMes02_006060	AT1G47980.1	1.00E-34	cassava4.1	012167r	unknown protein; FUNCTIONS IN: molecular_function unknown; INV	1.475	3.98E-06	6.57E-05
RknMes02_002455	AT3G46780.1	2.00E-30	cassava4.1	006229r	plastid transcriptionally active 16	1.467	5.64E-07	1.85E-05
RknMes02_010899	AT5G53750.1	3.00E-15	cassava4.1	009282r	CBS domain-containing protein///Cystathionine beta-synthase (CBS) fa	1.463	2.00E-06	4.16E-05
RknMes02_048226	AT3G16380.1	0	cassava4.1	004058r	poly(A) binding protein 6	1.462	1.86E-07	1.01E-05
RknMes02_000950	AT5G15450.1	0	cassava4.1	001040r	casein lytic proteinase B3	1.459	5.43E-09	1.60E-06
RknMes02_028371	AT4G01870.1	0	cassava4.1	034137r	tolB protein-related	1.456	4.85E-06	7.50E-05
RknMes02_047343						1.455	1.45E-08	2.64E-06
RknMes02_025738	AT5G13750.3	0	cassava4.1	025256r	zinc induced facilitator-like 1	1.455	7.57E-07	2.26E-05
RknMes02_019499						1.452	1.57E-07	9.25E-06
RknMes02_040923	AT5G13740.1	7.00E-30			zinc induced facilitator-like 2///zinc induced facilitator 1	1.448	1.48E-06	3.42E-05
RknMes02_013200	AT4G34131.1	3.00E-08	cassava4.1	029076r	UDP-glucosyl transferase 73B3	1.436	2.09E-08	3.27E-06
RknMes02_022618	AT2G29500.1	0	cassava4.1	033656r	HSP20-like chaperones superfamily protein	1.433	3.66E-09	1.37E-06
RknMes02_001711	AT4G32460.2	1.00E-19	cassava4.1	010077r	Protein of unknown function, DUF642	1.427	2.31E-09	1.13E-06
RknMes02_007712	AT3G16240.1	4.00E-13	cassava4.1	014710r	delta tonoplast integral protein///Unknown	1.427	5.44E-06	8.08E-05
RknMes02_049315	AT1G13990.1	0	cassava4.1	013257r	AT1G13990.1///unknown protein; FUNCTIONS IN: molecular_functio	1.425	1.81E-07	9.93E-06
RknMes02_056529	AT2G37980.1	0	cassava4.1	030087r	O-fucosyltransferase family protein	1.424	1.36E-06	3.27E-05
RknMes02_033789	AT1G73480.1	6.00E-09	cassava4.1	006764r	Unknown///alpha/beta-Hydrolases superfamily protein	1.413	1.78E-06	3.88E-05
RknMes02_031151	AT3G20660.1	0	cassava4.1	025738r	organic cation/carnitine transporter4	1.410	9.98E-07	2.65E-05
RknMes02_049247	AT4G36830.1	0	cassava4.1	012827r	GNS1/SUR4 membrane protein family	1.401	4.74E-06	7.36E-05
RknMes02_057542	AT3G20660.1	0	cassava4.1	031755r	organic cation/carnitine transporter4	1.398	4.22E-07	1.58E-05
RknMes02_007075	AT1G73480.1	8.00E-38	cassava4.1	006764r	Unknown///alpha/beta-Hydrolases superfamily protein	1.393	2.32E-06	4.60E-05
RknMes02_050613			cassava4.1_019740m			1.390	9.94E-07	2.65E-05
RknMes02_050840	AT4G22190.1	1.00E-06	cassava4.1	020395r	unknown protein; Has 283 Blast hits to 154 proteins in 44 species: Arc	1.385	4.52E-07	1.64E-05
RknMes02_009896	AT2G33830.2	1.00E-09			Dormancy/auxin associated family protein	1.378	7.33E-06	9.90E-05
RknMes02_039276	AT5G06300.1	0	cassava4.1	013240r	Putative lysine decarboxylase family protein	1.377	4.95E-06	7.58E-05
RknMes02_032910	AT3G10420.2	2.00E-39	cassava4.1	002788r	P-loop containing nucleoside triphosphate hydrolases superfamily prote	1.369	2.33E-07	1.14E-05
RknMes02_003633	AT3G13380.1	9.00E-11	cassava4.1	030762r	BRI1-like 3///BRI1 like	1.364	1.45E-06	3.38E-05
RknMes02_055734	AT2G39040.1	0	cassava4.1	028760r	Peroxidase superfamily protein	1.362	2.66E-08	3.66E-06
RknMes02_037703	AT2G45400.1	3.00E-33	cassava4.1	014136r	NAD(P)-binding Rossmann-fold superfamily protein	1.361	1.42E-06	3.35E-05
RknMes02_036843	AT2G30860.1	0	cassava4.1	016203r	glutathione S-transferase PHI 9	1.360	2.52E-06	4.83E-05
RknMes02_001446	AT2G47800.1	3.00E-32	cassava4.1	000205r	multidrug resistance-associated protein 5///multidrug resistance-associa	1.359	1.13E-07	7.83E-06
RknMes02_028518	AT5G05690.2	0	cassava4.1	006864r	Cytochrome P450 superfamily protein	1.357	1.64E-08	2.84E-06
RknMes02_055658	AT5G52570.1	0	cassava4.1	028637r	beta-carotene hydroxylase 2	1.356	3.42E-06	5.91E-05
RknMes02_015555	AT3G13110.1	4.00E-28	cassava4.1	034339r	serine acetyltransferase 2;2	1.352	9.83E-09	2.13E-06
RknMes02_052004	AT1G32700.1	0	cassava4.1	022404r	PLATZ transcription factor family protein	1.347	9.51E-09	2.10E-06
RknMes02_026630	AT2G17840.1	0	cassava4.1	008726r	Senescence/dehydration-associated protein-related	1.340	1.57E-06	3.56E-05
RknMes02_037859			cassava4.1_020412m			1.336	6.76E-08	5.96E-06
RknMes02_001770	AT5G05690.3	0	cassava4.1	015740r	Cytochrome P450 superfamily protein	1.332	7.21E-08	6.16E-06
RknMes02_037324	AT5G53750.1	4.00E-12	cassava4.1	009282r	CBS domain-containing protein///Cystathionine beta-synthase (CBS) fa	1.329	6.18E-06	8.81E-05
RknMes02_032390	AT3G21760.1	0	cassava4.1	031478r	UDP-Glycosyltransferase superfamily protein///UDP-glucosyl transfera	1.326	1.67E-06	3.72E-05
RknMes02_001781	AT3G58110.2	3.00E-28	cassava4.1	004503r	unknown protein; FUNCTIONS IN: molecular_function unknown; INV	1.317	3.51E-09	1.34E-06
RknMes02_039401	AT3G47640.3	6.00E-11	cassava4.1	015506r	basic helix-loop-helix (bHLH) DNA-binding superfamily protein	1.310	3.36E-06	5.85E-05
RknMes02_004093	AT3G10340.1	3.00E-12	cassava4.1	003117r	phenylalanine ammonia-lyase 2///phenylalanine ammonia-lyase 4	1.298	2.55E-06	4.86E-05
RknMes02_054014	AT5G55620.1	5.00E-21	cassava4.1	025845r	unknown protein; BEST Arabidopsis thaliana protein match is: unknow	1.298	4.01E-07	1.53E-05
RknMes02_032798	AT2G26070.1	0	cassava4.1	014738r	Protein of unknown function (DUF778)	1.296	1.13E-06	2.89E-05
RknMes02_011864			cassava4.1_029076m			1.294	2.23E-08	3.36E-06
RknMes02_004784	AT4G01870.1	0	cassava4.1	034137r	tolB protein-related	1.290	3.80E-07	1.49E-05
RknMes02_025883	AT4G05120.1	0	cassava4.1	008484r	Major facilitator superfamily protein	1.290	1.39E-06	3.31E-05
RknMes02_023158	AT5G02230.2	8.00E-31	cassava4.1	013061r	Haloacid dehalogenase-like hydrolase (HAD) superfamily protein	1.288	2.38E-06	4.68E-05
RknMes02_044166						1.279	2.36E-06	4.66E-05
RknMes02_036565	AT5G15450.1	1.00E-30	cassava4.1	001040r	casein lytic proteinase B3	1.279	2.12E-09	1.12E-06
RknMes02_026629	AT4G02940.1	3.00E-33	cassava4.1	004853r	oxidoreductase, 2OG-Fe(II) oxygenase family protein	1.278	2.02E-06	4.20E-05
RknMes02_009909	AT4G20360.1	1.00E-12			RAB GTPase homolog E1B///Unknown///"Nucleic acid-binding, OB-fo	1.274	3.81E-06	6.39E-05
RknMes02_013666	AT3G47950.1	1.00E-12	cassava4.1	001144r	H(+)-ATPase 11///H(+)-ATPase 4	1.271	2.95E-06	5.34E-05
RknMes02_055311	AT5G54370.1	3.00E-39	cassava4.1	028042r	Late embryogenesis abundant (LEA) protein-related	1.262	8.84E-07	2.48E-05
RknMes02_049066	AT5G13140.1	0	cassava4.1	011637r	Pollen Ole e 1 allergen and extensin family protein	1.261	6.22E-08	5.70E-06
RknMes02_031868	AT1G59740.1	0	cassava4.1	003848r	Major facilitator superfamily protein	1.261	4.13E-06	6.72E-05
RknMes02_005722	AT5G47020.1	2.00E-16	cassava4.1	028442r	AT5G47020.1///unknown protein; FUNCTIONS IN: molecular_functio	1.257	7.01E-07	2.14E-05
RknMes02_032229	AT1G07180.1	0	cassava4.1	008961r	alternative NAD(P)H dehydrogenase 2///alternative NAD(P)H dehydro	1.254	6.15E-06	8.79E-05
RknMes02_053380	AT2G19330.1	0	cassava4.1	024749r	plant intracellular ras group-related LRR 6	1.254	1.59E-08	2.78E-06
RknMes02_006585	AT3G10420.2	2.00E-26	cassava4.1	002788r	P-loop containing nucleoside triphosphate hydrolases superfamily prote	1.254	5.24E-07	1.77E-05
RknMes02_032671						1.249	3.39E-06	5.88E-05
RknMes02_010395			cassava4.1_001592m			1.248	3.67E-07	1.46E-05
RknMes02_013420			cassava4.1_010019m			1.246	6.00E-09	1.66E-06
RknMes02_024920	AT5G39000.1	6.00E-34	cassava4.1	002842r	Unknown///Malectin/receptor-like protein kinase family protein	1.242	2.30E-06	4.58E-05
RknMes02_008915	AT5G19900.1	4.00E-16	cassava4.1	005214r	Unknown	1.242	6.62E-06	9.24E-05
RknMes02_056263	AT2G34930.1	0	cassava4.1	029622r	disease resistance family protein / LRR family protein	1.240	2.46E-07	1.18E-05
RknMes02_015540	AT3G10420.2	1.00E-32	cassava4.1	002788r	P-loop containing nucleoside triphosphate hydrolases superfamily prote	1.238	4.66E-08	4.91E-06
RknMes02_007453	AT1G01320.2	0	cassava4.1	000135r	Tetratricopeptide repeat (TPR)-like superfamily protein	1.236	3.61E-06	6.15E-05
RknMes02_013976	AT1G59740.1	4.00E-12	cassava4.1	003848r	Major facilitator superfamily protein	1.235	5.49E-06	8.12E-05
RknMes02_017072	AT3G10340.1	0	cassava4.1	003117r	phenylalanine ammonia-lyase 2///phenylalanine ammonia-lyase 4	1.230	1.43E-06	3.35E-05
RknMes02_016900	AT1G01720.1	0	cassava4.1	013132r	NAC (No Apical Meristem) domain transcriptional regulator superfami	1.224	2.31E-08	3.37E-06
RknMes02_026966	AT2G20770.1	0	cassava4.1	008542r	GCR2-like 2	1.223	2.58E-06	4.89E-05
RknMes02_033743						1.221	9.97E-07	2.65E-05
RknMes02_033557	AT2G36630.1	0	cassava4.1	007299r	Sulfite exporter TauE/SafE family protein	1.219	1.20E-07	8.01E-06
RknMes02_008470	AT2G36630.1	1.00E-36	cassava4.1	007299r	Sulfite exporter TauE/SafE family protein	1.219	7.14E-08	6.14E-06
RknMes02_037858	AT3G51250.1	0	cassava4.1	008726r	Senescence/dehydration-associated protein-related	1.218	4.41E-06	7.00E-05
RknMes02_003198	AT5G15450.1	0	cassava4.1	001040r	casein lytic proteinase B3	1.213	1.58E-07	9.27E-06
RknMes02_021431						1.211	3.48E-07	1.42E-05
RknMes02_058917	AT1G23550.1	0	cassava4.1	033992r	similar to RCD one 2	1.208	5.41E-06	8.05E-05
RknMes02_051464	AT3G06240.1	1.00E-24	cassava4.1	021488r	F-box family protein///F-box and associated interaction domains-contai	1.204	1.78E-08	2.95E-06
RknMes02_038559	AT3G55610.1	1.00E-28	cassava4.1	002374r	delta 1-pyrroline-5-carboxylate synthase 2	1.204	5.22E-08	5.17E-06

RknMes02_037016	AT3G26300.1	0	cassava4.1 005635r	cytochrome P450, family 71, subfamily B, polypeptide 34///"cytochrom	1.197	2.30E-06	4.58E-05
RknMes02_010377			cassava4.1_008686m		1.196	9.50E-07	2.58E-05
RknMes02_002633			cassava4.1_034204m		1.188	2.43E-06	4.73E-05
RknMes02_006744	AT3G48990.1	7.00E-42	cassava4.1 005514r	Unknown///AMP-dependent synthetase and ligase family protein	1.187	4.81E-06	7.44E-05
RknMes02_050121	AT5G13740.1	0	cassava4.1 017498r	zinc induced facilitator-like 2///zinc induced facilitator 1	1.185	4.31E-06	6.91E-05
RknMes02_034493	AT2G42010.1	0	cassava4.1 000791r	cassava4.1 019836m///phospholipase D beta 1///phospholipase D delta	1.184	6.26E-08	5.72E-06
RknMes02_003952	AT2G39800.4	8.00E-25	cassava4.1 002374r	delta1-pyrroline-5-carboxylate synthase 1	1.180	1.31E-06	3.19E-05
RknMes02_038204	AT3G14460.1	5.00E-13	cassava4.1 000496r	NB-ARC domain-containing disease resistance protein///Unknown///LF	1.178	8.95E-07	2.49E-05
RknMes02_005129	AT1G62440.1	4.00E-06	cassava4.1 012776r	beta-galactosidase 8///Unknown	1.171	1.72E-06	3.79E-05
RknMes02_032932	AT4G29010.1	0	cassava4.1 029596r	Unknown///Enoyl-CoA hydratase/isomerase family	1.168	2.29E-07	1.13E-05
RknMes02_033706	AT1G33060.2	2.00E-25	cassava4.1 011070r	NAC 014	1.163	8.46E-07	2.41E-05
RknMes02_056665	AT5G20150.1	1.40E-45	cassava4.1 030318r	SPX domain gene 3///SPX domain gene 1	1.161	1.07E-06	2.78E-05
RknMes02_006939	AT5G60600.3	0	cassava4.1 002363r	4-hydroxy-3-methylbut-2-enyl diphosphate synthase	1.159	7.33E-08	6.19E-06
RknMes02_036162	AT1G21790.1	0	cassava4.1 024644r	TRAM, LAG1 and CLN8 (TLC) lipid-sensing domain containing prote	1.159	1.85E-09	1.04E-06
RknMes02_035114	AT1G06320.1	4.00E-16	cassava4.1 017107r	unknown protein; Has 24 Blast hits to 24 proteins in 10 species: Archa	1.156	4.09E-06	6.68E-05
RknMes02_039100	AT1G08830.2	0	cassava4.1 018987r	copper/zinc superoxide dismutase 1	1.154	6.94E-08	6.04E-06
RknMes02_056242	AT2G04240.2	0	cassava4.1 029590r	Unknown///RING/U-box superfamily protein///brassinosteroid-responsi	1.153	1.74E-07	9.78E-06
RknMes02_025705	AT3G16910.1	0	cassava4.1 004631r	acyl-activating enzyme 7	1.150	2.37E-07	1.15E-05
RknMes02_057627	AT4G32480.1	0	cassava4.1 031890r	Protein of unknown function (DUF506)	1.145	4.63E-07	1.66E-05
RknMes02_004493	AT3G27020.1	7.00E-37	cassava4.1 002751r	YELLOW STRIPE like 6	1.143	4.01E-06	6.60E-05
RknMes02_051505	AT1G52540.1	0	cassava4.1 021554r	Protein kinase superfamily protein	1.140	4.01E-06	6.60E-05
RknMes02_025897	AT2G36880.2	0	cassava4.1 009356r	methionine adenosyltransferase 3	1.138	1.90E-06	4.03E-05
RknMes02_053085			cassava4.1_024261m		1.138	6.92E-07	2.12E-05
RknMes02_010140	AT4G03260.2	2.00E-13		Outer arm dynein light chain 1 protein	1.136	5.70E-06	8.34E-05
RknMes02_001451	AT3G16050.1	0	cassava4.1 034454r	pyridoxine biosynthesis 1.2	1.124	1.27E-06	3.12E-05
RknMes02_056527	AT5G65300.1	6.00E-11	cassava4.1 030085r	AT5G65300.1	1.121	6.36E-06	8.97E-05
RknMes02_039995	AT4G36990.1	0	cassava4.1 013174r	heat shock factor 4	1.119	5.70E-06	8.34E-05
RknMes02_000305	AT5G15950.2	2.00E-44	cassava4.1 010527r	Adenosylmethionine decarboxylase family protein	1.113	4.70E-06	7.32E-05
RknMes02_041887			cassava4.1_019298m		1.111	3.96E-07	1.52E-05
RknMes02_040650	AT1G32200.2	4.00E-09		phospholipid/glycerol acyltransferase family protein	1.108	5.93E-06	8.55E-05
RknMes02_050463	AT2G34340.1	1.00E-28	cassava4.1 019132r	Protein of unknown function, DUF584	1.108	2.01E-09	1.09E-06
RknMes02_038607	AT1G18420.1	4.00E-06	cassava4.1 016459r	Aluminium activated malate transporter family protein	1.106	2.48E-07	1.18E-05
RknMes02_039868	AT3G04070.2	8.00E-16	cassava4.1 010010r	NAC domain containing protein 47	1.106	1.69E-07	9.58E-06
RknMes02_007572	AT3G18770.1	1.00E-14	cassava4.1 000289r	Autophagy-related protein 13///Unknown	1.105	4.73E-06	7.36E-05
RknMes02_016583					1.102	4.82E-07	1.69E-05
RknMes02_006801	AT3G04240.1	0	cassava4.1 002691r	Tetratricopeptide repeat (TPR)-like superfamily protein	1.101	2.10E-06	4.31E-05
RknMes02_002774	AT1G15290.1	2.00E-06	cassava4.1 034222r	Tetratricopeptide repeat (TPR)-like superfamily protein///Unknown	1.097	1.04E-06	2.74E-05
RknMes02_022087					1.095	6.29E-10	6.06E-07
RknMes02_003201	AT2G38280.2	2.00E-40	cassava4.1 001581r	AMP deaminase, putative / myoadenylate deaminase, putative	1.082	3.58E-08	4.31E-06
RknMes02_024633					1.079	3.26E-06	5.72E-05
RknMes02_006959	AT1G20960.2	0	cassava4.1 032346r	U5 small nuclear ribonucleoprotein helicase, putative	1.078	4.37E-06	6.97E-05
RknMes02_028012	AT1G06570.2	0	cassava4.1 007612r	phytoene desaturation 1	1.077	6.00E-07	1.91E-05
RknMes02_020844	AT2G46270.2	2.00E-35	cassava4.1 008455r	G-box binding factor 3	1.076	1.71E-07	9.64E-06
RknMes02_018780	AT5G52450.1	2.00E-41	cassava4.1 008918r	MATE efflux family protein	1.074	1.10E-07	7.75E-06
RknMes02_002097			cassava4.1_000487m		1.073	3.36E-07	1.40E-05
RknMes02_036257	AT5G23240.1	3.00E-29	cassava4.1 006681r	DNAJ heat shock N-terminal domain-containing protein	1.065	5.15E-07	1.75E-05
RknMes02_051754	AT2G44940.1	0	cassava4.1 021971r	Integrase-type DNA-binding superfamily protein	1.064	1.80E-07	9.93E-06
RknMes02_000350	AT1G36990.1	2.00E-21	cassava4.1 022907r	AT1G36990.1///unknown protein; LOCATED IN: chloroplast; EXPRES	1.060	3.72E-07	1.47E-05
RknMes02_023063					1.059	5.23E-06	7.88E-05
RknMes02_048914	AT3G18440.1	0	cassava4.1 010543r	aluminum-activated malate transporter 9///Unknown///Aluminium activ	1.059	6.24E-06	8.87E-05
RknMes02_048319	AT4G29000.1	0	cassava4.1 004978r	Tesmin/TSO1-like CXC domain-containing protein	1.058	3.38E-09	1.31E-06
RknMes02_010107	AT5G15450.1	1.00E-17	cassava4.1 004911r	casein lytic proteinase B3	1.057	3.13E-07	1.35E-05
RknMes02_029029	AT3G11410.1	0	cassava4.1 008162r	protein phosphatase 2CA	1.057	6.73E-06	9.34E-05
RknMes02_027619	AT1G60420.1	0	cassava4.1 004565r	DC1 domain-containing protein	1.056	2.83E-07	1.27E-05
RknMes02_054690	AT4G11360.1	3.00E-14	cassava4.1 026970r	RING/U-box superfamily protein	1.054	4.25E-08	4.68E-06
RknMes02_010304	AT4G29010.1	3.00E-06		Unknown///Enoyl-CoA hydratase/isomerase family	1.054	2.58E-07	1.21E-05
RknMes02_025071	AT4G02940.1	2.00E-31	cassava4.1 004959r	oxidoreductase, 2OG-Fe(II) oxygenase family protein	1.050	1.37E-08	2.57E-06
RknMes02_032939	AT1G17120.1	0	cassava4.1 003994r	cationic amino acid transporter 8	1.049	4.21E-06	6.81E-05
RknMes02_036224	AT1G69490.1	0	cassava4.1 013467r	NAC (No Apical Meristem) domain transcriptional regulator superfami	1.046	4.34E-06	6.94E-05
RknMes02_035498	AT2G40080.1	8.00E-28	cassava4.1 019555r	Protein of unknown function (DUF1313)	1.044	6.24E-08	5.71E-06
RknMes02_009729	AT2G38280.2	0	cassava4.1 001581r	AMP deaminase, putative / myoadenylate deaminase, putative	1.044	3.48E-07	1.42E-05
RknMes02_043801					1.044	4.32E-08	4.73E-06
RknMes02_056452	AT2G24960.2	0	cassava4.1 029947r	unknown protein; FUNCTIONS IN: molecular function unknown; INV	1.044	3.45E-08	4.23E-06
RknMes02_006377	AT1G64060.1	1.00E-34	cassava4.1 001193r	respiratory burst oxidase protein F	1.042	2.63E-07	1.22E-05
RknMes02_021298					1.041	3.08E-07	1.33E-05
RknMes02_005986	AT1G26190.1	2.00E-27	cassava4.1 003200r	Phosphoribulokinase / Uridine kinase family	1.040	5.03E-06	7.68E-05
RknMes02_047584					1.035	1.43E-06	3.35E-05
RknMes02_021498	AT3G56400.1	2.00E-35	cassava4.1 033846r	WRKY DNA-binding protein 70	1.034	2.48E-07	1.18E-05
RknMes02_008115	AT1G67810.1	9.00E-35	cassava4.1 014023r	sulfur E2	1.033	2.00E-06	4.18E-05
RknMes02_033431	AT5G09590.1	0	cassava4.1 002955r	mitochondrial HSO70 2	1.030	8.78E-07	2.47E-05
RknMes02_026782	AT2G22600.1	1.00E-18		RNA-binding KH domain-containing protein	1.025	2.89E-06	5.27E-05
RknMes02_021003	AT1G12845.1	3.00E-17	cassava4.1 029487r	unknown protein; FUNCTIONS IN: molecular function unknown; INV	1.023	4.24E-07	1.59E-05
RknMes02_047925	AT3G18100.1	0	cassava4.1 000461r	myb domain protein 4r1	1.018	2.08E-06	4.29E-05
RknMes02_048414	AT1G72770.3	0	cassava4.1 005959r	homology to ABI1	1.018	8.72E-10	7.37E-07
RknMes02_014638	AT5G15950.2	2.00E-14	cassava4.1 021499r	Adenosylmethionine decarboxylase family protein	1.015	6.91E-06	9.50E-05
RknMes02_012677	AT4G32710.1	1.00E-10	cassava4.1 002563r	Protein kinase superfamily protein///protein serine/threonine kinases;pr	1.015	1.41E-07	8.79E-06
RknMes02_002166	AT5G20250.4	2.00E-31	cassava4.1 002004r	Raffinose synthase family protein	1.013	2.48E-07	1.18E-05
RknMes02_026166	AT3G27010.1	0	cassava4.1 034237r	TEOSINTE BRANCHED 1, cycloidea, PCF (TCP)-domain family prot	1.010	2.24E-07	1.12E-05
RknMes02_049514	AT5G02230.2	0	cassava4.1 014451r	Haloacid dehalogenase-like hydrolase (HAD) superfamily protein	1.008	1.73E-06	3.81E-05
RknMes02_031268			cassava4.1_010104m		1.006	2.80E-08	3.75E-06
RknMes02_024410	AT5G25350.1	0	cassava4.1 003247r	EIN3-binding F box protein 2	1.006	8.51E-07	2.42E-05
RknMes02_036711	AT1G72770.3	0	cassava4.1 004984r	homology to ABI1	1.005	8.02E-10	6.97E-07
RknMes02_012447	AT5G15810.1	8.00E-12	cassava4.1 003890r	N2,N2-dimethylguanosine tRNA methyltransferase	1.003	1.81E-06	3.92E-05
RknMes02_001315	AT4G29010.1	0	cassava4.1 006963r	Unknown///Enoyl-CoA hydratase/isomerase family	1.003	1.53E-07	9.15E-06
RknMes02_027347	AT4G10250.1	4.00E-18	cassava4.1 028591r	HSP20-like chaperones superfamily protein	1.003	1.10E-06	2.83E-05
RknMes02_052892			cassava4.1_023929m		1.003	1.80E-06	3.91E-05
RknMes02_013330	AT1G32640.1	7.00E-16	cassava4.1 002918r	Unknown///Basic helix-loop-helix (bHLH) DNA-binding family protein	1.003	4.07E-06	6.66E-05
RknMes02_008336	AT5G42810.1	8.00E-23	cassava4.1 007401r	inositol-pentakisphosphate 2-kinase 1	1.001	6.33E-06	8.93E-05

1) AGI code is shown if proteins encoded in each cassava gene (probe ID) have high amino acid sequence similarity (E value ≤ 10^{-5}) to *Arabidopsis* homologs.

2) E-value shows similarity in amino acid sequence between each cassava gene (probe ID) and *Arabidopsis* homolog.

3) Encoded proteins/other features indicate the putative functions of the gene products that are expected from sequence similarity. The information for the NCBI protein reference sequence with the highest sequence similarity to the probes is shown.

4) Plants that were not pretreated with SAHA were used.

TABLE S3. Genes up-regulated in cassava roots by SAHA treatment

Probe ID	AGI code[1)]	E-value[2)]	Cassava ID	Encoded proteins/other features[3)]	log_2 ratio (SAHA 24 h/non-treated)	p-value	BH FDR
RknMes02 051874	AT1G13280.1	0	cassava4.1 022180	allene oxide cyclase 4	5.135	3.51E-11	1.70E-07
RknMes02 058141	AT3G06720.2	0	cassava4.1 032699	importin alpha isoform 1	4.941	1.79E-09	1.01E-06
RknMes02 055141	AT2G40210.1	5.00E-33	cassava4.1 027752	AGAMOUS-like 48	4.914	2.60E-09	1.18E-06
RknMes02 048392	AT2G38940.1	0	cassava4.1 005715	phosphate transporter 1;7///Unknown///phosphate transporter 1;5///phosp	4.837	6.58E-08	5.87E-06
RknMes02 058688	AT3G28880.1	0	cassava4.1 033616	Ankyrin repeat family protein	4.808	2.98E-10	4.22E-07
RknMes02 055845	AT3G51880.4	6.00E-16	cassava4.1 028951	high mobility group B2///high mobility group B1	4.757	3.24E-07	1.37E-05
RknMes02 058115	AT3G07390.1	0	cassava4.1 032657	auxin-responsive family protein///Auxin-responsive family protein	4.728	1.10E-06	2.84E-05
RknMes02 001781	AT3G58110.2	3.00E-28	cassava4.1 004503	unknown protein; FUNCTIONS IN: molecular function unknown; INVO	4.715	3.51E-09	1.34E-06
RknMes02 052316	AT4G01950.1	0	cassava4.1 022947	glycerol-3-phosphate acyltransferase 3	4.700	1.28E-07	8.32E-06
RknMes02 001711	AT4G32460.2	1.00E-19	cassava4.1 010077	Protein of unknown function, DUF642	4.685	2.31E-09	1.13E-06
RknMes02 057300	AT2G13810.1	0	cassava4.1 031362	AGD2-like defense response protein 1	4.524	2.31E-09	1.13E-06
RknMes02 055925	AT4G34135.1	0	cassava4.1 029076	UDP-glucosyltransferase 73B2///UDP-glucosyl transferase 73B3	4.435	7.19E-09	1.80E-06
RknMes02 054888	AT1G01690.1	0	cassava4.1 027325	putative recombination initiation defects 3	4.407	6.03E-08	5.59E-06
RknMes02 050833	AT4G39250.1	2.00E-32	cassava4.1 020375	RAD-like 1///RAD-like 6	4.361	4.47E-09	1.48E-06
RknMes02_039148			cassava4.1_002717m		4.350	2.31E-09	1.13E-06
RknMes02 013200	AT4G34131.1	3.00E-08	cassava4.1 029076	UDP-glucosyl transferase 73B3	4.301	2.09E-08	3.27E-06
RknMes02 055406	AT5G17350.1	4.00E-27	cassava4.1 028188	AT5G17350.1///AT3G03280.1	4.299	9.10E-12	1.08E-07
RknMes02 057295	AT4G37770.1	0	cassava4.1 031356	1-amino-cyclopropane-1-carboxylate synthase 8	4.083	2.55E-07	1.20E-05
RknMes02 001575	AT4G25640.2	6.00E-30	cassava4.1 006392	detoxifying efflux carrier 35	4.064	3.21E-07	1.37E-05
RknMes02 057627	AT4G32480.1	0	cassava4.1 031890	Protein of unknown function (DUF506)	4.056	4.63E-07	1.66E-05
RknMes02 058010	AT5G07610.1	1.00E-16	cassava4.1 032488	F-box family protein	4.005	1.18E-07	7.95E-06
RknMes02_011864			cassava4.1_029076m		3.971	2.23E-08	3.36E-06
RknMes02 057486	AT2G21220.1	1.00E-35	cassava4.1 031669	SAUR-like auxin-responsive protein family	3.878	1.51E-10	2.98E-07
RknMes02 024608	AT3G04620.1	0	cassava4.1 023523	Alba DNA/RNA-binding protein	3.876	1.13E-06	2.89E-05
RknMes02 055721	AT4G39250.1	3.00E-36	cassava4.1 028739	RAD-like 1///RAD-like 6	3.860	9.54E-10	7.62E-07
RknMes02 054063	AT1G14440.2	2.80E-45	cassava4.1 025934	homeobox protein 33///homeobox protein 31	3.836	4.87E-06	7.51E-05
RknMes02 051551	AT2G33480.2	5.00E-10	cassava4.1 021626	NAC domain containing protein 52	3.799	2.96E-08	3.89E-06
RknMes02 057805	AT3G24060.1	1.00E-13	cassava4.1 032165	Plant self-incompatibility protein S1 family	3.776	3.68E-07	1.47E-05
RknMes02 048226	AT3G16380.1	0	cassava4.1 004058	poly(A) binding protein 6	3.775	1.86E-07	1.01E-05
RknMes02 055397	AT2G26140.1	0	cassava4.1 028173	FTSH protease 4	3.745	9.44E-08	7.16E-06
RknMes02 001499	AT5G54160.1	1.00E-21	cassava4.1 013376	O-methyltransferase 1	3.697	6.28E-06	8.90E-05
RknMes02 004432	AT5G11420.1	2.00E-23	cassava4.1 010027	Protein of unknown function, DUF642	3.646	5.92E-09	1.65E-06
RknMes02 053062	AT1G68390.1	0	cassava4.1 024214	Core-2/I-branching beta-1,6-N-acetylglucosaminyltransferase family prot	3.612	7.05E-10	6.51E-07
RknMes02 050655	AT3G04710.3	1.00E-14	cassava4.1 019864	ankyrin repeat family protein	3.533	6.33E-08	5.74E-06
RknMes02 056068	AT3G28880.1	3.00E-30	cassava4.1 029312	Ankyrin repeat family protein	3.501	1.20E-06	3.00E-05
RknMes02 050561	AT3G04620.1	2.00E-33	cassava4.1 019564	Alba DNA/RNA-binding protein	3.498	1.61E-07	9.31E-06
RknMes02 022851	AT3G47800.1	0	cassava4.1 025611	Galactose mutarotase-like superfamily protein	3.424	2.62E-09	1.18E-06
RknMes02 026790	AT2G26870.1	0	cassava4.1 005538	non-specific phospholipase C2	3.386	4.07E-12	6.01E-08
RknMes02 051740	AT1G54115.1	0	cassava4.1 021941	cation calcium exchanger 4	3.375	6.01E-09	1.66E-06
RknMes02 054668	AT4G36740.1	0	cassava4.1 026937	homeobox protein 40///homeobox protein 21	3.366	4.30E-09	1.46E-06
RknMes02 053782	AT4G17380.1	0	cassava4.1 025428	MUTS-like protein 4	3.333	3.11E-07	1.34E-05
RknMes02 057539	AT5G07610.1	3.00E-08	cassava4.1 031747	F-box family protein	3.303	7.51E-11	2.44E-07
RknMes02 052163	AT2G22840.1	0	cassava4.1 022687	growth-regulating factor 1	3.294	1.70E-06	3.76E-05
RknMes02 054016	AT5G42120.1	0	cassava4.1 025847	Concanavalin A-like lectin protein kinase family protein	3.244	3.96E-07	1.52E-05
RknMes02 000416	AT2G26870.1	0	cassava4.1 005538	non-specific phospholipase C2	3.200	1.37E-11	1.20E-07
RknMes02 055230	AT3G05950.1	0	cassava4.1 027899	RmlC-like cupins superfamily protein	3.166	5.15E-06	7.80E-05
RknMes02 000277	AT4G25640.2	0	cassava4.1 006392	detoxifying efflux carrier 35	3.149	1.34E-06	3.23E-05
RknMes02_003748			cassava4.1_017297m		3.144	5.35E-09	1.59E-06
RknMes02 052114	AT3G14670.2	3.00E-06	cassava4.1 022594	cassava4.1_022594m	3.138	1.41E-06	3.34E-05
RknMes02 028787	AT1G19640.1	2.00E-12	cassava4.1 010155	S-adenosyl-L-methionine-dependent methyltransferases superfamily prot	3.117	3.04E-08	3.94E-06
RknMes02 051840	AT3G09280.1	1.00E-12	cassava4.1 022123	AT3G09280.1///Unknown	3.094	9.67E-07	2.61E-05
RknMes02 051464	AT3G06240.1	1.00E-24	cassava4.1 021488	F-box family protein///F-box and associated interaction domains-containi	3.000	1.78E-08	2.95E-06
RknMes02 012739	AT2G18550.1	3.00E-32	cassava4.1 027319	homeobox protein 21	2.951	1.32E-08	2.53E-06
RknMes02 048720	AT1G03790.1	0	cassava4.1 008973	Zinc finger C-x8-C-x5-C-x3-H type family protein	2.912	4.36E-08	4.76E-06
RknMes02 056640	AT3G11930.1	0	cassava4.1 030279	Adenine nucleotide alpha hydrolases-like superfamily protein	2.902	2.04E-08	3.24E-06
RknMes02 054342	AT2G28420.1	0	cassava4.1 026393	Lactoylglutathione lyase / glyoxalase I family protein	2.830	6.03E-07	1.92E-05
RknMes02_007372			cassava4.1_011919m		2.829	1.34E-06	3.23E-05
RknMes02 022677	AT4G25040.1	4.00E-12	cassava4.1 017297	Uncharacterised protein family (UPF0497)///Unknown	2.787	2.33E-10	3.82E-07
RknMes02 039445	AT2G41380.1	0	cassava4.1 014142	S-adenosyl-L-methionine-dependent methyltransferases superfamily prot	2.764	3.28E-08	4.13E-06
RknMes02_046039					2.745	4.59E-06	7.20E-05
RknMes02 025204	AT1G09960.1	4.00E-33	cassava4.1 007925	Unknown///sucrose transporter 4	2.739	6.83E-06	9.43E-05
RknMes02 034590	AT1G10340.2	4.00E-18	cassava4.1 004232	Ankyrin repeat family protein	2.726	2.05E-07	1.06E-05
RknMes02_035809			cassava4.1_012760m		2.717	1.27E-08	2.45E-06
RknMes02 057031	AT5G01260.2	1.00E-25	cassava4.1 030934	Carbohydrate-binding-like fold	2.715	2.94E-06	5.33E-05
RknMes02 009054	AT4G35590.1	4.00E-31	cassava4.1 024901	NIN like protein 7///RWP-RK domain-containing protein	2.707	1.94E-08	3.14E-06
RknMes02 051856	AT1G35910.1	0	cassava4.1 022149	Haloacid dehalogenase-like hydrolase (HAD) superfamily protein///treha	2.705	2.03E-06	4.21E-05
RknMes02 058825	AT1G33760.1	0	cassava4.1 033833	Integrase-type DNA-binding superfamily protein	2.683	2.49E-06	4.79E-05
RknMes02_047242					2.643	1.41E-06	3.33E-05
RknMes02 055022	AT4G33870.1	0	cassava4.1 027550	Peroxidase superfamily protein	2.638	2.83E-09	1.18E-06
RknMes02 056535	AT5G53980.1	9.00E-34	cassava4.1 030094	homeobox protein 52	2.619	3.01E-08	3.92E-06
RknMes02 057698	AT2G45400.1	0	cassava4.1 032002	NAD(P)-binding Rossmann-fold superfamily protein	2.618	2.17E-08	3.32E-06
RknMes02_006016			cassava4.1_015713m		2.614	1.47E-07	9.03E-06
RknMes02_056495			cassava4.1_030018m		2.614	2.35E-06	4.64E-05
RknMes02_023054			cassava4.1_021277m		2.611	4.39E-06	6.98E-05
RknMes02 056633	AT1G29050.1	0	cassava4.1 030267	TRICHOME BIREFRINGENCE-LIKE 38	2.606	1.64E-07	9.40E-06
RknMes02 011799	AT2G28090.1	2.00E-13	cassava4.1 011919	Heavy metal transport/detoxification superfamily protein	2.579	2.36E-06	4.66E-05
RknMes02 007011	AT3G01470.1	9.00E-31	cassava4.1 012760	homeobox 1	2.565	1.46E-08	2.64E-06
RknMes02 053638	AT1G19780.1	0	cassava4.1 025182	cyclic nucleotide gated channel 8	2.549	6.34E-06	8.95E-05
RknMes02 054294	AT1G67810.1	0	cassava4.1 026316	sulfur E2	2.548	1.28E-09	8.44E-07
RknMes02_043801					2.532	4.32E-08	4.73E-06
RknMes02 037654	AT4G29110.1	1.00E-07	cassava4.1 016277	unknown protein; FUNCTIONS IN: molecular function unknown; INVO	2.527	9.47E-08	7.18E-06
RknMes02 051165	AT2G36020.1	7.00E-25	cassava4.1 021018	HVA22-like protein J	2.523	3.48E-06	5.98E-05
RknMes02 053434	AT4G22600.1	0	cassava4.1 024833	AT4G22600.1	2.518	3.63E-07	1.46E-05
RknMes02 032596	AT3G56400.1	2.00E-42	cassava4.1 013417	WRKY DNA-binding protein 70	2.500	4.11E-07	1.56E-05
RknMes02 057007	AT5G57620.1	0	cassava4.1 030900	myb domain protein 36	2.488	1.46E-06	3.40E-05
RknMes02_015109			cassava4.1_017002m		2.482	4.00E-09	1.42E-06
RknMes02_044414					2.472	1.08E-06	2.80E-05
RknMes02 019058	AT1G80050.1	3.00E-18		adenine phosphoribosyl transferase 2	2.458	2.43E-08	3.44E-06
RknMes02 032811	AT4G25640.2	0	cassava4.1 006392	detoxifying efflux carrier 35	2.457	2.72E-07	1.24E-05
RknMes02_002071			cassava4.1_017002m		2.447	5.51E-09	1.60E-06
RknMes02_002293					2.444	7.68E-07	2.27E-05
RknMes02 050000	AT4G15630.1	0	cassava4.1 016898	Uncharacterised protein family (UPF0497)	2.442	4.30E-06	6.89E-05
RknMes02_003930			cassava4.1_031768m		2.433	8.23E-07	2.37E-05
RknMes02 035498	AT2G40080.1	8.00E-28	cassava4.1 019555	Protein of unknown function (DUF1313)	2.431	6.24E-08	5.71E-06
RknMes02 051638	AT4G22660.1	2.00E-10	cassava4.1 021777	F-box family protein with a domain of unknown function (DUF295)	2.427	3.02E-06	5.43E-05
RknMes02 050020	AT5G51160.1	2.00E-19	cassava4.1 017002	Ankyrin repeat family protein	2.417	2.14E-09	1.12E-06
RknMes02 057548	AT3G50770.1	2.00E-29	cassava4.1 031768	calmodulin-like 41	2.415	2.56E-06	4.87E-05
RknMes02_023448			cassava4.1_009880m		2.410	2.50E-06	4.80E-05
RknMes02 053766	AT4G33870.1	0	cassava4.1 025400	Peroxidase superfamily protein	2.395	5.08E-07	1.74E-05
RknMes02_047468					2.369	5.83E-07	1.88E-05
RknMes02 051562	AT5G64360.4	2.00E-13	cassava4.1 021648	Chaperone DnaJ-domain superfamily protein	2.353	2.89E-12	5.97E-08
RknMes02 012269	AT1G06460.1	2.00E-10	cassava4.1 015713	alpha-crystallin domain 32.1	2.341	4.63E-06	7.23E-05
RknMes02 055451	AT4G15630.1	0	cassava4.1 028267	Uncharacterised protein family (UPF0497)	2.338	1.04E-06	2.74E-05
RknMes02 051803	AT3G09270.1	3.00E-11	cassava4.1 022056	glutathione S-transferase TAU 8///glutathione S-transferase tau 7	2.334	2.91E-07	1.29E-05
RknMes02 052318	AT1G65980.1	0	cassava4.1 022949	thioredoxin-dependent peroxidase 1	2.331	2.58E-08	3.58E-06
RknMes02 049317	AT2G16050.1	2.90E-44	cassava4.1 013278	Cysteine/Histidine-rich C1 domain family protein	2.304	9.60E-07	2.60E-05
RknMes02 057133	AT2G18460.1	0	cassava4.1 031089	like COV 3	2.291	7.93E-10	6.97E-07
RknMes02 033982	AT3G54820.1	0	cassava4.1 011438	plasma membrane intrinsic protein 2;5	2.238	1.23E-08	2.42E-06
RknMes02 058917	AT1G23550.1	0	cassava4.1 033992	similar to RCD one 2	2.227	5.41E-06	8.05E-05
RknMes02 022193	AT1G67810.1	1.00E-23	cassava4.1 014023	sulfur E2	2.217	2.00E-07	1.05E-05
RknMes02 036024	AT5G64750.1	5.00E-38	cassava4.1 007311	ethylene response factor 110///Unknown///Integrase-type DNA-binding s	2.215	3.12E-06	5.55E-05
RknMes02 007402	AT4G37870.1	0	cassava4.1 030131	phosphoenolpyruvate carboxykinase 1	2.165	4.74E-08	4.92E-06
RknMes02 057836	AT1G17020.1	0	cassava4.1 032207	2-oxoglutarate (2OG) and Fe(II)-dependent oxygenase superfamily protei	2.157	7.69E-09	1.85E-06
RknMes02 019176	AT4G01580.1	2.00E-17	cassava4.1 013238	AP2/B3-like transcriptional factor family protein	2.157	1.82E-06	3.93E-05
RknMes02 053760	AT3G20475.1	0	cassava4.1 025390	MUTS-homologue 5	2.153	2.55E-10	3.87E-07
RknMes02 053618	AT5G08460.1	0	cassava4.1 025139	GDSL-like Lipase/Acylhydrolase superfamily protein	2.145	4.71E-08	4.92E-06
RknMes02 052689	AT5G16080.1	0	cassava4.1 023579	carboxyesterase 17	2.143	1.59E-06	3.58E-05
RknMes02 030435	AT4G37870.1	0	cassava4.1 033411	phosphoenolpyruvate carboxykinase 1	2.141	1.39E-08	2.60E-06
RknMes02 058899	AT1G02400.1	0	cassava4.1 033960	gibberellin 2-oxidase 6	2.132	8.04E-07	2.33E-05
RknMes02 000313	AT5G33370.1	3.00E-43		GDSL-like Lipase/Acylhydrolase superfamily protein	2.111	1.60E-06	3.61E-05
RknMes02_038567					2.106	1.08E-06	2.80E-05

RknMes02 004259	AT4G37870.1	9.00E-23	cassava4.1 033411	phosphoenolpyruvate carboxykinase 1	2.094	2.39E-09	1.15E-06
RknMes02 052664	AT5G64310.1	9.00E-15	cassava4.1 023538	Unknown///arabinogalactan protein 1///cassava4.1_023538m	2.093	2.67E-06	4.99E-05
RknMes02_034435			cassava4.1_011089m		2.083	3.14E-06	5.58E-05
RknMes02 057556	AT4G02170.1	2.00E-19	cassava4.1 031780	AT4G02170.1	2.081	5.91E-06	8.54E-05
RknMes02_049508			cassava4.1_014414m		2.078	5.77E-08	5.45E-06
RknMes02 051622	AT2G21790.1	0	cassava4.1 021751	ribonucleotide reductase 1	2.071	3.61E-08	4.32E-06
RknMes02 037315	AT4G00330.1	4.00E-18	cassava4.1 032262	calmodulin-binding receptor-like cytoplasmic kinase 2	2.042	8.00E-09	1.88E-06
RknMes02_039767			cassava4.1_008879m		2.035	4.71E-07	1.67E-05
RknMes02 006356	AT3G28510.1	0	cassava4.1 005875	P-loop containing nucleoside triphosphate hydrolases superfamily protei	2.027	4.33E-06	6.93E-05
RknMes02 058174	AT2G44990.1	0	cassava4.1 032749	carotenoid cleavage dioxygenase 7	2.022	1.78E-07	9.87E-06
RknMes02 032390	AT3G21760.1	0	cassava4.1 031478	UDP-Glycosyltransferase superfamily protein///UDP-glucosyl transferase	2.002	1.67E-06	3.72E-05
RknMes02 055143	AT5G39820.1	6.00E-12	cassava4.1 027756	NAC-like, activated by AP3/PI	2.002	2.02E-06	4.21E-05
RknMes02 039442	AT5G55560.1	0	cassava4.1 012772	Protein kinase superfamily protein	1.997	2.41E-08	3.43E-06
RknMes02 052720	AT1G25220.1	0	cassava4.1 023625	anthranilate synthase beta subunit 1	1.955	1.43E-06	3.36E-05
RknMes02 030263	AT1G80050.1	0	cassava4.1 016994	adenine phosphoribosyl transferase 2	1.943	1.52E-09	9.44E-07
RknMes02 022940	AT4G20970.1	2.00E-12	cassava4.1 024041	basic helix-loop-helix (bHLH) DNA-binding superfamily protein	1.940	1.22E-07	8.07E-06
RknMes02 004093	AT3G10340.1	3.00E-12	cassava4.1 003117	phenylalanine ammonia-lyase 2///phenylalanine ammonia-lyase 4	1.937	2.55E-06	4.86E-05
RknMes02 006435	AT5G65690.1	1.00E-32	cassava4.1 030131	phosphoenolpyruvate carboxykinase 2	1.936	7.84E-08	6.40E-06
RknMes02 032464	AT4G05070.1	8.00E-07		Unknown	1.929	2.83E-06	5.20E-05
RknMes02 040157	AT5G65690.1	0	cassava4.1 030131	phosphoenolpyruvate carboxykinase 2	1.927	3.50E-08	4.27E-06
RknMes02 003389	AT4G15560.1	4.00E-39	cassava4.1 030091	Deoxyxylulose-5-phosphate synthase	1.924	5.53E-09	1.60E-06
RknMes02_042934					1.923	2.20E-06	4.45E-05
RknMes02 034595	AT1G34050.1	1.00E-34	cassava4.1 004232	Ankyrin repeat family protein	1.906	1.11E-06	2.85E-05
RknMes02 049538	AT1G74650.1	0	cassava4.1 014624	myb domain protein 31///myb domain protein 96	1.903	1.26E-08	2.44E-06
RknMes02 014452	AT3G05500.1	1.00E-31	cassava4.1 028020	Rubber elongation factor protein (REF)	1.873	8.41E-11	2.44E-07
RknMes02 020142	AT3G60670.1	0	cassava4.1 014925	PLATZ transcription factor family protein	1.873	1.97E-07	1.04E-05
RknMes02 017956	AT5G14230.1	0	cassava4.1 002222	AT5G14230.1///"CONTAINS InterPro DOMAIN/s: Ankyrin repeat-conta	1.861	2.48E-06	4.78E-05
RknMes02 034530	AT4G23810.1	8.00E-35	cassava4.1 010768	WRKY family transcription factor///WRKY DNA-binding protein 30	1.860	2.97E-06	5.36E-05
RknMes02 026274	AT5G40390.1	0	cassava4.1 002019	Raffinose synthase family protein	1.856	2.13E-08	3.30E-06
RknMes02 013101	AT1G69200.1	3.00E-06	cassava4.1 008596	Unknown///fructokinase-like 2	1.847	1.80E-06	3.91E-05
RknMes02 002136	AT5G22870.1	6.00E-07	cassava4.1 017573	Late embryogenesis abundant (LEA) hydroxyproline-rich glycoprotein fa	1.841	3.79E-08	4.44E-06
RknMes02 058273	AT1G35910.1	0	cassava4.1 032922	Haloacid dehalogenase-like hydrolase (HAD) superfamily protein///treha	1.840	8.57E-07	2.43E-05
RknMes02 055668	AT5G55850.3	3.00E-19	cassava4.1 028651	RPM1-interacting protein 4 (RIN4) family protein	1.819	1.15E-07	7.87E-06
RknMes02 050135	AT3G52460.1	6.00E-19	cassava4.1 017573	Unknown///hydroxyproline-rich glycoprotein family protein	1.814	2.61E-09	1.18E-06
RknMes02 051345	AT4G05120.1	0	cassava4.1 021308	Major facilitator superfamily protein	1.808	5.92E-06	8.55E-05
RknMes02 025998	AT2G18193.1	3.00E-28	cassava4.1 009105	P-loop containing nucleoside triphosphate hydrolases superfamily protei	1.795	2.15E-06	4.38E-05
RknMes02 053794	AT3G06720.2	0	cassava4.1 025451	importin alpha isoform 1	1.792	1.28E-06	3.14E-05
RknMes02 022969	AT5G24900.1	3.00E-34	cassava4.1 006808	cytochrome P450, family 714, subfamily A, polypeptide 2	1.778	7.08E-07	2.15E-05
RknMes02 037016	AT3G26300.1	0	cassava4.1 005635	cytochrome P450, family 71, subfamily B, polypeptide 34///"cytochrome	1.767	2.30E-06	4.58E-05
RknMes02 029441	AT5G24910.1	0	cassava4.1 005649	cytochrome P450, family 714, subfamily A, polypeptide 1	1.749	2.05E-07	1.07E-05
RknMes02 017072	AT3G10340.1	0	cassava4.1 003117	phenylalanine ammonia-lyase 2///phenylalanine ammonia-lyase 4	1.748	1.43E-06	3.35E-05
RknMes02 016408	AT4G36470.1	6.00E-09	cassava4.1 010155	S-adenosyl-L-methionine-dependent methyltransferases superfamily prot	1.742	1.47E-07	9.03E-06
RknMes02 048688	AT5G07440.2	0	cassava4.1 008693	glutamate dehydrogenase 2	1.737	2.40E-06	4.70E-05
RknMes02 049529	AT3G54420.1	0	cassava4.1 014554	homolog of carrot EP3-3 chitinase	1.733	1.95E-06	4.12E-05
RknMes02 056066	AT3G06600.2	2.00E-07	cassava4.1 029310	cassava4.1_029310m	1.712	2.45E-06	4.76E-05
RknMes02_045593					1.687	1.82E-07	9.95E-06
RknMes02 028371	AT4G01870.1	0	cassava4.1 034137	tolB protein-related	1.686	4.85E-06	7.50E-05
RknMes02 056065	AT3G22830.1	0	cassava4.1 029306	heat shock transcription factor A7A///heat shock transcription factor A6	1.676	1.13E-06	2.88E-05
RknMes02 023973	AT5G07440.3	4.00E-28	cassava4.1 008693	glutamate dehydrogenase 2	1.671	3.49E-07	1.42E-05
RknMes02 009426	AT5G14860.1	2.90E-44	cassava4.1 022533	UDP-Glycosyltransferase superfamily protein	1.668	3.24E-06	5.69E-05
RknMes02 025971	AT1G43910.1	0	cassava4.1 006163	P-loop containing nucleoside triphosphate hydrolases superfamily protei	1.654	2.05E-06	4.24E-05
RknMes02 052194	AT4G24970.1	0	cassava4.1 022738	Histidine kinase-, DNA gyrase B-, and HSP90-like ATPase family protei	1.653	4.19E-08	4.63E-06
RknMes02 050157	AT4G05430.1	2.00E-33	cassava4.1 017684	Carbohydrate-binding X8 domain superfamily protein	1.640	5.34E-06	7.98E-05
RknMes02 056248	AT4G39230.1	0	cassava4.1 029598	NmrA-like negative transcriptional regulator family protein	1.629	1.25E-07	8.20E-06
RknMes02 011304	AT1G43910.1	5.00E-22	cassava4.1 006163	P-loop containing nucleoside triphosphate hydrolases superfamily protei	1.624	1.17E-06	2.95E-05
RknMes02 031261	AT4G27410.2	0	cassava4.1 010999	NAC (No Apical Meristem) domain transcriptional regulator superfamily	1.618	1.87E-07	1.01E-05
RknMes02 004082	AT1G17840.1	1.00E-17	cassava4.1 005391	white-brown complex homolog protein 11	1.617	5.04E-07	1.73E-05
RknMes02_057376			cassava4.1_031490m		1.616	1.06E-07	7.53E-06
RknMes02 039581	AT5G24910.1	0	cassava4.1 005649	cytochrome P450, family 714, subfamily A, polypeptide 1	1.610	2.84E-07	1.27E-05
RknMes02 052535	AT3G09270.1	0	cassava4.1 023315	glutathione S-transferase TAU 8///glutathione S-transferase tau 7	1.602	4.47E-07	1.63E-05
RknMes02 027276	AT1G52980.1	8.00E-30	cassava4.1 019581	GTP-binding family protein	1.597	4.51E-09	1.48E-06
RknMes02 052347	AT3G51810.1	1.00E-38	cassava4.1 022993	Stress induced protein	1.586	4.54E-06	7.15E-05
RknMes02 005864	AT5G02230.2	7.00E-08	cassava4.1 014451	Haloacid dehalogenase-like hydrolase (HAD) superfamily protein	1.584	1.52E-06	3.49E-05
RknMes02 009571	AT3G09550.1	8.00E-43	cassava4.1 007548	Ankyrin repeat family protein	1.584	1.16E-06	2.93E-05
RknMes02 053679	AT2G35950.1	5.00E-07	cassava4.1 025248	embryo sac development arrest 12	1.582	6.08E-06	8.71E-05
RknMes02 029383	AT2G41190.1	0	cassava4.1 007924	Transmembrane amino acid transporter family protein	1.580	3.41E-06	5.90E-05
RknMes02 035114	AT1G06320.1	4.00E-16	cassava4.1 017107	unknown protein; Has 24 Blast hits to 24 proteins in 10 species: Archae -	1.579	4.09E-06	6.68E-05
RknMes02 023158	AT5G02230.2	8.00E-31	cassava4.1 013061	Haloacid dehalogenase-like hydrolase (HAD) superfamily protein	1.578	2.38E-06	4.68E-05
RknMes02 055923	AT3G55350.1	0	cassava4.1 029072	PIF / Ping-Pong family of plant transposases	1.565	6.17E-08	5.67E-06
RknMes02 005365	AT2G37980.1	1.00E-14	cassava4.1 030087	O-fucosyltransferase family protein	1.551	2.09E-07	1.07E-05
RknMes02 012991	AT5G07440.3	7.00E-43	cassava4.1 008701	glutamate dehydrogenase 2	1.550	5.19E-06	7.84E-05
RknMes02 051666	AT4G02570.4	0	cassava4.1 021824	cullin 1	1.549	2.84E-06	5.21E-05
RknMes02 008572	AT4G15417.1	3.00E-37	cassava4.1 031224	RNAse II-like 1	1.533	7.85E-08	6.40E-06
RknMes02 034114	AT5G26667.3	7.00E-27	cassava4.1 016091	P-loop containing nucleoside triphosphate hydrolases superfamily protei	1.529	2.46E-07	1.18E-05
RknMes02_007714			cassava4.1_022173m		1.524	2.18E-07	1.10E-05
RknMes02 057388	AT2G17570.1	0	cassava4.1 031506	Undecaprenyl pyrophosphate synthetase family protein	1.522	1.11E-07	7.77E-06
RknMes02 003139	AT5G02790.1	3.00E-41	cassava4.1 015004	Glutathione S-transferase family protein	1.516	7.40E-08	6.19E-06
RknMes02 048719	AT5G22300.1	0	cassava4.1 008964	nitrilase 4	1.514	8.03E-08	6.47E-06
RknMes02 051348	AT2G28930.3	0	cassava4.1 021311	protein kinase 1B	1.509	2.49E-06	4.80E-05
RknMes02 054990	AT2G26070.1	0	cassava4.1 027499	Protein of unknown function (DUF778)	1.505	6.62E-06	9.24E-05
RknMes02 010313	AT5G62790.2	4.00E-14	cassava4.1 006908	1-deoxy-D-xylulose 5-phosphate reductoisomerase	1.503	2.24E-09	1.12E-06
RknMes02_016203			cassava4.1_018507m		1.502	1.71E-08	2.91E-06
RknMes02 048519	AT3G21760.1	0	cassava4.1 007027	UDP-Glycosyltransferase superfamily protein///UDP-glucosyl transferase	1.496	6.93E-07	2.12E-05
RknMes02 049851	AT3G22160.1	2.00E-14	cassava4.1 016216	VQ motif-containing protein///Unknown	1.495	5.92E-06	8.55E-05
RknMes02 054777	AT4G12570.1	7.00E-29	cassava4.1 027126	ubiquitin protein ligase 5///MAPK/ERK kinase kinase 1	1.492	6.47E-07	2.02E-05
RknMes02 001717	AT5G08260.1	7.00E-15	cassava4.1 020819	serine carboxypeptidase-like 35	1.492	1.21E-06	3.02E-05
RknMes02 005143	AT2G29630.3	2.00E-17	cassava4.1 003359	thiaminC	1.489	9.74E-07	2.62E-05
RknMes02 010122	AT5G60800.2	7.00E-13	cassava4.1 010149	Unknown///Heavy metal transport/detoxification superfamily protein	1.489	1.53E-06	3.50E-05
RknMes02 048263	AT1G26610.1	5.00E-33	cassava4.1 004354	C2H2-like zinc finger protein	1.488	7.20E-06	9.77E-05
RknMes02 031179	AT5G60800.2	2.00E-34	cassava4.1 010149	Unknown///Heavy metal transport/detoxification superfamily protein	1.486	1.84E-06	3.97E-05
RknMes02 057524	AT1G65980.1	0	cassava4.1 031724	thioredoxin-dependent peroxidase 1	1.484	1.47E-07	9.03E-06
RknMes02 008115	AT1G67810.1	9.00E-35	cassava4.1 014023	sulfur E2	1.481	2.00E-06	4.18E-05
RknMes02 052829	AT4G02390.1	0	cassava4.1 023824	poly(ADP-ribose) polymerase	1.479	9.83E-09	2.13E-06
RknMes02 010887	AT2G43590.1	2.00E-12	cassava4.1 014554	Chitinase family protein	1.463	3.71E-06	6.27E-05
RknMes02 014511	AT4G02390.1	1.00E-11		poly(ADP-ribose) polymerase	1.459	5.80E-08	5.46E-06
RknMes02 031388	AT5G65780.1	0	cassava4.1 012023	branched-chain amino acid aminotransferase 5 / branched-chain amino a	1.458	2.55E-10	3.87E-07
RknMes02 023807	AT4G00330.1	6.00E-30	cassava4.1 031817	calmodulin-binding receptor-like cytoplasmic kinase 2	1.455	2.15E-07	1.09E-05
RknMes02 004885	AT2G14660.1	9.00E-37	cassava4.1 022246	unknown protein; CONTAINS InterPro DOMAIN/s: Uncharacterised pro	1.454	3.58E-07	1.45E-05
RknMes02 030292	AT5G08260.1	0	cassava4.1 010410	serine carboxypeptidase-like 35	1.451	3.52E-07	1.43E-05
RknMes02_035613			cassava4.1_011649m		1.446	3.12E-09	1.24E-06
RknMes02 007453	AT1G01320.2	0	cassava4.1 000135	Tetratricopeptide repeat (TPR)-like superfamily protein	1.445	3.61E-06	6.15E-05
RknMes02_001636			cassava4.1_007088m		1.444	1.67E-07	9.53E-06
RknMes02 051046	AT5G08260.1	1.00E-23	cassava4.1 020819	serine carboxypeptidase-like 35	1.439	3.75E-07	1.48E-05
RknMes02 011040	AT2G12190.1	6.00E-10	cassava4.1 032642	Cytochrome P450 superfamily protein	1.435	6.93E-06	9.52E-05
RknMes02_043823					1.425	2.05E-07	1.07E-05
RknMes02 003488	AT3G54140.1	4.00E-14	cassava4.1 024513	peptide transporter 1	1.424	1.99E-07	1.05E-05
RknMes02_015171			cassava4.1_020405m		1.420	6.33E-07	1.99E-05
RknMes02 010687	AT4G15560.1	6.00E-36	cassava4.1 030091	Deoxyxylulose-5-phosphate synthase	1.417	1.57E-10	2.99E-07
RknMes02 049514	AT5G02230.2	0	cassava4.1 014451	Haloacid dehalogenase-like hydrolase (HAD) superfamily protein	1.408	1.73E-06	3.81E-05
RknMes02 041567	AT2G04520.1	8.00E-33	cassava4.1 023644	Nucleic acid-binding, OB-fold-like protein	1.407	4.23E-06	6.83E-05
RknMes02 036843	AT2G30860.1	0	cassava4.1 016203	glutathione S-transferase PHI 9	1.403	2.52E-06	4.83E-05
RknMes02_042540					1.397	4.32E-07	1.60E-05
RknMes02 003788	AT2G23970.1	9.00E-30	cassava4.1 023112	Class I glutamine amidotransferase-like superfamily protein	1.391	3.68E-07	1.47E-05
RknMes02_047156			cassava4.1_029851m		1.389	1.32E-06	3.20E-05
RknMes02_019499					1.382	1.57E-07	9.25E-06
RknMes02 013976	AT1G59740.1	4.00E-12	cassava4.1 003848	Major facilitator superfamily protein	1.379	5.49E-06	8.12E-05
RknMes02 052026	AT3G57230.1	6.00E-28	cassava4.1 022444	AGAMOUS-like 16	1.379	2.81E-06	5.17E-05
RknMes02 054233	AT5G42200.1	2.00E-33	cassava4.1 026205	RING/U-box superfamily protein	1.378	4.88E-08	4.97E-06
RknMes02 037825	AT2G01290.1	0	cassava4.1 014596	ribose-5-phosphate isomerase 2	1.374	6.20E-07	1.96E-05
RknMes02 048321	AT1G07180.1	0	cassava4.1 004993	alternative NAD(P)H dehydrogenase 2///alternative NAD(P)H dehydrog	1.364	5.50E-07	1.82E-05
RknMes02 004319	AT1G17680.2	8.00E-15	cassava4.1 025348	tetratricopeptide repeat (TPR)-containing protein	1.349	6.47E-09	1.70E-06
RknMes02 019566	AT4G27450.1	1.00E-39	cassava4.1 014315	Aluminium induced protein with YGL and LRDR motifs	1.349	1.35E-06	3.25E-05
RknMes02 037144	AT3G45970.1	6.00E-17	cassava4.1 015495	expansin-like A1	1.347	1.94E-06	4.11E-05
RknMes02 039694	AT3G03341.1	2.00E-26	cassava4.1 020659	unknown protein; Has 30201 Blast hits to 17322 proteins in 780 species:	1.341	7.35E-08	6.19E-06

RknMes02 011302	AT1G72280.1	1.00E-10	cassava4.1 007206	endoplasmic reticulum oxidoreductins 1	1.333	5.07E-06	7.72E-05
RknMes02 048405	AT2G26150.1	0	cassava4.1 005870	heat shock transcription factor A2	1.330	2.12E-06	4.34E-05
RknMes02 031868	AT1G59740.1	0	cassava4.1 003848	Major facilitator superfamily protein	1.325	4.13E-06	6.72E-05
RknMes02 055219	AT3G01600.1	0	cassava4.1 027882	NAC (No Apical Meristem) domain transcriptional regulator superfamily	1.321	1.16E-08	2.33E-06
RknMes02 058121	AT4G28780.1	0	cassava4.1 032666	GDSL-like Lipase/Acylhydrolase superfamily protein	1.316	2.92E-06	5.30E-05
RknMes02_047584					1.314	1.43E-06	3.35E-05
RknMes02_044477					1.310	1.15E-06	2.92E-05
RknMes02 006731	AT3G48000.1	0	cassava4.1 005092	aldehyde dehydrogenase 2B4	1.310	2.81E-06	5.17E-05
RknMes02 031931	AT4G01870.1	0	cassava4.1 034137	tolB protein-related	1.308	4.83E-07	1.69E-05
RknMes02 002656	AT5G42010.1	8.00E-09	cassava4.1 020893	Transducin/WD40 repeat-like superfamily protein	1.300	1.58E-06	3.57E-05
RknMes02 006698	AT4G21920.1	4.00E-08	cassava4.1 030675	unknown protein; FUNCTIONS IN: molecular function unknown; INVO	1.295	4.51E-06	7.12E-05
RknMes02_013801			cassava4.1_007311m		1.293	2.33E-06	4.62E-05
RknMes02 028518	AT5G05690.2	0	cassava4.1 006864	Cytochrome P450 superfamily protein	1.292	1.64E-08	2.84E-06
RknMes02 040089	AT5G07680.2	2.00E-30	cassava4.1 010869	NAC domain containing protein 80///NAC domain containing protein 10	1.291	5.68E-08	5.42E-06
RknMes02 056529	AT2G37980.1	0	cassava4.1 030087	O-fucosyltransferase family protein	1.288	1.36E-06	3.27E-05
RknMes02 000586	AT1G21850.1	3.00E-30	cassava4.1 005161	SKU5 similar 8	1.287	6.19E-06	8.82E-05
RknMes02 003150	AT5G08790.1	2.00E-20	cassava4.1 012943	NAC (No Apical Meristem) domain transcriptional regulator superfamily	1.285	1.46E-06	3.40E-05
RknMes02 032680	AT3G02470.4	9.00E-36	cassava4.1 010384	S-adenosylmethionine decarboxylase	1.275	1.77E-06	3.87E-05
RknMes02 031518	AT2G39700.1	0	cassava4.1 014440	expansin A9///expansin A4	1.275	5.77E-08	5.45E-06
RknMes02 040263	AT1G20270.1	2.00E-18	cassava4.1 013142	2-oxoglutarate (2OG) and Fe(II)-dependent oxygenase superfamily protei	1.274	6.29E-06	8.91E-05
RknMes02 049315	AT1G13990.1	0	cassava4.1 013257	AT1G13990.1///unknown protein; FUNCTIONS IN: molecular function	1.274	1.81E-07	9.93E-06
RknMes02_023528			cassava4.1_007913m		1.271	1.04E-06	2.74E-05
RknMes02 013142	AT3G48000.1	1.00E-14	cassava4.1 005092	aldehyde dehydrogenase 2B4	1.269	4.45E-08	4.82E-06
RknMes02 008877	AT4G31240.2	6.00E-06	cassava4.1 012358	protein kinase C-like zinc finger protein	1.266	1.14E-07	7.87E-06
RknMes02 037544	AT1G07180.1	1.00E-34	cassava4.1 005184	alternative NAD(P)H dehydrogenase 2///alternative NAD(P)H dehydrog	1.266	3.79E-07	1.49E-05
RknMes02 050293	AT5G20670.1	3.00E-38	cassava4.1 018327	Protein of unknown function (DUF1677)	1.255	5.52E-08	5.34E-06
RknMes02 016327	AT4G36470.1	7.00E-07	cassava4.1 010155	S-adenosyl-L-methionine-dependent methyltransferases superfamily prot	1.251	1.99E-06	4.16E-05
RknMes02 035642	AT1G65910.1	5.00E-14	cassava4.1 009209	NAC domain containing protein 28	1.241	9.65E-11	2.44E-07
RknMes02 004784	AT4G01870.1	0	cassava4.1 034137	tolB protein-related	1.237	3.80E-07	1.49E-05
RknMes02 001102	AT1G74650.1	1.00E-14	cassava4.1 012505	myb domain protein 31///myb domain protein 96	1.237	9.36E-07	2.56E-05
RknMes02_010628			cassava4.1_001759m		1.233	2.87E-10	4.22E-07
RknMes02_004348			cassava4.1_011742m		1.225	7.22E-08	6.16E-06
RknMes02 010961	AT5G09225.1	5.00E-13		unknown protein; FUNCTIONS IN: molecular function unknown; INVO	1.219	1.03E-07	7.46E-06
RknMes02 039276	AT5G06300.1	0	cassava4.1 013240	Putative lysine decarboxylase family protein	1.216	4.95E-06	7.58E-05
RknMes02 013312	AT1G31280.1	1.00E-13	cassava4.1 000920	Argonaute family protein	1.215	1.38E-07	8.73E-06
RknMes02 049176	AT3G18990.1	2.00E-28	cassava4.1 012400	AP2/B3-like transcriptional factor family protein	1.213	1.50E-07	9.06E-06
RknMes02 015088	AT1G13990.1	6.00E-18	cassava4.1 013257	AT1G13990.1///unknown protein; FUNCTIONS IN: molecular function	1.212	3.65E-07	1.46E-05
RknMes02 034016	AT4G12300.1	0	cassava4.1 005921	cytochrome P450, family 706, subfamily A, polypeptide 4	1.211	1.40E-06	3.33E-05
RknMes02 025563	AT4G37300.1	3.00E-17	cassava4.1 017664	maternal effect embryo arrest 59	1.210	1.16E-07	7.87E-06
RknMes02 052307	AT1G51080.1	0	cassava4.1 022931	AT1G51080.1	1.210	2.63E-06	4.95E-05
RknMes02 011696	AT3G07720.1	7.00E-35	cassava4.1 011729	Galactose oxidase/kelch repeat superfamily protein	1.210	5.73E-06	8.37E-05
RknMes02 037190	AT2G28680.1	5.00E-33	cassava4.1 010513	RmlC-like cupins superfamily protein	1.209	9.16E-09	2.07E-06
RknMes02 018428	AT3G47420.1	4.00E-11	cassava4.1 005536	phosphate starvation-induced gene 3	1.208	1.49E-07	9.04E-06
RknMes02 024133	AT5G08380.1	0	cassava4.1 011258	alpha-galactosidase 1	1.207	8.03E-09	1.88E-06
RknMes02 029530	AT2G23810.1	0	cassava4.1 013791	tetraspanin11///tetraspanin8///tetraspanin7	1.203	1.37E-06	3.29E-05
RknMes02_008482			cassava4.1_010513m		1.203	1.03E-07	7.43E-06
RknMes02 058926	AT2G29420.1	0	cassava4.1 034007	glutathione S-transferase tau 7///glutathione S-transferase TAU 1	1.199	1.14E-06	2.90E-05
RknMes02 049743	AT3G03330.1	0	cassava4.1 015717	NAD(P)-binding Rossmann-fold superfamily protein	1.198	2.10E-09	1.12E-06
RknMes02 025483	AT3G55350.1	1.00E-32	cassava4.1 021993	PIF / Ping-Pong family of plant transposases	1.197	9.83E-07	2.63E-05
RknMes02 024844	AT2G16230.1	0	cassava4.1 007302	O-Glycosyl hydrolases family 17 protein	1.196	2.10E-06	4.30E-05
RknMes02 040118	AT3G07130.1	0	cassava4.1 005206	purple acid phosphatase 15	1.195	4.95E-06	7.59E-05
RknMes02 001250	AT4G23060.1	3.00E-07		IQ-domain 22///Unknown	1.189	1.64E-07	9.38E-06
RknMes02 036388	AT2G44480.2	0	cassava4.1 005766	Glycosyl hydrolase superfamily protein///beta glucosidase 17	1.188	5.75E-07	1.87E-05
RknMes02 034375	AT1G21840.1	5.60E-45	cassava4.1 014923	urease accessory protein F	1.188	2.45E-07	1.17E-05
RknMes02 039417	AT3G07360.1	0	cassava4.1 032891	plant U-box 9	1.184	6.45E-06	9.08E-05
RknMes02 051166	AT5G59030.1	1.00E-42	cassava4.1 021023	Ctr copper transporter family///copper transporter 1	1.183	9.58E-07	2.60E-05
RknMes02 054089	AT1G65570.1	0	cassava4.1 025978	Pectin lyase-like superfamily protein	1.183	2.59E-06	4.90E-05
RknMes02 038586	AT1G03220.1	2.00E-28	cassava4.1 007945	Eukaryotic aspartyl protease family protein	1.181	2.13E-06	4.35E-05
RknMes02 036162	AT1G21790.1	0	cassava4.1 024644	TRAM, LAG1 and CLN8 (TLC) lipid-sensing domain containing protein	1.179	1.85E-09	1.04E-06
RknMes02 008825	AT2G36580.1	2.00E-12	cassava4.1 006316	Pyruvate kinase family protein	1.176	4.36E-07	1.61E-05
RknMes02 028376	AT2G44065.2	6.00E-39	cassava4.1 016396	Ribosomal protein L2 family///Unknown///ribosomal protein L2	1.175	2.66E-06	4.98E-05
RknMes02 009639	AT1G34300.1	5.00E-21	cassava4.1 001853	receptor-like protein kinase 1///S-domain-2 5///lectin protein kinase fami	1.174	4.18E-07	1.57E-05
RknMes02 033990	AT2G45120.1	0	cassava4.1 010237	C2H2-like zinc finger protein	1.169	8.09E-08	6.48E-06
RknMes02_031569			cassava4.1_011649m		1.168	3.66E-09	1.37E-06
RknMes02 035308	AT2G03470.2	3.00E-12	cassava4.1 013932	ELM2 domain-containing protein	1.164	9.17E-07	2.53E-05
RknMes02 011200	AT2G24520.1	0	cassava4.1 001102	H(+)-ATPase 5	1.162	3.96E-07	1.52E-05
RknMes02 026417	AT5G50330.1	0	cassava4.1 006600	Protein kinase superfamily protein	1.161	8.51E-08	6.74E-06
RknMes02 010508	AT1G19600.1	1.00E-24	cassava4.1 010130	pfkB-like carbohydrate kinase family protein	1.161	2.59E-06	4.91E-05
RknMes02 029688	AT5G49610.1	4.00E-12	cassava4.1 010646	F-box family protein	1.155	1.64E-06	3.66E-05
RknMes02 001879	AT1G65980.1	7.00E-35	cassava4.1 017973	thioredoxin-dependent peroxidase 1	1.153	3.63E-06	6.16E-05
RknMes02 047454	AT2G28680.1	4.00E-38	cassava4.1 010513	RmlC-like cupins superfamily protein	1.151	4.10E-08	4.57E-06
RknMes02 031531	AT5G24020.1	0	cassava4.1 011379	septum site-determining protein (MIND)	1.151	1.73E-08	2.92E-06
RknMes02 036309	AT2G01290.1	0	cassava4.1 014064	ribose-5-phosphate isomerase 2	1.150	4.10E-06	6.69E-05
RknMes02 025071	AT4G02940.1	2.00E-31	cassava4.1 004959	oxidoreductase, 2OG-Fe(II) oxygenase family protein	1.150	1.37E-08	2.57E-06
RknMes02 004828	AT4G33090.1	7.00E-31	cassava4.1 003844	aminopeptidase M1	1.149	1.71E-07	9.64E-06
RknMes02 000236	AT1G09950.1	1.00E-08	cassava4.1 033345	RESPONSE TO ABA AND SALT 1	1.148	1.86E-06	3.99E-05
RknMes02 004375	AT1G05010.1	6.00E-15	cassava4.1 012052	ethylene-forming enzyme	1.148	6.95E-06	9.54E-05
RknMes02_021431					1.147	3.48E-07	1.42E-05
RknMes02 008708	AT1G60420.1	1.00E-27	cassava4.1 004565	DC1 domain-containing protein	1.145	2.51E-06	4.82E-05
RknMes02 034684	AT1G23800.1	0	cassava4.1 005133	aldehyde dehydrogenase 2B7	1.144	4.36E-06	6.96E-05
RknMes02_024400					1.143	5.85E-07	1.88E-05
RknMes02 049955	AT2G35900.1	0	cassava4.1 016725	unknown protein; FUNCTIONS IN: molecular function unknown; INVO	1.142	2.48E-09	1.17E-06
RknMes02 056377	AT2G32460.2	0	cassava4.1 029822	myb domain protein 101	1.142	1.05E-06	2.76E-05
RknMes02 049458	AT2G23620.1	0	cassava4.1 014110	methyl esterase 1	1.142	2.52E-06	4.83E-05
RknMes02 020155	AT4G21120.1	0	cassava4.1 027926	amino acid transporter 1	1.142	3.12E-06	5.55E-05
RknMes02 054005	AT5G57560.1	0	cassava4.1 025832	Xyloglucan endotransglucosylase/hydrolase family protein///xyloglucan e	1.142	2.22E-09	1.12E-06
RknMes02 014958	AT1G55000.3	1.00E-13	cassava4.1 014166	peptidoglycan-binding LysM domain-containing protein	1.141	3.62E-06	6.15E-05
RknMes02 008108	AT1G04420.1	2.00E-32	cassava4.1 008972	NAD(P)-linked oxidoreductase superfamily protein	1.141	7.00E-08	6.05E-06
RknMes02 003522	AT5G02800.1	1.00E-39	cassava4.1 008502	Protein kinase superfamily protein	1.139	5.82E-07	1.88E-05
RknMes02 014510	AT3G02630.1	8.00E-27	cassava4.1 009202	Plant stearoyl-acyl-carrier-protein desaturase family protein	1.138	1.42E-11	1.20E-07
RknMes02 009542	AT1G55740.1	4.00E-15	cassava4.1 002249	seed imbibition 1	1.135	1.88E-06	4.01E-05
RknMes02 055886	AT4G26700.2	0	cassava4.1 029014	fimbrin 1	1.134	5.06E-06	7.71E-05
RknMes02 025404	AT1G67420.2	4.00E-18	cassava4.1 001482	Zn-dependent exopeptidases superfamily protein	1.133	2.41E-06	4.72E-05
RknMes02 054650	AT5G55850.3	9.00E-21	cassava4.1 026901	RPM1-interacting protein 4 (RIN4) family protein	1.132	3.21E-06	5.66E-05
RknMes02 031259	AT5G65280.1	0	cassava4.1 008284	GCR2-like 1	1.131	2.72E-07	1.24E-05
RknMes02 051084	AT5G24320.1	0	cassava4.1 020893	Transducin/WD40 repeat-like superfamily protein	1.130	2.47E-06	4.77E-05
RknMes02 036565	AT5G15450.1	1.00E-30	cassava4.1 001040	casein lytic proteinase B3	1.127	2.12E-09	1.12E-06
RknMes02 010484	AT5G57050.1	3.00E-11	cassava4.1 010060	Protein phosphatase 2C family protein	1.127	1.82E-06	3.93E-05
RknMes02 012429	AT1G12010.1	2.00E-08	cassava4.1 012052	2-oxoglutarate (2OG) and Fe(II)-dependent oxygenase superfamily protei	1.126	1.63E-06	3.66E-05
RknMes02 030183	AT3G52870.1	0	cassava4.1 014011	IQ calmodulin-binding motif family protein	1.126	1.78E-06	3.88E-05
RknMes02 031436	AT3G48700.1	0	cassava4.1 026915	carboxyesterase 13	1.122	1.41E-06	3.34E-05
RknMes02 052218	AT5G16850.1	0	cassava4.1 022790	telomerase reverse transcriptase	1.116	1.51E-06	3.46E-05
RknMes02 001279	AT5G02790.1	3.00E-11	cassava4.1 015004	Glutathione S-transferase family protein	1.113	2.97E-06	5.36E-05
RknMes02 028619	AT4G11820.2	9.00E-24	cassava4.1 007081	hydroxymethylglutaryl-CoA synthase / HMG-CoA synthase / 3-hydroxy-3	1.113	5.56E-06	8.19E-05
RknMes02 055949	AT5G08391.1	7.00E-27	cassava4.1 029115	cassava4.1_033061m///Protein of unknown function (DUF 3339)	1.113	4.45E-08	4.82E-06
RknMes02 035810	AT2G37220.1	0	cassava4.1 013463	RNA-binding (RRM/RBD/RNP motifs) family protein	1.110	2.68E-07	1.23E-05
RknMes02 040160	AT1G04280.1	7.00E-24	cassava4.1 005863	P-loop containing nucleoside triphosphate hydrolases superfamily protein	1.108	1.90E-07	1.02E-05
RknMes02 001446	AT2G47800.1	3.00E-32	cassava4.1 000205	multidrug resistance-associated protein 5///multidrug resistance-associate	1.107	1.13E-07	7.83E-06
RknMes02 024963	AT5G59220.1	1.00E-12	cassava4.1 007913	Protein phosphatase 2C family protein///highly ABA-induced PP2C gene	1.105	1.42E-07	8.83E-06
RknMes02 004871	AT1G20270.1	2.00E-35	cassava4.1 013142	2-oxoglutarate (2OG) and Fe(II)-dependent oxygenase superfamily protei	1.104	7.39E-06	9.96E-05
RknMes02 011372	AT3G53230.1	2.00E-32	cassava4.1 001878	cell division cycle 48///"ATPase, AAA-type, CDC48 protein"	1.103	2.81E-06	5.18E-05
RknMes02_021408					1.103	2.88E-06	5.25E-05
RknMes02 033693	AT3G03330.1	0	cassava4.1 024654	NAD(P)-binding Rossmann-fold superfamily protein	1.102	8.04E-09	1.88E-06
RknMes02 036575	AT4G33090.1	0	cassava4.1 001498	aminopeptidase M1	1.102	3.84E-07	1.50E-05
RknMes02_034955			cassava4.1_009209m		1.101	1.16E-10	2.54E-07
RknMes02 039434	AT4G34500.1	0	cassava4.1 026968	Protein kinase superfamily protein///cassava4.1_017407m	1.100	3.79E-07	1.49E-05
RknMes02 034385	AT1G54520.1	2.00E-34	cassava4.1 017125	Unknown///unknown protein; FUNCTIONS IN: molecular function unkn	1.099	5.16E-06	7.80E-05
RknMes02 023606	AT4G33220.1	0	cassava4.1 005656	pectin methylesterase 44	1.097	1.86E-06	4.00E-05
RknMes02 027987	AT1G60420.1	0	cassava4.1 008293	DC1 domain-containing protein	1.097	8.74E-08	6.85E-06
RknMes02 013377	AT5G05690.1	2.80E-45	cassava4.1 006864	Cytochrome P450 superfamily protein	1.094	8.62E-08	6.81E-06
RknMes02 031881	AT5G21920.1	0	cassava4.1 014893	YGGT family protein	1.092	2.80E-07	1.26E-05
RknMes02 024886	AT2G41810.1	0	cassava4.1 010037	Protein of unknown function, DUF642	1.089	1.46E-07	9.00E-06
RknMes02 010471	AT3G54100.1	1.00E-09	cassava4.1 030087	O-fucosyltransferase family protein	1.086	2.45E-06	4.75E-05
RknMes02 049194	AT2G02240.1	0	cassava4.1 012495	F-box family protein///phloem protein 2-B1	1.080	9.73E-08	7.23E-06

RknMes02 027200	AT3G19553.1	0	cassava4.1 006044	Amino acid permease family protein	1.076	7.90E-07	2.31E-05
RknMes02 027039	AT1G79070.1	2.00E-31	cassava4.1 018223	SNARE-associated protein-related	1.074	4.96E-09	1.54E-06
RknMes02 003917	AT2G29630.3	2.00E-30	cassava4.1 003350	thiaminC	1.074	1.34E-06	3.23E-05
RknMes02 032316	AT5G64700.1	0	cassava4.1 010374	nodulin MtN21 /EamA-like transporter family protein	1.073	3.27E-06	5.73E-05
RknMes02 032617	AT1G76490.1	2.00E-29	cassava4.1 003914	hydroxy methylglutaryl CoA reductase 1	1.065	5.85E-06	8.47E-05
RknMes02 000259	AT4G10160.1	1.00E-08	cassava4.1 021144	RING/U-box superfamily protein	1.065	7.03E-06	9.62E-05
RknMes02 035946	AT1G34300.1	1.00E-27	cassava4.1 001853	receptor-like protein kinase 1///S-domain-2 5///lectin protein kinase fami	1.062	7.96E-07	2.32E-05
RknMes02 001491	AT1G79070.1	8.00E-17	cassava4.1 018156	SNARE-associated protein-related	1.061	1.87E-07	1.01E-05
RknMes02 003889	AT3G04300.1	4.00E-36	cassava4.1 023553	RmlC-like cupins superfamily protein	1.059	3.45E-07	1.42E-05
RknMes02 022618	AT2G29500.1	0	cassava4.1 033656	HSP20-like chaperones superfamily protein	1.056	3.66E-09	1.37E-06
RknMes02 021928	AT1G21840.1	3.00E-40	cassava4.1 014923	urease accessory protein F	1.054	2.54E-07	1.20E-05
RknMes02 035616	AT2G28680.1	0	cassava4.1 010513	RmlC-like cupins superfamily protein	1.053	3.78E-08	4.44E-06
RknMes02 031570	AT2G28680.1	0	cassava4.1 010513	RmlC-like cupins superfamily protein	1.053	7.89E-08	6.42E-06
RknMes02 013610	AT3G52990.2	5.00E-17	cassava4.1 006316	Pyruvate kinase family protein	1.051	1.09E-06	2.82E-05
RknMes02 000403	AT2G40460.1	3.00E-30	cassava4.1 004356	Major facilitator superfamily protein	1.050	3.73E-06	6.28E-05
RknMes02_010647			cassava4.1_006597m		1.050	1.03E-06	2.71E-05
RknMes02 013621	AT1G15690.1	4.00E-10	cassava4.1 002160	Inorganic H pyrophosphatase family protein	1.049	9.56E-08	7.22E-06
RknMes02 001099	AT2G24520.1	0	cassava4.1 001104	H(+)-ATPase 5	1.049	6.09E-06	8.71E-05
RknMes02 007712	AT3G16240.1	4.00E-13	cassava4.1 014710	delta tonoplast integral protein///Unknown	1.048	5.44E-06	8.08E-05
RknMes02 006068	AT1G07430.1	2.00E-20	cassava4.1 007913	highly ABA-induced PP2C gene 2	1.046	1.03E-07	7.43E-06
RknMes02 049022	AT5G23680.1	4.00E-18	cassava4.1 011238	Sterile alpha motif (SAM) domain-containing protein	1.046	1.09E-06	2.81E-05
RknMes02_001387					1.045	9.54E-09	2.10E-06
RknMes02 012112	AT5G28500.1	2.00E-18	cassava4.1 026834	unknown protein; BEST Arabidopsis thaliana protein match is: unknown	1.043	5.72E-06	8.35E-05
RknMes02 057665	AT1G66810.1	1.00E-06	cassava4.1 031947	Zinc finger C-x8-C-x5-C-x3-H type family protein	1.042	3.43E-07	1.42E-05
RknMes02 058666	AT3G48520.1	0	cassava4.1 033581	cytochrome P450, family 94, subfamily B, polypeptide 3	1.041	5.58E-08	5.38E-06
RknMes02 053211	AT1G76500.1	0	cassava4.1 024479	Predicted AT-hook DNA-binding family protein	1.041	7.98E-07	2.33E-05
RknMes02_050345			cassava4.1_018564m		1.040	4.96E-07	1.72E-05
RknMes02 049485	AT4G13720.1	0	cassava4.1 014284	Inosine triphosphate pyrophosphatase family protein	1.039	1.14E-07	7.87E-06
RknMes02 014407	AT2G28680.1	9.00E-15	cassava4.1 010513	RmlC-like cupins superfamily protein	1.036	9.08E-08	7.01E-06
RknMes02 049247	AT4G36830.1	0	cassava4.1 012827	GNS1/SUR4 membrane protein family	1.034	4.74E-06	7.36E-05
RknMes02 024426	AT3G13120.2	0	cassava4.1 016092	Unknown///Ribosomal protein S10p/S20e family protein	1.032	4.32E-06	6.92E-05
RknMes02 026433	AT2G43710.1	0	cassava4.1 009202	Plant stearoyl-acyl-carrier-protein desaturase family protein	1.030	1.86E-08	3.04E-06
RknMes02 027619	AT1G60420.1	0	cassava4.1 004565	DC1 domain-containing protein	1.025	2.83E-07	1.27E-05
RknMes02 005842	AT1G04280.1	0	cassava4.1 005863	P-loop containing nucleoside triphosphate hydrolases superfamily protei	1.025	1.58E-07	9.27E-06
RknMes02_008267			cassava4.1_011240m		1.025	2.58E-08	3.58E-06
RknMes02 048312	AT1G17680.2	0	cassava4.1 004904	tetratricopeptide repeat (TPR)-containing protein	1.024	1.30E-07	8.40E-06
RknMes02 014898	AT5G28500.1	6.00E-07	cassava4.1 026834	unknown protein; BEST Arabidopsis thaliana protein match is: unknown	1.023	3.30E-08	4.14E-06
RknMes02 058901	AT4G26200.1	0	cassava4.1 033963	1-amino-cyclopropane-1-carboxylate synthase 7	1.021	6.10E-06	8.73E-05
RknMes02 005512	AT1G76490.1	0	cassava4.1 003914	hydroxy methylglutaryl CoA reductase 1	1.021	6.07E-07	1.93E-05
RknMes02 033399	AT3G21760.1	0	cassava4.1 018585	UDP-Glycosyltransferase superfamily protein///UDP-glucosyl transferase	1.021	2.29E-06	4.58E-05
RknMes02 032061	AT3G61870.1	0	cassava4.1 013792	unknown protein; FUNCTIONS IN: molecular function unknown; INVO	1.015	2.81E-06	5.17E-05
RknMes02 002919	AT3G54310.1	6.00E-17	cassava4.1 013894	unknown protein; BEST Arabidopsis thaliana protein match is: unknown	1.015	3.71E-07	1.47E-05
RknMes02 036257	AT5G23240.1	3.00E-29	cassava4.1 006681	DNAJ heat shock N-terminal domain-containing protein	1.010	5.15E-07	1.75E-05
RknMes02 047856	AT5G03850.1	1.00E-21		Nucleic acid-binding, OB-fold-like protein	1.007	2.84E-06	5.20E-05
RknMes02 011906	AT4G39970.1	3.00E-15	cassava4.1 012518	Haloacid dehalogenase-like hydrolase (HAD) superfamily protein	1.006	1.75E-07	9.78E-06
RknMes02 053612	AT4G03320.1	0	cassava4.1 025130	translocon at the inner envelope membrane of chloroplasts 20-IV	1.004	2.08E-08	3.27E-06
RknMes02 024112	AT3G16000.1	4.10E-44	cassava4.1 027490	MAR binding filament-like protein 1	1.004	5.84E-06	8.46E-05
RknMes02 013842	AT5G15450.1	1.00E-09	cassava4.1 029777	casein lytic proteinase B3	1.002	8.71E-08	6.85E-06
RknMes02 024972	AT3G47420.1	4.00E-21	cassava4.1 005536	phosphate starvation-induced gene 3	1.002	1.46E-08	2.64E-06
RknMes02 025530	AT1G26480.1	0	cassava4.1 015311	general regulatory factor 12	1.001	3.24E-06	5.69E-05
RknMes02 049835	AT5G63130.1	8.00E-40	cassava4.1 016137	Octicosapeptide/Phox/Bem1p family protein	1.001	7.90E-07	2.31E-05
RknMes02 000900	AT4G34500.1	3.00E-24	cassava4.1 026968	Protein kinase superfamily protein///cassava4.1_017407m	1.000	6.18E-09	1.67E-06

1) AGI code is shown if proteins encoded in each cassava gene (probe ID) have high amino acid sequence similarity (E value ≤ 10^{-5}) to *Arabidopsis* homologs.

2) E-value shows similarity in amino acid sequence between each cassava gene (probe ID) and *Arabidopsis* homolog.

3) Encoded proteins/other features indicate the putative functions of the gene products that are expected from sequence similarity. The information for the NCBI protein reference sequence with the highest sequence similarity to the probes is shown.

TABLE S4. Genes up-regulated in cassava roots by SAHA treatment under 2 h NaCl

Probe ID	AGI code[1)]	E-value[2)]	Cassava ID	Encoded proteins/other features[3)]	$\log_2$ ratio {(NaCl 2 h after SAHA 24 h)/(NaCl 2 h after non-SAHA 24 h)}	p-value	BH FDR
RknMes02_057300	AT2G13810.1	0	cassava4.1_031362m	AGD2-like defense response protein 1	7.348	2.31E-09	1.13E-06
RknMes02_058141	AT3G06720.2	0	cassava4.1_032699m	importin alpha isoform 1	6.563	1.79E-09	1.01E-06
RknMes02_051874	AT1G13280.1	0	cassava4.1_022180m	allene oxide cyclase 4	5.966	3.51E-11	1.70E-07
RknMes02_052316	AT4G01950.1	0	cassava4.1_022947m	glycerol-3-phosphate acyltransferase 3	5.717	1.28E-07	8.32E-06
RknMes02_055141	AT2G40210.1	5.00E-33	cassava4.1_027752m	AGAMOUS-like 48	5.695	2.60E-09	1.18E-06
RknMes02_058688	AT3G28880.1	0	cassava4.1_033616m	Ankyrin repeat family protein	5.456	2.98E-10	4.22E-07
RknMes02_050833	AT4G39250.1	2.00E-32	cassava4.1_020375m	RAD-like 1///RAD-like 6	5.386	4.47E-09	1.48E-06
RknMes02_009639	AT1G34300.1	5.00E-21	cassava4.1_001853m	receptor-like protein kinase 1///S-domain-2 5///lectin protein kinase family protein	5.369	4.18E-07	1.57E-05
RknMes02_053062	AT1G68390.1	0	cassava4.1_024214m	Core-2/I-branching beta-1,6-N-acetylglucosaminyltransferase family protein	5.273	7.05E-10	6.51E-07
RknMes02_057007	AT5G57620.1	0	cassava4.1_030900m	myb domain protein 36	5.228	1.46E-06	3.40E-05
RknMes02_058115	AT3G07390.1	0	cassava4.1_032657m	auxin-responsive family protein///Auxin-responsive family protein	5.167	1.10E-06	2.84E-05
RknMes02_058010	AT5G07610.1	1.00E-16	cassava4.1_032488m	F-box family protein	4.974	1.18E-07	7.95E-06
RknMes02_053766	AT4G33870.1	0	cassava4.1_025400m	Peroxidase superfamily protein	4.907	5.08E-07	1.74E-05
RknMes02_001711	AT4G32460.2	1.00E-19	cassava4.1_010077m	Protein of unknown function, DUF642	4.892	2.31E-09	1.13E-06
RknMes02_057698	AT2G45400.1	0	cassava4.1_032002m	NAD(P)-binding Rossmann-fold superfamily protein	4.872	2.17E-08	3.32E-06
RknMes02_051856	AT1G35910.1	0	cassava4.1_022149m	Haloacid dehalogenase-like hydrolase (HAD) superfamily protein///trehalose-6-phosphate phospl	4.833	2.03E-06	4.21E-05
RknMes02_050655	AT3G04710.3	1.00E-14	cassava4.1_019864m	ankyrin repeat family protein	4.790	6.33E-08	5.74E-06
RknMes02_054888	AT1G01690.1	0	cassava4.1_027325m	putative recombination initiation defects 3	4.788	6.03E-08	5.59E-06
RknMes02_057539	AT5G07610.1	3.00E-08	cassava4.1_031747m	F-box family protein	4.770	7.51E-11	2.44E-07
RknMes02_055406	AT5G17350.1	4.00E-27	cassava4.1_028188m	AT5G17350.1///AT3G03280.1	4.768	9.10E-12	1.08E-07
RknMes02_001781	AT3G58110.2	3.00E-28	cassava4.1_004503m	unknown protein; FUNCTIONS IN: molecular_function unknown; INVOLVED IN: biological_pr	4.741	3.51E-09	1.34E-06
RknMes02_054063	AT1G14440.2	2.80E-45	cassava4.1_025934m	homeobox protein 33///homeobox protein 31	4.683	4.87E-06	7.51E-05
RknMes02_052664	AT5G64310.1	9.00E-15	cassava4.1_023538m	Unknown///arabinogalactan protein 1///cassava4.1_023538m	4.529	2.67E-06	4.99E-05
RknMes02_035946	AT1G34300.1	1.00E-27	cassava4.1_001853m	receptor-like protein kinase 1///S-domain-2 5///lectin protein kinase family protein	4.492	7.96E-07	2.32E-05
RknMes02_037315	AT4G00330.1	4.00E-18	cassava4.1_032262m	calmodulin-binding receptor-like cytoplasmic kinase 2	4.438	8.00E-09	1.88E-06
RknMes02_051803	AT3G09270.1	3.00E-11	cassava4.1_022056m	glutathione S-transferase TAU 8///glutathione S-transferase tau 7	4.369	2.91E-07	1.29E-05
RknMes02_037654	AT4G29110.1	1.00E-07	cassava4.1_016277m	unknown protein; FUNCTIONS IN: molecular_function unknown; INVOLVED IN: response to c	4.337	9.47E-08	7.18E-06
RknMes02_051464	AT3G06240.1	1.00E-24	cassava4.1_021488m	F-box family protein///F-box and associated interaction domains-containing protein	4.284	1.78E-08	2.95E-06
RknMes02_056068	AT3G28880.1	3.00E-30	cassava4.1_029312m	Ankyrin repeat family protein	4.275	1.20E-06	3.00E-05
RknMes02_053782	AT4G17380.1	0	cassava4.1_025428m	MUTS-like protein 4	4.231	3.11E-07	1.34E-05
RknMes02_049288	AT5G52060.1	0	cassava4.1_013107m	BCL-2-associated athanogene 1	4.218	7.41E-07	2.22E-05
RknMes02_055721	AT4G39250.1	3.00E-36	cassava4.1_028739m	RAD-like 1///RAD-like 6	4.154	9.54E-10	7.62E-07
RknMes02_000416	AT2G26870.1	0	cassava4.1_005538m	non-specific phospholipase C2	4.138	1.37E-11	1.20E-07
RknMes02_051638	AT4G22660.1	2.00E-10	cassava4.1_021777m	F-box family protein with a domain of unknown function (DUF295)	4.008	3.02E-06	5.43E-05
RknMes02_051551	AT2G33480.2	5.00E-10	cassava4.1_021626m	NAC domain containing protein 52	3.998	2.96E-08	3.89E-06
RknMes02_052689	AT5G16080.1	0	cassava4.1_023579m	carboxyesterase 17	3.989	1.59E-06	3.58E-05
RknMes02_020256	AT3G19270.1	0	cassava4.1_006983m	cytochrome P450, family 707, subfamily A, polypeptide 4	3.985	1.45E-06	3.39E-05
RknMes02_011799	AT2G28090.1	2.00E-13	cassava4.1_011919m	Heavy metal transport/detoxification superfamily protein	3.984	2.36E-06	4.66E-05
RknMes02_055845	AT3G51880.4	6.00E-16	cassava4.1_028951m	high mobility group B2///high mobility group B1	3.974	3.24E-07	1.37E-05
RknMes02_007372			cassava4.1_011919m		3.962	1.34E-06	3.23E-05
RknMes02_026790	AT2G26870.1	0	cassava4.1_005538m	non-specific phospholipase C2	3.934	4.07E-12	6.01E-08
RknMes02_051562	AT5G64360.4	2.00E-13	cassava4.1_021648m	Chaperone DnaJ-domain superfamily protein	3.929	2.89E-12	5.97E-08
RknMes02_054294	AT1G67810.1	0	cassava4.1_026316m	sulfur E2	3.918	1.28E-09	8.44E-07
RknMes02_056535	AT5G53980.1	9.00E-34	cassava4.1_030094m	homeobox protein 52	3.912	3.01E-08	3.92E-06
RknMes02_055397	AT2G26140.1	0	cassava4.1_028173m	FTSH protease 4	3.786	9.44E-08	7.16E-06
RknMes02_039148			cassava4.1_002717m		3.689	2.31E-09	1.13E-06
RknMes02_057486	AT2G21220.1	1.00E-35	cassava4.1_031669m	SAUR-like auxin-responsive protein family	3.613	1.51E-10	2.98E-07
RknMes02_056495			cassava4.1_030018m		3.609	2.35E-06	4.64E-05
RknMes02_053760	AT3G20475.1	0	cassava4.1_025390m	MUTS-homologue 5	3.547	2.55E-10	3.87E-07
RknMes02_001575	AT4G25640.2	6.00E-30	cassava4.1_006392m	detoxifying efflux carrier 35	3.523	3.21E-07	1.37E-05
RknMes02_023807	AT4G00330.1	6.00E-30	cassava4.1_031817m	calmodulin-binding receptor-like cytoplasmic kinase 2	3.503	2.15E-07	1.09E-05
RknMes02_056633	AT1G29050.1	0	cassava4.1_030267m	TRICHOME BIREFRINGENCE-LIKE 38	3.450	1.64E-07	9.40E-06
RknMes02_054016	AT5G42120.1	0	cassava4.1_025847m	Concanavalin A-like lectin protein kinase family protein	3.408	3.96E-07	1.52E-05
RknMes02_052163	AT2G22840.1	0	cassava4.1_022687m	growth-regulating factor 1	3.407	1.70E-06	3.76E-05
RknMes02_022940	AT4G20970.1	2.00E-12	cassava4.1_024041m	basic helix-loop-helix (bHLH) DNA-binding superfamily protein	3.384	1.22E-07	8.07E-06
RknMes02_053494	AT1G72570.1	0	cassava4.1_024934m	Integrase-type DNA-binding superfamily protein	3.366	2.86E-08	3.79E-06
RknMes02_048392	AT2G38940.1	0	cassava4.1_005715m	phosphate transporter 1;7///Unknown///phosphate transporter 1;5///phosphate transporter 1;4	3.364	6.58E-08	5.87E-06
RknMes02_003488	AT3G54140.1	4.00E-14	cassava4.1_024513m	peptide transporter 1	3.345	1.99E-07	1.05E-05
RknMes02_013101	AT1G69200.1	3.00E-06	cassava4.1_008596m	Unknown///fructokinase-like 2	3.311	1.80E-06	3.91E-05
RknMes02_020142	AT3G60670.1	0	cassava4.1_014925m	PLATZ transcription factor family protein	3.307	1.97E-07	1.04E-05
RknMes02_053434	AT4G22600.1	0	cassava4.1_024833m	AT4G22600.1	3.273	3.63E-07	1.46E-05
RknMes02_044414					3.261	1.08E-06	2.80E-05
RknMes02_050000	AT4G15630.1	0	cassava4.1_016898m	Uncharacterised protein family (UPF0497)	3.233	4.30E-06	6.89E-05
RknMes02_052194	AT4G24970.1	0	cassava4.1_022738m	Histidine kinase-, DNA gyrase B-, and HSP90-like ATPase family protein	3.230	4.19E-08	4.63E-06
RknMes02_046039					3.223	4.59E-06	7.20E-05
RknMes02_033982	AT3G54820.1	0	cassava4.1_011438m	plasma membrane intrinsic protein 2;5	3.215	1.23E-08	2.42E-06
RknMes02_007453	AT1G01320.2	0	cassava4.1_000135m	Tetratricopeptide repeat (TPR)-like superfamily protein	3.206	3.61E-06	6.15E-05
RknMes02_049317	AT2G16050.1	2.90E-44	cassava4.1_013278m	Cysteine/Histidine-rich C1 domain family protein	3.132	9.60E-07	2.60E-05
RknMes02_053618	AT5G08460.1	0	cassava4.1_025139m	GDSL-like Lipase/Acylhydrolase superfamily protein	3.131	4.71E-08	4.92E-06
RknMes02_034590	AT1G10340.2	4.00E-18	cassava4.1_004232m	Ankyrin repeat family protein	3.112	2.05E-07	1.06E-05
RknMes02_000277	AT4G25640.2	0	cassava4.1_006392m	detoxifying efflux carrier 35	3.077	1.34E-06	3.23E-05
RknMes02_055022	AT4G33870.1	0	cassava4.1_027550m	Peroxidase superfamily protein	3.064	2.83E-09	1.18E-06
RknMes02_017956	AT5G14230.1	0	cassava4.1_002222m	AT5G14230.1///"CONTAINS InterPro DOMAIN/s: Ankyrin repeat-containing domain (InterPro:I	3.049	2.48E-06	4.78E-05
RknMes02_056377	AT2G32460.2	0	cassava4.1_029822m	myb domain protein 101	3.039	1.05E-06	2.76E-05
RknMes02_054668	AT4G36740.1	0	cassava4.1_026937m	homeobox protein 40///homeobox protein 21	3.030	4.30E-09	1.46E-06
RknMes02_055451	AT4G15630.1	0	cassava4.1_028267m	Uncharacterised protein family (UPF0497)	3.000	1.04E-06	2.74E-05
RknMes02_023448			cassava4.1_009880m		2.935	2.50E-06	4.80E-05
RknMes02_031436	AT3G48700.1	0	cassava4.1_026915m	carboxyesterase 13	2.906	1.41E-06	3.34E-05
RknMes02_043801					2.895	4.32E-08	4.73E-06
RknMes02_006435	AT5G65690.1	1.00E-32	cassava4.1_030131m	phosphoenolpyruvate carboxykinase 2	2.872	7.84E-08	6.40E-06
RknMes02_056066	AT3G06600.2	2.00E-07	cassava4.1_029310m	cassava4.1_029310m	2.865	2.45E-06	4.76E-05
RknMes02_050157	AT4G05430.1	2.00E-33	cassava4.1_017684m	Carbohydrate-binding X8 domain superfamily protein	2.855	5.34E-06	7.98E-05
RknMes02_056640	AT3G11930.1	0	cassava4.1_030279m	Adenine nucleotide alpha hydrolases-like superfamily protein	2.853	2.04E-08	3.24E-06
RknMes02_008825	AT2G36580.1	2.00E-12	cassava4.1_006316m	Pyruvate kinase family protein	2.842	4.36E-07	1.61E-05
RknMes02_055230	AT3G05950.1	0	cassava4.1_027899m	RmlC-like cupins superfamily protein	2.841	5.15E-06	7.80E-05
RknMes02_057133	AT2G18460.1	0	cassava4.1_031089m	like COV 3	2.836	7.93E-10	6.97E-07
RknMes02_048720	AT1G03790.1	0	cassava4.1_008973m	Zinc finger C-x8-C-x5-C-x3-H type family protein	2.766	4.36E-08	4.76E-06
RknMes02_040157	AT5G65690.1	0	cassava4.1_030131m	phosphoenolpyruvate carboxykinase 2	2.765	3.50E-08	4.27E-06
RknMes02_019176	AT4G01580.1	2.00E-17	cassava4.1_013238m	AP2/B3-like transcriptional factor family protein	2.761	1.82E-06	3.93E-05
RknMes02_051165	AT2G36020.1	7.00E-25	cassava4.1_021018m	HVA22-like protein J	2.747	3.48E-06	5.98E-05
RknMes02_057627	AT4G32480.1	0	cassava4.1_031890m	Protein of unknown function (DUF506)	2.738	4.63E-07	1.66E-05
RknMes02_052167	AT5G24530.1	0	cassava4.1_022692m	2-oxoglutarate (2OG) and Fe(II)-dependent oxygenase superfamily protein	2.729	3.06E-06	5.48E-05
RknMes02_048263	AT1G26610.1	5.00E-33	cassava4.1_004354m	C2H2-like zinc finger protein	2.719	7.20E-06	9.77E-05
RknMes02_032811	AT4G25640.2	0	cassava4.1_006392m	detoxifying efflux carrier 35	2.706	2.72E-07	1.24E-05
RknMes02_054342	AT2G28420.1	0	cassava4.1_026393m	Lactoylglutathione lyase / glyoxalase I family protein	2.696	6.03E-07	1.92E-05
RknMes02_034530	AT4G23810.1	8.00E-35	cassava4.1_010768m	WRKY family transcription factor///WRKY DNA-binding protein 30	2.660	2.97E-06	5.36E-05
RknMes02_047468					2.659	5.83E-07	1.88E-05
RknMes02_057836	AT1G17020.1	0	cassava4.1_032207m	2-oxoglutarate (2OG) and Fe(II)-dependent oxygenase superfamily protein///senescence-related g	2.654	7.69E-09	1.85E-06
RknMes02_001193	AT5G48820.1	8.00E-10	cassava4.1_032196m	Unknown	2.645	4.23E-06	6.83E-05
RknMes02_004432	AT5G11420.1	2.00E-23	cassava4.1_010027m	Protein of unknown function, DUF642	2.630	5.92E-09	1.65E-06
RknMes02_000900	AT4G34500.1	3.00E-24	cassava4.1_026968m	Protein kinase superfamily protein///cassava4.1_017407m	2.628	6.18E-09	1.67E-06
RknMes02_034595	AT1G34050.1	1.00E-34	cassava4.1_004232m	Ankyrin repeat family protein	2.604	1.11E-06	2.85E-05
RknMes02_039434	AT4G34500.1	0	cassava4.1_026968m	Protein kinase superfamily protein///cassava4.1_017407m	2.589	3.79E-07	1.49E-05
RknMes02_053308	AT1G54200.1	0	cassava4.1_024636m	AT3G42800.1///unknown protein; BEST Arabidopsis thaliana protein match is: unknown protein	2.569	3.46E-08	4.24E-06
RknMes02_051666	AT4G02570.4	0	cassava4.1_021824m	cullin 1	2.567	2.84E-06	5.21E-05
RknMes02_024350	AT3G51240.1	0	cassava4.1_010212m	flavanone 3-hydroxylase	2.560	2.17E-06	4.41E-05
RknMes02_039767			cassava4.1_008879m		2.557	4.71E-07	1.67E-05
RknMes02_055668	AT5G55850.3	3.00E-19	cassava4.1_028651m	RPM1-interacting protein 4 (RIN4) family protein	2.551	1.15E-07	7.87E-06
RknMes02_022677	AT4G25040.1	4.00E-12	cassava4.1_017297m	Uncharacterised protein family (UPF0497)///Unknown	2.547	2.33E-10	3.82E-07
RknMes02_056248	AT4G39230.1	0	cassava4.1_029598m	NmrA-like negative transcriptional regulator family protein	2.543	1.25E-07	8.20E-06
RknMes02_000313	AT5G33370.1	3.00E-43		GDSL-like Lipase/Acylhydrolase superfamily protein	2.539	1.60E-06	3.61E-05
RknMes02_003389	AT4G15560.1	4.00E-39	cassava4.1_030091m	Deoxyxylulose-5-phosphate synthase	2.525	5.53E-09	1.60E-06
RknMes02_007402	AT4G37870.1	0	cassava4.1_030131m	phosphoenolpyruvate carboxykinase 1	2.525	4.74E-08	4.92E-06
RknMes02_040160	AT1G04280.1	7.00E-24	cassava4.1_005863m	P-loop containing nucleoside triphosphate hydrolases superfamily protein	2.518	1.90E-07	1.02E-05
RknMes02_005011	AT3G44610.1	8.00E-44	cassava4.1_007698m	Protein kinase superfamily protein	2.518	6.52E-07	2.03E-05
RknMes02_058174	AT2G44990.1	0	cassava4.1_032749m	carotenoid cleavage dioxygenase 7	2.501	1.78E-07	9.87E-06
RknMes02_052114	AT3G14670.2	3.00E-06	cassava4.1_022594m	cassava4.1_022594m	2.497	1.41E-06	3.34E-05
RknMes02_030183	AT3G52870.1	0	cassava4.1_014011m	IQ calmodulin-binding motif family protein	2.487	1.78E-06	3.88E-05
RknMes02_054272	AT3G09270.1	0	cassava4.1_026272m	glutathione S-transferase TAU 8///glutathione S-transferase tau 7	2.487	9.66E-07	2.61E-05
RknMes02_047928	AT3G27870.1	0	cassava4.1_000480m	ATPase E1-E2 type family protein / haloacid dehalogenase-like hydrolase family protein	2.482	1.41E-09	8.96E-07
RknMes02_010687	AT4G15560.1	6.00E-36	cassava4.1_030091m	Deoxyxylulose-5-phosphate synthase	2.479	1.57E-10	2.99E-07
RknMes02_041567	AT2G04520.1	8.00E-33	cassava4.1_023644m	Nucleic acid-binding, OB-fold-like protein	2.472	4.23E-06	6.83E-05
RknMes02_038192	AT3G44610.1	0	cassava4.1_007698m	Protein kinase superfamily protein	2.467	4.43E-07	1.62E-05

RknMes02_013610	AT3G52990.2	5.00E-17	cassava4.1_006316m	Pyruvate kinase family protein	2.457	1.09E-06	2.82E-05
RknMes02_032617	AT1G76490.1	2.00E-29	cassava4.1_003914m	hydroxy methylglutaryl CoA reductase 1	2.448	5.85E-06	8.47E-05
RknMes02_052307	AT1G51080.1	0	cassava4.1_022931m	AT1G51080.1	2.446	2.63E-06	4.95E-05
RknMes02_013200	AT4G34131.1	3.00E-08	cassava4.1_029076m	UDP-glucosyl transferase 73B3	2.395	2.09E-08	3.27E-06
RknMes02_022476	AT5G59050.2	5.00E-10	cassava4.1_010283m	unknown protein; Has 35333 Blast hits to 34131 proteins in 2444 species: Archae - 798; Bacteria	2.386	9.18E-10	7.43E-07
RknMes02_039325	AT5G54880.1	0	cassava4.1_034239m	DTW domain-containing protein	2.384	3.46E-07	1.42E-05
RknMes02_005842	AT1G04280.1	0	cassava4.1_005863m	P-loop containing nucleoside triphosphate hydrolases superfamily protein	2.373	1.58E-07	9.27E-06
RknMes02_030843	AT1G75090.1	0	cassava4.1_011004m	DNA glycosylase superfamily protein///Unknown	2.359	2.39E-06	4.69E-05
RknMes02_057556	AT4G02170.1	2.00E-19	cassava4.1_031780m	AT4G02170.1	2.343	5.91E-06	8.54E-05
RknMes02_033467	AT1G75090.1	1.00E-11	cassava4.1_011004m	DNA glycosylase superfamily protein///Unknown	2.331	3.77E-07	1.48E-05
RknMes02_055143	AT5G39820.1	6.00E-12	cassava4.1_027756m	NAC-like, activated by AP3/PI	2.327	2.02E-06	4.21E-05
RknMes02_010122	AT5G60800.2	7.00E-13	cassava4.1_010149m	Unknown///Heavy metal transport/detoxification superfamily protein	2.320	1.53E-06	3.50E-05
RknMes02_042934					2.312	2.20E-06	4.45E-05
RknMes02_045593					2.308	1.82E-07	9.95E-06
RknMes02_031518	AT2G39700.1	0	cassava4.1_014440m	expansin A9///expansin A4	2.299	5.77E-08	5.45E-06
RknMes02_004348			cassava4.1_011742m		2.296	7.22E-08	6.16E-06
RknMes02_030435	AT4G37870.1	0	cassava4.1_033411m	phosphoenolpyruvate carboxykinase 1	2.296	1.39E-08	2.60E-06
RknMes02_001717	AT5G08260.1	7.00E-15	cassava4.1_020819m	serine carboxypeptidase-like 35	2.295	1.21E-06	3.02E-05
RknMes02_003748			cassava4.1_017297m		2.294	5.35E-09	1.59E-06
RknMes02_048226	AT3G16380.1	0	cassava4.1_004058m	poly(A) binding protein 6	2.290	1.86E-07	1.01E-05
RknMes02_034621	AT3G52870.1	1.00E-32	cassava4.1_005734m	IQ calmodulin-binding motif family protein	2.286	1.94E-07	1.03E-05
RknMes02_025971	AT1G43910.1	0	cassava4.1_006163m	P-loop containing nucleoside triphosphate hydrolases superfamily protein	2.262	2.05E-06	4.24E-05
RknMes02_021249	AT1G76490.1	4.00E-30	cassava4.1_003914m	hydroxy methylglutaryl CoA reductase 1	2.259	2.96E-07	1.30E-05
RknMes02_011864			cassava4.1_029076m		2.251	2.23E-08	3.36E-06
RknMes02_015171			cassava4.1_020405m		2.251	6.33E-07	1.99E-05
RknMes02_006988			cassava4.1_006747m		2.250	4.25E-06	6.84E-05
RknMes02_012269	AT1G06460.1	2.00E-10	cassava4.1_015713m	alpha-crystallin domain 32.1	2.248	4.63E-06	7.23E-05
RknMes02_004852	AT1G64430.2	7.00E-23	cassava4.1_013173m	Pentatricopeptide repeat (PPR) superfamily protein	2.240	6.71E-06	9.32E-05
RknMes02_011304	AT1G43910.1	5.00E-22	cassava4.1_006163m	P-loop containing nucleoside triphosphate hydrolases superfamily protein	2.230	1.17E-06	2.95E-05
RknMes02_009571	AT3G09550.1	8.00E-43	cassava4.1_007548m	Ankyrin repeat family protein	2.225	1.16E-06	2.93E-05
RknMes02_051622	AT2G21790.1	0	cassava4.1_021751m	ribonucleotide reductase 1	2.222	3.61E-08	4.32E-06
RknMes02_023054			cassava4.1_021277m		2.217	4.39E-06	6.98E-05
RknMes02_048519	AT3G21760.1	0	cassava4.1_007027m	UDP-Glycosyltransferase superfamily protein///UDP-glucosyl transferase 71B6	2.214	6.93E-07	2.12E-05
RknMes02_031697	AT1G34770.1	7.00E-27	cassava4.1_023953m	CONTAINS InterPro DOMAIN/s: MAGE protein (InterPro:IPR002190); Has 1274 Blast hits to 1	2.208	2.23E-07	1.11E-05
RknMes02_038586	AT1G03220.1	2.00E-28	cassava4.1_007945m	Eukaryotic aspartyl protease family protein	2.198	2.13E-06	4.35E-05
RknMes02_037190	AT2G28680.1	5.00E-33	cassava4.1_010513m	RmlC-like cupins superfamily protein	2.191	9.16E-09	2.07E-06
RknMes02_052535	AT3G09270.1	0	cassava4.1_023315m	glutathione S-transferase TAU 8///glutathione S-transferase tau 7	2.184	4.47E-07	1.63E-05
RknMes02_029441	AT5G24910.1	0	cassava4.1_005649m	cytochrome P450, family 714, subfamily A, polypeptide 1	2.181	2.05E-07	1.07E-05
RknMes02_004885	AT2G14660.1	9.00E-37	cassava4.1_022246m	unknown protein; CONTAINS InterPro DOMAIN/s: Uncharacterised protein family UPF0310 (Ir	2.177	3.58E-07	1.45E-05
RknMes02_022969	AT5G24900.1	3.00E-34	cassava4.1_006808m	cytochrome P450, family 714, subfamily A, polypeptide 2	2.177	7.08E-07	2.15E-05
RknMes02_007011	AT3G01470.1	9.00E-31	cassava4.1_012760m	homeobox 1	2.173	1.46E-08	2.64E-06
RknMes02_048557	AT4G32000.2	0	cassava4.1_007289m	Protein kinase superfamily protein///Unknown	2.173	9.01E-07	2.50E-05
RknMes02_052720	AT1G25220.1	0	cassava4.1_023625m	anthranilate synthase beta subunit 1	2.168	1.43E-06	3.36E-05
RknMes02_004259	AT4G37870.1	9.00E-23	cassava4.1_033411m	phosphoenolpyruvate carboxykinase 1	2.168	2.39E-09	1.15E-06
RknMes02_009054	AT4G35590.1	4.00E-31	cassava4.1_024901m	NIN like protein 7///RWP-RK domain-containing protein	2.160	1.94E-08	3.14E-06
RknMes02_048668	AT3G25410.1	0	cassava4.1_008480m	Sodium Bile acid symporter family	2.159	1.49E-06	3.43E-05
RknMes02_030292	AT5G08260.1	0	cassava4.1_010410m	serine carboxypeptidase-like 35	2.157	3.52E-07	1.43E-05
RknMes02_035809			cassava4.1_012760m		2.156	1.27E-08	2.45E-06
RknMes02_031742	AT4G12320.1	0	cassava4.1_005312m	cytochrome P450, family 706, subfamily A, polypeptide 6///"cytochrome P450, family 706, subfa	2.152	3.25E-06	5.71E-05
RknMes02_001250	AT4G23060.1	3.00E-07		IQ-domain 22///Unknown	2.152	1.64E-07	9.38E-06
RknMes02_005512	AT1G76490.1	0	cassava4.1_003914m	hydroxy methylglutaryl CoA reductase 1	2.148	6.07E-07	1.93E-05
RknMes02_001499	AT5G54160.1	1.00E-21	cassava4.1_013376m	O-methyltransferase 1	2.147	6.28E-06	8.90E-05
RknMes02_030263	AT1G80050.1	0	cassava4.1_016994m	adenine phosphoribosyl transferase 2	2.145	1.52E-09	9.44E-07
RknMes02_013439					2.139	4.42E-07	1.62E-05
RknMes02_006698	AT4G21920.1	4.00E-08	cassava4.1_030675m	unknown protein; FUNCTIONS IN: molecular_function unknown; INVOLVED IN: N-terminal p	2.130	4.51E-06	7.12E-05
RknMes02_039442	AT5G55560.1	0	cassava4.1_012772m	Protein kinase superfamily protein	2.130	2.41E-08	3.43E-06
RknMes02_023980	AT2G36870.1	0	cassava4.1_013124m	xyloglucan endotransglucosylase/hydrolase 32	2.123	6.43E-07	2.01E-05
RknMes02_042673					2.119	5.42E-07	1.80E-05
RknMes02_055923	AT3G55350.1	0	cassava4.1_029072m	PIF / Ping-Pong family of plant transposases	2.117	6.17E-08	5.67E-06
RknMes02_037144	AT3G45970.1	6.00E-17	cassava4.1_015495m	expansin-like A1	2.112	1.94E-06	4.11E-05
RknMes02_049508			cassava4.1_014414m		2.108	5.77E-08	5.45E-06
RknMes02_034435			cassava4.1_011089m		2.102	3.14E-06	5.58E-05
RknMes02_055925	AT4G34135.1	0	cassava4.1_029076m	UDP-glucosyltransferase 73B2///UDP-glucosyl transferase 73B3	2.101	7.19E-09	1.80E-06
RknMes02_021073	AT1G72880.2	0	cassava4.1_022171m	Survival protein SurE-like phosphatase/nucleotidase	2.098	4.53E-09	1.48E-06
RknMes02_014623	AT3G25410.1	2.00E-07	cassava4.1_008480m	Sodium Bile acid symporter family	2.094	2.04E-07	1.06E-05
RknMes02_049693			cassava4.1_015474m		2.093	8.58E-07	2.43E-05
RknMes02_036183	AT3G22810.1	0	cassava4.1_007076m	Plant protein of unknown function (DUF828) with plant pleckstrin homology-like region	2.083	1.20E-07	8.01E-06
RknMes02_013621	AT1G15690.1	4.00E-10	cassava4.1_002160m	Inorganic H pyrophosphatase family protein	2.083	9.56E-08	7.22E-06
RknMes02_053679	AT2G35950.1	5.00E-07	cassava4.1_025248m	embryo sac development arrest 12	2.080	6.08E-06	8.71E-05
RknMes02_054653	AT4G03965.1	3.00E-36	cassava4.1_026906m	RING/U-box superfamily protein	2.074	1.18E-06	2.96E-05
RknMes02_000403	AT2G40460.1	3.00E-30	cassava4.1_004356m	Major facilitator superfamily protein	2.073	3.73E-06	6.28E-05
RknMes02_035810	AT2G37220.1	0	cassava4.1_013463m	RNA-binding (RRM/RBD/RNP motifs) family protein	2.064	2.68E-07	1.23E-05
RknMes02_000647	AT4G31940.1	6.00E-17	cassava4.1_005168m	cytochrome P450, family 82, subfamily C, polypeptide 2///"cytochrome P450, family 82, subfami	2.063	4.28E-06	6.88E-05
RknMes02_051348	AT2G28930.3	0	cassava4.1_021311m	protein kinase 1B	2.048	2.49E-06	4.80E-05
RknMes02_039581	AT5G24910.1	0	cassava4.1_005649m	cytochrome P450, family 714, subfamily A, polypeptide 1	2.047	2.84E-07	1.27E-05
RknMes02_051084	AT5G24320.1	0	cassava4.1_020893m	Transducin/WD40 repeat-like superfamily protein	2.039	2.47E-06	4.77E-05
RknMes02_006016			cassava4.1_015713m		2.032	1.47E-07	9.03E-06
RknMes02_022664	AT2G13440.1	0	cassava4.1_003007m	glucose-inhibited division family A protein	2.023	1.42E-07	8.86E-06
RknMes02_051046	AT5G08260.1	1.00E-23	cassava4.1_020819m	serine carboxypeptidase-like 35	2.021	3.75E-07	1.48E-05
RknMes02_040226	AT1G75290.1	5.00E-16	cassava4.1_034037m	NAD(P)-binding Rossmann-fold superfamily protein	2.018	1.40E-06	3.33E-05
RknMes02_031443	AT2G37220.1	0	cassava4.1_018180m	RNA-binding (RRM/RBD/RNP motifs) family protein	2.002	4.70E-08	4.92E-06
RknMes02_052366	AT1G21830.1	3.00E-10	cassava4.1_023020m	AT1G21830.1	1.999	5.47E-06	8.10E-05
RknMes02_052829	AT4G02390.1	0	cassava4.1_023824m	poly(ADP-ribose) polymerase	1.996	9.83E-09	2.13E-06
RknMes02_039445	AT2G41380.1	0	cassava4.1_014142m	S-adenosyl-L-methionine-dependent methyltransferases superfamily protein	1.996	3.28E-08	4.13E-06
RknMes02_010647			cassava4.1_006597m		1.991	1.03E-06	2.71E-05
RknMes02_053612	AT4G03320.1	0	cassava4.1_025130m	translocon at the inner envelope membrane of chloroplasts 20-IV	1.991	2.08E-08	3.27E-06
RknMes02_049984	AT1G58170.1	0	cassava4.1_016826m	Disease resistance-responsive (dirigent-like protein) family protein	1.989	2.87E-06	5.25E-05
RknMes02_058121	AT4G28780.1	0	cassava4.1_032666m	GDSL-like Lipase/Acylhydrolase superfamily protein	1.978	2.92E-06	5.30E-05
RknMes02_019058	AT1G80050.1	3.00E-18		adenine phosphoribosyl transferase 2	1.978	2.43E-08	3.44E-06
RknMes02_027039	AT1G79070.1	2.00E-31	cassava4.1_018223m	SNARE-associated protein-related	1.977	4.96E-09	1.54E-06
RknMes02_031179	AT5G60800.2	2.00E-34	cassava4.1_010149m	Unknown///Heavy metal transport/detoxification superfamily protein	1.974	1.84E-06	3.97E-05
RknMes02_049176	AT3G18990.1	2.00E-28	cassava4.1_012400m	AP2/B3-like transcriptional factor family protein	1.970	1.50E-07	9.06E-06
RknMes02_048405	AT2G26150.1	0	cassava4.1_005870m	heat shock transcription factor A2	1.962	2.12E-06	4.34E-05
RknMes02_046527	AT5G60160.1	1.00E-28	cassava4.1_006533m	Zn-dependent exopeptidases superfamily protein	1.959	2.08E-06	4.28E-05
RknMes02_051740	AT1G54115.1	0	cassava4.1_021941m	cation calcium exchanger 4	1.954	6.01E-09	1.66E-06
RknMes02_034644	AT5G53420.1	0	cassava4.1_013858m	CCT motif family protein	1.951	1.40E-06	3.32E-05
RknMes02_008108	AT1G04420.1	2.00E-32	cassava4.1_008972m	NAD(P)-linked oxidoreductase superfamily protein	1.947	7.00E-08	6.05E-06
RknMes02_039863	AT5G20050.1	0	cassava4.1_007583m	Protein kinase superfamily protein	1.947	1.85E-07	1.01E-05
RknMes02_032068	AT3G23000.1	0	cassava4.1_008191m	CBL-interacting protein kinase 7	1.946	5.52E-07	1.82E-05
RknMes02_012029	AT1G27620.1	1.00E-10	cassava4.1_007495m	HXXXD-type acyl-transferase family protein	1.933	2.54E-06	4.84E-05
RknMes02_031200	AT5G48500.1	1.00E-35	cassava4.1_018166m	unknown protein; BEST Arabidopsis thaliana protein match is: unknown protein (TAIR:AT2G10	1.930	1.31E-06	3.19E-05
RknMes02_028619	AT4G11820.2	9.00E-24	cassava4.1_007081m	hydroxymethylglutaryl-CoA synthase / HMG-CoA synthase / 3-hydroxy-3-methylglutaryl coenzyr	1.929	5.56E-06	8.19E-05
RknMes02_010313	AT5G62790.2	4.00E-14	cassava4.1_006908m	1-deoxy-D-xylulose 5-phosphate reductoisomerase	1.927	2.24E-09	1.12E-06
RknMes02_007614	AT4G34500.1	1.00E-38	cassava4.1_026968m	Protein kinase superfamily protein///cassava4.1_017407m	1.925	1.54E-07	9.16E-06
RknMes02_042989	AT1G21240.1	5.00E-34	cassava4.1_022223m	wall associated kinase 3	1.921	5.65E-06	8.29E-05
RknMes02_057031	AT5G01260.2	1.00E-25	cassava4.1_030934m	Carbohydrate-binding-like fold	1.919	2.94E-06	5.33E-05
RknMes02_002656	AT5G42010.1	8.00E-09	cassava4.1_020893m	Transducin/WD40 repeat-like superfamily protein	1.915	1.58E-06	3.57E-05
RknMes02_004375	AT1G05010.1	6.00E-15	cassava4.1_012052m	ethylene-forming enzyme	1.915	6.95E-06	9.54E-05
RknMes02_053183	AT1G77400.1	5.00E-37	cassava4.1_024425m	AT1G77400.1	1.914	3.16E-06	5.61E-05
RknMes02_010998	AT3G52610.1	2.00E-30	cassava4.1_006747m	unknown protein; Has 68 Blast hits to 67 proteins in 21 species: Archae - 0; Bacteria - 11; Metaz	1.911	4.59E-06	7.20E-05
RknMes02_014511	AT4G02390.1	1.00E-11		poly(ADP-ribose) polymerase	1.907	5.80E-08	5.46E-06
RknMes02_032596	AT3G56400.1	2.00E-42	cassava4.1_013417m	WRKY DNA-binding protein 70	1.901	4.11E-07	1.56E-05
RknMes02_022618	AT2G29500.1	0	cassava4.1_033656m	HSP20-like chaperones superfamily protein	1.900	3.66E-09	1.37E-06
RknMes02_049780	AT4G36390.1	2.00E-23	cassava4.1_015930m	Methylthiotransferase	1.899	4.57E-08	4.86E-06
RknMes02_053794	AT3G06720.2	0	cassava4.1_025451m	importin alpha isoform 1	1.887	1.28E-06	3.14E-05
RknMes02_031640	AT1G79160.1	8.00E-44	cassava4.1_014502m	unknown protein; BEST Arabidopsis thaliana protein match is: unknown protein (TAIR:AT1G16	1.878	1.91E-06	4.06E-05
RknMes02_021162					1.876	6.76E-07	2.09E-05
RknMes02_030645	AT1G72880.2	1.00E-14	cassava4.1_022171m	Survival protein SurE-like phosphatase/nucleotidase	1.868	9.23E-09	2.07E-06
RknMes02_031531	AT5G24020.1	0	cassava4.1_011379m	septum site-determining protein (MIND)	1.868	1.73E-08	2.92E-06
RknMes02_032669	AT1G80840.1	1.00E-08	cassava4.1_033249m	WRKY DNA-binding protein 40///WRKY DNA-binding protein 18	1.864	1.16E-06	2.93E-05
RknMes02_035933	AT2G38610.2	0	cassava4.1_013445m	RNA-binding KH domain-containing protein	1.858	1.36E-08	2.56E-06
RknMes02_042780					1.845	4.16E-06	6.75E-05
RknMes02_007490	AT1G80760.1	0	cassava4.1_027276m	NOD26-like intrinsic protein 6;1	1.842	9.60E-08	7.22E-06
RknMes02_051166	AT5G59030.1	1.00E-42	cassava4.1_021023m	Ctr copper transporter family///copper transporter 1	1.840	9.58E-07	2.60E-05
RknMes02_020013	AT5G37670.1	1.00E-33	cassava4.1_019659m	HSP20-like chaperones superfamily protein	1.837	3.21E-06	5.66E-05
RknMes02_010174			cassava4.1_010705m		1.832	7.43E-08	6.19E-06
RknMes02_014741	AT1G27620.1	1.00E-08	cassava4.1_007495m	HXXXD-type acyl-transferase family protein	1.830	6.42E-08	5.78E-06

RknMes02_028606	AT4G02940.1	8.00E-37	cassava4.1_004959m	oxidoreductase, 2OG-Fe(II) oxygenase family protein	1.828	5.26E-06	7.90E-05
RknMes02_032968	AT4G39970.1	0	cassava4.1_013098m	Haloacid dehalogenase-like hydrolase (HAD) superfamily protein	1.826	1.76E-07	9.80E-06
RknMes02_009806	AT1G01540.2	5.00E-09	cassava4.1_005727m	Protein kinase superfamily protein	1.820	2.16E-06	4.39E-05
RknMes02_025630					1.814	4.87E-07	1.70E-05
RknMes02_050135	AT3G52460.1	6.00E-19	cassava4.1_017573m	Unknown///hydroxyproline-rich glycoprotein family protein	1.810	2.61E-09	1.18E-06
RknMes02_013123			cassava4.1_032103m		1.807	7.44E-07	2.23E-05
RknMes02_005363	AT2G34790.1	8.00E-44	cassava4.1_004979m	FAD-binding Berberine family protein	1.804	9.96E-07	2.65E-05
RknMes02_050020	AT5G51160.1	2.00E-19	cassava4.1_017002m	Ankyrin repeat family protein	1.803	2.14E-09	1.12E-06
RknMes02_014176	AT3G25900.1	1.00E-24	cassava4.1_011630m	Homocysteine S-methyltransferase family protein	1.802	7.55E-08	6.25E-06
RknMes02_026864	AT4G31940.1	0	cassava4.1_005168m	cytochrome P450, family 82, subfamily C, polypeptide 2///"cytochrome P450, family 82, subfami	1.798	1.63E-07	9.36E-06
RknMes02_050321	AT2G20515.1	2.80E-45	cassava4.1_018459m	AT2G20515.1	1.797	4.24E-07	1.59E-05
RknMes02_032680	AT3G02470.4	9.00E-36	cassava4.1_010384m	S-adenosylmethionine decarboxylase	1.794	1.77E-06	3.87E-05
RknMes02_052218	AT5G16850.1	0	cassava4.1_022790m	telomerase reverse transcriptase	1.790	1.51E-06	3.46E-05
RknMes02_001491	AT1G79070.1	8.00E-17	cassava4.1_018156m	SNARE-associated protein-related	1.784	1.87E-07	1.01E-05
RknMes02_035017			cassava4.1_018099m		1.774	1.17E-07	7.93E-06
RknMes02_012429	AT1G12010.1	2.00E-08	cassava4.1_012052m	2-oxoglutarate (2OG) and Fe(II)-dependent oxygenase superfamily protein	1.772	1.63E-06	3.66E-05
RknMes02_025530	AT1G26480.1	0	cassava4.1_015311m	general regulatory factor 12	1.771	3.24E-06	5.69E-05
RknMes02_051360	AT2G22460.1	0	cassava4.1_021332m	Protein of unknown function, DUF617	1.771	2.91E-07	1.29E-05
RknMes02_004458	AT5G13860.1	2.00E-06		ELCH-like	1.768	9.39E-08	7.14E-06
RknMes02_032098	AT4G37760.1	0	cassava4.1_006000m	FAD/NAD(P)-binding oxidoreductase family protein///squalene epoxidase 2///squalene epoxidas	1.767	3.35E-06	5.84E-05
RknMes02_024886	AT2G41810.1	0	cassava4.1_010037m	Protein of unknown function, DUF642	1.767	1.46E-07	9.00E-06
RknMes02_057388	AT2G17570.1	0	cassava4.1_031506m	Undecaprenyl pyrophosphate synthetase family protein	1.764	1.11E-07	7.77E-06
RknMes02_009542	AT1G55740.1	4.00E-15	cassava4.1_002249m	seed imbibition 1	1.761	1.88E-06	4.01E-05
RknMes02_036091	AT2G26190.1	0	cassava4.1_005851m	Unknown///calmodulin-binding family protein	1.759	7.20E-06	9.78E-05
RknMes02_038768	AT1G58440.1	0	cassava4.1_006000m	FAD/NAD(P)-binding oxidoreductase family protein	1.759	1.22E-06	3.04E-05
RknMes02_057805	AT3G24060.1	1.00E-13	cassava4.1_032165m	Plant self-incompatibility protein S1 family	1.755	3.68E-07	1.47E-05
RknMes02_011013			cassava4.1_016218m		1.752	3.32E-06	5.80E-05
RknMes02_025204	AT1G09960.1	4.00E-33	cassava4.1_007925m	Unknown///sucrose transporter 4	1.750	6.83E-06	9.43E-05
RknMes02_012316	AT5G22800.1	1.00E-08	cassava4.1_000907m	Alanyl-tRNA synthetase, class IIc	1.748	1.40E-08	2.61E-06
RknMes02_010565	AT5G43960.2	2.00E-18	cassava4.1_007259m	Unknown///Nuclear transport factor 2 (NTF2) family protein with RNA binding (RRM-RBD-RN	1.744	3.65E-06	6.20E-05
RknMes02_049345	AT3G01470.1	0	cassava4.1_013480m	homeobox 1	1.742	1.07E-06	2.78E-05
RknMes02_027276	AT1G52980.1	8.00E-30	cassava4.1_019581m	GTP-binding family protein	1.738	4.51E-09	1.48E-06
RknMes02_025103	AT4G20170.1	5.00E-40	cassava4.1_005572m	Domain of unknown function (DUF23)	1.734	1.75E-07	9.78E-06
RknMes02_048095	AT2G28350.1	0	cassava4.1_002668m	auxin response factor 10	1.733	5.64E-08	5.42E-06
RknMes02_000236	AT1G09950.1	1.00E-08	cassava4.1_033345m	RESPONSE TO ABA AND SALT 1	1.732	1.86E-06	3.99E-05
RknMes02_005623	AT5G24020.1	0	cassava4.1_011379m	septum site-determining protein (MIND)	1.729	4.13E-08	4.59E-06
RknMes02_033990	AT2G45120.1	0	cassava4.1_010237m	C2H2-like zinc finger protein	1.725	8.09E-08	6.48E-06
RknMes02_053502	AT1G14205.1	0	cassava4.1_024946m	Ribosomal L18p/L5e family protein	1.721	3.00E-10	4.22E-07
RknMes02_051771	AT1G18340.1	0	cassava4.1_022008m	basal transcription factor complex subunit-related	1.718	8.99E-07	2.50E-05
RknMes02_001387					1.717	9.54E-09	2.10E-06
RknMes02_058825	AT1G33760.1	0	cassava4.1_033833m	Integrase-type DNA-binding superfamily protein	1.715	2.49E-06	4.79E-05
RknMes02_037825	AT2G01290.1	0	cassava4.1_014596m	ribose-5-phosphate isomerase 2	1.713	6.20E-07	1.96E-05
RknMes02_013107	AT4G00370.1	3.00E-39	cassava4.1_005826m	Major facilitator superfamily protein	1.712	4.13E-06	6.72E-05
RknMes02_056961	AT2G24762.1	6.00E-17	cassava4.1_003829m	glutamine dumper 4	1.707	2.29E-08	3.36E-06
RknMes02_033702	AT5G64260.1	0	cassava4.1_012973m	EXORDIUM like 2	1.702	1.31E-06	3.19E-05
RknMes02_012739	AT2G18550.1	3.00E-32	cassava4.1_027319m	homeobox protein 21	1.700	1.32E-08	2.53E-06
RknMes02_031388	AT5G65780.1	0	cassava4.1_012023m	branched-chain amino acid aminotransferase 5 / branched-chain amino acid transaminase 5 (BCA	1.694	2.55E-10	3.87E-07
RknMes02_044477					1.692	1.15E-06	2.92E-05
RknMes02_011696	AT3G07720.1	7.00E-35	cassava4.1_011729m	Galactose oxidase/kelch repeat superfamily protein	1.688	5.73E-06	8.37E-05
RknMes02_024844	AT2G16230.1	0	cassava4.1_007302m	O-Glycosyl hydrolases family 17 protein	1.687	2.10E-06	4.30E-05
RknMes02_018495	AT4G23010.3	0	cassava4.1_010920m	UDP-galactose transporter 2	1.686	6.83E-08	5.99E-06
RknMes02_010961	AT5G09225.1	5.00E-13		unknown protein; FUNCTIONS IN: molecular function unknown; INVOLVED IN: biological pr	1.685	1.03E-07	7.46E-06
RknMes02_030480	AT2G37390.2	3.00E-40	cassava4.1_013260m	Chloroplast-targeted copper chaperone protein	1.679	1.78E-07	9.88E-06
RknMes02_037255	AT2G22430.1	3.00E-14	cassava4.1_012269m	homeobox protein 6	1.678	9.66E-08	7.22E-06
RknMes02_007039	AT4G27960.2	3.00E-25	cassava4.1_016218m	ubiquitin conjugating enzyme 9	1.676	5.39E-06	8.03E-05
RknMes02_002136	AT5G22870.1	6.00E-07	cassava4.1_017573m	Late embryogenesis abundant (LEA) hydroxyproline-rich glycoprotein family	1.675	3.79E-08	4.44E-06
RknMes02_001046	AT5G58900.1	2.00E-13	cassava4.1_012770m	Homeodomain-like transcriptional regulator	1.674	1.58E-06	3.57E-05
RknMes02_049852	AT1G73885.1	2.00E-34	cassava4.1_016218m	AT1G73885.1	1.673	1.60E-06	3.61E-05
RknMes02_023973	AT5G07440.3	4.00E-28	cassava4.1_008693m	glutamate dehydrogenase 2	1.672	3.49E-07	1.42E-05
RknMes02_003822	AT5G09760.1	2.00E-42	cassava4.1_004459m	Plant invertase/pectin methylesterase inhibitor superfamily	1.671	1.45E-07	8.98E-06
RknMes02_049851	AT3G22160.1	2.00E-14	cassava4.1_016216m	VQ motif-containing protein///Unknown	1.667	5.92E-06	8.55E-05
RknMes02_031958	AT5G09760.1	5.00E-37	cassava4.1_006847m	Plant invertase/pectin methylesterase inhibitor superfamily	1.661	4.41E-07	1.62E-05
RknMes02_057665	AT1G66810.1	1.00E-06	cassava4.1_031947m	Zinc finger C-x8-C-x5-C-x3-H type family protein	1.657	3.43E-07	1.42E-05
RknMes02_034955			cassava4.1_009209m		1.650	1.16E-10	2.54E-07
RknMes02_054089	AT1G65570.1	0	cassava4.1_025978m	Pectin lyase-like superfamily protein	1.649	2.59E-06	4.90E-05
RknMes02_011906	AT4G39970.1	3.00E-15	cassava4.1_012518m	Haloacid dehalogenase-like hydrolase (HAD) superfamily protein	1.647	1.75E-07	9.78E-06
RknMes02_005509			cassava4.1_000498m		1.645	8.62E-07	2.44E-05
RknMes02_050345			cassava4.1_018564m		1.645	4.96E-07	1.72E-05
RknMes02_049813	AT3G16300.1	0	cassava4.1_016042m	Uncharacterised protein family (UPF0497)	1.636	3.24E-06	5.70E-05
RknMes02_035642	AT1G65910.1	5.00E-14	cassava4.1_009209m	NAC domain containing protein 28	1.636	9.65E-11	2.44E-07
RknMes02_007766	AT5G09760.1	5.60E-45	cassava4.1_006847m	Plant invertase/pectin methylesterase inhibitor superfamily	1.635	1.31E-07	8.43E-06
RknMes02_054650	AT5G55850.3	9.00E-21	cassava4.1_026901m	RPM1-interacting protein 4 (RIN4) family protein	1.634	3.21E-06	5.66E-05
RknMes02_011352	AT3G43740.2	1.00E-14	cassava4.1_015871m	Leucine-rich repeat (LRR) family protein	1.633	2.06E-06	4.26E-05
RknMes02_006465	AT5G64260.1	3.00E-24	cassava4.1_012973m	EXORDIUM like 2	1.630	1.65E-06	3.68E-05
RknMes02_049955	AT2G35900.1	0	cassava4.1_016725m	unknown protein; FUNCTIONS IN: molecular function unknown; INVOLVED IN: biological pr	1.628	2.48E-09	1.17E-06
RknMes02_036388	AT2G44480.2	0	cassava4.1_005766m	Glycosyl hydrolase superfamily protein///beta glucosidase 17	1.627	5.75E-07	1.87E-05
RknMes02_008482			cassava4.1_010513m		1.626	1.03E-07	7.43E-06
RknMes02_015109			cassava4.1_017002m		1.624	4.00E-09	1.42E-06
RknMes02_004910	AT3G49220.1	6.00E-16	cassava4.1_003930m	Plant invertase/pectin methylesterase inhibitor superfamily	1.619	4.79E-06	7.42E-05
RknMes02_048719	AT5G22300.1	0	cassava4.1_008964m	nitrilase 4	1.619	8.03E-08	6.47E-06
RknMes02_035449	AT3G14200.1	0	cassava4.1_014653m	Chaperone DnaJ-domain superfamily protein	1.619	1.16E-06	2.93E-05
RknMes02_030184	AT1G62780.1	0	cassava4.1_015022m	unknown protein; FUNCTIONS IN: molecular function unknown; INVOLVED IN: biological pr	1.617	2.76E-09	1.18E-06
RknMes02_049235	AT1G73440.1	0	cassava4.1_012758m	calmodulin-related	1.615	8.77E-07	2.47E-05
RknMes02_022084			cassava4.1_013343m		1.615	5.57E-08	5.38E-06
RknMes02_025483	AT3G55350.1	1.00E-32	cassava4.1_021993m	PIF / Ping-Pong family of plant transposases	1.614	9.83E-07	2.63E-05
RknMes02_057548	AT3G50770.1	2.00E-29	cassava4.1_031768m	calmodulin-like 41	1.614	2.56E-06	4.87E-05
RknMes02_029879	AT5G03710.1	6.00E-06		Unknown	1.607	1.41E-06	3.33E-05
RknMes02_002293					1.607	7.68E-07	2.27E-05
RknMes02_003930			cassava4.1_031768m		1.604	8.23E-07	2.37E-05
RknMes02_021125			cassava4.1_019870m		1.603	1.47E-06	3.41E-05
RknMes02_007753	AT5G20540.1	2.00E-35	cassava4.1_009818m	BREVIS RADIX-like 4	1.602	5.71E-07	1.87E-05
RknMes02_026417	AT5G50330.1	0	cassava4.1_006600m	Protein kinase superfamily protein	1.602	8.51E-08	6.74E-06
RknMes02_049358	AT3G60410.3	5.00E-28	cassava4.1_013535m	Unknown///Protein of unknown function (DUF1639)	1.599	5.82E-11	2.29E-07
RknMes02_010628			cassava4.1_001759m		1.599	2.87E-10	4.22E-07
RknMes02_034401	AT5G67200.1	2.00E-43	cassava4.1_003207m	Leucine-rich repeat protein kinase family protein	1.595	8.22E-07	2.36E-05
RknMes02_002071			cassava4.1_017002m		1.593	5.51E-09	1.60E-06
RknMes02_056904	AT5G48500.1	1.00E-26	cassava4.1_030733m	unknown protein; BEST Arabidopsis thaliana protein match is: unknown protein (TAIR:AT2G10	1.591	1.15E-06	2.91E-05
RknMes02_012991	AT5G07440.3	7.00E-43	cassava4.1_008701m	glutamate dehydrogenase 2	1.588	5.19E-06	7.84E-05
RknMes02_001636			cassava4.1_007088m		1.580	1.67E-07	9.53E-06
RknMes02_032061	AT3G61870.1	0	cassava4.1_013792m	unknown protein; FUNCTIONS IN: molecular function unknown; INVOLVED IN: biological pr	1.579	2.81E-06	5.17E-05
RknMes02_039001	AT2G43945.1	8.00E-14	cassava4.1_016545m	unknown protein; LOCATED IN: chloroplast; BEST Arabidopsis thaliana protein match is: unkn	1.574	1.33E-06	3.22E-05
RknMes02_019499					1.573	1.57E-07	9.25E-06
RknMes02_001763	AT2G19780.1	4.00E-37	cassava4.1_008576m	Leucine-rich repeat (LRR) family protein	1.571	7.16E-08	6.15E-06
RknMes02_048993	AT4G24050.1	0	cassava4.1_011047m	NAD(P)-binding Rossmann-fold superfamily protein	1.570	2.21E-08	3.35E-06
RknMes02_011604			cassava4.1_016042m		1.569	3.85E-06	6.42E-05
RknMes02_029768	AT1G78700.1	0	cassava4.1_011718m	BES1/BZR1 homolog 3///BES1/BZR1 homolog 4	1.556	5.07E-06	7.72E-05
RknMes02_014898	AT5G28500.1	6.00E-07	cassava4.1_026834m	unknown protein; BEST Arabidopsis thaliana protein match is: unknown protein (TAIR:AT3G04	1.555	3.30E-08	4.14E-06
RknMes02_021811	AT2G27880.1	0	cassava4.1_000940m	Argonaute family protein	1.551	4.63E-06	7.23E-05
RknMes02_014510	AT3G02630.1	8.00E-27	cassava4.1_009202m	Plant stearoyl-acyl-carrier-protein desaturase family protein	1.550	1.42E-11	1.20E-07
RknMes02_009745	AT1G18680.1	3.00E-26	cassava4.1_028673m	HNH endonuclease domain-containing protein	1.544	6.89E-08	6.01E-06
RknMes02_053638	AT1G19780.1	0	cassava4.1_025182m	cyclic nucleotide gated channel 8	1.544	6.34E-06	8.95E-05
RknMes02_025242			cassava4.1_016895m		1.537	1.08E-06	2.80E-05
RknMes02_058666	AT3G48520.1	0	cassava4.1_033581m	cytochrome P450, family 94, subfamily B, polypeptide 3	1.536	5.58E-08	5.38E-06
RknMes02_052318	AT1G65980.1	0	cassava4.1_022949m	thioredoxin-dependent peroxidase 1	1.534	2.58E-08	3.58E-06
RknMes02_037504	AT1G68060.1	5.00E-17	cassava4.1_007403m	microtubule-associated proteins 70-1	1.525	7.38E-06	9.95E-05
RknMes02_007426	AT2G36000.2	2.00E-34	cassava4.1_012762m	Mitochondrial transcription termination factor family protein	1.524	5.07E-06	7.72E-05
RknMes02_053876	AT3G07040.1	3.00E-35	cassava4.1_025604m	NB-ARC domain-containing disease resistance protein	1.523	5.84E-06	8.46E-05
RknMes02_023231	AT2G39950.1	4.00E-24	cassava4.1_003002m	unknown protein; Has 978 Blast hits to 254 proteins in 81 species: Archae - 0; Bacteria - 8; Meta	1.520	3.43E-06	5.93E-05
RknMes02_049743	AT3G03330.1	0	cassava4.1_015717m	NAD(P)-binding Rossmann-fold superfamily protein	1.520	2.10E-09	1.12E-06
RknMes02_005143	AT2G29630.3	2.00E-17	cassava4.1_003359m	thiaminC	1.513	9.74E-07	2.62E-05
RknMes02_048688	AT5G07440.2	0	cassava4.1_008693m	glutamate dehydrogenase 2	1.506	2.40E-06	4.70E-05
RknMes02_039417	AT3G07360.1	0	cassava4.1_032891m	plant U-box 9	1.501	6.45E-06	9.08E-05
RknMes02_049788	AT5G21090.1	0	cassava4.1_015963m	Leucine-rich repeat (LRR) family protein	1.501	2.61E-07	1.22E-05
RknMes02_031807	AT5G53370.1	0	cassava4.1_003930m	Plant invertase/pectin methylesterase inhibitor superfamily///pectin methylesterase PCR fragmen	1.499	5.03E-06	7.68E-05
RknMes02_002192	AT2G30020.1	2.00E-14	cassava4.1_009896m	Protein phosphatase 2C family protein	1.498	8.91E-07	2.49E-05
RknMes02_037463	AT1G56200.2	1.00E-17	cassava4.1_017469m	embryo defective 1303	1.497	1.21E-10	2.55E-07
RknMes02_026784	AT4G26370.1	0	cassava4.1_012367m	antitermination NusB domain-containing protein	1.494	6.26E-06	8.89E-05

RknMes02_007714			cassava4.1_022173m		1.494	2.18E-07	1.10E-05
RknMes02_014325	AT1G80660.1	0	cassava4.1_001102m	H(+)-ATPase 9///H(+)-ATPase 6	1.493	3.97E-06	6.57E-05
RknMes02_001702	AT1G58440.1	0	cassava4.1_005310m	FAD/NAD(P)-binding oxidoreductase family protein	1.491	9.21E-07	2.53E-05
RknMes02_009808			cassava4.1_018166m		1.484	1.66E-08	2.86E-06
RknMes02_039654	AT3G18295.1	3.00E-19	cassava4.1_010362m	Protein of unknown function (DUF1639)	1.482	2.77E-07	1.25E-05
RknMes02_049409			cassava4.1_013825m		1.480	8.12E-09	1.90E-06
RknMes02_002825	AT5G66130.1	4.00E-22	cassava4.1_003824m	RADIATION SENSITIVE 17	1.479	8.44E-09	1.95E-06
RknMes02_001127	AT2G38970.1	2.00E-32		Zinc finger (C3HC4-type RING finger) family protein	1.478	1.22E-08	2.42E-06
RknMes02_015255	AT1G22410.1	0	cassava4.1_009820m	Class-II DAHP synthetase family protein	1.477	4.21E-09	1.45E-06
RknMes02_049835	AT5G63130.1	8.00E-40	cassava4.1_016137m	Octicosapeptide/Phox/Bem1p family protein	1.476	7.90E-07	2.31E-05
RknMes02_025071	AT4G02940.1	2.00E-31	cassava4.1_004959m	oxidoreductase, 2OG-Fe(II) oxygenase family protein	1.474	1.37E-08	2.57E-06
RknMes02_055886	AT4G26700.2	0	cassava4.1_029014m	fimbrin 1	1.473	5.06E-06	7.71E-05
RknMes02_014240	AT3G44220.1	7.00E-32		Late embryogenesis abundant (LEA) hydroxyproline-rich glycoprotein family	1.471	6.61E-06	9.23E-05
RknMes02_020777	AT1G18340.1	7.00E-23	cassava4.1_022008m	basal transcription factor complex subunit-related	1.471	8.96E-07	2.49E-05
RknMes02_002489	AT3G60410.3	1.00E-06	cassava4.1_013343m	Unknown///Protein of unknown function (DUF1639)	1.464	8.76E-09	2.01E-06
RknMes02_020155	AT4G21120.1	0	cassava4.1_027926m	amino acid transporter 1	1.463	3.12E-06	5.55E-05
RknMes02_041238	AT3G48830.1	2.00E-22	cassava4.1_005493m	polynucleotide adenylyltransferase family protein / RNA recognition motif (RRM)-containing prc	1.461	3.57E-07	1.45E-05
RknMes02_026024	AT3G14860.2	0	cassava4.1_006580m	NHL domain-containing protein	1.457	7.22E-08	6.16E-06
RknMes02_034168	AT1G01970.1	0	cassava4.1_010436m	Tetratricopeptide repeat (TPR)-like superfamily protein	1.457	1.36E-06	3.27E-05
RknMes02_015113	AT1G73440.1	2.00E-18	cassava4.1_012758m	calmodulin-related	1.450	3.83E-07	1.50E-05
RknMes02_003139	AT5G02790.1	3.00E-41	cassava4.1_015004m	Glutathione S-transferase family protein	1.450	7.40E-08	6.19E-06
RknMes02_032390	AT3G21760.1	0	cassava4.1_031478m	UDP-Glycosyltransferase superfamily protein///UDP-glucosyl transferase 71B6	1.449	1.67E-06	3.72E-05
RknMes02_029920	AT3G20760.1	1.00E-24	cassava4.1_009430m	Nse4, component of Smc5/6 DNA repair complex	1.448	1.06E-10	2.44E-07
RknMes02_035308	AT2G03470.2	3.00E-12	cassava4.1_013932m	ELM2 domain-containing protein	1.444	9.17E-07	2.53E-05
RknMes02_005045	AT3G04550.1	3.00E-23	cassava4.1_026834m	unknown protein; INVOLVED IN: biological process unknown; LOCATED IN: chloroplast stror	1.441	1.46E-08	2.64E-06
RknMes02_009118	AT3G51240.2	6.00E-17	cassava4.1_010212m	flavanone 3-hydroxylase	1.439	1.82E-07	9.95E-06
RknMes02_003150	AT5G08790.1	2.00E-20	cassava4.1_012943m	NAC (No Apical Meristem) domain transcriptional regulator superfamily protein	1.438	1.46E-06	3.40E-05
RknMes02_028175	AT3G23000.1	0	cassava4.1_008412m	CBL-interacting protein kinase 7	1.438	5.40E-07	1.80E-05
RknMes02_052026	AT3G57230.1	6.00E-28	cassava4.1_022444m	AGAMOUS-like 16	1.437	2.81E-06	5.17E-05
RknMes02_027058	AT4G01370.1	0	cassava4.1_009399m	MAP kinase 4	1.432	7.78E-07	2.29E-05
RknMes02_040214	AT2G27880.1	2.00E-15	cassava4.1_000940m	Argonaute family protein	1.431	3.19E-06	5.64E-05
RknMes02_022578	AT1G48100.1	2.00E-14	cassava4.1_006566m	Pectin lyase-like superfamily protein	1.431	4.29E-07	1.60E-05
RknMes02_000227	AT3G52380.1	0	cassava4.1_012128m	chloroplast RNA-binding protein 33	1.430	7.77E-08	6.36E-06
RknMes02_048869	AT5G47500.1	0	cassava4.1_010240m	Pectin lyase-like superfamily protein	1.429	1.46E-06	3.39E-05
RknMes02_048904	AT5G60580.3	0	cassava4.1_010466m	RING/U-box superfamily protein	1.428	2.37E-08	3.40E-06
RknMes02_004828	AT4G33090.1	7.00E-31	cassava4.1_003844m	aminopeptidase M1	1.426	1.71E-07	9.64E-06
RknMes02_006756	AT4G14580.1	1.00E-27	cassava4.1_008412m	Unknown///CBL-interacting protein kinase 4	1.426	2.55E-08	3.56E-06
RknMes02_039709	AT2G16365.4	2.00E-11	cassava4.1_004275m	F-box family protein	1.424	2.59E-06	4.90E-05
RknMes02_035498	AT2G40080.1	8.00E-28	cassava4.1_019555m	Protein of unknown function (DUF1313)	1.422	6.24E-08	5.71E-06
RknMes02_009748			cassava4.1_004640m		1.421	3.07E-06	5.48E-05
RknMes02_053211	AT1G76500.1	0	cassava4.1_024479m	Predicted AT-hook DNA-binding family protein	1.420	7.98E-07	2.33E-05
RknMes02_029387	AT5G67350.1	2.00E-38	cassava4.1_014725m	unknown protein; Has 1807 Blast hits to 1807 proteins in 277 species: Archae - 0; Bacteria - 0; M	1.419	1.44E-06	3.37E-05
RknMes02_056167	AT3G63095.1	3.00E-19	cassava4.1_029472m	Tetratricopeptide repeat (TPR)-like superfamily protein///Unknown	1.417	2.82E-06	5.18E-05
RknMes02_026944	AT4G20170.1	7.00E-41	cassava4.1_005572m	Domain of unknown function (DUF23)	1.416	6.83E-06	9.43E-05
RknMes02_004319	AT1G17680.2	8.00E-15	cassava4.1_025348m	tetratricopeptide repeat (TPR)-containing protein	1.416	6.47E-09	1.70E-06
RknMes02_013341	AT1G58440.1	4.00E-06		FAD/NAD(P)-binding oxidoreductase family protein	1.413	1.50E-07	9.04E-06
RknMes02_049061	AT4G02590.2	0	cassava4.1_011595m	basic helix-loop-helix (bHLH) DNA-binding superfamily protein	1.411	1.29E-08	2.47E-06
RknMes02_034375	AT1G21840.1	5.60E-45	cassava4.1_014923m	urease accessory protein F	1.411	2.45E-07	1.17E-05
RknMes02_055949	AT5G08391.1	7.00E-27	cassava4.1_029115m	cassava4.1_033061m///Protein of unknown function (DUF 3339)	1.410	4.45E-08	4.82E-06
RknMes02_006356	AT3G28510.1	0	cassava4.1_005875m	P-loop containing nucleoside triphosphate hydrolases superfamily protein	1.408	4.33E-06	6.93E-05
RknMes02_051546	AT3G58000.1	3.00E-30	cassava4.1_021620m	VQ motif-containing protein	1.407	2.22E-06	4.49E-05
RknMes02_006744	AT3G48990.1	7.00E-42	cassava4.1_005514m	Unknown///AMP-dependent synthetase and ligase family protein	1.406	4.81E-06	7.44E-05
RknMes02_049408	AT5G40670.1	0	cassava4.1_013812m	PQ-loop repeat family protein / transmembrane family protein	1.402	3.74E-06	6.30E-05
RknMes02_014452	AT3G05500.1	1.00E-31	cassava4.1_028020m	Rubber elongation factor protein (REF)	1.402	8.41E-11	2.44E-07
RknMes02_049485	AT4G13720.1	0	cassava4.1_014284m	Inosine triphosphate pyrophosphatase family protein	1.400	1.14E-07	7.87E-06
RknMes02_049371	AT3G60410.3	0	cassava4.1_013601m	Unknown///Protein of unknown function (DUF1639)	1.400	4.26E-10	4.84E-07
RknMes02_052876	AT5G53290.1	2.00E-22	cassava4.1_023899m	Integrase-type DNA-binding superfamily protein	1.400	6.00E-06	8.62E-05
RknMes02_038979	AT1G51130.1	7.00E-06	cassava4.1_009430m	Nse4, component of Smc5/6 DNA repair complex	1.396	1.77E-10	3.17E-07
RknMes02_042540					1.395	4.32E-07	1.60E-05
RknMes02_033846	AT3G29770.1	2.00E-26	cassava4.1_009635m	methyl esterase 11///methyl esterase 15	1.394	2.68E-06	5.00E-05
RknMes02_011031	AT4G01370.1	9.00E-38	cassava4.1_009399m	MAP kinase 4	1.393	1.18E-06	2.96E-05
RknMes02_026293	AT5G64410.1	0	cassava4.1_002179m	oligopeptide transporter 4	1.387	5.52E-06	8.16E-05
RknMes02_057295	AT4G37770.1	0	cassava4.1_031356m	1-amino-cyclopropane-1-carboxylate synthase 8	1.383	2.55E-07	1.20E-05
RknMes02_012112	AT5G28500.1	2.00E-18	cassava4.1_026834m	unknown protein; BEST Arabidopsis thaliana protein match is: unknown protein (TAIR:AT3G04	1.383	5.72E-06	8.35E-05
RknMes02_011815	AT3G51670.1	1.00E-09	cassava4.1_009464m	SEC14 cytosolic factor family protein / phosphoglyceride transfer family protein	1.381	2.03E-06	4.22E-05
RknMes02_031337	AT3G52740.1	2.00E-21	cassava4.1_018233m	AT3G52740.1///unknown protein; BEST Arabidopsis thaliana protein match is: unknown protein	1.379	6.22E-06	8.85E-05
RknMes02_033248	AT1G69935.1	3.00E-36	cassava4.1_016677m	short hypocotyl in white light1	1.379	8.25E-07	2.37E-05
RknMes02_026433	AT2G43710.1	0	cassava4.1_009202m	Plant stearoyl-acyl-carrier-protein desaturase family protein	1.378	1.86E-08	3.04E-06
RknMes02_018236	AT5G17240.1	9.00E-43	cassava4.1_006758m	SET domain group 40	1.375	1.01E-06	2.68E-05
RknMes02_048946	AT1G64770.1	0	cassava4.1_010721m	NDH-dependent cyclic electron flow 1	1.374	4.12E-06	6.71E-05
RknMes02_000391	AT5G42340.1	6.00E-30	cassava4.1_031969m	Plant U-Box 15	1.370	7.34E-06	9.91E-05
RknMes02_051786	AT4G37630.2	7.00E-25	cassava4.1_022030m	cyclin d5;1	1.367	1.19E-06	2.99E-05
RknMes02_023474	AT3G57190.1	0	cassava4.1_027259m	peptide chain release factor, putative	1.364	4.30E-07	1.60E-05
RknMes02_029518	AT1G78380.1	0	cassava4.1_015761m	glutathione S-transferase TAU 19///glutathione S-transferase TAU 22	1.361	2.67E-06	4.99E-05
RknMes02_009762					1.361	2.67E-06	4.99E-05
RknMes02_011200	AT2G24520.1	0	cassava4.1_001102m	H(+)-ATPase 5	1.359	3.96E-07	1.52E-05
RknMes02_054686	AT2G36890.1	0	cassava4.1_026964m	myb domain protein 36///myb domain protein 68	1.358	7.09E-06	9.68E-05
RknMes02_003468			cassava4.1_023635m		1.354	6.94E-06	9.54E-05
RknMes02_049194	AT2G02240.1	0	cassava4.1_012495m	F-box family protein///phloem protein 2-B1	1.353	9.73E-08	7.23E-06
RknMes02_037544	AT1G07180.1	1.00E-34	cassava4.1_005184m	alternative NAD(P)H dehydrogenase 2///alternative NAD(P)H dehydrogenase 1	1.353	3.79E-07	1.49E-05
RknMes02_025694	AT2G24520.1	0	cassava4.1_001102m	H(+)-ATPase 5	1.351	1.30E-06	3.17E-05
RknMes02_054990	AT2G26070.1	0	cassava4.1_027499m	Protein of unknown function (DUF778)	1.346	6.62E-06	9.24E-05
RknMes02_004911	AT5G20320.1	2.00E-24	cassava4.1_033044m	dicer-like 4	1.346	8.37E-08	6.68E-06
RknMes02_004082	AT1G17840.1	1.00E-17	cassava4.1_005391m	white-brown complex homolog protein 11	1.346	5.04E-07	1.73E-05
RknMes02_033883	AT4G24770.1	2.00E-32	cassava4.1_011472m	31-kDa RNA binding protein	1.345	6.66E-08	5.93E-06
RknMes02_005914	AT5G16710.1	2.00E-07	cassava4.1_013773m	dehydroascorbate reductase 1	1.344	8.28E-07	2.38E-05
RknMes02_013700	AT5G47500.1	3.00E-12	cassava4.1_010240m	Pectin lyase-like superfamily protein	1.344	1.99E-06	4.16E-05
RknMes02_031058	AT2G18510.1	4.00E-09		Unknown///RNA-binding (RRM/RBD/RNP motifs) family protein	1.343	1.32E-07	8.49E-06
RknMes02_039414	AT1G50510.1	3.00E-31	cassava4.1_011202m	indigoidine synthase A family protein	1.341	7.77E-07	2.29E-05
RknMes02_001102	AT1G74650.1	1.00E-14	cassava4.1_012505m	myb domain protein 31///myb domain protein 96	1.340	9.36E-07	2.56E-05
RknMes02_040219	AT3G23000.1	4.00E-10	cassava4.1_008412m	CBL-interacting protein kinase 7	1.337	7.63E-07	2.27E-05
RknMes02_001565	AT2G18960.1	0	cassava4.1_001102m	H(+)-ATPase 1///H(+)-ATPase 6	1.337	2.57E-07	1.21E-05
RknMes02_023606	AT4G33220.1	0	cassava4.1_005656m	pectin methylesterase 44	1.336	1.86E-06	4.00E-05
RknMes02_011040	AT2G12190.1	6.00E-10	cassava4.1_032642m	Cytochrome P450 superfamily protein	1.335	6.93E-06	9.52E-05
RknMes02_047584					1.333	1.43E-06	3.35E-05
RknMes02_017720	AT1G32690.1	6.00E-25	cassava4.1_022081m	unknown protein; FUNCTIONS IN: molecular function unknown; INVOLVED IN: biological pr	1.330	2.27E-08	3.36E-06
RknMes02_033621	AT3G01400.1	0	cassava4.1_010775m	ARM repeat superfamily protein	1.327	4.11E-10	4.82E-07
RknMes02_002942	AT2G25430.1	1.00E-25	cassava4.1_027918m	epsin N-terminal homology (ENTH) domain-containing protein / clathrin assembly protein-relate	1.322	2.53E-07	1.20E-05
RknMes02_057855	AT5G63380.1	0	cassava4.1_032243m	Unknown///AMP-dependent synthetase and ligase family protein	1.319	6.06E-08	5.61E-06
RknMes02_021431					1.312	3.48E-07	1.42E-05
RknMes02_025684	AT4G31010.2	2.00E-43	cassava4.1_009417m	RNA-binding CRS1 / YhbY (CRM) domain-containing protein	1.311	3.66E-06	6.21E-05
RknMes02_048824	AT4G34160.1	0	cassava4.1_009900m	CYCLIN D3;2///CYCLIN D3;1	1.311	5.51E-07	1.82E-05
RknMes02_010994	AT4G10760.1	2.00E-31	cassava4.1_002250m	mRNAadenosine methylase	1.311	1.59E-07	9.28E-06
RknMes02_013842	AT5G15450.1	1.00E-09	cassava4.1_029777m	casein lytic proteinase B3	1.309	8.71E-08	6.85E-06
RknMes02_037541	AT3G49600.1	2.00E-35		ubiquitin-specific protease 26	1.302	3.66E-06	6.21E-05
RknMes02_048448	AT5G40010.1	0	cassava4.1_006349m	AAA-ATPase 1	1.301	9.49E-07	2.58E-05
RknMes02_038612	AT2G46915.1	0	cassava4.1_002376m	Protein of unknown function (DUF3754)	1.301	2.81E-07	1.26E-05
RknMes02_004050	AT1G76490.1	0	cassava4.1_003982m	hydroxy methylglutaryl CoA reductase 1	1.298	7.12E-07	2.16E-05
RknMes02_042389	AT4G15733.1	1.00E-09		SCR-like 11	1.295	5.94E-06	8.57E-05
RknMes02_009326	AT3G23600.2	2.00E-06	cassava4.1_015055m	alpha/beta-Hydrolases superfamily protein	1.294	8.07E-07	2.33E-05
RknMes02_031527	AT1G31650.1	3.00E-23	cassava4.1_005718m	RHO guanyl-nucleotide exchange factor 14	1.294	3.18E-07	1.36E-05
RknMes02_001099	AT2G24520.1	0	cassava4.1_001104m	H(+)-ATPase 5	1.294	6.09E-06	8.71E-05
RknMes02_050561	AT3G04620.1	2.00E-33	cassava4.1_019564m	Alba DNA/RNA-binding protein	1.293	1.61E-07	9.31E-06
RknMes02_003718	AT5G19530.2	4.00E-25	cassava4.1_011613m	S-adenosyl-L-methionine-dependent methyltransferases superfamily protein	1.290	4.91E-06	7.55E-05
RknMes02_033310			cassava4.1_007858m		1.290	5.48E-06	8.11E-05
RknMes02_032531	AT4G39330.1	0	cassava4.1_010429m	cinnamyl alcohol dehydrogenase 9	1.287	2.04E-06	4.23E-05
RknMes02_054005	AT5G57560.1	0	cassava4.1_025832m	Xyloglucan endotransglucosylase/hydrolase family protein///xyloglucan endotransglycosylase 6	1.285	2.22E-09	1.12E-06
RknMes02_008267			cassava4.1_011240m		1.285	2.58E-08	3.58E-06
RknMes02_003571	AT5G62350.1	1.00E-19	cassava4.1_024072m	Plant invertase/pectin methylesterase inhibitor superfamily protein	1.284	3.98E-06	6.57E-05
RknMes02_039100	AT1G08830.2	0	cassava4.1_018987m	copper/zinc superoxide dismutase 1	1.283	6.94E-08	6.04E-06
RknMes02_023700	AT3G54430.1	5.00E-11	cassava4.1_013969m	SHI-related sequence 6	1.283	4.58E-07	1.65E-05
RknMes02_035332	AT1G09320.1	2.00E-06	cassava4.1_010435m	agenet domain-containing protein	1.281	3.72E-07	1.47E-05
RknMes02_002824	AT3G01400.1	5.00E-37	cassava4.1_011186m	ARM repeat superfamily protein	1.281	2.69E-09	1.18E-06
RknMes02_022193	AT1G67810.1	1.00E-23	cassava4.1_014023m	sulfur E2	1.281	2.00E-07	1.05E-05
RknMes02_036528	AT1G23400.1	0	cassava4.1_004594m	RNA-binding CRS1 / YhbY (CRM) domain-containing protein	1.275	3.85E-07	1.50E-05
RknMes02_053024	AT3G12650.1	0	cassava4.1_024145m	AT3G12650.1	1.275	4.00E-08	4.51E-06
RknMes02_031630	AT4G36910.1	0	cassava4.1_018773m	Cystathionine beta-synthase (CBS) family protein	1.275	6.15E-10	6.06E-07

RknMes02_036457	AT1G58440.1	6.00E-40	cassava4.1_005294m	FAD/NAD(P)-binding oxidoreductase family protein	1.269	3.45E-06	5.95E-05
RknMes02_021928	AT1G21840.1	3.00E-40	cassava4.1_014923m	urease accessory protein F	1.268	2.54E-07	1.20E-05
RknMes02_031881	AT5G21920.1	0	cassava4.1_014893m	YGGT family protein	1.268	2.80E-07	1.26E-05
RknMes02_026731	AT5G41960.1	2.00E-41	cassava4.1_030596m	unknown protein; FUNCTIONS IN: molecular_function unknown; INVOLVED IN: biological_pr	1.268	2.87E-07	1.28E-05
RknMes02_001771	AT3G48990.1	0	cassava4.1_005514m	Unknown///AMP-dependent synthetase and ligase family protein	1.265	5.13E-06	7.78E-05
RknMes02_048955	AT4G35640.1	0	cassava4.1_010801m	serine acetyltransferase 3;2///para-aminobenzoate (PABA) synthase family protein	1.265	1.85E-06	3.97E-05
RknMes02_048321	AT1G07180.1	0	cassava4.1_004993m	alternative NAD(P)H dehydrogenase 2///alternative NAD(P)H dehydrogenase 1	1.264	5.50E-07	1.82E-05
RknMes02_012544	AT3G19810.1	9.00E-27	cassava4.1_028235m	Protein of unknown function (DUF177)	1.263	7.07E-08	6.10E-06
RknMes02_059073	AT1G12120.1	2.00E-30	cassava4.1_034447m	Plant protein of unknown function (DUF863)	1.262	9.94E-08	7.29E-06
RknMes02_005302	AT1G12100.1	9.00E-23	cassava4.1_032920m	Bifunctional inhibitor/lipid-transfer protein/seed storage 2S albumin superfamily protein	1.261	8.37E-07	2.39E-05
RknMes02_024112	AT3G16000.1	4.10E-44	cassava4.1_027490m	MAR binding filament-like protein 1	1.260	5.84E-06	8.46E-05
RknMes02_033693	AT3G03330.1	0	cassava4.1_024654m	NAD(P)-binding Rossmann-fold superfamily protein	1.259	8.04E-09	1.88E-06
RknMes02_052417	AT1G50910.1	0	cassava4.1_023111m	AT1G50910.1	1.259	4.42E-07	1.62E-05
RknMes02_006731	AT3G48000.1	0	cassava4.1_005092m	aldehyde dehydrogenase 2B4	1.259	2.81E-06	5.17E-05
RknMes02_022462	AT4G39980.1	5.00E-40	cassava4.1_005461m	3-deoxy-d-arabino-heptulosonate 7-phosphate synthase///3-deoxy-D-arabino-heptulosonate 7-pho	1.259	2.61E-07	1.22E-05
RknMes02_048521	AT5G04610.1	0	cassava4.1_007035m	S-adenosyl-L-methionine-dependent methyltransferases superfamily protein	1.258	3.13E-06	5.56E-05
RknMes02_014105	AT3G01400.1	1.00E-06	cassava4.1_010775m	ARM repeat superfamily protein	1.255	2.68E-08	3.67E-06
RknMes02_010015	AT4G21350.1	2.00E-16	cassava4.1_031118m	plant U-box 8	1.255	4.50E-06	7.10E-05
RknMes02_044012	AT2G43420.1	9.00E-06		3-beta hydroxysteroid dehydrogenase/isomerase family protein	1.255	2.34E-06	4.63E-05
RknMes02_036905	AT1G01710.1	0	cassava4.1_008312m	Acyl-CoA thioesterase family protein	1.253	1.16E-06	2.93E-05
RknMes02_039869	AT3G23570.1	0	cassava4.1_015055m	alpha/beta-Hydrolases superfamily protein	1.252	6.29E-08	5.73E-06
RknMes02_002643	AT1G67500.2	1.00E-28	cassava4.1_000079m	recovery protein 3	1.249	1.98E-06	4.15E-05
RknMes02_009439			cassava4.1_014353m		1.246	5.27E-06	7.91E-05
RknMes02_001770	AT5G05690.3	0	cassava4.1_015740m	Cytochrome P450 superfamily protein	1.245	7.21E-08	6.16E-06
RknMes02_020982	AT1G62790.2	8.00E-28	cassava4.1_017979m	receptor serine/threonine kinase, putative	1.243	7.18E-06	9.76E-05
RknMes02_010666	AT5G03450.1	5.00E-09	cassava4.1_023635m	Transducin/WD40 repeat-like superfamily protein	1.243	5.26E-07	1.77E-05
RknMes02_009271	AT5G01270.2	2.00E-26	cassava4.1_021834m	carboxyl-terminal domain (ctd) phosphatase-like 2	1.242	3.13E-07	1.35E-05
RknMes02_050568	AT4G14723.1	2.00E-31	cassava4.1_019595m	allergen-related///BEST Arabidopsis thaliana protein match is: allergen-related (TAIR:AT3G228	1.242	5.95E-06	8.57E-05
RknMes02_034848	AT5G44280.2	8.00E-42	cassava4.1_024012m	RING 1A	1.240	1.25E-08	2.43E-06
RknMes02_038809					1.238	1.85E-07	1.00E-05
RknMes02_003305	AT2G18960.1	7.00E-32	cassava4.1_001104m	H(+)-ATPase 1///H(+)-ATPase 6	1.236	5.63E-06	8.28E-05
RknMes02_003788	AT2G23970.1	9.00E-30	cassava4.1_023112m	Class I glutamine amidotransferase-like superfamily protein	1.235	3.68E-07	1.47E-05
RknMes02_031095	AT5G06410.1	6.00E-43	cassava4.1_024900m	DNAJ heat shock N-terminal domain-containing protein	1.230	1.93E-10	3.36E-07
RknMes02_013312	AT1G31280.1	1.00E-13	cassava4.1_000920m	Argonaute family protein	1.230	1.38E-07	8.73E-06
RknMes02_006336	AT5G20320.2	5.00E-28	cassava4.1_001038m	dicer-like 4	1.229	2.13E-06	4.36E-05
RknMes02_029090	AT3G12780.1	0	cassava4.1_008680m	phosphoglycerate kinase 1///Unknown	1.223	5.34E-08	5.27E-06
RknMes02_035613			cassava4.1_011649m		1.222	3.12E-09	1.24E-06
RknMes02_008115	AT1G67810.1	9.00E-35	cassava4.1_014023m	sulfur E2	1.221	2.00E-06	4.18E-05
RknMes02_013065	AT1G12420.1	4.00E-18	cassava4.1_007717m	ACT domain repeat 8	1.218	4.12E-06	6.71E-05
RknMes02_036575	AT4G33090.1	0	cassava4.1_001498m	aminopeptidase M1	1.217	3.84E-07	1.50E-05
RknMes02_010365	AT1G04620.1	4.00E-11	cassava4.1_007271m	coenzyme F420 hydrogenase family / dehydrogenase, beta subunit family	1.217	2.76E-06	5.12E-05
RknMes02_013142	AT3G48000.1	1.00E-14	cassava4.1_005092m	aldehyde dehydrogenase 2B4	1.216	4.45E-08	4.82E-06
RknMes02_048385	AT3G05990.1	0	cassava4.1_005652m	Leucine-rich repeat (LRR) family protein	1.215	2.41E-07	1.16E-05
RknMes02_046769					1.214	4.92E-06	7.56E-05
RknMes02_013643	AT1G01710.1	3.00E-25	cassava4.1_008312m	Acyl-CoA thioesterase family protein	1.214	1.87E-06	4.01E-05
RknMes02_009064	AT1G70070.1	1.00E-28	cassava4.1_034348m	DEAD/DEAH box helicase, putative	1.212	1.09E-06	2.81E-05
RknMes02_026057	AT2G07715.1	3.00E-08		Nucleic acid-binding, OB-fold-like protein///ribosomal protein L2	1.209	1.06E-06	2.77E-05
RknMes02_017072	AT3G10340.1	0	cassava4.1_003117m	phenylalanine ammonia-lyase 2///phenylalanine ammonia-lyase 4	1.208	1.43E-06	3.35E-05
RknMes02_031569			cassava4.1_011649m		1.206	3.66E-09	1.37E-06
RknMes02_000111	AT3G14860.2	1.00E-23	cassava4.1_006580m	NHL domain-containing protein	1.205	1.33E-07	8.50E-06
RknMes02_034684	AT1G23800.1	0	cassava4.1_005133m	aldehyde dehydrogenase 2B7	1.205	4.36E-06	6.96E-05
RknMes02_008945	AT5G37640.1	4.00E-07		ubiquitin 9///polyubiquitin 10	1.199	4.03E-06	6.61E-05
RknMes02_014112	AT5G12330.4	2.00E-10	cassava4.1_013969m	Lateral root primordium (LRP) protein-related	1.198	4.98E-08	5.03E-06
RknMes02_048962	AT4G35640.1	0	cassava4.1_010831m	serine acetyltransferase 3;2///para-aminobenzoate (PABA) synthase family protein	1.198	1.29E-06	3.15E-05
RknMes02_001318	AT2G42960.1	0	cassava4.1_005944m	Protein kinase superfamily protein	1.197	4.62E-07	1.65E-05
RknMes02_003917	AT2G29630.3	2.00E-30	cassava4.1_003350m	thiaminC	1.197	1.34E-06	3.23E-05
RknMes02_050584	AT5G41960.1	2.00E-12	cassava4.1_019656m	unknown protein; FUNCTIONS IN: molecular_function unknown; INVOLVED IN: biological_pr	1.193	3.57E-07	1.45E-05
RknMes02_006886	AT1G05170.2	7.00E-16	cassava4.1_009046m	Galactosyltransferase family protein	1.191	3.78E-06	6.35E-05
RknMes02_004424	AT5G01270.2	8.00E-23	cassava4.1_021834m	carboxyl-terminal domain (ctd) phosphatase-like 2	1.189	4.19E-07	1.57E-05
RknMes02_011700	AT3G12780.1	2.00E-16	cassava4.1_008680m	phosphoglycerate kinase 1///Unknown	1.188	1.99E-07	1.05E-05
RknMes02_032935	AT5G35110.1	2.00E-19	cassava4.1_019728m	AT5G35110.1///unknown protein; BEST Arabidopsis thaliana protein match is: unknown protein	1.187	2.00E-06	4.18E-05
RknMes02_037379	AT5G17240.1	3.00E-29	cassava4.1_006758m	SET domain group 40	1.186	1.04E-07	7.46E-06
RknMes02_020673	AT2G32710.1	4.00E-11	cassava4.1_014373m	inhibitor/interactor with cyclin-dependent kinase	1.184	9.03E-07	2.50E-05
RknMes02_038051	AT1G70000.2	0	cassava4.1_012449m	Unknown///myb-like transcription factor family protein	1.183	1.32E-07	8.50E-06
RknMes02_022848	AT4G09350.1	0	cassava4.1_014639m	Chaperone DnaJ-domain superfamily protein	1.182	1.44E-06	3.37E-05
RknMes02_025381			cassava4.1_009201m		1.182	6.21E-06	8.84E-05
RknMes02_033876	AT5G20740.1	2.00E-27	cassava4.1_021617m	Plant invertase/pectin methylesterase inhibitor superfamily protein	1.182	2.21E-06	4.47E-05
RknMes02_027463	AT3G11150.1	1.00E-36	cassava4.1_009937m	2-oxoglutarate (2OG) and Fe(II)-dependent oxygenase superfamily protein	1.179	5.59E-07	1.84E-05
RknMes02_027411	AT4G14930.1	0	cassava4.1_012374m	Survival protein SurE-like phosphatase/nucleotidase	1.177	2.93E-06	5.31E-05
RknMes02_025133	AT5G46295.1	5.00E-11	cassava4.1_023391m	unknown protein; BEST Arabidopsis thaliana protein match is: unknown protein (TAIR:AT1G06	1.174	4.16E-06	6.75E-05
RknMes02_009039	AT1G36940.2	2.00E-12	cassava4.1_022208m	unknown protein; FUNCTIONS IN: molecular_function unknown; INVOLVED IN: biological_pr	1.172	1.12E-06	2.88E-05
RknMes02_001970	AT5G66470.1	0	cassava4.1_008321m	RNA binding;GTP binding///Unknown	1.170	1.95E-06	4.11E-05
RknMes02_031259	AT5G65280.1	0	cassava4.1_008284m	GCR2-like 1	1.170	2.72E-07	1.24E-05
RknMes02_049630	AT5G54680.1	0	cassava4.1_015138m	basic helix-loop-helix (bHLH) DNA-binding superfamily protein	1.169	3.54E-08	4.29E-06
RknMes02_028395	AT2G37630.1	2.00E-16	cassava4.1_010610m	myb-like HTH transcriptional regulator family protein	1.165	3.86E-07	1.50E-05
RknMes02_040233	AT3G16520.3	3.00E-11	cassava4.1_006967m	UDP-glucosyl transferase 88A1///cassava4.1_028897m///cassava4.1_019900m///UDP-glucosyl tr	1.165	2.32E-06	4.61E-05
RknMes02_042382	AT5G06410.1	2.00E-12		DNAJ heat shock N-terminal domain-containing protein	1.160	5.15E-07	1.75E-05
RknMes02_004998	AT5G47040.1	1.00E-41	cassava4.1_001387m	lon protease 2	1.158	2.31E-07	1.14E-05
RknMes02_002774	AT1G15290.1	2.00E-06	cassava4.1_034222m	Tetratricopeptide repeat (TPR)-like superfamily protein///Unknown	1.157	1.04E-06	2.74E-05
RknMes02_003326	AT1G35510.1	3.00E-26	cassava4.1_004225m	O-fucosyltransferase family protein	1.156	1.72E-06	3.80E-05
RknMes02_035098	AT1G74970.1	0	cassava4.1_016342m	ribosomal protein S9	1.156	2.32E-07	1.14E-05
RknMes02_045249	AT1G21980.1	2.00E-14	cassava4.1_017511m	phosphatidylinositol-4-phosphate 5-kinase 1	1.156	2.85E-07	1.27E-05
RknMes02_029834	AT2G03170.1	3.00E-24	cassava4.1_024765m	SKP1-like 14	1.154	2.29E-06	4.58E-05
RknMes02_006330	AT4G10760.1	0	cassava4.1_002250m	mRNAadenosine methylase	1.153	1.77E-07	9.85E-06
RknMes02_034967	AT5G66920.1	0	cassava4.1_005181m	SKU5 similar 17	1.152	6.04E-06	8.66E-05
RknMes02_025934	AT3G52180.2	0	cassava4.1_009735m	dual specificity protein phosphatase (DsPTP1) family protein	1.150	1.63E-07	9.37E-06
RknMes02_000259	AT4G10160.1	1.00E-08	cassava4.1_021144m	RING/U-box superfamily protein	1.148	7.03E-06	9.62E-05
RknMes02_037296			cassava4.1_019664m		1.146	1.16E-07	7.88E-06
RknMes02_010731	AT4G28080.1	7.00E-33	cassava4.1_034222m	Tetratricopeptide repeat (TPR)-like superfamily protein	1.145	2.24E-06	4.51E-05
RknMes02_048844	AT1G13270.1	0	cassava4.1_010044m	methionine aminopeptidase 1B	1.145	2.64E-09	1.18E-06
RknMes02_032978	AT5G13030.1	0	cassava4.1_004303m	unknown protein; FUNCTIONS IN: molecular_function unknown; INVOLVED IN: biological_pr	1.144	1.27E-08	2.45E-06
RknMes02_047966	AT5G52950.1	0	cassava4.1_000901m	AT5G52950.1	1.143	1.03E-06	2.71E-05
RknMes02_008154	AT2G39570.1	8.00E-41	cassava4.1_008379m	ACT domain-containing protein	1.142	1.10E-06	2.83E-05
RknMes02_005354	AT3G53010.1	0	cassava4.1_012755m	Domain of unknown function (DUF303)	1.140	1.56E-07	9.23E-06
RknMes02_054765	AT1G18750.1	0	cassava4.1_027107m	AGAMOUS-like 65	1.138	1.17E-09	8.41E-07
RknMes02_051840	AT3G09280.1	1.00E-12	cassava4.1_022123m	AT3G09280.1///Unknown	1.137	9.67E-07	2.61E-05
RknMes02_058901	AT4G26200.1	0	cassava4.1_033963m	1-amino-cyclopropane-1-carboxylate synthase 7	1.137	6.10E-06	8.73E-05
RknMes02_048166	AT2G46040.1	0	cassava4.1_003489m	ARID/BRIGHT DNA-binding domain;ELM2 domain protein	1.136	1.86E-06	3.99E-05
RknMes02_010606	AT1G52870.2	3.00E-06		Peroxisomal membrane 22 kDa (Mpv17/PMP22) family protein	1.136	2.82E-06	5.18E-05
RknMes02_007602	AT5G64970.1	2.00E-30	cassava4.1_025172m	Mitochondrial substrate carrier family protein	1.136	5.39E-08	5.27E-06
RknMes02_036309	AT2G01290.1	0	cassava4.1_014064m	ribose-5-phosphate isomerase 2	1.136	4.10E-06	6.69E-05
RknMes02_005847	AT3G05820.1	0	cassava4.1_002971m	invertase H	1.134	3.40E-08	4.19E-06
RknMes02_052543	AT1G53025.1	0	cassava4.1_023333m	ubiquitin-conjugating enzyme 23	1.133	6.20E-06	8.83E-05
RknMes02_026917	AT4G33090.1	0	cassava4.1_001499m	aminopeptidase M1	1.133	3.42E-06	5.91E-05
RknMes02_003586	AT4G20360.1	1.00E-39	cassava4.1_028531m	RAB GTPase homolog E1B///Unknown///"Nucleic acid-binding, OB-fold-like protein"	1.133	2.85E-08	3.79E-06
RknMes02_000598	AT4G30210.2	7.00E-43	cassava4.1_006173m	P450 reductase 2	1.131	2.22E-07	1.11E-05
RknMes02_033830	AT5G44400.1	0	cassava4.1_004979m	FAD-binding Berberine family protein	1.130	3.87E-07	1.50E-05
RknMes02_048827	AT3G51820.1	0	cassava4.1_009920m	UbiA prenyltransferase family protein	1.130	2.94E-09	1.21E-06
RknMes02_000991					1.129	4.88E-08	4.97E-06
RknMes02_003198	AT5G15450.1	0	cassava4.1_001040m	casein lytic proteinase B3	1.128	1.58E-07	9.27E-06
RknMes02_058926	AT2G29420.1	0	cassava4.1_034007m	glutathione S-transferase tau 7///glutathione S-transferase TAU 1	1.128	1.14E-06	2.90E-05
RknMes02_058917	AT1G23550.1	0	cassava4.1_033992m	similar to RCD one 2	1.127	5.41E-06	8.05E-05
RknMes02_000612	AT2G37630.1	2.00E-31	cassava4.1_010610m	myb-like HTH transcriptional regulator family protein	1.124	4.00E-08	4.51E-06
RknMes02_038791	AT1G22710.1	8.00E-17	cassava4.1_007460m	sucrose-proton symporter 2	1.124	1.00E-10	2.44E-07
RknMes02_033921	AT4G25600.1	0	cassava4.1_016238m	Oxoglutarate/iron-dependent oxygenase	1.123	8.97E-07	2.49E-05
RknMes02_001481			cassava4.1_002945m		1.122	1.62E-10	2.99E-07
RknMes02_027619	AT1G60420.1	0	cassava4.1_004565m	DC1 domain-containing protein	1.122	2.83E-07	1.27E-05
RknMes02_021704	AT3G16000.1	2.00E-33	cassava4.1_027490m	MAR binding filament-like protein 1	1.118	6.09E-06	8.71E-05
RknMes02_037861	AT1G04620.1	0	cassava4.1_007271m	coenzyme F420 hydrogenase family / dehydrogenase, beta subunit family	1.116	4.65E-07	1.66E-05
RknMes02_031979	AT4G10760.1	2.00E-43	cassava4.1_002250m	mRNAadenosine methylase	1.113	1.19E-06	2.99E-05
RknMes02_001173	AT5G23060.1	0	cassava4.1_009468m	calcium sensing receptor	1.112	5.96E-07	1.91E-05
RknMes02_044305	AT5G41980.1	0		CONTAINS InterPro DOMAIN/s: Putative harbinger transposase-derived nuclease (InterPro:IPR0	1.111	1.59E-06	3.59E-05
RknMes02_049203	AT3G60390.1	0	cassava4.1_012590m	homeobox-leucine zipper protein 3///homeobox-leucine zipper protein 4	1.109	1.19E-09	8.41E-07
RknMes02_026116	AT5G41370.1	0	cassava4.1_002131m	homolog of xeroderma pigmentosum complementation group B 1	1.106	6.17E-07	1.95E-05
RknMes02_053965	AT2G01900.1	0	cassava4.1_025766m	DNAse I-like superfamily protein	1.106	7.73E-08	6.33E-06
RknMes02_032915	AT1G13570.1	1.00E-22	cassava4.1_007904m	F-box/RNI-like superfamily protein	1.105	1.96E-06	4.13E-05
RknMes02_000071	AT1G67840.1	2.00E-40	cassava4.1_003242m	chloroplast sensor kinase	1.104	1.31E-06	3.18E-05

RknMes02_008789			cassava4.1_015534m		1.104	7.55E-08	6.25E-06
RknMes02_038125	AT2G04842.1	0	cassava4.1_023900m	threonyl-tRNA synthetase, putative / threonine--tRNA ligase, putative	1.103	9.41E-07	2.57E-05
RknMes02_014850			cassava4.1_028335m		1.103	1.33E-06	3.22E-05
RknMes02_038894	AT1G77280.1	0	cassava4.1_002273m	Protein kinase protein with adenine nucleotide alpha hydrolases-like domain	1.103	4.47E-06	7.07E-05
RknMes02_006302	AT3G15880.3	6.00E-18	cassava4.1_000558m	Transducin family protein / WD-40 repeat family protein///WUS-interacting protein 2	1.101	2.55E-08	3.56E-06
RknMes02_032677					1.100	1.45E-06	3.38E-05
RknMes02_017340	AT1G58170.1	9.00E-36	cassava4.1_031388m	Disease resistance-responsive (dirigent-like protein) family protein	1.099	4.67E-06	7.28E-05
RknMes02_030087	AT4G34230.1	0	cassava4.1_012822m	GroES-like zinc-binding alcohol dehydrogenase family protein///cinnamyl alcohol dehydrogenase	1.099	3.05E-07	1.33E-05
RknMes02_013636	AT5G34930.1	3.00E-19	cassava4.1_003968m	prephenate dehydrogenase family protein///arogenate dehydrogenase	1.097	7.80E-08	6.38E-06
RknMes02_036565	AT5G15450.1	1.00E-30	cassava4.1_001040m	casein lytic proteinase B3	1.096	2.12E-09	1.12E-06
RknMes02_023193	AT2G39140.1	7.00E-35	cassava4.1_009165m	pseudouridine synthase family protein	1.096	3.68E-06	6.23E-05
RknMes02_055626	AT1G48960.1	0	cassava4.1_028587m	Adenine nucleotide alpha hydrolases-like superfamily protein	1.095	2.39E-07	1.16E-05
RknMes02_002526	AT1G01710.1	6.00E-15	cassava4.1_008312m	Acyl-CoA thioesterase family protein	1.092	4.13E-06	6.72E-05
RknMes02_016693	AT5G14680.1	0	cassava4.1_017511m	Adenine nucleotide alpha hydrolases-like superfamily protein	1.092	4.18E-07	1.57E-05
RknMes02_027515	AT3G04560.1	0	cassava4.1_008870m	unknown protein; FUNCTIONS IN: molecular function unknown; INVOLVED IN: biological pr	1.091	1.92E-07	1.03E-05
RknMes02_052928	AT4G31930.1	1.00E-12	cassava4.1_023980m	Mitochondrial glycoprotein family protein	1.090	3.00E-06	5.39E-05
RknMes02_031570	AT2G28680.1	0	cassava4.1_010513m	RmlC-like cupins superfamily protein	1.090	7.89E-08	6.42E-06
RknMes02_049644	AT2G01890.1	0	cassava4.1_015221m	purple acid phosphatase 8///purple acid phosphatase 3	1.090	1.49E-06	3.44E-05
RknMes02_032171	AT5G01270.2	2.00E-44	cassava4.1_021834m	carboxyl-terminal domain (ctd) phosphatase-like 2	1.089	2.64E-07	1.23E-05
RknMes02_035830	AT1G15290.1	1.00E-16	cassava4.1_034222m	Tetratricopeptide repeat (TPR)-like superfamily protein///Unknown	1.089	1.47E-06	3.41E-05
RknMes02_049538	AT1G74650.1	0	cassava4.1_014624m	myb domain protein 31///myb domain protein 96	1.085	1.26E-08	2.44E-06
RknMes02_034125	AT5G50870.1	0	cassava4.1_016816m	ubiquitin-conjugating enzyme 27	1.084	2.11E-06	4.33E-05
RknMes02_001933	AT5G67500.2	9.00E-25	cassava4.1_026681m	voltage dependent anion channel 2	1.084	3.77E-07	1.48E-05
RknMes02_014581					1.083	6.68E-06	9.30E-05
RknMes02_032985	AT5G63060.1	1.00E-36	cassava4.1_014212m	Sec14p-like phosphatidylinositol transfer family protein	1.082	1.59E-07	9.28E-06
RknMes02_001892	AT4G13930.1	0	cassava4.1_006915m	serine hydroxymethyltransferase 4	1.082	4.96E-07	1.72E-05
RknMes02_024960	AT1G22130.1	7.00E-31	cassava4.1_015534m	AGAMOUS-like 104	1.081	2.11E-06	4.33E-05
RknMes02_049971	AT4G01900.1	0	cassava4.1_016781m	GLNB1 homolog	1.080	8.78E-07	2.47E-05
RknMes02_024785	AT5G11790.1	0	cassava4.1_010943m	N-MYC downregulated-like 2	1.080	3.60E-06	6.13E-05
RknMes02_048312	AT1G17680.2	0	cassava4.1_004904m	tetratricopeptide repeat (TPR)-containing protein	1.078	1.30E-07	8.40E-06
RknMes02_032161	AT1G73060.1	0	cassava4.1_027070m	Low PSII Accumulation 3	1.078	4.06E-06	6.65E-05
RknMes02_004418	AT5G11790.1	0	cassava4.1_010943m	N-MYC downregulated-like 2	1.078	3.60E-06	6.13E-05
RknMes02_000130	AT3G60390.1	2.00E-37	cassava4.1_023832m	homeobox-leucine zipper protein 3///homeobox-leucine zipper protein 4	1.077	4.10E-08	4.57E-06
RknMes02_048776	AT2G19810.1	0	cassava4.1_009450m	Zinc finger C-x8-C-x5-C-x3-H type family protein///CCCH-type zinc finger family protein	1.074	4.21E-07	1.58E-05
RknMes02_033590	AT2G33610.1	0	cassava4.1_007232m	switch subunit 3	1.074	1.08E-07	7.65E-06
RknMes02_049368	AT2G34260.1	0	cassava4.1_013590m	transducin family protein / WD-40 repeat family protein	1.074	5.89E-09	1.65E-06
RknMes02_038099	AT2G38280.2	0	cassava4.1_001581m	AMP deaminase, putative / myoadenylate deaminase, putative	1.073	8.82E-09	2.01E-06
RknMes02_031679	AT1G71880.1	5.00E-19	cassava4.1_004111m	sucrose-proton symporter 2	1.071	1.55E-06	3.53E-05
RknMes02_032007	AT1G02860.2	0	cassava4.1_011736m	SPX (SYG1/Pho81/XPR1) domain-containing protein	1.070	2.51E-08	3.54E-06
RknMes02_000950	AT5G15450.1	0	cassava4.1_001040m	casein lytic proteinase B3	1.069	5.43E-09	1.60E-06
RknMes02_050283	AT5G14680.1	0	cassava4.1_018282m	Adenine nucleotide alpha hydrolases-like superfamily protein	1.069	2.95E-07	1.30E-05
RknMes02_032220	AT4G24770.1	0	cassava4.1_011472m	31-kDa RNA binding protein	1.069	8.61E-07	2.44E-05
RknMes02_050293	AT5G20670.1	3.00E-38	cassava4.1_018327m	Protein of unknown function (DUF1677)	1.069	5.52E-08	5.34E-06
RknMes02_026766	AT5G49950.1	0	cassava4.1_004523m	alpha/beta-Hydrolases superfamily protein	1.068	2.44E-06	4.75E-05
RknMes02_052116	AT1G13680.1	0	cassava4.1_022597m	PLC-like phosphodiesterases superfamily protein	1.067	2.59E-06	4.90E-05
RknMes02_002882	AT1G67070.1	6.00E-23	cassava4.1_007953m	Mannose-6-phosphate isomerase, type I	1.066	1.33E-07	8.50E-06
RknMes02_025177	AT5G33280.1	0	cassava4.1_006977m	Voltage-gated chloride channel family protein	1.065	3.08E-11	1.66E-07
RknMes02_016203			cassava4.1_018507m		1.064	1.71E-08	2.91E-06
RknMes02_049026	AT1G76270.1	0	cassava4.1_011273m	O-fucosyltransferase family protein	1.063	2.15E-07	1.09E-05
RknMes02_025409	AT4G30210.2	5.00E-40	cassava4.1_006173m	P450 reductase 2	1.063	1.22E-06	3.03E-05
RknMes02_034385	AT1G54520.1	2.00E-34	cassava4.1_017125m	Unknown///unknown protein; FUNCTIONS IN: molecular function unknown; INVOLVED IN: b	1.062	5.16E-06	7.80E-05
RknMes02_001446	AT2G47800.1	3.00E-32	cassava4.1_000205m	multidrug resistance-associated protein 5///multidrug resistance-associated protein 4	1.061	1.13E-07	7.83E-06
RknMes02_004203	AT2G23890.1	1.00E-27	cassava4.1_033221m	HAD-superfamily hydrolase, subfamily IG, 5\'-nucleotidase///"HAD-superfamily hydrolase, subfa	1.060	1.35E-06	3.25E-05
RknMes02_038098	AT1G12200.1	2.00E-21	cassava4.1_021346m	Flavin-binding monooxygenase family protein	1.058	7.13E-06	9.72E-05
RknMes02_039382	AT2G37630.1	0	cassava4.1_010610m	myb-like HTH transcriptional regulator family protein	1.058	1.12E-08	2.28E-06
RknMes02_004093	AT3G10340.1	3.00E-12	cassava4.1_003117m	phenylalanine ammonia-lyase 2///phenylalanine ammonia-lyase 4	1.057	2.55E-06	4.86E-05
RknMes02_024608	AT3G04620.1	0	cassava4.1_023523m	Alba DNA/RNA-binding protein	1.055	1.13E-06	2.89E-05
RknMes02_029935	AT4G34480.1	0	cassava4.1_007302m	O-Glycosyl hydrolases family 17 protein///Glycosyl hydrolase family 17 protein	1.054	1.21E-06	3.02E-05
RknMes02_008884	AT5G15450.1	0	cassava4.1_001040m	casein lytic proteinase B3	1.053	5.95E-08	5.55E-06
RknMes02_055219	AT3G01600.1	0	cassava4.1_027882m	NAC (No Apical Meristem) domain transcriptional regulator superfamily protein///NAC domain	1.052	1.16E-08	2.33E-06
RknMes02_027818	AT4G30950.1	3.00E-35	cassava4.1_005244m	fatty acid desaturase 6	1.051	4.75E-07	1.68E-05
RknMes02_037016	AT3G26300.1	0	cassava4.1_005635m	cytochrome P450, family 71, subfamily B, polypeptide 34///"cytochrome P450, family 71, subfam	1.050	2.30E-06	4.58E-05
RknMes02_049155	AT5G04490.1	0	cassava4.1_012212m	vitamin E pathway gene 5	1.049	4.60E-06	7.21E-05
RknMes02_032589	AT2G46370.4	0	cassava4.1_004899m	Auxin-responsive GH3 family protein	1.049	4.69E-06	7.30E-05
RknMes02_049807	AT2G04690.1	0	cassava4.1_016027m	Pyridoxamine 5\'-phosphate oxidase family protein	1.048	2.94E-07	1.30E-05
RknMes02_015902	AT2G43980.1	2.00E-17	cassava4.1_006347m	inositol 1,3,4-trisphosphate 5/6-kinase 4	1.044	7.05E-06	9.64E-05
RknMes02_039352	AT1G76990.5	0	cassava4.1_007514m	ACT domain repeat 3	1.043	7.68E-07	2.27E-05
RknMes02_031924	AT3G46550.1	0	cassava4.1_025211m	Fasciclin-like arabinogalactan family protein	1.043	2.47E-07	1.18E-05
RknMes02_002414	AT3G47420.1	2.00E-11	cassava4.1_005536m	phosphate starvation-induced gene 3	1.043	8.32E-09	1.93E-06
RknMes02_047242					1.043	1.41E-06	3.33E-05
RknMes02_032488	AT4G30210.2	0	cassava4.1_006173m	P450 reductase 2	1.042	4.39E-07	1.62E-05
RknMes02_015002	AT1G08510.1	6.00E-22	cassava4.1_008444m	fatty acyl-ACP thioesterases B	1.042	3.71E-07	1.47E-05
RknMes02_010499	AT3G03530.1	3.00E-31	cassava4.1_005614m	non-specific phospholipase C4	1.041	1.47E-07	9.03E-06
RknMes02_034334			cassava4.1_018004m		1.040	9.81E-07	2.63E-05
RknMes02_005174	AT3G15880.2	2.00E-31	cassava4.1_000547m	Transducin family protein / WD-40 repeat family protein///WUS-interacting protein 2	1.040	2.89E-06	5.27E-05
RknMes02_058943	AT3G02340.1	3.00E-35	cassava4.1_034045m	RING/U-box superfamily protein	1.039	6.29E-06	8.91E-05
RknMes02_000576	AT3G15880.3	9.00E-18	cassava4.1_000550m	Transducin family protein / WD-40 repeat family protein///WUS-interacting protein 2	1.039	8.28E-07	2.38E-05
RknMes02_004185	AT3G52380.1	1.00E-14	cassava4.1_011206m	chloroplast RNA-binding protein 33	1.037	3.08E-08	3.95E-06
RknMes02_054995	AT5G19790.1	3.00E-24	cassava4.1_027506m	related to AP2 11	1.037	5.84E-06	8.46E-05
RknMes02_058101	AT3G53690.1	0	cassava4.1_032629m	RING/U-box superfamily protein	1.037	2.09E-06	4.30E-05
RknMes02_000350	AT1G36990.1	2.00E-21	cassava4.1_022907m	AT1G36990.1///unknown protein; LOCATED IN: chloroplast; EXPRESSED IN: 24 plant structu	1.037	3.72E-07	1.47E-05
RknMes02_003176	AT1G26160.1	4.00E-16	cassava4.1_014119m	CONTAINS InterPro DOMAIN/s: F-box domain, Skp2-like (InterPro:IPR022364), FIST C domai	1.037	2.65E-06	4.97E-05
RknMes02_024456	AT4G16070.2	5.60E-45	cassava4.1_003487m	Mono-/di-acylglycerol lipase, N-terminal;Lipase, class 3	1.036	3.23E-07	1.37E-05
RknMes02_055529	AT4G31420.1	0	cassava4.1_028415m	Zinc finger protein 622	1.034	3.15E-06	5.60E-05
RknMes02_031511	AT2G38450.1	4.00E-37	cassava4.1_018364m	CONTAINS InterPro DOMAIN/s: Sel1-like (InterPro:IPR006597); BEST Arabidopsis thaliana pr	1.032	1.50E-07	9.04E-06
RknMes02_010383	AT1G13270.1	1.00E-19	cassava4.1_010044m	methionine aminopeptidase 1B	1.030	5.62E-09	1.60E-06
RknMes02_006178			cassava4.1_003655m		1.029	1.20E-07	8.01E-06
RknMes02_037640	AT1G26300.1	1.00E-15	cassava4.1_011324m	Unknown	1.027	1.75E-07	9.80E-06
RknMes02_038911	AT3G13120.2	3.00E-34	cassava4.1_016092m	Unknown///Ribosomal protein S10p/S20e family protein	1.026	3.28E-06	5.74E-05
RknMes02_001879	AT1G65980.1	7.00E-35	cassava4.1_017973m	thioredoxin-dependent peroxidase 1	1.026	3.63E-06	6.16E-05
RknMes02_021226			cassava4.1_001423m		1.023	6.77E-08	5.96E-06
RknMes02_035114	AT1G06320.1	4.00E-16	cassava4.1_017107m	unknown protein; Has 24 Blast hits to 24 proteins in 10 species: Archae - 0; Bacteria - 2; Metazo	1.023	4.09E-06	6.68E-05
RknMes02_014796	AT1G79550.2	3.00E-12	cassava4.1_006605m	phosphoglycerate kinase	1.022	4.55E-07	1.65E-05
RknMes02_004682	AT2G35510.1	3.00E-25	cassava4.1_007563m	WWE protein-protein interaction domain protein family///similar to RCD one 1	1.021	8.45E-07	2.41E-05
RknMes02_002603	AT1G70000.2	5.00E-33	cassava4.1_012449m	Unknown///myb-like transcription factor family protein	1.021	2.78E-06	5.14E-05
RknMes02_010062	AT1G80490.2	1.00E-15	cassava4.1_000550m	TOPLESS-related 1///Transducin family protein / WD-40 repeat family protein	1.019	5.02E-07	1.73E-05
RknMes02_033307	AT2G39770.2	0	cassava4.1_010388m	Glucose-1-phosphate adenylyltransferase family protein	1.018	3.75E-06	6.31E-05
RknMes02_048712	AT3G63270.1	0	cassava4.1_008892m	AT3G63270.1	1.018	2.15E-07	1.09E-05
RknMes02_007057	AT2G24260.1	3.00E-19	cassava4.1_012570m	LJRHL1-like 1	1.018	5.19E-06	7.84E-05
RknMes02_039898	AT4G30950.1	0	cassava4.1_005244m	fatty acid desaturase 6	1.017	6.76E-08	5.96E-06
RknMes02_047925	AT3G18100.1	0	cassava4.1_000461m	myb domain protein 4r1	1.016	2.08E-06	4.29E-05
RknMes02_012098			cassava4.1_010060m		1.016	5.24E-09	1.58E-06
RknMes02_009729	AT2G38280.2	0	cassava4.1_001581m	AMP deaminase, putative / myoadenylate deaminase, putative	1.015	3.48E-07	1.42E-05
RknMes02_048886	AT2G26100.1	0	cassava4.1_010336m	Galactosyltransferase family protein	1.014	1.51E-07	9.06E-06
RknMes02_026255	AT4G34350.1	0	cassava4.1_007171m	4-hydroxy-3-methylbut-2-enyl diphosphate reductase	1.014	2.78E-06	5.14E-05
RknMes02_048659	AT2G27430.1	0	cassava4.1_008413m	ARM repeat superfamily protein	1.013	3.63E-07	1.46E-05
RknMes02_039844	AT1G61667.1	0	cassava4.1_017671m	Protein of unknown function, DUF538	1.013	8.09E-08	6.48E-06
RknMes02_034407	AT2G39950.2	4.00E-19	cassava4.1_003002m	unknown protein; FUNCTIONS IN: molecular function unknown; INVOLVED IN: biological pr	1.011	7.22E-06	9.80E-05
RknMes02_053096	AT5G45320.1	0	cassava4.1_024278m	AT5G45320.1	1.011	1.79E-06	3.89E-05
RknMes02_052747	AT3G43540.2	0	cassava4.1_023671m	Protein of unknown function (DUF1350)	1.010	2.61E-07	1.22E-05
RknMes02_018428	AT3G47420.1	4.00E-11	cassava4.1_005536m	phosphate starvation-induced gene 3	1.009	1.49E-07	9.04E-06
RknMes02_005231	AT2G02090.1	2.00E-28	cassava4.1_002042m	SNF2 domain-containing protein / helicase domain-containing protein	1.009	3.91E-07	1.51E-05
RknMes02_014727	AT4G17510.1	7.00E-10	cassava4.1_034222m	ubiquitin C-terminal hydrolase 3	1.004	6.98E-06	9.57E-05
RknMes02_003201	AT2G38280.2	2.00E-40	cassava4.1_001581m	AMP deaminase, putative / myoadenylate deaminase, putative	1.001	3.58E-08	4.31E-06

1) AGI code is shown if proteins encoded in each cassava gene (probe ID) have high amino acid sequence similarity (E value $\leq 10^{-5}$) to *Arabidopsis* homologs.

2) E-value shows similarity in amino acid sequence between each cassava gene (probe ID) and *Arabidopsis* homolog.

3) Encoded proteins/other features indicate the putative functions of the gene products that are expected from sequence similarity. The information for the NCBI protein reference sequence with the highest sequence similarity to the probes is shown.

TABLE S5. Genes up-regulated in cassava roots by SAHA treatment under 24 h NaCl

Probe ID	AGI code[1)]	E-value[2)]	Cassava ID	Encoded proteins/other features[3)]	log_2 ratio {(NaCl 24 h after SAHA 24 h)/(NaCl 24 h after non-SAHA 24 h)}	p-value	BH FDR
RknMes02_057300	AT2G13810.1	0	cassava4.1_031362m	AGD2-like defense response protein 1	7.049	2.31E-09	1.13E-06
RknMes02_058115	AT3G07390.1	0	cassava4.1_032657m	auxin-responsive family protein///Auxin-responsive family protein	5.000	1.10E-06	2.84E-05
RknMes02_051874	AT1G13280.1	0	cassava4.1_022180m	allene oxide cyclase 4	4.707	3.51E-11	1.70E-07
RknMes02_057486	AT2G21220.1	1.00E-35	cassava4.1_031669m	SAUR-like auxin-responsive protein family	4.596	1.51E-10	2.98E-07
RknMes02_052316	AT4G01950.1	0	cassava4.1_022947m	glycerol-3-phosphate acyltransferase 3	4.487	1.28E-07	8.32E-06
RknMes02_051551	AT2G33480.2	5.00E-10	cassava4.1_021626m	NAC domain containing protein 52	4.457	2.96E-08	3.89E-06
RknMes02_039148			cassava4.1_002717m		4.044	2.31E-09	1.13E-06
RknMes02_058141	AT3G06720.2	0	cassava4.1_032699m	importin alpha isoform 1	4.044	1.79E-09	1.01E-06
RknMes02_055141	AT2G40210.1	5.00E-33	cassava4.1_027752m	AGAMOUS-like 48	3.809	2.60E-09	1.18E-06
RknMes02_055406	AT5G17350.1	4.00E-27	cassava4.1_028188m	AT5G17350.1///AT3G03280.1	3.765	9.10E-12	1.08E-07
RknMes02_057007	AT5G57620.1	0	cassava4.1_030900m	myb domain protein 36	3.757	1.46E-06	3.40E-05
RknMes02_056640	AT3G11930.1	0	cassava4.1_030279m	Adenine nucleotide alpha hydrolases-like superfamily protein	3.720	2.04E-08	3.24E-06
RknMes02_028787	AT1G19640.1	2.00E-12	cassava4.1_010155m	S-adenosyl-L-methionine-dependent methyltransferases superfamily protein///j	3.695	3.04E-08	3.94E-06
RknMes02_057539	AT5G07610.1	3.00E-08	cassava4.1_031747m	F-box family protein	3.609	7.51E-11	2.44E-07
RknMes02_054063	AT1G14440.2	2.80E-45	cassava4.1_025934m	homeobox protein 33///homeobox protein 31	3.598	4.87E-06	7.51E-05
RknMes02_016408	AT4G36470.1	6.00E-09	cassava4.1_010155m	S-adenosyl-L-methionine-dependent methyltransferases superfamily protein	3.550	1.47E-07	9.03E-06
RknMes02_052163	AT2G22840.1	0	cassava4.1_022687m	growth-regulating factor 1	3.540	1.70E-06	3.76E-05
RknMes02_053062	AT1G68390.1	0	cassava4.1_024214m	Core-2/I-branching beta-1,6-N-acetylglucosaminyltransferase family protein	3.491	7.05E-10	6.51E-07
RknMes02_053782	AT4G17380.1	0	cassava4.1_025428m	MUTS-like protein 4	3.320	3.11E-07	1.34E-05
RknMes02_055845	AT3G51880.4	6.00E-16	cassava4.1_028951m	high mobility group B2///high mobility group B1	3.292	3.24E-07	1.37E-05
RknMes02_050833	AT4G39250.1	2.00E-32	cassava4.1_020375m	RAD-like 1///RAD-like 6	3.292	4.47E-09	1.48E-06
RknMes02_054668	AT4G36740.1	0	cassava4.1_026937m	homeobox protein 40///homeobox protein 21	3.282	4.30E-09	1.46E-06
RknMes02_055721	AT4G39250.1	3.00E-36	cassava4.1_028739m	RAD-like 1///RAD-like 6	3.243	9.54E-10	7.62E-07
RknMes02_001711	AT4G32460.2	1.00E-19	cassava4.1_010077m	Protein of unknown function, DUF642	3.224	2.31E-09	1.13E-06
RknMes02_058688	AT3G28880.1	0	cassava4.1_033616m	Ankyrin repeat family protein	3.222	2.98E-10	4.22E-07
RknMes02_001781	AT3G58110.2	3.00E-28	cassava4.1_004503m	unknown protein; FUNCTIONS IN: molecular_function unknown; INVOLVE	3.199	3.51E-09	1.34E-06
RknMes02_044414					3.137	1.08E-06	2.80E-05
RknMes02_054888	AT1G01690.1	0	cassava4.1_027325m	putative recombination initiation defects 3	3.128	6.03E-08	5.59E-06
RknMes02_037654	AT4G29110.1	1.00E-07	cassava4.1_016277m	unknown protein; FUNCTIONS IN: molecular_function unknown; INVOLVE	2.989	9.47E-08	7.18E-06
RknMes02_058010	AT5G07610.1	1.00E-16	cassava4.1_032488m	F-box family protein	2.982	1.18E-07	7.95E-06
RknMes02_055397	AT2G26140.1	0	cassava4.1_028173m	FTSH protease 4	2.940	9.44E-08	7.16E-06
RknMes02_052664	AT5G64310.1	9.00E-15	cassava4.1_023538m	Unknown///arabinogalactan protein 1///cassava4.1_023538m	2.918	2.67E-06	4.99E-05
RknMes02_001499	AT5G54160.1	1.00E-21	cassava4.1_013376m	O-methyltransferase 1	2.918	6.28E-06	8.90E-05
RknMes02_012269	AT1G06460.1	2.00E-10	cassava4.1_015713m	alpha-crystallin domain 32.1	2.841	4.63E-06	7.23E-05
RknMes02_051562	AT5G64360.4	2.00E-13	cassava4.1_021648m	Chaperone DnaJ-domain superfamily protein	2.832	2.89E-12	5.97E-08
RknMes02_014532	AT1G17020.1	2.00E-22	cassava4.1_010623m	2-oxoglutarate (2OG) and Fe(II)-dependent oxygenase superfamily protein///se	2.798	1.64E-06	3.67E-05
RknMes02_051803	AT3G09270.1	3.00E-11	cassava4.1_022056m	glutathione S-transferase TAU 8///glutathione S-transferase tau 7	2.770	2.91E-07	1.29E-05
RknMes02_006016			cassava4.1_015713m		2.766	1.47E-07	9.03E-06
RknMes02_039174	AT1G17020.1	3.00E-28	cassava4.1_010623m	2-oxoglutarate (2OG) and Fe(II)-dependent oxygenase superfamily protein///se	2.710	1.29E-06	3.15E-05
RknMes02_051840	AT3G09280.1	1.00E-12	cassava4.1_022123m	AT3G09280.1///Unknown	2.697	9.67E-07	2.61E-05
RknMes02_013200	AT4G34131.1	3.00E-08	cassava4.1_029076m	UDP-glucosyl transferase 73B3	2.665	2.09E-08	3.27E-06
RknMes02_019058	AT1G80050.1	3.00E-18		adenine phosphoribosyl transferase 2	2.655	2.43E-08	3.44E-06
RknMes02_012739	AT2G18550.1	3.00E-32	cassava4.1_027319m	homeobox protein 21	2.639	1.32E-08	2.53E-06
RknMes02_057627	AT4G32480.1	0	cassava4.1_031890m	Protein of unknown function (DUF506)	2.598	4.63E-07	1.66E-05
RknMes02_055925	AT4G34135.1	0	cassava4.1_029076m	UDP-glucosyltransferase 73B2///UDP-glucosyl transferase 73B3	2.592	7.19E-09	1.80E-06
RknMes02_011864			cassava4.1_029076m		2.577	2.23E-08	3.36E-06
RknMes02_045593					2.562	1.82E-07	9.95E-06
RknMes02_033982	AT3G54820.1	0	cassava4.1_011438m	plasma membrane intrinsic protein 2;5	2.527	1.23E-08	2.42E-06
RknMes02_020256	AT3G19270.1	0	cassava4.1_006983m	cytochrome P450, family 707, subfamily A, polypeptide 4	2.460	1.45E-06	3.39E-05
RknMes02_057698	AT2G45400.1	0	cassava4.1_032002m	NAD(P)-binding Rossmann-fold superfamily protein	2.443	2.17E-08	3.32E-06
RknMes02_042989	AT1G21240.1	5.00E-34	cassava4.1_022223m	wall associated kinase 3	2.397	5.65E-06	8.29E-05
RknMes02_052833	AT1G14870.1	1.40E-45	cassava4.1_023829m	PLANT CADMIUM RESISTANCE 2	2.391	7.66E-07	2.27E-05
RknMes02_051856	AT1G35910.1	0	cassava4.1_022149m	Haloacid dehalogenase-like hydrolase (HAD) superfamily protein///trehalose-6	2.382	2.03E-06	4.21E-05
RknMes02_056495			cassava4.1_030018m		2.361	2.35E-06	4.64E-05
RknMes02_058174	AT2G44990.1	0	cassava4.1_032749m	carotenoid cleavage dioxygenase 7	2.350	1.78E-07	9.87E-06
RknMes02_047242					2.348	1.41E-06	3.33E-05
RknMes02_048392	AT2G38940.1	0	cassava4.1_005715m	phosphate transporter 1;7///Unknown///phosphate transporter 1;5///phosphate t	2.340	6.58E-08	5.87E-06
RknMes02_052194	AT4G24970.1	0	cassava4.1_022738m	Histidine kinase-, DNA gyrase B-, and HSP90-like ATPase family protein	2.290	4.19E-08	4.63E-06
RknMes02_057133	AT2G18460.1	0	cassava4.1_031089m	like COV 3	2.270	7.93E-10	6.97E-07
RknMes02_007402	AT4G37870.1	0	cassava4.1_030131m	phosphoenolpyruvate carboxykinase 1	2.246	4.74E-08	4.92E-06
RknMes02_049851	AT3G22160.1	2.00E-14	cassava4.1_016216m	VQ motif-containing protein///Unknown	2.241	5.92E-06	8.55E-05
RknMes02_052318	AT1G65980.1	0	cassava4.1_022949m	thioredoxin-dependent peroxidase 1	2.240	2.58E-08	3.58E-06
RknMes02_004259	AT4G37870.1	9.00E-23	cassava4.1_033411m	phosphoenolpyruvate carboxykinase 1	2.217	2.39E-09	1.15E-06
RknMes02_047468					2.187	5.83E-07	1.88E-05
RknMes02_030435	AT4G37870.1	0	cassava4.1_033411m	phosphoenolpyruvate carboxykinase 1	2.157	1.39E-08	2.60E-06
RknMes02_035498	AT2G40080.1	8.00E-28	cassava4.1_019555m	Protein of unknown function (DUF1313)	2.148	6.24E-08	5.71E-06
RknMes02_052991	AT1G17020.1	0	cassava4.1_024085m	2-oxoglutarate (2OG) and Fe(II)-dependent oxygenase superfamily protein///se	2.146	1.39E-06	3.32E-05
RknMes02_016327	AT4G36470.1	7.00E-07	cassava4.1_010155m	S-adenosyl-L-methionine-dependent methyltransferases superfamily protein	2.114	1.99E-06	4.16E-05
RknMes02_017956	AT5G14230.1	0	cassava4.1_002222m	AT5G14230.1///"CONTAINS InterPro DOMAIN/s: Ankyrin repeat-containing	2.108	2.48E-06	4.78E-05
RknMes02_007372			cassava4.1_011919m		2.082	1.34E-06	3.23E-05
RknMes02_035809			cassava4.1_012760m		2.077	1.27E-08	2.45E-06
RknMes02_040157	AT5G65690.1	0	cassava4.1_030131m	phosphoenolpyruvate carboxykinase 2	2.069	3.50E-08	4.27E-06
RknMes02_050133	AT3G57950.1	4.00E-38	cassava4.1_017571m	AT3G57950.1///AT5G06790.1	2.060	5.89E-06	8.52E-05
RknMes02_014452	AT3G05500.1	1.00E-31	cassava4.1_028020m	Rubber elongation factor protein (REF)	2.051	8.41E-11	2.44E-07
RknMes02_049317	AT2G16050.1	2.90E-44	cassava4.1_013278m	Cysteine/Histidine-rich C1 domain family protein	2.042	9.60E-07	2.60E-05
RknMes02_004432	AT5G11420.1	2.00E-23	cassava4.1_010027m	Protein of unknown function, DUF642	2.037	5.92E-09	1.65E-06
RknMes02_006435	AT5G65690.1	1.00E-32	cassava4.1_030131m	phosphoenolpyruvate carboxykinase 2	2.034	7.84E-08	6.40E-06
RknMes02_054342	AT2G28420.1	0	cassava4.1_026393m	Lactoylglutathione lyase / glyoxalase I family protein	2.030	6.03E-07	1.92E-05
RknMes02_055022	AT4G33870.1	0	cassava4.1_027550m	Peroxidase superfamily protein	2.019	2.83E-09	1.18E-06
RknMes02_022851	AT3G47800.1	0	cassava4.1_025611m	Galactose mutarotase-like superfamily protein	2.005	2.62E-09	1.18E-06
RknMes02_051464	AT3G06240.1	1.00E-24	cassava4.1_021488m	F-box family protein///F-box and associated interaction domains-containing pr	2.002	1.78E-08	2.95E-06
RknMes02_043801					1.992	4.32E-08	4.73E-06
RknMes02_054294	AT1G67810.1	0	cassava4.1_026316m	sulfur E2	1.986	1.28E-09	8.44E-07
RknMes02_057805	AT3G24060.1	1.00E-13	cassava4.1_032165m	Plant self-incompatibility protein S1 family	1.985	3.68E-07	1.47E-05
RknMes02_057836	AT1G17020.1	0	cassava4.1_032207m	2-oxoglutarate (2OG) and Fe(II)-dependent oxygenase superfamily protein///se	1.967	7.69E-09	1.85E-06
RknMes02_057295	AT4G37770.1	0	cassava4.1_031356m	1-amino-cyclopropane-1-carboxylate synthase 8	1.909	2.55E-07	1.20E-05
RknMes02_019176	AT4G01580.1	2.00E-17	cassava4.1_013238m	AP2/B3-like transcriptional factor family protein	1.893	1.82E-06	3.93E-05
RknMes02_007011	AT3G01470.1	9.00E-31	cassava4.1_012760m	homeobox 1	1.889	1.46E-08	2.64E-06
RknMes02_051622	AT2G21790.1	0	cassava4.1_021751m	ribonucleotide reductase 1	1.885	3.61E-08	4.32E-06
RknMes02_006356	AT3G28510.1	0	cassava4.1_005875m	P-loop containing nucleoside triphosphate hydrolases superfamily protein	1.881	4.33E-06	6.93E-05
RknMes02_051348	AT2G28930.3	0	cassava4.1_021311m	protein kinase 1B	1.876	2.49E-06	4.80E-05
RknMes02_052167	AT5G24530.1	0	cassava4.1_022692m	2-oxoglutarate (2OG) and Fe(II)-dependent oxygenase superfamily protein	1.872	3.06E-06	5.48E-05
RknMes02_042934					1.864	2.20E-06	4.45E-05
RknMes02_011799	AT2G28090.1	2.00E-13	cassava4.1_011919m	Heavy metal transport/detoxification superfamily protein	1.847	2.36E-06	4.66E-05
RknMes02_056068	AT3G28880.1	3.00E-30	cassava4.1_029312m	Ankyrin repeat family protein	1.839	1.20E-06	3.00E-05
RknMes02_058273	AT1G35910.1	0	cassava4.1_032922m	Haloacid dehalogenase-like hydrolase (HAD) superfamily protein///trehalose-6	1.831	8.57E-07	2.43E-05
RknMes02_056535	AT5G53980.1	9.00E-34	cassava4.1_030094m	homeobox protein 52	1.826	3.01E-08	3.92E-06
RknMes02_048263	AT1G26610.1	5.00E-33	cassava4.1_004354m	C2H2-like zinc finger protein	1.821	7.20E-06	9.77E-05
RknMes02_048226	AT3G16380.1	0	cassava4.1_004058m	poly(A) binding protein 6	1.776	1.86E-07	1.01E-05
RknMes02_051165	AT2G36020.1	7.00E-25	cassava4.1_021018m	HVA22-like protein J	1.766	3.48E-06	5.98E-05
RknMes02_053638	AT1G19780.1	0	cassava4.1_025182m	cyclic nucleotide gated channel 8	1.754	6.34E-06	8.95E-05
RknMes02_057388	AT2G17570.1	0	cassava4.1_031506m	Undecaprenyl pyrophosphate synthetase family protein	1.749	1.11E-07	7.77E-06
RknMes02_044477					1.742	1.15E-06	2.92E-05
RknMes02_030263	AT1G80050.1	0	cassava4.1_016994m	adenine phosphoribosyl transferase 2	1.733	1.52E-09	9.44E-07
RknMes02_039442	AT5G55560.1	0	cassava4.1_012772m	Protein kinase superfamily protein	1.729	2.41E-08	3.43E-06
RknMes02_009054	AT4G35590.1	4.00E-31	cassava4.1_024901m	NIN like protein 7///RWP-RK domain-containing protein	1.720	1.94E-08	3.14E-06
RknMes02_050655	AT3G04710.3	1.00E-14	cassava4.1_019864m	ankyrin repeat family protein	1.708	6.33E-08	5.74E-06
RknMes02_037315	AT4G00330.1	4.00E-18	cassava4.1_032262m	calmodulin-binding receptor-like cytoplasmic kinase 2	1.688	8.00E-09	1.88E-06
RknMes02_038586	AT1G03220.1	2.00E-28	cassava4.1_007945m	Eukaryotic aspartyl protease family protein	1.677	2.13E-06	4.35E-05
RknMes02_057556	AT4G02170.1	2.00E-19	cassava4.1_031780m	AT4G02170.1	1.667	5.91E-06	8.54E-05
RknMes02_055668	AT5G55850.3	3.00E-19	cassava4.1_028651m	RPM1-interacting protein 4 (RIN4) family protein	1.661	1.15E-07	7.87E-06
RknMes02_054016	AT5G42120.1	0	cassava4.1_025847m	Concanavalin A-like lectin protein kinase family protein	1.645	3.96E-07	1.52E-05
RknMes02_034590	AT1G10340.2	4.00E-18	cassava4.1_004232m	Ankyrin repeat family protein	1.639	2.05E-07	1.06E-05
RknMes02_003389	AT4G15560.1	4.00E-39	cassava4.1_030091m	Deoxyxylulose-5-phosphate synthase	1.630	5.53E-09	1.60E-06
RknMes02_053766	AT4G33870.1	0	cassava4.1_025400m	Peroxidase superfamily protein	1.629	5.08E-07	1.74E-05
RknMes02_022677	AT4G25040.1	4.00E-12	cassava4.1_017297m	Uncharacterised protein family (UPF0497)///Unknown	1.626	2.33E-10	3.82E-07
RknMes02_008572	AT4G15417.1	3.00E-37	cassava4.1_031224m	RNAse II-like 1	1.625	7.85E-08	6.40E-06
RknMes02_051786	AT4G37630.2	7.00E-25	cassava4.1_022030m	cyclin d5;1	1.625	1.19E-06	2.99E-05

RknMes02_008825	AT2G36580.1	2.00E-12	cassava4.1_006316m	Pyruvate kinase family protein	1.598	4.36E-07	1.61E-05
RknMes02_038653	AT5G65280.1	2.00E-36	cassava4.1_008284m	GCR2-like 1	1.590	5.00E-07	1.72E-05
RknMes02_053434	AT4G22600.1	0	cassava4.1_024833m	AT4G22600.1	1.560	3.63E-07	1.46E-05
RknMes02_048720	AT1G03790.1	0	cassava4.1_008973m	Zinc finger C-x8-C-x5-C-x3-H type family protein	1.538	4.36E-08	4.76E-06
RknMes02_052907			cassava4.1_023951m		1.531	6.82E-06	9.43E-05
RknMes02_058917	AT1G23550.1	0	cassava4.1_033992m	similar to RCD one 2	1.526	5.41E-06	8.05E-05
RknMes02_020142	AT3G60670.1	0	cassava4.1_014925m	PLATZ transcription factor family protein	1.524	1.97E-07	1.04E-05
RknMes02_013107	AT4G00370.1	3.00E-39	cassava4.1_005826m	Major facilitator superfamily protein	1.522	4.13E-06	6.72E-05
RknMes02_054650	AT5G55850.3	9.00E-21	cassava4.1_026901m	RPM1-interacting protein 4 (RIN4) family protein	1.511	3.21E-06	5.66E-05
RknMes02_000403	AT2G40460.1	3.00E-30	cassava4.1_004356m	Major facilitator superfamily protein	1.502	3.73E-06	6.28E-05
RknMes02_034530	AT4G23810.1	8.00E-35	cassava4.1_010768m	WRKY family transcription factor///WRKY DNA-binding protein 30	1.500	2.97E-06	5.36E-05
RknMes02_023807	AT4G00330.1	6.00E-30	cassava4.1_031817m	calmodulin-binding receptor-like cytoplasmic kinase 2	1.495	2.15E-07	1.09E-05
RknMes02_026790	AT2G26870.1	0	cassava4.1_005538m	non-specific phospholipase C2	1.494	4.07E-12	6.01E-08
RknMes02_056377	AT2G32460.2	0	cassava4.1_029822m	myb domain protein 101	1.490	1.05E-06	2.76E-05
RknMes02_052535	AT3G09270.1	0	cassava4.1_023315m	glutathione S-transferase TAU 8///glutathione S-transferase tau 7	1.480	4.47E-07	1.63E-05
RknMes02_013610	AT3G52990.2	5.00E-17	cassava4.1_006316m	Pyruvate kinase family protein	1.479	1.09E-06	2.82E-05
RknMes02_003748			cassava4.1_017297m		1.470	5.35E-09	1.59E-06
RknMes02_010687	AT4G15560.1	6.00E-36	cassava4.1_030091m	Deoxyxylulose-5-phosphate synthase	1.467	1.57E-10	2.99E-07
RknMes02_031259	AT5G65280.1	0	cassava4.1_008284m	GCR2-like 1	1.465	2.72E-07	1.24E-05
RknMes02_050321	AT2G20515.1	2.80E-45	cassava4.1_018459m	AT2G20515.1	1.462	4.24E-07	1.59E-05
RknMes02_003488	AT3G54140.1	4.00E-14	cassava4.1_024513m	peptide transporter 1	1.447	1.99E-07	1.05E-05
RknMes02_015171			cassava4.1_020405m		1.442	6.33E-07	1.99E-05
RknMes02_000416	AT2G26870.1	0	cassava4.1_005538m	non-specific phospholipase C2	1.435	1.37E-11	1.20E-07
RknMes02_051546	AT3G58000.1	3.00E-30	cassava4.1_021620m	VQ motif-containing protein	1.435	2.22E-06	4.49E-05
RknMes02_048321	AT1G07180.1	0	cassava4.1_004993m	alternative NAD(P)H dehydrogenase 2///alternative NAD(P)H dehydrogenase	1.423	5.50E-07	1.82E-05
RknMes02_031518	AT2G39700.1	0	cassava4.1_014440m	expansin A9///expansin A4	1.412	5.77E-08	5.45E-06
RknMes02_052307	AT1G51080.1	0	cassava4.1_022931m	AT1G51080.1	1.410	2.63E-06	4.95E-05
RknMes02_037544	AT1G07180.1	1.00E-34	cassava4.1_005184m	alternative NAD(P)H dehydrogenase 2///alternative NAD(P)H dehydrogenase	1.403	3.79E-07	1.49E-05
RknMes02_031436	AT3G48700.1	0	cassava4.1_026915m	carboxyesterase 13	1.398	1.41E-06	3.34E-05
RknMes02_020730	AT4G21960.1	0	cassava4.1_012064m	Peroxidase superfamily protein	1.390	5.15E-06	7.80E-05
RknMes02_005354	AT3G53010.1	0	cassava4.1_012755m	Domain of unknown function (DUF303)	1.390	1.56E-07	9.23E-06
RknMes02_028619	AT4G11820.2	9.00E-24	cassava4.1_007081m	hydroxymethylglutaryl-CoA synthase / HMG-CoA synthase / 3-hydroxy-3-met	1.373	5.56E-06	8.19E-05
RknMes02_053760	AT3G20475.1	0	cassava4.1_025390m	MUTS-homologue 5	1.363	2.55E-10	3.87E-07
RknMes02_029441	AT5G24910.1	0	cassava4.1_005649m	cytochrome P450, family 714, subfamily A, polypeptide 1	1.360	2.05E-07	1.07E-05
RknMes02_050561	AT3G04620.1	2.00E-33	cassava4.1_019564m	Alba DNA/RNA-binding protein	1.360	1.61E-07	9.31E-06
RknMes02_038832	AT3G24310.1	0	cassava4.1_014512m	myb domain protein 305	1.358	1.85E-07	1.01E-05
RknMes02_032390	AT3G21760.1	0	cassava4.1_031478m	UDP-Glycosyltransferase superfamily protein///UDP-glucosyl transferase 71B(	1.356	1.67E-06	3.72E-05
RknMes02_048519	AT3G21760.1	0	cassava4.1_007027m	UDP-Glycosyltransferase superfamily protein///UDP-glucosyl transferase 71B(	1.348	6.93E-07	2.12E-05
RknMes02_049835	AT5G63130.1	8.00E-40	cassava4.1_016137m	Octicosapeptide/Phox/Bem1p family protein	1.341	7.90E-07	2.31E-05
RknMes02_009571	AT3G09550.1	8.00E-43	cassava4.1_007548m	Ankyrin repeat family protein	1.336	1.16E-06	2.93E-05
RknMes02_005365	AT2G37980.1	1.00E-14	cassava4.1_030087m	O-fucosyltransferase family protein	1.331	2.09E-07	1.07E-05
RknMes02_052720	AT1G25220.1	0	cassava4.1_023625m	anthranilate synthase beta subunit 1	1.330	1.43E-06	3.36E-05
RknMes02_032596	AT3G56400.1	2.00E-42	cassava4.1_013417m	WRKY DNA-binding protein 70	1.324	4.11E-07	1.56E-05
RknMes02_031388	AT5G65780.1	0	cassava4.1_012023m	branched-chain amino acid aminotransferase 5 / branched-chain amino acid tra	1.322	2.55E-10	3.87E-07
RknMes02_056248	AT4G39230.1	0	cassava4.1_029598m	NmrA-like negative transcriptional regulator family protein	1.322	1.25E-07	8.20E-06
RknMes02_021696	AT4G21960.1	0	cassava4.1_012064m	Peroxidase superfamily protein	1.318	2.46E-06	4.77E-05
RknMes02_038567					1.315	1.08E-06	2.80E-05
RknMes02_003889	AT3G04300.1	4.00E-36	cassava4.1_023553m	RmlC-like cupins superfamily protein	1.309	3.45E-07	1.42E-05
RknMes02_058926	AT2G29420.1	0	cassava4.1_034007m	glutathione S-transferase tau 7///glutathione S-transferase TAU 1	1.306	1.14E-06	2.90E-05
RknMes02_025204	AT1G09960.1	4.00E-33	cassava4.1_007925m	Unknown///sucrose transporter 4	1.301	6.83E-06	9.43E-05
RknMes02_015206	AT4G21960.1	1.00E-22	cassava4.1_012064m	Peroxidase superfamily protein	1.287	1.87E-06	4.00E-05
RknMes02_037144	AT3G45970.1	6.00E-17	cassava4.1_015495m	expansin-like A1	1.285	1.94E-06	4.11E-05
RknMes02_053679	AT2G35950.1	5.00E-07	cassava4.1_025248m	embryo sac development arrest 12	1.280	6.08E-06	8.71E-05
RknMes02_036024	AT5G64750.1	5.00E-38	cassava4.1_007311m	ethylene response factor 110///Unknown///Integrase-type DNA-binding superf	1.280	3.12E-06	5.55E-05
RknMes02_053494	AT1G72570.1	0	cassava4.1_024934m	Integrase-type DNA-binding superfamily protein	1.268	2.86E-08	3.79E-06
RknMes02_039434	AT4G34500.1	0	cassava4.1_026968m	Protein kinase superfamily protein///cassava4.1_017407m	1.267	3.79E-07	1.49E-05
RknMes02_039581	AT5G24910.1	0	cassava4.1_005649m	cytochrome P450, family 714, subfamily A, polypeptide 1	1.267	2.84E-07	1.27E-05
RknMes02_053618	AT5G08460.1	0	cassava4.1_025139m	GDSL-like Lipase/Acylhydrolase superfamily protein	1.265	4.71E-08	4.92E-06
RknMes02_023448			cassava4.1_009880m		1.265	2.50E-06	4.80E-05
RknMes02_042235			cassava4.1_013018m		1.261	2.32E-06	4.61E-05
RknMes02_031261	AT4G27410.2	0	cassava4.1_010999m	NAC (No Apical Meristem) domain transcriptional regulator superfamily prote	1.260	1.87E-07	1.01E-05
RknMes02_022969	AT5G24900.1	3.00E-34	cassava4.1_006808m	cytochrome P450, family 714, subfamily A, polypeptide 2	1.258	7.08E-07	2.15E-05
RknMes02_039767			cassava4.1_008879m		1.251	4.71E-07	1.67E-05
RknMes02_001771	AT3G48990.1	0	cassava4.1_005514m	Unknown///AMP-dependent synthetase and ligase family protein	1.240	5.13E-06	7.78E-05
RknMes02_005986	AT1G26190.1	2.00E-27	cassava4.1_003200m	Phosphoribulokinase / Uridine kinase family	1.237	5.03E-06	7.68E-05
RknMes02_025528	AT3G14440.1	0	cassava4.1_026283m	nine-cis-epoxycarotenoid dioxygenase 3	1.235	2.19E-06	4.44E-05
RknMes02_058666	AT3G48520.1	0	cassava4.1_033581m	cytochrome P450, family 94, subfamily B, polypeptide 3	1.234	5.58E-08	5.38E-06
RknMes02_053014	AT1G69850.1	0	cassava4.1_024123m	nitrate transporter 1:2///Major facilitator superfamily protein	1.230	1.02E-06	2.70E-05
RknMes02_021136					1.228	3.90E-06	6.49E-05
RknMes02_052689	AT5G16080.1	0	cassava4.1_023579m	carboxyesterase 17	1.224	1.59E-06	3.58E-05
RknMes02_049203	AT3G60390.1	0	cassava4.1_012590m	homeobox-leucine zipper protein 3///homeobox-leucine zipper protein 4	1.219	1.19E-09	8.41E-07
RknMes02_024400					1.215	5.85E-07	1.88E-05
RknMes02_003788	AT2G23970.1	9.00E-30	cassava4.1_023112m	Class I glutamine amidotransferase-like superfamily protein	1.208	3.68E-07	1.47E-05
RknMes02_009039	AT1G36940.2	2.00E-12	cassava4.1_022208m	unknown protein; FUNCTIONS IN: molecular function unknown; INVOLVE	1.205	1.12E-06	2.88E-05
RknMes02_032617	AT1G76490.1	2.00E-29	cassava4.1_003914m	hydroxy methylglutaryl CoA reductase 1	1.204	5.85E-06	8.47E-05
RknMes02_039445	AT2G41380.1	0	cassava4.1_014142m	S-adenosyl-L-methionine-dependent methyltransferases superfamily protein	1.195	3.28E-08	4.13E-06
RknMes02_003718	AT5G19530.2	4.00E-25	cassava4.1_011613m	S-adenosyl-L-methionine-dependent methyltransferases superfamily protein	1.194	4.91E-06	7.55E-05
RknMes02_053183	AT1G77400.1	5.00E-37	cassava4.1_024425m	AT1G77400.1	1.194	3.16E-06	5.61E-05
RknMes02_004737	AT3G14440.1	0	cassava4.1_026283m	nine-cis-epoxycarotenoid dioxygenase 3	1.183	2.84E-06	5.21E-05
RknMes02_055451	AT4G15630.1	0	cassava4.1_028267m	Uncharacterised protein family (UPF0497)	1.182	1.04E-06	2.74E-05
RknMes02_005512	AT1G76490.1	0	cassava4.1_003914m	hydroxy methylglutaryl CoA reductase 1	1.175	6.07E-07	1.93E-05
RknMes02_000900	AT4G34500.1	3.00E-24	cassava4.1_026968m	Protein kinase superfamily protein///cassava4.1_017407m	1.171	6.18E-09	1.67E-06
RknMes02_005864	AT5G02230.2	7.00E-08	cassava4.1_014451m	Haloacid dehalogenase-like hydrolase (HAD) superfamily protein	1.163	1.52E-06	3.49E-05
RknMes02_022679	AT4G40010.1	0	cassava4.1_024910m	SNF1-related protein kinase 2.7	1.163	5.15E-06	7.80E-05
RknMes02_011696	AT3G07720.1	7.00E-35	cassava4.1_011729m	Galactose oxidase/kelch repeat superfamily protein	1.162	5.73E-06	8.37E-05
RknMes02_003150	AT5G08790.1	2.00E-20	cassava4.1_012943m	NAC (No Apical Meristem) domain transcriptional regulator superfamily prote	1.156	1.46E-06	3.40E-05
RknMes02_026293	AT5G64410.1	0	cassava4.1_002179m	oligopeptide transporter 4	1.155	5.52E-06	8.16E-05
RknMes02_050840	AT4G22190.1	1.00E-06	cassava4.1_020395m	unknown protein; Has 283 Blast hits to 154 proteins in 44 species: Archae - 0;	1.155	4.52E-07	1.64E-05
RknMes02_010887	AT2G43590.1	2.00E-12	cassava4.1_014554m	Chitinase family protein	1.155	3.71E-06	6.27E-05
RknMes02_021249	AT1G76490.1	4.00E-30	cassava4.1_003914m	hydroxy methylglutaryl CoA reductase 1	1.154	2.96E-07	1.30E-05
RknMes02_042780					1.149	4.16E-06	6.75E-05
RknMes02_001717	AT5G08260.1	7.00E-15	cassava4.1_020819m	serine carboxypeptidase-like 35	1.142	1.21E-06	3.02E-05
RknMes02_024608	AT3G04620.1	0	cassava4.1_023523m	Alba DNA/RNA-binding protein	1.142	1.13E-06	2.89E-05
RknMes02_006744	AT3G48990.1	7.00E-42	cassava4.1_005514m	Unknown///AMP-dependent synthetase and ligase family protein	1.137	4.81E-06	7.44E-05
RknMes02_018836			cassava4.1_019524m		1.134	7.76E-09	1.85E-06
RknMes02_051435	AT3G28480.1	0	cassava4.1_021443m	Oxoglutarate/iron-dependent oxygenase	1.134	3.83E-06	6.40E-05
RknMes02_014511	AT4G02390.1	1.00E-11		poly(ADP-ribose) polymerase	1.133	5.80E-08	5.46E-06
RknMes02_009118	AT3G51240.2	6.00E-17	cassava4.1_010212m	flavanone 3-hydroxylase	1.132	1.82E-07	9.95E-06
RknMes02_033990	AT2G45120.1	0	cassava4.1_010237m	C2H2-like zinc finger protein	1.118	8.09E-08	6.48E-06
RknMes02_007712	AT3G16240.1	4.00E-13	cassava4.1_014710m	delta tonoplast integral protein///Unknown	1.115	5.44E-06	8.08E-05
RknMes02_049693			cassava4.1_015474m		1.112	8.58E-07	2.43E-05
RknMes02_029813			cassava4.1_013018m		1.110	1.06E-06	2.78E-05
RknMes02_017072	AT3G10340.1	0	cassava4.1_003117m	phenylalanine ammonia-lyase 2///phenylalanine ammonia-lyase 4	1.109	1.43E-06	3.35E-05
RknMes02_000130	AT3G60390.1	2.00E-37	cassava4.1_023832m	homeobox-leucine zipper protein 3///homeobox-leucine zipper protein 4	1.101	4.10E-08	4.57E-06
RknMes02_039325	AT5G54880.1	0	cassava4.1_034239m	DTW domain-containing protein	1.101	3.46E-07	1.42E-05
RknMes02_057524	AT1G65980.1	0	cassava4.1_031724m	thioredoxin-dependent peroxidase 1	1.099	1.47E-07	9.03E-06
RknMes02_041021	AT5G15190.2	4.00E-08	cassava4.1_019923m	AT5G15190.1///Unknown///"unknown protein; FUNCTIONS IN: molecular f	1.097	2.56E-06	4.87E-05
RknMes02_025971	AT1G43910.1	0	cassava4.1_006163m	P-loop containing nucleoside triphosphate hydrolases superfamily protein	1.095	2.05E-06	4.24E-05
RknMes02_023982	AT3G06760.1	2.00E-19	cassava4.1_016736m	Drought-responsive family protein	1.094	1.45E-06	3.38E-05
RknMes02_004885	AT2G14660.1	9.00E-37	cassava4.1_022246m	unknown protein; CONTAINS InterPro DOMAIN/s: Uncharacterised protein	1.092	3.58E-07	1.45E-05
RknMes02_000313	AT5G33370.1	3.00E-43		GDSL-like Lipase/Acylhydrolase superfamily protein	1.092	1.60E-06	3.61E-05
RknMes02_029387	AT5G67350.1	2.00E-38	cassava4.1_014725m	unknown protein; Has 1807 Blast hits to 1807 proteins in 277 species: Archae	1.088	1.44E-06	3.37E-05
RknMes02_011304	AT1G43910.1	5.00E-22	cassava4.1_006163m	P-loop containing nucleoside triphosphate hydrolases superfamily protein	1.087	1.17E-06	2.95E-05
RknMes02_049644	AT2G01890.1	0	cassava4.1_015221m	purple acid phosphatase 8///purple acid phosphatase 3	1.084	1.49E-06	3.44E-05
RknMes02_030183	AT3G52870.1	0	cassava4.1_014011m	IQ calmodulin-binding motif family protein	1.084	1.78E-06	3.88E-05
RknMes02_055949	AT5G08391.1	7.00E-27	cassava4.1_029115m	cassava4.1_033061m///Protein of unknown function (DUF 3339)	1.076	4.45E-08	4.82E-06
RknMes02_040089	AT5G07680.2	2.00E-30	cassava4.1_010869m	NAC domain containing protein 80///NAC domain containing protein 100	1.070	5.68E-08	5.42E-06
RknMes02_034595	AT1G34050.1	1.00E-34	cassava4.1_004232m	Ankyrin repeat family protein	1.070	1.11E-06	2.85E-05
RknMes02_004871	AT1G20270.1	2.00E-35	cassava4.1_013142m	2-oxoglutarate (2OG) and Fe(II)-dependent oxygenase superfamily protein	1.068	7.39E-06	9.96E-05
RknMes02_040160	AT1G04280.1	7.00E-24	cassava4.1_005863m	P-loop containing nucleoside triphosphate hydrolases superfamily protein	1.063	1.90E-07	1.02E-05
RknMes02_034385	AT1G54520.1	2.00E-34	cassava4.1_017125m	Unknown///unknown protein; FUNCTIONS IN: molecular_function unknown	1.061	5.16E-06	7.80E-05
RknMes02_014240	AT3G44220.1	7.00E-32		Late embryogenesis abundant (LEA) hydroxyproline-rich glycoprotein family	1.059	6.61E-06	9.23E-05

RknMes02_015088	AT1G13990.1	6.00E-18	cassava4.1_013257m	AT1G13990.1///unknown protein; FUNCTIONS IN: molecular_function unkr	1.057	3.65E-07	1.46E-05
RknMes02_058983	AT1G24530.1	0	cassava4.1_034104m	Transducin/WD40 repeat-like superfamily protein	1.054	2.37E-07	1.15E-05
RknMes02_035946	AT1G34300.1	1.00E-27	cassava4.1_001853m	receptor-like protein kinase 1///S-domain-2 5///lectin protein kinase family pro	1.051	7.96E-07	2.32E-05
RknMes02_049458	AT2G23620.1	0	cassava4.1_014110m	methyl esterase 1	1.051	2.52E-06	4.83E-05
RknMes02_057031	AT5G01260.2	1.00E-25	cassava4.1_030934m	Carbohydrate-binding-like fold	1.048	2.94E-06	5.33E-05
RknMes02_019499					1.047	1.57E-07	9.25E-06
RknMes02_054777	AT4G12570.1	7.00E-29	cassava4.1_027126m	ubiquitin protein ligase 5///MAPK/ERK kinase kinase 1	1.047	6.47E-07	2.02E-05
RknMes02_005842	AT1G04280.1	0	cassava4.1_005863m	P-loop containing nucleoside triphosphate hydrolases superfamily protein	1.044	1.58E-07	9.27E-06
RknMes02_021467	AT2G29420.1	2.00E-31	cassava4.1_026996m	glutathione S-transferase tau 7///glutathione S-transferase TAU 1	1.040	1.06E-06	2.77E-05
RknMes02_010313	AT5G62790.2	4.00E-14	cassava4.1_006908m	1-deoxy-D-xylulose 5-phosphate reductoisomerase	1.039	2.24E-09	1.12E-06
RknMes02_058899	AT1G02400.1	0	cassava4.1_033960m	gibberellin 2-oxidase 6	1.037	8.04E-07	2.33E-05
RknMes02_023158	AT5G02230.2	8.00E-31	cassava4.1_013061m	Haloacid dehalogenase-like hydrolase (HAD) superfamily protein	1.037	2.38E-06	4.68E-05
RknMes02_054995	AT5G19790.1	3.00E-24	cassava4.1_027506m	related to AP2 11	1.033	5.84E-06	8.46E-05
RknMes02_006698	AT4G21920.1	4.00E-08	cassava4.1_030675m	unknown protein; FUNCTIONS IN: molecular_function unknown; INVOLVE	1.033	4.51E-06	7.12E-05
RknMes02_024960	AT1G22130.1	7.00E-31	cassava4.1_015534m	AGAMOUS-like 104	1.032	2.11E-06	4.33E-05
RknMes02_038435			cassava4.1_012036m		1.030	6.10E-06	8.72E-05
RknMes02_010628			cassava4.1_001759m		1.028	2.87E-10	4.22E-07
RknMes02_049315	AT1G13990.1	0	cassava4.1_013257m	AT1G13990.1///unknown protein; FUNCTIONS IN: molecular_function unkr	1.026	1.81E-07	9.93E-06
RknMes02_026417	AT5G50330.1	0	cassava4.1_006600m	Protein kinase superfamily protein	1.024	8.51E-08	6.74E-06
RknMes02_001193	AT5G48820.1	8.00E-10	cassava4.1_032196m	Unknown	1.020	4.23E-06	6.83E-05
RknMes02_035613			cassava4.1_011649m		1.017	3.12E-09	1.24E-06
RknMes02_001491	AT1G79070.1	8.00E-17	cassava4.1_018156m	SNARE-associated protein-related	1.015	1.87E-07	1.01E-05
RknMes02_035449	AT3G14200.1	0	cassava4.1_014653m	Chaperone DnaJ-domain superfamily protein	1.015	1.16E-06	2.93E-05
RknMes02_017720	AT1G32690.1	6.00E-25	cassava4.1_022081m	unknown protein; FUNCTIONS IN: molecular_function unknown; INVOLVE	1.015	2.27E-08	3.36E-06
RknMes02_030292	AT5G08260.1	0	cassava4.1_010410m	serine carboxypeptidase-like 35	1.014	3.52E-07	1.43E-05
RknMes02_037229	AT1G67070.1	0	cassava4.1_007966m	Mannose-6-phosphate isomerase, type I	1.014	1.13E-06	2.89E-05
RknMes02_047156			cassava4.1_029851m		1.006	1.32E-06	3.20E-05
RknMes02_012429	AT1G12010.1	2.00E-08	cassava4.1_012052m	2-oxoglutarate (2OG) and Fe(II)-dependent oxygenase superfamily protein	1.003	1.63E-06	3.66E-05

[1]AGI code is shown if proteins encoded in each cassava gene (probe ID) have high amino acid sequence similarity (E value $\leq 10^{-5}$) to *Arabidopsis* homologs.

[2]E-value shows similarity in amino acid sequence between each cassava gene (probe ID) and *Arabidopsis* homolog.

[3]Encoded proteins/other features indicate the putative functions of the gene products that are expected from sequence similarity. The information for the NCBI protein reference sequence with the highest sequence similarity to the probes is shown.

TABLE S6. List of qRT-PCR primer sequences used for this research

Name	Forward primer	Reverse primer
MeSOS1	TTGGAGATGATGAGGAACTTGG	CTAGGGTCTACGCATCCTTCTA
MeAOC4	TTCTCCTACAGAAGCCAAACG	CTGGCTCAAGCGGAGATAAA
MePAL1	CCATGAGAAAGACCCACTTACA	GCTGCTCGGATAACCTCAAT
MeCAD6	GGCTCTTCTACCTACCCTCTT	CCAACCTTATCTCCCACCTTG
MeCOMT1	GGTGATTGTGGTGAGCCATA	CCCTCCAGGGAATAGAAACATC
MeActin	TTGCAGACCGTATGAGCAAG	AAGCACTTCCTGTGGACGAT

TABLE S7. Genes with significant fold changes (p < 0.0001) in at least one condition in cassava leaves

Probe ID	AGI code[1)]	Encoded proteins/other features[2)]	BH FDR
RknMes02_007648	AT5G37710	Alpha/beta-Hydrolases superfamily protein	7.50E-04
RknMes02_002053	AT2G38540	Lipid transporter protein 1	9.82E-04
RknMes02_028032			7.50E-04
RknMes02_006064	AT5G16390	Biotin carboxyl carrier protein 1	2.58E-04
RknMes02_004079	AT1G10550	Xyloglucan:Xyloglucosyl Transferase 33	3.33E-04
RknMes02_029964			8.17E-04
RknMes02_007746	AT1G27840	ATCSA-1	1.65E-04
RknMes02_022935	AT3G45880	2-oxoglutarate (2OG) and Fe(II)-dependent oxygenase s	8.17E-04
RknMes02_011421			1.65E-04
RknMes02_026460	AT3G05545	RING/U-box superfamily protein	7.63E-04
RknMes02_009157			3.57E-05
RknMes02_019550	AT1G31350	Kar-Up F-Box 1	4.02E-04
RknMes02_000675	AT2G39980	Hxxxd-type acyl-transferase family protein	1.30E-04
RknMes02_017165	AT5G37710	Alpha/beta-hydrolases superfamily protein	5.03E-04
RknMes02_003208	AT1G61100	Disease resistance protein (Tir Class)	1.30E-04
RknMes02_017818	AT2G03350	Unknown protein	5.03E-04
RknMes02_054108	AT1G72430	Small auxin upregulated RNA 78	6.27E-04
RknMes02_035380	AT1G07350	Serine/arginine rich-like protein 45A	8.17E-04
RknMes02_053207	AT2G17230	Exordium like 5	3.21E-04
RknMes02_006631	AT1G66180	Putative aspartyl protease (Asp).	4.26E-04
RknMes02_002817	AT4G37930	Serine hydroxymethyltransferase 1	1.30E-04
RknMes02_034638	AT5G67300	Arabidopsis thaliana Myb domain protein 44	3.33E-04
RknMes02_014068	AT5G25190	Ethylene and salt inducible 3	6.77E-04
RknMes02_020254	AT1G68400	Leucine-rich repeat transmembrane protein kinase famil	3.33E-04
RknMes02_009738	AT4G30920	Leucyl aminopeptidase 2	2.33E-04
RknMes02_030370	AT1G57680	Candidate G-protein coupled receptor 1	1.30E-04
RknMes02_011636	AT2G17230	Exordium like 5	4.26E-04
RknMes02_040138	AT2G17230	Exordium like 5	3.33E-04
RknMes02_056851	AT2G02760	Ubiquitin-conjugating enzyme 2	6.67E-04
RknMes02_031360	AT4G08950	Exordium	5.03E-04
RknMes02_031294	AT2G38310	Regulatory components of ABA receptor 10	6.32E-04
RknMes02_037398	AT2G31940	Unknown protein	9.66E-04
RknMes02_024329	AT3G46510	Arabidopsis Thaliana plant U-box 13	4.83E-04
RknMes02_002254	AT3G19680	Unknown protein	1.30E-04
RknMes02_045212			4.26E-04
RknMes02_014136	AT1G35140	Exordium like 1	6.67E-04
RknMes02_030091	AT4G37260	AtMYB73	4.26E-04
RknMes02_004641	AT4G08950	Exordium	1.30E-04
RknMes02_037055	AT5G37540	Eukaryotic aspartyl protease family protein	5.22E-04
RknMes02_021776	AT1G16810	Unknown protein	5.03E-04
RknMes02_026357	AT1G10020	Unknown protein	5.09E-04
RknMes02_028378	AT3G19680	Unknown protein	8.17E-04
RknMes02_050483	AT1G72430	Small auxin upregulated RNA 78	1.65E-04
RknMes02_025430	AT4G27430	Cop1-interacting protein 7	2.96E-04
RknMes02_049549	AT5G43830	Aluminium induced protein with Ygl And Lrdr Motifs	8.72E-04
RknMes02_002776	AT3G57800	Basic helix-loop-helix (BHLH) DNA-binding superfami	7.46E-04
RknMes02_009549	AT2G38540	Arabidopsis Thaliana lipid transfer protein 1	6.67E-04
RknMes02_026124	AT2G05160	CCCH-type zinc finger family protein with RNA-bindin	8.17E-04
RknMes02_053776	AT2G23690	Unknown protein	8.72E-04
RknMes02_021944	AT3G21330	Basic helix-loop-helix (BHLH) DNA-binding superfami	1.30E-04
RknMes02_058660	AT2G23690	Unknown protein	5.62E-04
RknMes02_011969	AT1G49890	QWRF domain containing 2	3.21E-04
RknMes02_026409			9.66E-04

[1)]The information for AGI locus ID with the highest sequence similarity with the probe is shown.

[2)]Encoded proteins/other features indicate the putative functions of the gene products that are expected from sequence similarity. The information for the NCBI protein reference sequence with the highest sequence similarity to the probes is shown.

TABLE S8. Hormone-related genes that upregulated by SAHA treatment during 0, 2 or 24 h NaCl incubation

Cassava ID	AGI code[1)]	log_2 ratio	Encoded proteins/other features[2)]	Related hormone signaling, responses and biosynthesis process
**Genes upregulated by SAHA treatment in the absence of NaCl ; log_2 ratio (SAHA 24 h/non-treated)**				
RknMes02_058115	AT3G07390	4.728	AUXIN-INDUCED IN ROOT CULTURES 12	response to auxin
RknMes02_057300	AT2G13810	4.524	AGD2-like defense response protein 1	salicylic acid mediated signaling pathway
RknMes02_057295	AT4G37770	4.083	1-amino-cyclopropane-1-carboxylate synthase 8	ethylene biosynthesis process
RknMes02_057486	AT2G21220	3.878	SAUR-like auxin-responsive protein family	response to auxin
RknMes02_028787	AT1G19640	3.117	jasmonic acid carboxyl methyltransferase	jasmonic acid biosynthetic process, jasmonic acid mediated signaling pathway
RknMes02_058825	AT1G33760	2.683	ERF022, ETHYLENE RESPONSE FACTOR022	ethylene-activated signaling pathway,
RknMes02_032596	AT3G56400	2.500	WRKY DNA-binding protein 70	jasmonic acid mediated signaling pathway
RknMes02_036024	AT5G64750	2.215	ethylene response factor 110///ABA represor 1	ethylene-activated signaling pathway, abscisic acid-activated signaling pathway
RknMes02_058899	AT1G02400	2.132	gibberellin 2-oxidase 6	gibberellin biosynthesis process
RknMes02_026274	AT5G40390	1.856	RAFFINOSE SYNTHASE 5, RS5, SEED IMBIBITION 1-LIKE, SIP1	response to abscisic acid
RknMes02_031261	AT4G27410	1.618	ATRD26, RD26, RESPONSIVE TO DESICCATION 26	response to abscisic acid
RknMes02_004082	AT1G17840	1.617	ABCG11, ARABIDOPSIS THALIANA WHITE-BROWN COMPLEX HOMOLOG PROTEIN 11	response to abscisic acid
RknMes02_051666	AT4G02570	1.549	cullin 1	involved in mediating responses to auxin and jasmonic acid.
RknMes02_003150	AT5G08790	1.285	ANAC081, ARABIDOPSIS NAC DOMAIN CONTAINING PROTEIN 81, ATAF2	response to Jasmonic acid and salicylic acid
RknMes02_001102	AT1G74650	1.237	myb domain protein 31///myb domain protein 96	response to gibberellin, response to salicylic acid
RknMes02_000236	AT1G09950	1.148	RESPONSE TO ABA AND SALT 1	response to abscisic acid
RknMes02_054005	AT5G57560	1.142	TCH4, TOUCH 4, XTH22, XYLOGLUCAN ENDOTRANSGLUCOSYLASE/HYDROLASE 22	response to auxin
RknMes02_010484	AT5G57050	1.127	ABA INSENSITIVE 2, ABI2,	abscisic acid-activated signaling pathway
RknMes02_024963	AT5G59220	1.105	HAI1, HIGHLY ABA-INDUCED PP2C GENE 1	negative regulation of abscisic acid-activated signaling pathway
RknMes02_006068	AT1G07430	1.046	HAI2, HIGHLY ABA-INDUCED PP2C GENE 2	negative regulation of abscisic acid-activated signaling pathway,
**Genes upregulated by SAHA treatment during 2 h NaCl incubation ; log_2 ratio {(NaCl 2 h after SAHA 24 h)/(NaCl 2 h after non-SAHA 24 h)}**				
RknMes02_057300	AT2G13810	7.348	AGD2-like defense response protein 1	salicylic acid mediated signaling pathway
RknMes02_051874	AT1G13280	5.966	allene oxide cyclase 4	jasmonic acid biosynthetic process
RknMes02_058115	AT3G07390	5.167	AUXIN-INDUCED IN ROOT CULTURES 12	response to auxin
RknMes02_020256	AT3G19270	3.985	CYTOCHROME P450, FAMILY 707, SUBFAMILY A, POLYPEPTIDE 4", CYP707A4	Abscisic acid catabolic process
RknMes02_057486	AT2G21220	3.613	SAUR-like auxin-responsive protein family	response to auxin
RknMes02_048392	AT2G38940	3.364	ATPT2, PHOSPHATE TRANSPORTER 1;4, PHT1;4	response to abscisic acid,
RknMes02_056377	AT2G32460	3.039	myb domain protein 101	gibberellic acid mediated signaling pathway, positive regulation of abscisic acid-activated signaling pathway
RknMes02_054668	AT4G36740	3.030	homeobox protein 40///homeobox protein 21	response to auxin
RknMes02_051666	AT4G02570	2.567	cullin 1	involved in mediating responses to auxin and jasmonic acid.
RknMes02_032596	AT3G56400	1.901	WRKY DNA-binding protein 70	jasmonic acid mediated signaling pathway
RknMes02_032098	AT4G37760	1.767	SQE3, SQUALENE EPOXIDASE 3	response to jasmonic acid
RknMes02_048095	AT2G28350	1.733	auxin response factor 10	auxin-activated signaling pathway
RknMes02_000236	AT1G09950	1.732	RESPONSE TO ABA AND SALT 1	ABA
RknMes02_037255	AT2G22430	1.678	homeobox protein 6	abscisic acid-activated signaling pathway, negative regulation of abscisic acid-activated signaling pathway,
RknMes02_049851	AT3G22160	1.667	JASMONATE-ASSOCIATED VQ MOTIF GENE 1, JAV1	Jasmonate regulated- plant defense
RknMes02_058666	AT3G48520	1.536	CYP94B3, CYTOCHROME P450, FAMILY 94, SUBFAMILY B, POLYPEPTIDE 3	jasmonic acid metabolic process
RknMes02_003150	AT5G08790	1.438	ANAC081, ARABIDOPSIS NAC DOMAIN CONTAINING PROTEIN 81, ATAF2	response to Jasmonic acid and salicylic acid
RknMes02_057295	AT4G37770	1.383	1-amino-cyclopropane-1-carboxylate synthase 8	ethylene biosynthesis process
RknMes02_029518	AT1G78380	1.361	glutathione S-transferase TAU 19///glutathione S-transferase TAU 22	Expression is induced by drought stress, oxidative stress, and high doses of auxin and cytokinin.
RknMes02_031259	AT5G65280	1.170	GCR2-like 1	ABA signal transduction
RknMes02_045249	AT1G21980	1.156	phosphatidylinositol-4-phosphate 5-kinase 1	Induced by ABA
RknMes02_058901	AT4G26200	1.137	1-amino-cyclopropane-1-carboxylate synthase 7	ethylene biosynthesis process
RknMes02_032589	AT2G46370	1.049	JAR1, JASMONATE RESISTANT 1	jasmonic acid mediated signaling pathway
**Genes upregulated by SAHA treatment during 24 h NaCl incubation ; log2 ratio {(NaCl 24 h after SAHA 24 h)/(NaCl 24 h after non-SAHA 24 h)}**				
RknMes02_058115	AT3G07390	5.000	AUXIN-INDUCED IN ROOT CULTURES 12	response to auxin
RknMes02_051874	AT1G13280	4.707	allene oxide cyclase 4	jasmonic acid biosynthetic process
RknMes02_057486	AT2G21220	4.596	SAUR-like auxin-responsive protein family	response to auxin
RknMes02_028787	AT1G19640	3.695	jasmonic acid carboxyl methyltransferase	jasmonic acid biosynthetic process, jasmonic acid mediated signaling pathway,
RknMes02_020256	AT3G19270	2.460	cytochrome P450, family 707, subfamily A, polypeptide 4	Abscisic acid catabolic process
RknMes02_057295	AT4G37770	1.909	1-amino-cyclopropane-1-carboxylate synthase 8	ethylene biosynthesis process
RknMes02_031259	AT5G65280	1.465	GCR2-like 1	ABA signal transduction
RknMes02_032596	AT3G56400	1.324	WRKY DNA-binding protein 70	jasmonic acid mediated signaling pathway
RknMes02_036024	AT5G64750	1.280	ethylene response factor 110///Unknown///Integrase-type DNA-binding superfamily protein	ethylene-activated signaling pathway
RknMes02_031261	AT4G27410	1.260	ATRD26, RD26, RESPONSIVE TO DESICCATION 26	response to abscisic acid
RknMes02_025528	AT3G14440	1.235	nine-cis-epoxycarotenoid dioxygenase 3	Abscisic acid biosynthetic process,
RknMes02_003150	AT5G08790	1.156	ANAC081, ARABIDOPSIS NAC DOMAIN CONTAINING PROTEIN 81, ATAF2	response to Jasmonic acid and salicylic acid

[1)]The information for AGI locus ID with the highest sequence similarity with the probe is shown.

[2)]Encoded proteins/other features indicate the putative functions of the gene products that are expected from sequence similarity. The information for the NCBI protein reference sequence with the highest sequence similarity to the probes is shown.

TABLE S9. Salt-responsive genes downregulated by SAHA treatment during 2 or 24 h NaCl incubation

Probe ID	AGI code[1)]	Encoded proteins/other features[2)]	BH FDR	log_2 ratio				
				w/o SAHA			with SAHA	
				2 h NaCl /0 h NaCl	24 h NaCl /0 h NaCl	SAHA 24 h/non-treated	(NaCl 2 h after SAHA 24 h)/(NaCl 2 h after non-SAHA 24 h)	(NaCl 24 h after SAHA 24 h)/(NaCl 24 h after non-SAHA 24 h)
RknMes02_002166	AT5G20250	Raffinose synthase family protein	4.79E-06	2.464	1.013	0.521	-1.245	0.001
RknMes02_003559	AT5G56870	beta-galactosidase 4	6.39E-05	2.653	0.487	-0.316	-1.231	-0.082
RknMes02_003707	AT4G26140	beta-galactosidase 12	1.79E-05	2.511	0.464	-0.337	-1.190	-0.114
RknMes02_003761	AT1G42430	unknown protein; BEST Arabidopsis thaliana protein match is: unk	4.93E-05	1.728	-0.038	-0.196	-1.333	-0.472
RknMes02_003801	AT3G13750	beta galactosidase 1///beta-galactosidase 12///beta-galactosidase 4	3.18E-05	2.407	0.267	-0.225	-1.140	0.022
RknMes02_004074	AT3G52800	A20/AN1-like zinc finger family protein	3.35E-05	1.108	-0.294	-0.203	-1.671	-0.041
RknMes02_004720	AT5G56870	beta-galactosidase 4	5.43E-05	2.562	0.362	-0.203	-1.283	-0.156
RknMes02_004901	AT3G52840	beta-galactosidase 2	5.27E-06	2.370	0.615	-1.029	-2.797	-1.125
RknMes02_005496	AT5G05140	Transcription elongation factor (TFIIS) family protein	6.28E-05	1.193	0.369	-0.173	-1.053	-0.073
RknMes02_006418	AT1G18100	PEBP (phosphatidylethanolamine-binding protein) family protein	6.48E-06	4.998	2.819	0.446	-1.826	0.228
RknMes02_007438	AT3G13750	beta galactosidase 1///beta-galactosidase 12///beta-galactosidase 4	7.26E-06	2.800	0.968	-0.715	-2.596	-1.178
RknMes02_008099	AT3G52840	beta-galactosidase 2	1.17E-05	2.608	0.288	-0.219	-1.316	0.010
RknMes02_008717	AT3G10030	aspartate/glutamate/uridylate kinase family protein	1.18E-05	1.163	0.482	-0.197	-1.049	-0.189
RknMes02_008877	AT4G31240	protein kinase C-like zinc finger protein	7.42E-06	5.793	5.162	1.266	-2.418	-0.244
RknMes02_008946	AT1G26580	FUNCTIONS IN: molecular_function unknown; INVOLVED IN: I	5.48E-05	1.295	0.744	-0.868	-1.484	0.154
RknMes02_009353	AT2G38760	annexin 3	3.07E-05	1.413	-0.011	-0.971	-1.654	-0.848
RknMes02_009448	AT1G05650	Pectin lyase-like superfamily protein	7.87E-05	4.101	1.808	-0.672	-2.815	-4.502
RknMes02_009918	AT4G08620	sulphate transporter 1;1	1.88E-05	1.311	-0.276	-1.059	-2.069	-0.644
RknMes02_009959			5.17E-05	1.328	0.388	-2.678	-3.043	-2.506
RknMes02_010945	AT5G15410	Cyclic nucleotide-regulated ion channel family protein	1.40E-05	2.041	1.973	-0.239	-1.356	-0.874
RknMes02_010968	AT4G33580	beta carbonic anhydrase 5	4.80E-05	2.172	0.334	-0.161	-1.265	-0.586
RknMes02_011243	AT1G23730	beta carbonic anhydrase 3	9.93E-06	2.292	0.708	-0.078	-1.190	-0.651
RknMes02_012078			5.24E-05	1.811	-0.044	-1.337	-2.171	-1.092
RknMes02_012263	AT3G10030	aspartate/glutamate/uridylate kinase family protein	5.99E-06	1.026	0.323	-0.265	-1.045	-0.110
RknMes02_012344	AT5G53450	OBP3-responsive gene 1	4.84E-05	2.017	0.558	-0.420	-1.055	-0.427
RknMes02_012445	AT2G36320	A20/AN1-like zinc finger family protein	1.52E-05	1.301	-0.457	-0.175	-1.710	-0.019
RknMes02_013897	AT3G13750	beta galactosidase 1///beta-galactosidase 12///beta-galactosidase 4	2.74E-06	2.634	0.415	-0.234	-1.275	-0.145
RknMes02_013918	AT5G14880	Potassium transporter family protein	4.47E-05	1.096	-0.439	-0.137	-1.551	-0.012
RknMes02_016270			8.91E-05	4.127	2.543	0.275	-1.240	-0.170
RknMes02_017023	AT4G16160	Mitochondrial import inner membrane translocase subunit Tim17/T	2.77E-05	5.332	5.118	-0.310	-2.687	-0.114
RknMes02_017212	AT3G22800	thiazole biosynthetic enzyme, chloroplast (ARA6) (THI1) (THI4)///	9.63E-05	2.861	0.648	0.146	-1.412	-0.208
RknMes02_018552			5.53E-05	1.083	0.830	-2.513	-1.531	-0.972
RknMes02_018780	AT5G52450.	MATE efflux family protein	7.75E-06	0.150	1.074	-1.224	-1.617	-1.048
RknMes02_020291	AT2G47600	magnesium/proton exchanger	1.46E-06	1.727	0.391	-0.820	-1.395	-0.917
RknMes02_021003	AT1G12845.	Unknown protein	1.59E-05	-1.085	1.023	-1.127	-0.624	-1.060
RknMes02_022438	AT4G26140	beta-galactosidase 12	7.56E-05	2.487	0.355	-1.571	-2.901	-1.618
RknMes02_023063			4.63E-05	1.060	1.059	-0.196	-1.192	-0.194
RknMes02_023600	AT1G45230	Protein of unknown function (DUF3223)	1.42E-05	2.621	0.829	0.140	-1.048	0.171
RknMes02_024117	AT1G27950	glycosylphosphatidylinositol-anchored lipid protein transfer 1	3.77E-05	1.179	1.884	0.447	-1.260	-0.251
RknMes02_024410	AT5G25350	EIN3-binding F box protein 2	6.20E-05	1.352	1.006	-0.040	-1.001	0.085
RknMes02_024529			1.34E-06	1.009	0.415	-0.337	-1.005	-0.330
RknMes02_024731	AT3G26770	Unknown///cassava4.1_020185m///NAD(P)-binding Rossmann-fol	2.48E-05	1.771	0.308	-1.199	-2.604	-1.368
RknMes02_024920	AT5G39000	Unknown///Malectin/receptor-like protein kinase family protein	2.82E-05	1.179	1.242	-0.836	-1.665	-0.687
RknMes02_025374	AT1G69840	SPFH/Band 7/PHB domain-containing membrane-associated protei	2.95E-06	2.735	1.514	0.192	-1.900	-0.954
RknMes02_025883	AT4G05120	Major facilitator superfamily protein	7.23E-06	4.053	1.290	0.627	-2.228	-0.477
RknMes02_026166	AT3G27010.	TEOSINTE BRANCHED 1, cycloidea, PCF (TCP)-domain family	1.12E-05	-0.052	1.010	-1.078	-1.144	-0.745
RknMes02_027432	AT3G60290	2-oxoglutarate (2OG) and Fe(II)-dependent oxygenase superfamily	4.50E-05	1.788	1.945	-0.471	-1.799	-0.404
RknMes02_027635			6.97E-07	1.486	-2.149	-0.931	-1.639	-1.149
RknMes02_027987	AT1G60420	DC1 domain-containing protein	5.27E-06	5.659	5.143	1.097	-2.384	-0.220
RknMes02_028547	AT2G32560	F-box family protein	4.16E-05	1.317	-0.402	0.246	-1.303	-0.009
RknMes02_029459			8.41E-05	1.120	0.122	-2.352	-3.870	-1.035
RknMes02_030412	AT4G26140	beta-galactosidase 12	2.44E-05	2.866	0.786	-0.455	-2.179	-0.914
RknMes02_030782	AT5G54585	Unknown	9.27E-06	1.163	0.557	-0.749	-1.138	-0.048
RknMes02_030872	AT1G56145	Leucine-rich repeat transmembrane protein kinase	4.20E-05	1.150	-0.503	-0.880	-1.471	-0.903
RknMes02_031789	AT1G68238	Unknown///AT1G68238.1	8.26E-06	2.285	-1.033	-0.178	-2.027	0.168
RknMes02_032772	AT1G53035	AT1G53035.1///unknown protein; FUNCTIONS IN: molecular_fu	9.53E-06	1.304	0.426	-0.343	-1.145	0.012
RknMes02_033342	AT4G30360	cyclic nucleotide-gated channel 17///cyclic nucleotide-gated channe	2.91E-06	1.568	0.812	-0.270	-1.187	-0.549
RknMes02_033442	AT2G43060	ILI1 binding bHLH 1	7.01E-05	1.270	0.195	-1.106	-1.513	-0.695
RknMes02_033587	AT5G26731	unknown protein; Has 30201 Blast hits to 17322 proteins in 780 sp	9.61E-06	1.339	0.956	-1.619	-1.708	-0.276
RknMes02_034218	AT2G28470	beta-galactosidase 8	1.91E-05	1.225	-0.524	-1.005	-1.949	-1.126
RknMes02_035812	AT1G17080	Ribosomal protein L18ae family	9.42E-05	1.168	-0.439	-0.535	-1.247	0.078
RknMes02_036203	AT2G47910	chlororespiratory reduction 6	1.88E-05	1.214	0.888	-1.003	-3.313	-1.327
RknMes02_036682	AT1G33420	RING/FYVE/PHD zinc finger superfamily protein	9.03E-06	1.284	0.308	-0.570	-1.730	-0.160
RknMes02_036778	AT5G28680	Malectin/receptor-like protein kinase family protein	1.37E-05	1.076	0.783	-0.829	-1.580	-0.677
RknMes02_037299	AT5G59520	ZRT/IRT-like protein 2	7.62E-05	1.346	-0.105	-2.240	-2.987	-3.235
RknMes02_037859			5.96E-06	0.558	1.336	-0.716	-1.608	-0.284
RknMes02_037895	AT1G34220	Regulator of Vps4 activity in the MVB pathway protein	2.78E-05	1.228	0.309	-0.410	-1.045	-0.363
RknMes02_038250	AT1G72200	RING/U-box superfamily protein	7.35E-05	2.261	-0.041	-1.152	-2.467	-0.605
RknMes02_039735	AT1G71120	GDSL-motif lipase/hydrolase 6	8.10E-06	2.449	1.936	-0.957	-1.747	-1.131
RknMes02_040383	AT5G53450	OBP3-responsive gene 1	1.01E-05	1.855	0.414	-0.519	-1.112	-0.464
RknMes02_040811			3.86E-05	0.748	1.533	-1.800	-2.411	-0.865
RknMes02_042045			1.49E-05	1.173	0.285	-0.747	-2.047	0.074
RknMes02_043315			6.19E-06	1.094	0.164	-0.803	-2.172	0.045
RknMes02_043452	AT5G05480	Peptide-N4-(N-acetyl-beta-glucosaminyl)asparagine amidase A pro	4.63E-06	2.229	2.358	-0.869	-2.027	-0.599
RknMes02_044688	AT4G25680	PPPDE putative thiol peptidase family protein///AT5G52220.2	9.54E-06	1.240	0.839	-1.075	-2.098	-0.903
RknMes02_045014			7.93E-06	1.253	0.925	-0.434	-2.263	0.533
RknMes02_046170	AT2G43870	Pectin lyase-like superfamily protein	1.12E-06	3.476	2.653	-5.166	-7.339	-7.585
RknMes02_046403			3.05E-05	1.640	0.606	-1.134	-2.145	-0.648
RknMes02_046476			3.31E-05	1.664	-0.714	-1.043	-1.872	-0.810
RknMes02_048319	AT4G29000.	Tesmin/TSO1-like CXC domain-containing protein	1.31E-06	0.893	1.058	-0.247	-1.000	-0.145
RknMes02_049259	AT1G28310	Dof-type zinc finger DNA-binding family protein	7.88E-06	2.697	0.718	-0.333	-1.717	-0.654
RknMes02_049295	AT2G06025	Acyl-CoA N-acyltransferases (NAT) superfamily protein	5.95E-05	1.237	-1.288	-0.154	-1.201	-0.153
RknMes02_049384	AT5G59480	Haloacid dehalogenase-like hydrolase (HAD) superfamily protein	1.72E-05	1.403	0.944	-1.466	-1.512	-2.881
RknMes02_049457	AT1G49320	unknown seed protein like 1	4.95E-06	2.994	-1.705	-3.661	-4.675	-3.567

RknMes02_049903	AT3G50830	cold-regulated 413-plasma membrane 2	2.16E-05	1.817	0.777	-0.816	-1.413	-0.401
RknMes02_049918	AT3G50830	cold-regulated 413-plasma membrane 2	6.61E-05	1.977	0.878	-0.715	-1.626	-0.501
RknMes02_050288	AT3G21510	histidine-containing phosphotransmitter 2///histidine-containing pho	2.44E-07	2.409	0.317	-0.468	-2.032	-0.781
RknMes02_050300	AT3G55646	AT3G55646.1///AT2G39855.2	5.27E-06	5.109	-1.299	-0.152	-1.668	-0.391
RknMes02_050301	AT5G66110	Heavy metal transport/detoxification superfamily protein	2.78E-05	4.508	2.356	0.948	-1.274	-0.483
RknMes02_050337	AT3G21510	histidine-containing phosphotransmitter 2///histidine-containing pho	9.15E-06	2.751	0.468	-0.259	-2.071	-0.781
RknMes02_050489	AT3G60370	FKBP-like peptidyl-prolyl cis-trans isomerase family protein	9.17E-05	2.208	-1.222	-0.025	-1.887	0.980
RknMes02_050840	AT4G22190	unknown protein; Has 283 Blast hits to 154 proteins in 44 species: .	1.20E-05	3.165	1.385	-0.427	-1.267	1.155
RknMes02_051011	AT3G48660	related to ABI3/VP1 2///Protein of unknown function (DUF 3339)	1.10E-05	3.298	2.245	0.327	-1.096	0.055
RknMes02_051310	AT5G14650	Pectin lyase-like superfamily protein	1.97E-05	3.625	0.505	-0.653	-4.006	-1.816
RknMes02_051397	AT4G35690	Arabidopsis protein of unknown function (DUF241)///Unknown	3.93E-05	4.043	1.827	-2.686	-3.415	-1.727
RknMes02_051417	AT1G19250	flavin-dependent monooxygenase 1	2.53E-05	1.286	0.393	-2.446	-3.289	-1.134
RknMes02_051493	AT3G07510	unknown protein; FUNCTIONS IN: molecular_function unknown;	1.74E-05	3.301	-1.152	-2.337	-4.603	-0.514
RknMes02_051689	AT4G35680	Arabidopsis protein of unknown function (DUF241)	5.84E-05	6.399	3.571	0.517	-1.653	-0.320
RknMes02_051939	AT2G41905	AT2G41905.1	2.99E-05	1.715	-0.424	-2.219	-4.171	-1.140
RknMes02_052131			5.99E-05	1.196	0.322	-2.297	-2.862	-0.787
RknMes02_052138	AT1G06620	2-oxoglutarate (2OG) and Fe(II)-dependent oxygenase superfamily	6.74E-06	1.410	0.645	-0.915	-1.791	-1.061
RknMes02_052192	AT4G35690	Arabidopsis protein of unknown function (DUF241)///Unknown	3.22E-05	4.412	0.208	-1.634	-1.602	0.142
RknMes02_052275	AT5G01350	AT5G01350.1///unknown protein; FUNCTIONS IN: molecular_fu	7.03E-05	1.090	1.694	-0.657	-1.078	-0.282
RknMes02_052293	AT3G52970	cytochrome P450, family 76, subfamily G, polypeptide 1///"cytochro	1.51E-05	1.755	-1.701	-0.334	-2.375	-2.026
RknMes02_052856	AT1G32583	AT1G32583.1	4.90E-05	3.002	0.988	0.185	-3.380	-0.680
RknMes02_052892			3.91E-05	1.318	1.003	-1.666	-2.109	-1.245
RknMes02_053085			2.12E-05	0.531	1.138	-0.739	-1.900	-0.296
RknMes02_053203	AT2G22590	UDP-Glycosyltransferase superfamily protein	6.16E-06	1.471	-0.775	-1.542	-2.091	-1.633
RknMes02_053251	AT2G41905	AT2G41905.1	6.81E-05	1.329	-0.457	-1.219	-3.180	-0.587
RknMes02_053380	AT2G19330	plant intracellular ras group-related LRR 6	1.79E-05	1.863	1.254	-2.351	-3.784	-3.136
RknMes02_053395	AT5G05840	Protein of unknown function (DUF620)	1.42E-06	1.465	-0.592	-1.554	-2.151	-1.060
RknMes02_053675	AT5G13910	Integrase-type DNA-binding superfamily protein	3.78E-05	4.010	-0.137	0.173	-2.984	-0.111
RknMes02_053712	AT1G15930	Ribosomal protein L7Ae/L30e/S12e/Gadd45 family protein	1.18E-06	1.742	-0.443	-0.085	-1.211	0.077
RknMes02_053786	AT2G24130	Leucine-rich receptor-like protein kinase family protein	9.64E-06	3.973	1.900	-0.499	-1.740	-0.007
RknMes02_053823	AT2G42850	cytochrome P450, family 718	3.66E-05	8.743	1.646	-0.274	-3.033	-1.996
RknMes02_054138	AT1G58350..	Putative serine esterase family protein	1.06E-05	0.726	1.932	-0.343	-2.112	-0.558
RknMes02_054305	AT3G57120	Protein kinase superfamily protein///Unknown	6.95E-05	1.340	-0.079	-4.300	-3.983	-4.020
RknMes02_054534	AT3G12160	RAB GTPase homolog A4D	2.47E-05	1.390	0.327	-1.100	-3.093	-1.934
RknMes02_054690	AT4G11360	RING/U-box superfamily protein	6.83E-05	5.531	1.054	-0.055	-3.651	-3.049
RknMes02_055023	AT4G25250	Plant invertase/pectin methylesterase inhibitor superfamily protein	8.55E-05	2.140	0.745	-1.993	-2.797	-6.263
RknMes02_055209	AT4G35720	Arabidopsis protein of unknown function (DUF241)	2.47E-06	3.021	0.622	-0.300	-1.881	-0.884
RknMes02_055268	AT4G25410	basic helix-loop-helix (bHLH) DNA-binding superfamily protein	9.04E-06	5.070	2.162	0.133	-3.248	-2.219
RknMes02_055373	AT3G55646	AT3G55646.1///AT2G39855.2	3.72E-05	5.518	-0.134	0.229	-2.009	-0.387
RknMes02_055484	AT5G60520	Late embryogenesis abundant (LEA) protein-related	1.77E-05	3.451	1.723	-7.143	-8.755	-9.060
RknMes02_055843	AT5G59190	Subtilisin-like serine endopeptidase family protein///subtilase family	1.88E-05	5.563	-0.241	0.088	-4.751	0.125
RknMes02_055888			1.62E-05	2.686	0.319	-3.285	-5.073	-3.605
RknMes02_056016	AT2G21490	dehydrin LEA	1.37E-05	2.763	1.568	-0.438	-1.664	-0.410
RknMes02_056263	AT2G34930.	disease resistance family protein / LRR family protein	1.18E-05	2.150	1.240	-2.482	-4.377	-2.258
RknMes02_056527	AT5G65300	AT5G65300.1	3.46E-05	2.097	1.121	-0.931	-2.380	-0.096
RknMes02_056665	AT5G20150	SPX domain gene 3///SPX domain gene 1	3.70E-05	1.257	1.161	-0.378	-1.873	-0.215
RknMes02_056689	AT3G59850	Pectin lyase-like superfamily protein	4.70E-05	4.766	2.922	-1.813	-4.406	-4.926
RknMes02_056706	AT1G11530	C-terminal cysteine residue is changed to a serine 1	5.28E-05	5.401	2.512	0.356	-4.488	-0.417
RknMes02_057053	AT1G06620	2-oxoglutarate (2OG) and Fe(II)-dependent oxygenase superfamily	2.26E-05	2.807	1.972	-0.626	-2.300	-0.914
RknMes02_057298	AT1G45616.	receptor like protein 7///receptor like protein 6///disease resistance f	5.30E-05	1.286	1.498	-1.725	-2.835	-1.815
RknMes02_057726			4.78E-05	4.203	-0.961	0.766	-2.291	-0.754
RknMes02_057990	AT2G21610	pectinesterase 11	9.90E-05	2.530	1.491	-5.255	-6.881	-7.404
RknMes02_058028	AT5G54570	beta glucosidase 41	4.16E-06	1.783	0.059	-1.932	-3.460	-1.567
RknMes02_058176	AT1G29670	GDSL-like Lipase/Acylhydrolase superfamily protein	7.78E-05	3.642	-0.636	-2.092	-3.196	-4.082
RknMes02_058439	AT1G65920	Regulator of chromosome condensation (RCC1) family with FYVE	9.90E-05	1.653	0.279	-0.827	-1.620	-1.093
RknMes02_058932	AT5G43870	Plant protein of unknown function (DUF828) with plant pleckstrin	2.24E-05	4.140	-0.539	-0.030	-2.304	0.288
RknMes02_059057	AT3G60220	RING/U-box superfamily protein///Unknown	3.94E-05	1.346	0.796	-1.056	-1.353	-0.060

[1]The information for AGI locus ID with the highest sequence similarity with the probe is shown.

[2]Encoded proteins/other features indicate the putative functions of the gene products that are expected from sequence similarity.

The information for the NCBI protein reference sequence with the highest sequence similarity to the probes is shown.
